# Addition of daratumumab to multiple myeloma backbone regimens significantly improves clinical outcomes: a systematic review and meta-analysis of randomised controlled trials

**DOI:** 10.1038/s41598-021-01440-x

**Published:** 2021-11-09

**Authors:** Szabolcs Kiss, Noémi Gede, Péter Hegyi, Bettina Nagy, Rita Deák, Fanni Dembrovszky, Stefania Bunduc, Bálint Erőss, Tamás Leiner, Zsolt Szakács, Hussain Alizadeh

**Affiliations:** 1 Doctoral School of Clinical Medicine, University of Szeged, Korányi fasor 8-10, Szeged, 6720 Hungary; 2 Institute for Translational Medicine, Medical School, University of Pécs, Szigeti Street 12, 2nd Floor, Pécs, 7624 Hungary; 3 Centre for Translational Medicine, Semmelweis University, Üllői Street 26, Budapest, 1085 Hungary; 4 Doctoral School, Carol Davila University of Medicine and Pharmacy, Bulevardul Eroii Sanitari 8, 050474 Bucureşti, Romania; 5 North West Anglia NHS Foundation Trust, Parkway Hinchingbrooke, Huntingdon, PE29 6NT UK; 6 Division of Haematology, First Department of Medicine, Medical School, University of Pécs, Ifjúság Street 13, Pécs, 7624 Hungary

**Keywords:** Cancer therapy, Haematological cancer, Cancer, Oncology, Cancer, Cancer therapy, Haematological cancer

## Abstract

Daratumumab has shown clinical benefit in multiple myeloma. We aimed to evaluate the safety and efficacy of adding daratumumab to backbone anti-myeloma treatments. Systematic search was performed up to August 2021 to identify randomised controlled trials comparing the outcomes of backbone therapy with and without daratumumab in relapsed/refractory and newly diagnosed myeloma (RRMM and NDMM, respectively). Odds ratios (ORs) and hazard ratios (HRs) were calculated with 95% confidence intervals (CIs). Primary outcomes were death or disease progression, minimal residual disease (MRD) negativity, and stringent complete response (sCR). Secondary outcomes were complete response or better and safety endpoints prespecified in the study protocol: PROSPERO (CRD42020222904). In NDMM, MRD negativity [OR = 3.61 (CI 2.33–5.61)] and sCR [OR = 2.29 (CI 1.49–3.51)] were more likely and death or disease progression [HR = 0.47 (CI 0.39–0.57)] was less likely to occur with daratumumab compared to control. Regarding RRMM, MRD negativity [OR = 5.43 (CI 2.76–10.66)] and sCR [OR = 3.08 (CI 2.00–4.76)] were more likely and death or disease progression was less likely [HR = 0.50 (CI 0.37–0.67)] with daratumumab compared to control. The addition of daratumumab has shown high clinical efficacy and acceptable toxicity profile for the treatment of NDMM and RRMM regarding the endpoints examined.

## Introduction

Multiple myeloma (MM) is the second most common haematologic malignancy, accounting for 1% of all cancers^1^. In the early 2000s, the approval of modern therapeutic options, such as immunomodulatory drugs (IMiD) and proteasome inhibitors (PI), greatly improved the relative survival rate in MM^2^. Although new innovative agents have brought a considerable breakthrough, this disease’s the treatment is still a challenging pursuit because of the frequent relapses. The 5-year survival rate is only 52.2%, despite the tremendous advances and continuous evolving therapeutic strategies^3^ Over the past decade, monoclonal antibodies were also proved to be an essential part of the therapeutic arsenal, especially in combination with the aforementioned novel agents^4^.

CD38 is overexpressed on myeloma cells’ surface, making this transmembrane glycoprotein a good target for immunotherapy in MM. Daratumumab is a CD38-targeted human IgG monoclonal antibody that exerts its antineoplastic effect through complement-dependent cytotoxicity, antibody-dependent cellular cytotoxicity and phagocytosis, programmed cell death after crosslinking, and inhibition of ectoenzyme function of CD38^5^.

This agent was approved as a monotherapy for relapsed MM by the Food and Drug Administration (FDA) and European Medicines Agency (EMA) in 2015 and 2016, respectively. Additionally, daratumumab is also indicated for combination therapy (e.g. with bortezomib, melphalan, and prednisone in newly diagnosed MM (NDMM) or with lenalidomide and dexamethasone in patients who have received at least one prior therapy^6^). Furthermore, new regimens including daratumumab are evaluated by ongoing clinical trials (NCT04288765, NCT03180736, NCT04649060, NCT03710603).

Since the FDA and EMA approved daratumumab, its incorporation into myeloma treatment regimens has significantly improved the outcomes of myeloma treatment including stringent complete response (sCR) and minimal residual disease (MRD) negativity, translating to prolonged progression-free survival (PFS) and overall survival (OS) with a relatively safe toxicity profile. Recent meta-analyses have assessed the safety and efficacy of daratumumab as an addition to backbone treatments in MM. Xu et al. found that addition of daratumumab to first-line regimens (bortezomib, melphalan, and prednisone or lenalidomide and dexamethasone) significantly improves PFS, compared to the same regimens alone, in NDMM. Furthermore, in their study, patients receiving daratumumab, compared to patients on the control arms, had a higher chance to achieve complete response (CR) rate or better^7^. Similar benefit of daratumumab was found by Wang et al. regarding CR or better in relapsed/refractory MM (RRMM)^8^. In another meta-analysis, Giri et al. revealed longer PFS in patients receiving daratumumab both in NDMM and RRMM regardless of cytogenetic risk^9^. The publication by Cao et al. focused on RRMM. Their results also suggested that daratumumab- based therapies enhance PFS in RRMM regardless of patient's baseline characteristics or previous therapeutic agents^10^. Another meta-analysis also investigated the impact of cytogenetic risk on the PFS benefit provided by the addition of daratumumab. They observed an increased PFS in RRMM patients with daratumumab; however, they did not reveal benefit of that agent in high genetic risk NDMM^11^. Similarly to that publication, the findings of two other studies could not support the survival benefit of daratumumab in high cytogenetic risk NDMM^12,13^.

Since these studies' publication, more evidence has become available, allowing us to re-evaluate these results performing meta-analytic calculations and to assess new endpoints such as sCR or MRD negativity. Furthermore, our objective was to resolve the discrepancies between the findings in previous reports. Besides these goals, our meta-analysis aims to summarise evidence on daratumumab containing backbone regimens' safety profile compared to the same combinations without daratumumab.

## Materials and methods

We report this study in accordance with the PRISMA 2020 statement: an updated guideline for reporting systematic reviews^14^. We fully adhered to our pre-study protocol registered in PROSPERO (CRD42020222904).

### Search strategy

We ran a systematic search in five electronic databases [MEDLINE (via PubMed), Embase, Web of Science, Cochrane Central Register of Controlled Trials (CENTRAL), and Scopus], dated from inception to August 3rd, 2021, with the query ‘(daratumumab) OR (humax-CD38) OR (humax-CD 38) OR (Darzalex) OR (anti-CD38) OR (antiCD38) OR (L01XC24) OR (945721–28-8) OR (DB09331) OR (4Z63YK6E0E) OR (D10777)’. No filter was applied. Reference lists of the eligible studies were also screened to identify relevant publications.

### Selection and eligibility criteria

Two independent review authors assessed all records at title, abstract, and full-text level. At each level of selection, inter-rater reliability was evaluated by calculating Cohen's kappa coefficient (κ)^15^. κ values ≤ 0 were interpreted as no agreement, 0.01–0.20 as none to slight agreement, 0.21–0.40 as fair agreement, 0.41–0.60 as moderate agreement, 0.61–0.80 as substantial agreement, 0.81–1.00 as almost perfect agreement, and 1.00 as a perfect agreement^15^. Disagreements have been resolved by third-party arbitration.

Only randomised controlled trials (RCTs) that compared the outcomes of a daratumumab containing regimen with the same treatment without daratumumab in patients with MM were eligible for inclusion. Furthermore, eligible studies had to provide data on least one of the following outcomes in both treatment arms: death or disease progression, sCR, CR or better, MRD negativity, thrombocytopenia, neutropenia, lymphopenia, anaemia, second primary malignancy, peripheral neuropathy, hypertension, cardiac failure, ischemic heart disease, acute renal failure. If more publication reported on the same trial (e.g. in case of CASTOR trial), the one that provided the most recent data was included in each outcome. If a study was not published in full-text article, the record was deemed ineligible for inclusion.

### Data extraction

Two independent review authors extracted the following data from the full text and corresponding supplementary information of eligible articles: study name, first author, year of publication, Digital Object Identifier (DOI), title, number of patients in the study and each treatment group, age and gender distribution, number of patients with the above-listed outcomes. For death or disease progression, hazard ratios (HRs) with the corresponding 95% confidence intervals (CIs) were extracted. If there were available data, we collected information on high- and standard cytogenetic risk subtypes. High cytogenetic risk subtype was defined as t(4;14) translocation, t(14;16) translocation, or 17p deletion and standard cytogenetic risk subtype as patients lacking all of these. Data on NDMM and RRMM were collected and analysed separately.

### Risk of bias assessment and certainty of the evidence

Two independent review authors evaluated the quality of the included studies using the RoB 2: a revised tool for assessing risk of bias in randomised trials^16^. The risk of bias assessment comprises five main domains: randomisation process, deviation from the intended intervention, missing outcome data, and selection of the reported results. These were rated as low risk, some concerns, or high risk of bias. Disagreements have been resolved by an independent third investigator.

Based on the approach proposed by the Grading of Recommendations, Assessment, Development and Evaluation (GRADE) Working Group, the certainty of the evidence was assessed by two review authors independently with the help of GRADE profiler software (GRADEpro GDT: GRADEpro Guideline Development Tool [Software]. McMaster University, 2020 (developed by Evidence Prime, Inc.). Available from gradepro.org.). Disagreements have been resolved by third-party arbitration.

### Statistical analysis

Pooled odds ratios (ORs) and hazard ratios (HRs) were calculated for dichotomous outcomes. A random-effect model was applied in all analyses with the DerSimonian–Laird estimation^17^. If there was an overlap between the two study populations, the study with the higher patient number was included in the analysis. Statistical heterogeneity was analysed using the I^2^ and χ^2^ tests to gain probability values; *p* < 0.10 was defined to indicate significant heterogeneity. The I^2^ test represents the percentage of total variability across studies because of heterogeneity. I^2^ values of 30–60%, 50–90%, and 75–100% corresponded to moderate, substantial, and considerable heterogeneity, respectively, based on Cochrane's handbook^18^. Forest plots displayed the results of the meta-analysis. Trial sequential boundaries for cumulative meta-analyses and the meta-analyses were performed with Stata 16 SE (Stata Corp).

### Ethics approval and consent to participate

Not required as data is not individualized and primary data was not collected. Not required as data is not individualized and primary data was not collected.

### Consent for publication

The corresponding author accepts responsibility for releasing this material on behalf of any and all co-authors.

## Results

### Systematic search and selection

**Figure 1:**
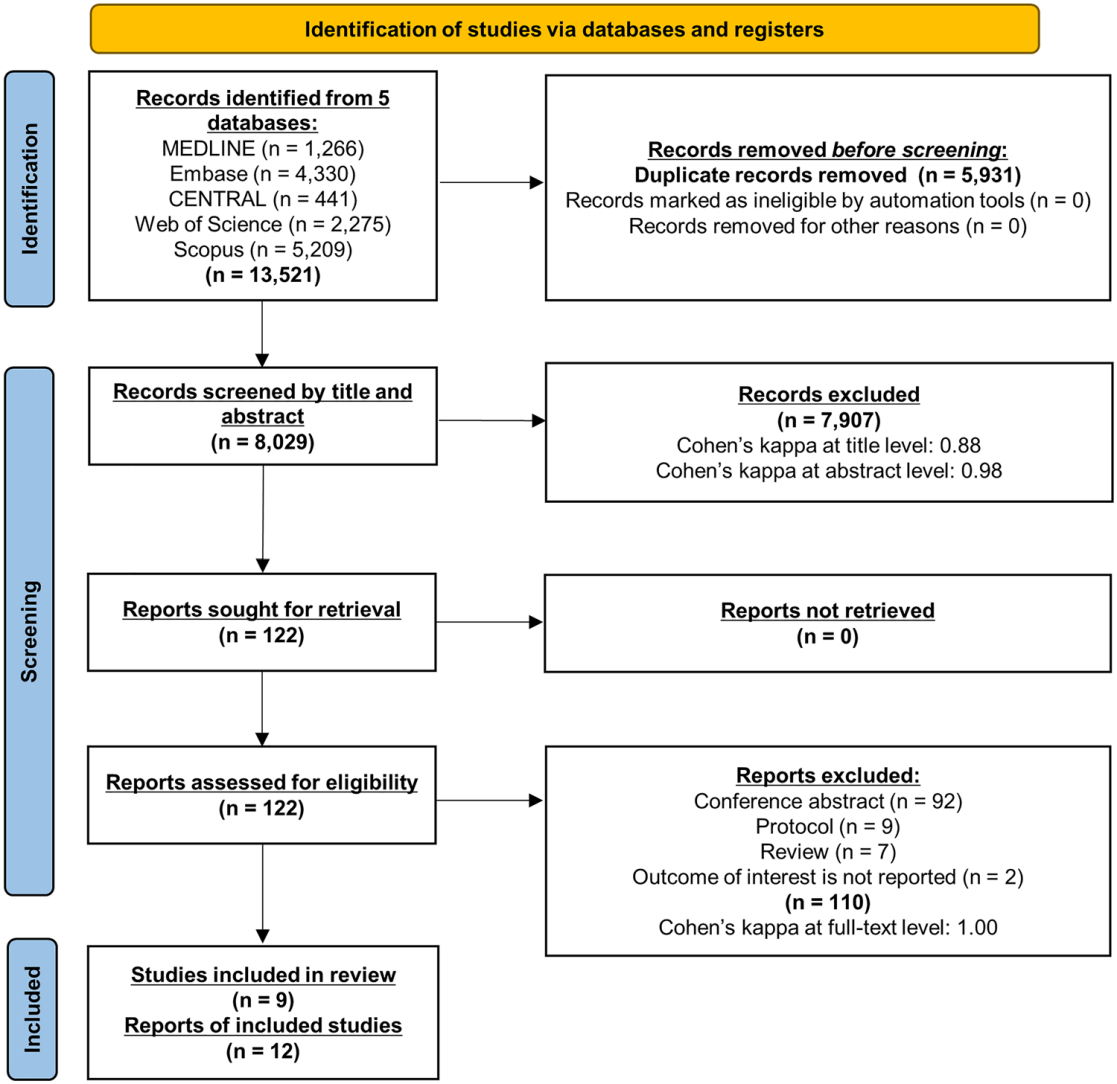
PRISMA Flow Diagram. This diagram details our systematic search and selection process.

A total of 13,521 records were identified, 12 of which proved to be eligible for inclusion in qualitative synthesis (Fig. 1)^19–30^. Reasons for exclusion regarding full-text assessment are provided in Appendix A. The inter-rater reliability was rated as almost perfect or perfect at all steps of selection.

### Characteristics of the studies included

Five and seven publications reported on patients with newly diagnosed and relapsed/refractory MM, respectively. The eligible papers reported on seven daratumumab containing regimens. The main characteristics of the studies included are summarised in Table 1.

**Table 1:** Characteristics of the studies included.

Study	Acronym and protocol registration number	Treatment allocation	No of patients	Age in years (median and range) and gender distribution (female%)		Follow-up period (median, in months)
				Daratumumab	Control	
**Studies reporting on newly diagnosed multiple myeloma regardless of cytogenetic risk**						
Facon et al.^22^	MAIA (NCT02252172)	DRd vs Rd	737	73 [50–90] (n.r.)	74 [45–89] (n.r.)	28.0
Moreau et al.^27^	CASSIOPEIA (NCT02541383)	DVTd vs VTd	1085	59 [22–65] (42%)	58 [26–65] (41%)	18.8
Mateos et al.^25^	ALCYONE (NCT02195479)	DVMP vs VMP	706	71 [40–93] (54%)	71 [50–91] (53%)	40.1
Voorhees et al.^29^	GRIFFIN (NCT02874742)	DRVd vs RVd	207	59 [29–70] (44%)	61 [40–70] (42%)	13.5
**Studies reporting on cytogenetic subgroups in newly diagnosed multiple myeloma**						
Mateos et al.^26^	ALCYONE (NCT02195479)	DVMP v. VMP	706	71 [40–93] (54%)	71 [50–91] (53%)	16.5
Facon et al.^22^	MAIA (NCT02252172)	DRd vs Rd	737	73 [50–90] (n.r.)	74 [45–89] (n.r.)	28.0
Moreau et al.^27^	CASSIOPEIA (NCT02541383)	DVTd vs VTd	1085	59 [22–65] (42%)	58 [26–65] (41%)	18.8
**Studies reporting on relapsed/refractory multiple myeloma regardless of cytogenetic risk**						
Dimopoulos et al.^20^	POLLUX (NCT02076009)	DRd vs Rd	569	73 [50–90] (n.r.)	74 [45–89] (n.r.)	13.5
Palumbo et al.^28^	CASTOR (NCT02136134)	DVd vs Vd	498	64 [30–88] (45%)	44 [33–85] (41%)	7.4
Dimopoulos et al.^19^	CANDOR (NCT03158688)	KdD vs Kd	466	64 [57–70]* (43%)	64.5 [59–71]* (41%)	17.2
Dimopoulos et al.^21^	APOLLO (NCT03180736)	DPd vs Pd	304	67 [42–86] (48%)	68 [35–90] (46%)	16.9
Lu et al.^24^	LEPUS (NCT03234972)	DVd vs Vd	211	61 [28–79] (40%)	61 [43–82] (40%)	8.2
**Studies reporting on cytogenetic subgroups in relapsed/refractory multiple myeloma**						
Dimopoulos et al.^19^	CANDOR (NCT03158688)	KdD vs Kd	466	64 [57–70]* (43%)	64.5 [59–71]* (41%)	17.2
Kaufman et al.^23^	POLLUX (NCT02076009)	DRd vs Rd	569	73 [50–90] (n.r.)	74 [45–89] (n.r.)	44.3
Weisel et al.^30^	CASTOR (NCT02136134)	DVd vs Vd	498	64 [30–88] (45%)	44 [33–85] (41%)	40.0
Dimopoulos et al.^21^	APOLLO (NCT03180736)	DPd vs Pd	304	67 [42–86] (48%)	68 [35–90] (46%)	16.9
Lu et al.^24^	LEPUS (NCT03234972)	DVd vs Vd	211	61 [28–79] (40%)	61 [43–82] (40%)	8.2

DPd, daratumumab, pomalidomide and dexamethasone; DRd, daratumumab, lenalidomide and dexamethasone; DRVd, daratumumab, bortezomib, lenalidomide and dexamethasone; DVd, daratumumab, bortezomib and dexamethasone; DVMP, daratumumab, bortezomib, melphalan, and prednisone; DVTd, daratumumab, bortezomib, thalidomide, and dexamethasone; Kd, carfilzomib and dexamethasone; KdD, carfilzomib, dexamethasone, and daratumumab; Pd, pomalidomide and dexamethasone; Rd, lenalidomide and dexamethasone; RVd, bortezomib, lenalidomide and dexamethasone; Vd, bortezomib and dexamethasone; n.r.; not reported; VMP bortezomib, melphalan, and prednisone; VTd, bortezomib, thalidomide, and dexamethasone.

*Interquartile range.

### Synthesis

#### Newly diagnosed multiple myeloma

##### Efficacy

Fig﻿ure﻿ 2 summarises the results of the meta-analyses. Death or disease progression were less likely to occur with daratumumab-containing regimens in overall (HR: 0.47, CI 0.39–0.57) and standard-risk MM (HR: 0.43, CI 0.35–0.53) compared to control treatment (all with statistical power ≥ 80%); however, we failed to reach the level of significance in high cytogenetic risk MM (with statistical power < 80%) (Fig. 3). MM patients receiving daratumumab-containing regimens were more likely to achieve CR or better (OR 2.14, CI 1.66–2.75), sCR (OR 2.29, CI 1.49–3.51), and MRD negativity (OR 3.61, CI 2.33–5.61) compared to control treatment, all with statistical power ≥ 80%.

**Figure 2:**
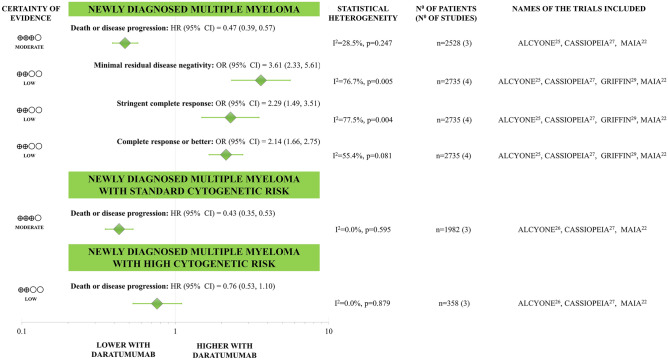
Clinical efficacy of backbone anti-myeloma regimens with and without daratumumab in newly diagnosed multiple myeloma.

**Figure 3:**
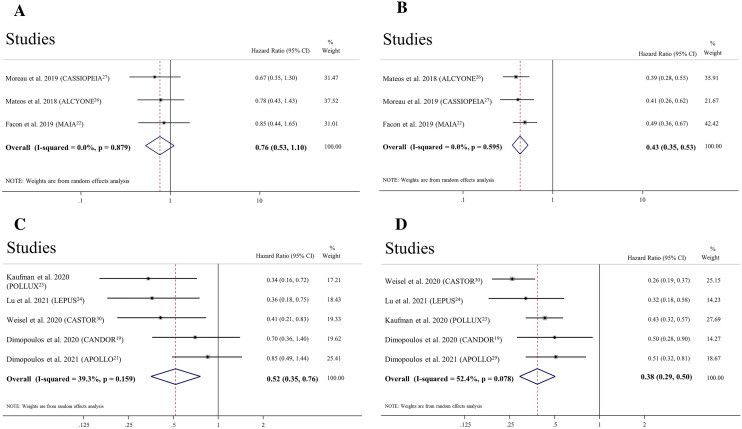
Effect of adding daratumumab to backbone anti-myeloma regimens on the risk of death of disease progression in (**A**) newly-diagnosed multiple myeloma with high cytogenetic risk, (**B**) newly-diagnosed multiple myeloma with standard cytogenetic risk, (**C**) relapsed/refractory multiple myeloma with high cytogenetic risk, and (**D**) relapsed/refractory multiple myeloma with standard cytogenetic risk.

##### Safety

**Figure 4:**
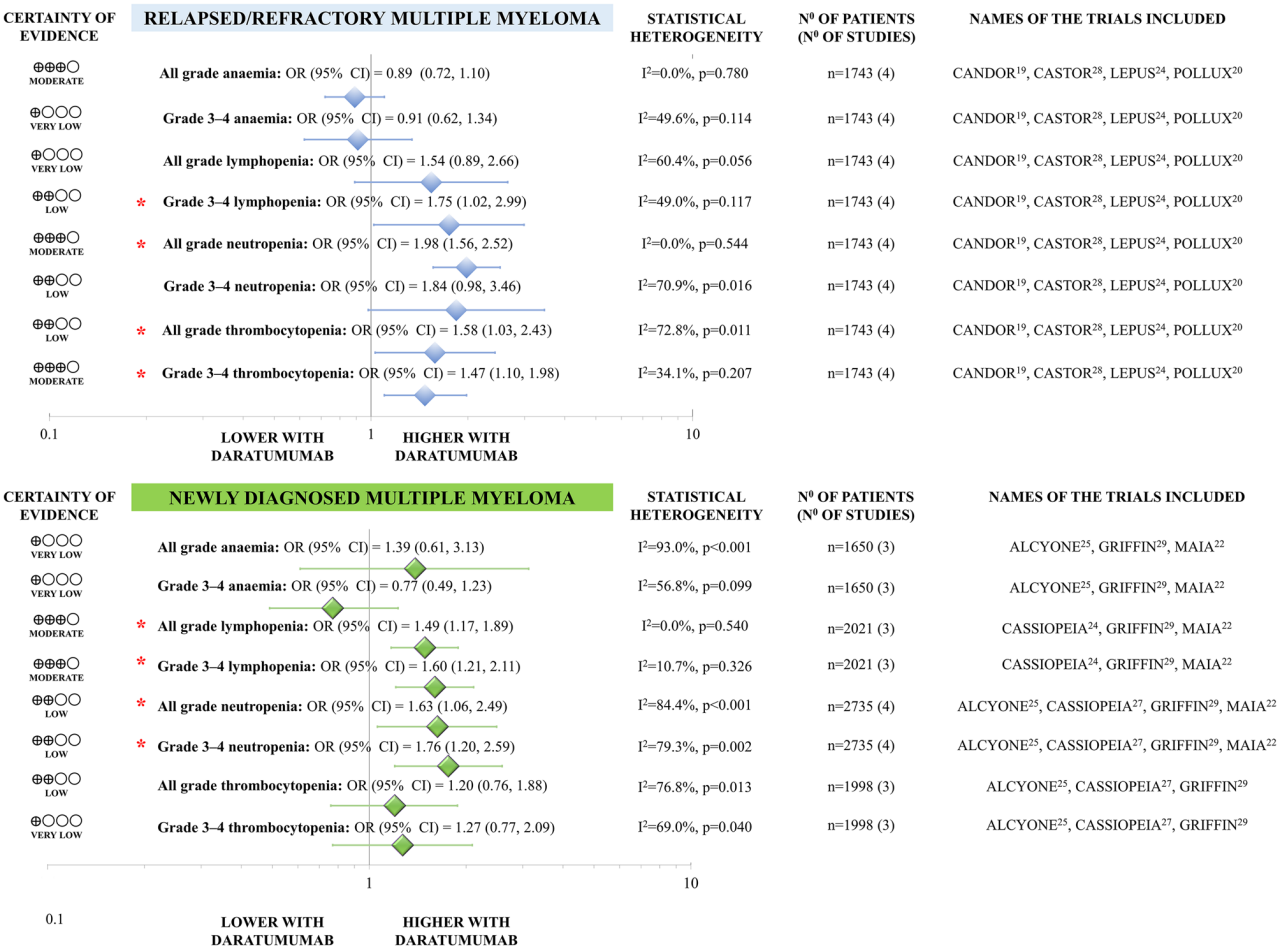
Haematological toxicity of daratumumab containing regimens compared to control in newly diagnosed and relapsed/refractory multiple myeloma.

Figure 4 summarises the results of meta-analyses on haematological toxicity. Incidence of anaemia and thrombocytopenia was not higher with daratumumab compared to the control group. On the other hand, lymphopenia and neutropenia occurred more frequently in the daratumumab group (all with statistical power ≥ 80% except for anaemia). Findings were consistent for grade 3–4 haematological toxicities. MM patients receiving daratumumab-containing treatment were less likely to develop peripheral neuropathy of any grade compared to control treatment (OR 0.76, CI 0.63–0.92, with statistical power ≥ 80%), whereas the frequency of grade 3–4 neuropathy was not significantly different between the groups (OR 0.80, CI 0.40–1.60, with statistical power < 80%). The second primary malignancy frequency was similar between the groups (OR 0.88, CI 0.54–1.45, with statistical power < 80%). No studies reported the other pre-specified outcomes, the risk of hypertension, acute cardiac and renal failure, and ischemic heart disease.

#### Relapsed/refractory multiple myeloma multiple myeloma

##### Efficacy

Death or disease progression were less likely to occur with daratumumab-containing regimens in overall (HR: 0.50, CI 0.37–0.67), standard-risk (HR: 0.38, CI 0.29–0.50), and high cytogenetic risk MM (HR: 0.52, CI 0.35–0.76) compared to the control treatment (all with statistical power ≥ 80%) (Fig. 3). MM patients receiving daratumumab-containing regimens were more likely to achieve CR or better (OR 3.50, CI 2.33–5.25), sCR (OR 3.08, CI 2.00–4.76), and MRD negativity (OR 5.43, CI 2.76–10.66) compared to control treatment, all with statistical power ≥ 80%.

##### Safety

Figure 3 summarises the results of the meta-analyses on haematological toxicity. Grade 3–4 lymphopenia, all grade neutropenia, and all grade and grade 3–4 thrombocytopenia were more common with daratumumab-containing vs control treatment (with statistical power ≥ 80%). We failed to detect a significant difference between the groups regarding other haematological toxicities. However, the comparison of all grade lymphopenia and grade 3–4 anaemia had less than 80% statistical power. We have not found difference in all grade (OR 2.21, CI 0.92–5.29, with statistical power ≥ 80%) and grade 3–4 hypertension (OR 3.21, CI 0.97–10.61, with statistical power ≥ 80%).

Data were insufficient for meta-analysis in the case of peripheral neuropathy and second primary malignancies (two non-overlapping study populations each); and in the case of acute cardiac failure, acute renal failure, ischemic heart disease (one non-overlapping population each) (shown in Table 2), and haematological toxicity in cytogenetic subgroups (two non-overlapping study populations each) (shown in Table 3).

**Table 2:** Non-haematological toxicity and second primary malignancy in relapsed/refractory multiple myeloma.

	Daratumumab-containing treatment (n/N, %)	Control treatment (n/N, %)
**All grade peripheral neuropathy**		
CANDOR study	53/308 (17%)	13/153 (8%)
CASTOR study	115/243 (47%)	89/237 (37%)
Substudy of CASTOR study		
Standard cytogenetic risk MM	67/137 (49%)	50/136 (37%)
High cytogenetic risk MM	22/40 (55%)	13/34 (38%)
**Grade 3–4 peripheral neuropathy**		
CANDOR study	0/308 (0%)	0/153 (0%)
CASTOR study	11/243 (4.5%)	16/237 (6.8%)
Substudy of CASTOR study		
Standard cytogenetic risk MM	4/137 (2.9%)	8/136 (5.9%)
High cytogenetic risk MM	2/40 (5.0%)	4/34 (12%)
**All grade hypertension**		
Substudy of CASTOR study		
Standard cytogenetic risk MM	15/137 (11%)	5/136 (3.7%)
High cytogenetic risk MM	4/40 (10%)	1/34 (2.9%)
Substudy of POLLUX study		
Standard cytogenetic risk MM	12/192 (6.3%)	8/176 (4.5%)
High cytogenetic risk MM	9/35 (25.7%)	2/34 (5.9%)
**Grade 3–4 hypertension**		
Substudy of CASTOR study		
Standard cytogenetic risk MM	9/137 (6.6%)	1/136 (0.7%)
High cytogenetic risk MM	2/40 (5%)	0/34 (0%)
Substudy of POLLUX study		
Standard cytogenetic risk MM	5/192 (2.6%)	2/176 (1.1%)
High cytogenetic risk MM	4/35 (11.4%)	0/34 (0%)
**Acute cardiac failure**		
CANDOR study	23/308 (7.5%)	16/153 (10%)
**Ischemic heart disease**		
CANDOR study	13/308 (4.2%)	5/153 (3.3%)
**Acute renal failure**		
CANDOR study	18/308 (5.8%)	12/153 (7.8%)
**Second primary malignancy**		
POLLUX study	8/286 (2.8%)	10/283 (3.6%)
CASTOR study	6/243 (2.5%)	1/237 (0.4%)

**Table 3:** Haematological toxicity in high and standard cytogenetic risk relapsed/refractory multiple myeloma (pooled results of the POLLUX and the CASTOR studies).

	Daratumumab-containing treatment (n/N, %)	Control treatment (n/N, %)
**All grade anaemia**		
Standard cytogenetic risk MM	116/329 (35.3%)	103/312 (33%)
High cytogenetic risk MM	20/75 (26.7%)	29/68 (42.6%)
**Grade 3–4 anaemia**		
Standard cytogenetic risk MM	56/329 (17%)	55/312 (17.6%)
High cytogenetic risk MM	11/75 (14.7)	16/68 (23.5%)
**All grade lymphopenia**		
Standard cytogenetic risk MM	28/329 (8.5%)	25/312 (8%)
High cytogenetic risk MM	9/75 (12%)	7/68 (10.3%)
**Grade 3–4 lymphopenia**		
Standard cytogenetic risk MM	22/329 (6.7%)	10/312 (3.2%)
High cytogenetic risk MM	8/75 (10.7%)	6/68 (8.8%)
**All grade neutropenia**		
Standard cytogenetic risk MM	145/329 (44.1%)	96/312 (30.8%)
High cytogenetic risk MM	31/75 (41.3%)	21/68 (30.9%)
**Grade 3–4 neutropenia**		
Standard cytogenetic risk MM	124/329 (37.7%)	75/312 (24.0%)
High cytogenetic risk MM	23/75 (30.7%)	18/68 (26.5%)
**All grade thrombocytopenia**		
Standard cytogenetic risk MM	140/329 (42.6%)	104/312 (33.3%)
High cytogenetic risk MM	37/75 (49.3%)	30/68 (44.1%)
**Grade 3–4 thrombocytopenia**		
Standard cytogenetic risk MM	90/329 (27.4%)	69/312 (22.1%)
High cytogenetic risk MM	27/75 (36%)	22/68 (32.4%)

### Risk of bias assessment and certainty of the evidence

The overall risk of bias was assessed as 'low risk' or 'some concern' for all studies. The most common reasons for 'some concern' assessments were the insufficient description of randomisation and allocation concealment processes or the lack of a statistical analysis plan. Detailed assessments for each endpoint are provided in Appendix A.

Certainty of evidence ranged between 'very low' and 'moderate'. A detailed assessment is shown in the GRADE evidence profile tables in Appendix B.

## Discussion

One of the main objectives of treatment in myeloma patients is to improve survival, both in NDMM and RRMM. In NDMM, the addition of daratumumab was associated with increased chance for PFS in each individual RCT and in our meta-analysis as well (moderate certainty). Regardless of these promising results, genetic risk stratification is a particularly important aspect of MM. About 15% of these patients carry myeloma with a high cytogenetic risk^31^. They are prone to worse therapeutic response, earlier relapse, and shorter PFS. While the survival benefit is still present in the standard cytogenetic risk population (moderate certainty), we have not found survival benefit in patients with high cytogenetic risk MM (low certainty).

The results about PFS benefit of daratumumab in high cytogenetic risk NDMM are controversial in the previous meta-analyses. We identified some possible explanations of this. Firstly, the paper of Giri et al.^9^, which had significant results in this analysis, incorporated their results from a conference abstract of the MAIA trial^32^ with a higher PFS benefit in the daratumumab arm compared to the one in other two publications^11,12^ and our paper. These three meta-analyses were more conservative and included only peer-reviewed full-text publications. Secondly, although all meta-analyses used the random effect model, the weights of the included studies slightly differ among the meta-analyses regarding this analysis. It has to be noted that the meta-analysis of Mohyuddin et al. did not reveal PFS benefit of adding daratumumab in high cytogenetic risk NDMM^13^; however that study did not use the HRs provided by the original publication and recalculated them from raw data. This resulted HRs that differed much from the ones calculated by the studies included. Thirdly, the four papers with neutral results used PFS values from more similar median follow up times (ALCYONE: 16.5 months; CASSIOPEIA: 18.8 months; MAIA: 28 months) and the one with significant PFS benefit in this population used HR from the MAIA trial with a median follow up of 36.4 months. This heterogeneity could also contribute to the difference between previous publications and reflect on the long term PFS benefit of daratumumab. However, regardless of the discrepancy in these findings, our TSA analysis confirmed that the statistical power was insufficient in this subgroup analysis, it seems to be early to preclude the benefit on this outcome.

In RRMM, PFS benefit was also observed in the daratumumab group (low certainty). This benefit was also found in the standard cytogenetic risk (low certainty) and in the high cytogenetic population (moderate certainty) as well. Therefore, our finding supports the incorporation of daratumumab regardless of the results of the cytogenetic assessment.

Several studies support that a better therapeutic response translates into longer PFS and OS in MM^33^. Based on this meta-analysis, patients on daratumumab have a better chance of achieving CR or better compared to control in NDMM (low certainty) and RRMM (moderate certainty). As modern therapeutic strategies led to deeper therapeutic response, sCR became an essential surrogate for survival endpoints^34^. Kapoor et al. found that patients achieving sCR had longer time to progression and longer OS after transplantation than those who only achieved CR, which underlines the prognostic significance of sCR^34^. Concerning sCR, we found that it is more likely to be achieved both in NDMM (low certainty) and RRMM patients (moderate certainty) receiving additional daratumumab compared to controls.

Detection of MRD is emerging as an important tool to assess the efficacy of treatments in MM^35^. Several studies have confirmed that MRD negativity is associated with improved survival both in patients with NDMM (ALCYONE, CASSIOPEIA, GRIFFIN, and MAIA trial) and RRMM (APOLLO, CANDOR, and LEPUS trial)^19,21,22,24,26–29^. In RRMM, MRD-negativity rates favoured daratumumab arm (moderate certainty). Regarding NDMM patients, the same associations were observed, and our pooled analyses support that the addition of daratumumab increases the chance of achieving MRD negativity (low certainty).

Despite the enhanced cytotoxicity on myeloma cells, better survival results, and therapeutic response, safety concerns arise^36^ as the target of this antibody is expressed on haematopoietic cells^37^. In NDMM, both all grade and grade 3–4 lymphopenia (moderate certainty) and neutropenia (low certainty) were more likely to appear in patients on daratumumab. These were consistent with our RRMM population results; however, the chance for all grade thrombocytopenia and grade 3–4 thrombocytopenia was also found to be increased (low certainty). Regarding the likelihood of anaemia, no difference was demonstrated.

Besides the bone marrow, other tissues, like peripheral and central neurons, also express CD38 which could raise clinical concern^37^. A previous meta-analysis demonstrated that the risk for peripheral neuropathy does not increase with the addition of daratumumab^36^. This study covered the literature until June 2019. Now all of their eligible studies have updated results; therefore, we could re-evaluate their findings and perform analyses on all grade and grade 3–4 neuropathy separately. Our results also support their finding.

With the increasingly longer survival of MM, second primary malignancies gained more significance^38^. In a population-based study of Sweden, the incidence of second primary malignancy was 5.5% after a median follow-up of 2.5 years^39^. These disorders mostly consist of mostly acute leukaemia or myelodysplasia and their incidence is about 2.19 times higher in MM compared to the general population^39^. Htut et al. found no increase in the incidence of second primary malignancies after the addition of daratumumab to backbone therapies^36^. We support their finding (very low certainty). However, as they pointed out, long-term follow-up results are required to confirm this observation.

This study has multiple strengths and limitations. First of all, the research was conducted with rigorous methodology adhering to the latest methodological recommendations and we reported our analyses transparently. Furthermore, our results are consistent with previous meta-analyses on the topic. Nevertheless, many of new publications on the subject emerged since these reviews were published, counting the longer follow-up data for the studies included in the precedent meta-analyses and the results of the GRIFFIN trial, the LEPUS trial, and the APOLLO trial^21,24,29^. This enabled us to re-evaluate their results and to assess new endpoints such as MRD negativity and sCR and to evaluate RRMM with subgroup analyses, which have not been included in a meta-analysis yet. There are limitations in this meta-analysis, including that different backbone regimens are evaluated in the individual RCTs. This issue was addressed by lowering the level of evidence due to indirectness in each evaluation. As the Trial Sequential Analyses have pointed out, some assessments are exposed to the possibility of imprecision. The overall risk of bias was generally ‘low’; however, certain domains had ‘some concern’ evaluation. Publication bias could not be assessed because the number of studies included in each analysis was insufficient for statistical analysis.

Implication for practice: our results support incorporating daratumumab in backbone therapies in MM which was associated with better therapeutic response and survival and favourable safety profile both in NDMM and RRMM.

Implication for science: additional studies are needed to specify further the population that gains the most benefits from this treatment, especially in high cytogenetic risk NDMM, where the OIS was not reached.

## Conclusion

Daratumumab has shown high clinical efficacy and acceptable toxicity profile for the treatment of both NDMM and RRMM for the endpoints examined.

## Supplementary Information

Supplementary Information 1.

Supplementary Information 2.

Supplementary Information 3.

## Data Availability

The datasets generated during and/or analysed during the current study are available from the corresponding author on reasonable request.

## Abbreviations

CENTRAL: Cochrane central register of controlled trials
CI: Confidence interval
CR: Complete response
DPd: Daratumumab, pomalidomide, and dexamethasone
DRd: Daratumumab, lenalidomide and dexamethasone
DRVd: Daratumumab, bortezomib, lenalidomide and dexamethasone
DVd: Daratumumab, bortezomib and dexamethasone
DVMP: Daratumumab, bortezomib, melphalan, and prednisone
DVTd: Daratumumab, bortezomib, thalidomide, and dexamethasone
GRADE: Grading of recommendations, assessment, development and evaluation
HR: Hazard ratio
IQR: Interquartile range
IRR: Infusion-related reaction
Kd: Carfilzomib and dexamethasone
KdD: Carfilzomib, dexamethasone, and daratumumab
MM: Multiple myeloma
MRD: Minimal residual disease negativity
NDMM: Newly diagnosed multiple myeloma
n.r.: Not reported
OIS: Optimal information size
OR: Odds ratio
OS: Overall survival
Pd: Pomalidomide and dexamethasone
PFS: Progression-free survival
PRISMA: Preferred reporting items for systematic reviews and meta‐analyses
RCT: Randomised controlled trial
Rd: Lenalidomide and dexamethasone
RRMM: Relapsed/refractory multiple myeloma
RVd: Bortezomib, lenalidomide and dexamethasone
sCR: Stringent complete response
TSA: Trial sequential analysis
Vd: Bortezomib and dexamethasone
VMP: Bortezomib, melphalan, and prednisone
VTd: Bortezomib, thalidomide, and dexamethasone
