# Supplementary material for: Addition of daratumumab to multiple myeloma backbone regimens significantly improves clinical outcomes: a systematic review and meta-analysis of randomised controlled trials
Source: Sci Rep. 2021 Nov 9;11:21916. doi: 10.1038/s41598-021-01440-x (PMC8578422; doi:10.1038/s41598-021-01440-x)
Supplement: Supplementary file 1 — Supplementary Information 1. [file 41598_2021_1440_MOESM1_ESM.pdf]

Appendix A

Summary: This document contains exclusion reasons for records of full-text assessment and additional forest plots not included in the main text. These forest plots are followed by the corresponding risk of bias assessment.

Addition of daratumumab to multiple myeloma backbone regimens: A systematic review and meta-analysis of randomised controlled trials

Szabolcs Kiss[1], Noémi Gede[2], Péter Hegyi[3], Bettina Nagy[2], Rita Deák[2], Fanni Dembrovszky[2], Stefania Bunduc[4], Bálint Erőss[2], Tamás Leiner[5], Zsolt Szakács[6 #], Hussain Alizadeh[6 # *]

[1]Doctoral School of Clinical Medicine, University of Szeged, Szeged, Hungary
Address: H-6720 Szeged, Korányi fasor 8-10., Hungary

[2]Institute for Translational Medicine, Medical School, University of Pécs, Pécs, Hungary
Address: H-7624 Pécs, Szigeti út 12. 2nd floor, Hungary

[3]Centre for Translational Medicine, Semmelweis University, Budapest, Hungary
Address: H-1085 Budapest, Üllői út 26., Hungary

[4]Doctoral school, Carol Davila University of Medicine and Pharmacy, Bucharest, Romania
Address: RO-050474 Bulevardul Eroii Sanitari 8, București, Romania

[5]North West Anglia NHS Foundation Trust, United Kingdom
Address: Parkway Hinchingbrooke, Huntingdon PE29 6NT, United Kingdom

[6]Division of Haematology, First Department of Medicine, Medical School, University of Pécs, Pécs, Hungary
Address: H-7624 Pécs, Ifjúság útja 13., Hungary

# equally contributed

*** Correspondence:**
Hussain Alizadeh M.D., Ph.D., Address: Division of Haematology, First Department of Medicine, Medical School, University of Pécs, H-7624 Pécs, Ifjúság Street 13., Hungary;
Tel: +36306436099
E-mail: alizadeh.hussain@pte.hu

Supplementary Table 1: Reasons of exclusion for individual studies in the full text assessment

Study		Reason for exclusion
Zweegman et al. 2019	Bortezomib, lenalidomide, and dexamethasone (VRd) Â± daratumumab (DARA) in patients (pts) with newly diagnosed multiple myeloma (NDMM) for whom transplant is not planned as initial therapy: a multicenter, randomized, phase III study (CEPHEUS)	Conference abstract
Weisel et al. 2020	Health-related quality of life outcomes from the phase 3 candor study in patients with relapsed or refractory multiple myeloma	Conference abstract
Weisel et al. 2018	Carfilzomib and dexamethasone versus 8 cycles of bortezomib and dexamethasone: An indirect comparison and exploratory analysis of the efficacy and safety of the randomized, phase 3 endeavor trial	Conference abstract
Weisel et al. 2019	Efficacy and safety of daratumumab, bortezomib, and dexamethasone (D-VD) versus bortezomib and dexamethasone (VD) in first relapse patients (pts) with multiple myeloma (mm): Four-year update of castor	Conference abstract
Weisel et al. 2017	Efficacy of daratumumab in combination with lenalidomide plus dexamethasone (DRd) or bortezomib plus dexamethasone (DVd) in relapsed or refractory multiple myeloma (RRMM) based on cytogenetic risk status	Conference abstract
Weisel et al. 2020	Carfilzomib, dexamethasone (KD) and daratumumab versus KD in relapsed or refractory multiple myeloma: Subgroup analysis of the candor study by number of prior lines of therapy and prior therapies	Conference abstract
Weisel et al. 2016	Phase 3 randomised study of daratumumab, bortezomib and dexamethasone (DVd) vs bortezomib and dexamethasone (Vd) in patients (pts) with relapsed or refractory multiple myeloma (RRMM): CASTOR	Conference abstract
Weisel et al. 2017	Efficacy and safety of daratumumab, bortezomib and dexamethasone (DVD) versus bortezomib and dexamethasone (VD) in relapsed or refractory multiple myeloma (RRMM): Updated analysis of castor	Conference abstract
Voorhees et al. 2019	Depth of response to daratumumab (DARA), lenalidomide, bortezomib, and dexamethasone (RVd) improves over time in patients (pts) with transplant-eligible newly diagnosed multiple myeloma (NDMM): Griffin study update	Conference abstract
Voorhees et al. 2017	Interim safety analysis of a phase 2 randomized study of daratumumab (Dara), Lenalidomide (R), Bortezomib (V), and Dexamethasone (d; Dara-Rvd) Vs. Rvd in patients (Pts) with newly diagnosed multiple myeloma (MM) eligible for high-dose therapy (HDT) and autologous stem cell transplantation (ASCT)	Conference abstract
Van Sanden et al. 2018	ADJUSTMENT FOR THE IMPACT OF SUBSEQUENT THERAPIES NOT AVAILABLE IN UK ON OVERALL SURVIVAL (OS) IN CASTOR TRIAL: A SUBGROUP ANALYSIS IN SECOND-LINE (2L) PATIENTS	Conference abstract
Usmani et al. 2019	Carfilzomib, dexamethasone, and daratumumab versus carfilzomib and dexamethasone for the treatment of patients with relapsed or refractory multiple myeloma (RRMM): Primary analysis results from the randomized, open-label, phase 3 study candor (NCT03158688)	Conference abstract

Supplementary Table 1: Reasons of exclusion for individual studies in the full text assessment (continued)

**Study**		**Reason for exclusion**
Usmani et al. 2019	Impact of age on efficacy and safety of daratumumab in combination with lenalidomide and dexamethasone (D-Rd) in patients (pts) with transplant-ineligible newly diagnosed multiple myeloma (NDMM): MAIA	Conference abstract
Usmani et al. 2016	Efficacy of daratumumab, lenalidomide, and dexamethasone versus lenalidomide and dexamethasone in relapsed or refractory multiple myeloma patients with 1 to 3 prior lines of therapy: Updated analysis of pollux	Conference abstract
Thein et al. 2019	Efficacy of upfront daratumumab combination regimen in patients with newly diagnosed multiple myeloma	Conference abstract
Spencer et al. 2017	Daratumumab, bortezomib, and dexamethasone (DVD) versus bortezomib and dexamethasone (VD) in relapsed or refractory multiple myeloma (RRMM): Updated efficacy and safety analysis of castor	Conference abstract
Sonneveld et al. 2019	Bortezomib, lenalidomide, and dexamethasone (VRd) ± daratumumab (DARA) in patients (pts) with transplant-eligible (TE) newly diagnosed multiple myeloma (NDMM): A multicenter, randomized, phase III study (PERSEUS)	Conference abstract
Sonneveld et al. 2019	Daratumumab Plus Bortezomib, Thalidomide, and Dexamethasone (D-VTd) in Transplant-eligible Newly Diagnosed Multiple Myeloma (NDMM): Subgroup Analysis of High-risk Patients (Pts) in CASSIOPEIA	Conference abstract
Shah et al. 2016	An open-label, randomised, phase 3 study of daratumumab, lenalidomide, and dexamethasone (DRd) versus lenalidomide and dexamethasone (Rd) in relapsed or refractory multiple myeloma (RRMM): POLLUX	Conference abstract
Shah et al. 2019	Daratumumab (DARA) plus lenalidomide versus lenalidomide alone as maintenance treatment in patients with newly diagnosed multiple myeloma (NDMM) after frontline autologous stem cell transplant (ASCT): Use of minimal residual disease (MRD) as a novel primary endpoint in the phase 3 auriga study	Conference abstract
Sebag et al. 2019	Lenalidomide plus bortezomib and dexamethasone in the treatment of newly diagnosed multiple myeloma: Results from a Canadian cost-effectiveness analysis	Conference abstract
San-Miguel et al. 2017	Efficacy by cytogenetic risk status for daratumumab in combination with lenalidomide and dexamethasone or bortezomib and dexamethasone in relapsed or refractory multiple myeloma	Conference abstract
San-Miguel et al. 2018	Daratumumab plus bortezomib-melphalan-prednisone (VMP) in elderly (≥75 years of age) patients with newly diagnosed multiple myeloma ineligible for transplantation (alcyone)	Conference abstract

Supplementary Table 1: Reasons of exclusion for individual studies in the full text assessment (continued)

Study		Reason for exclusion
Potluri et al. 2019	PCN254 AN INDIRECT COMPARISON OF ELOTUZUMAB, CARFILZOMIB, AND DARATUMUMAB WHEN GIVEN IN COMBINATION WITH POMALIDOMIDE AND DEXAMETHASONE FOR RELAPSED/REFRACTORY MULTIPLE MYELOMA	Conference abstract
Palumbo et al. 2016	Phase III randomized controlled study of daratumumab, bortezomib, and dexamethasone (DVd) versus bortezomib and dexamethasone (Vd) in patients (pts) with relapsed or refractory multiple myeloma (RRMM): CASTOR study	Conference abstract
Palumbo et al. 2016	Phase 3 randomised controlled study of daratumumab, bortezomib and dexamethasone versus bortezomib and dexamethasone in patients with relapsed or refractory multiple myeloma: Castor	Conference abstract
Moreau et al. 2017	Daratumumab, lenalidomide, and dexamethasone (DRD) versus lenalidomide and dexamethasone (RD) in relapsed or refractory multiple myeloma (RRMM) based on prior treatment history, renal function, and cytogenetic risk: Subgroup analyses of pollux	Conference abstract
Moreau et al. 2016	Efficacy of daratumumab, lenalidomide and dexamethasone versus lenalidomide and dexamethasone alone for relapsed or refractory multiple myeloma among patients with 1 to 3 prior lines of therapy based on previous treatment exposure: Updated analysis of pollux	Conference abstract
Moreau et al. 2019	Comparative efficacy and safety of bortezomib, thalidomide, and dexamethasone (VTD) without and with daratumumab (D-VTD) from cassiopeia versus VTD from PETHEMA/GEM in patients with newly diagnosed multiple myeloma using propensity score matching (PSM)	Conference abstract
Moreau et al. 2019	Phase 3 randomized study of daratumumab (DARA) + bortezomib/thalidomide/dexamethasone (D-VTd) vs VTd in transplant-eligible (TE) newly diagnosed multiple myeloma (NDMM): CASSIOPEIA Part 1 results	Conference abstract
Moreau et al. 2019	A Matching-adjusted Indirect Comparison (MAIC) of Bortezomib-Thalidomide-Dexamethasone (VTd) and Daratumumab Plus VTd (D-VTd) Versus Bortezomib-Dexamethasone (Vd) in Patients with Newly Diagnosed Multiple Myeloma (NDMM) who are Transplant Eligible (TE)	Conference abstract
Mateos et al. 2016	Efficacy of daratumumab, bortezomib, and dexamethasone versus bortezomib and dexamethasone in relapsed or refractory myeloma based on prior lines of therapy: Updated analysis of castor	Conference abstract
Mateos et al. 2017	Phase 3 randomized study of daratumumab plus bortezomib, melphalan, and prednisone (D-VMP) versus bortezomib, melphalan, and prednisone (VMP) in newly diagnosed multiple myeloma (NDMM) patients (Pts) ineligible for transplant (ALCYONE)	Conference abstract
Mateos et al. 2015	A randomized open-label study of bortezomib, melphalan, and prednisone (VMP) versus daratumumab (DARA) plus VMP in patients with previously untreated multiple myeloma (MM) who are ineligible for high-dose therapy: 54767414MMY3007 (Alcyone)	Conference abstract

Supplementary Table 1: Reasons of exclusion for individual studies in the full text assessment (continued)

Study		Reason for exclusion
Maiese et al. 2018	Cost per median month of progression-free survival for daratumumab plus bortezomib and dexamethasone compared with carfilzomib plus dexamethasone in relapsed/refractory multiple myeloma	Conference abstract
Lin et al. 2019	Daratumumab Plus Lenalidomide Versus Lenalidomide Alone as Maintenance Treatment in Patients With Newly Diagnosed Multiple Myeloma After Frontline Transplant: A Multicenter, Randomized, Phase 3 Study (AURIGA)	Conference abstract
Lentzsch et al. 2017	Daratumumab, bortezomib, and dexamethasone versus bortezomib and dexamethasone for relapsed/refractory multiple myeloma (RRMM) patients: An update of overall survival in castor	Conference abstract
Landgren et al. 2019	Weekly carfilzomib, lenalidomide, dexamethasone and daratumumab (wKRd-D) combination therapy provides unprecedented MRD negativity rates in newly diagnosed multiple myeloma: A clinical and correlative phase 2 study	Conference abstract
Kaufman et al. 2019	Four-year follow-up of the phase 3 pollux study of daratumumab plus lenalidomide and dexamethasone (D-RD) versus lenalidomide and dexamethasone (RD) alone in relapsed or refractory multiple myeloma (RRMM)	Conference abstract
Jenner et al. 2020	Tailoring treatment for patients with newly diagnosed high-risk myeloma - Feasibility results of the UKMRA OPTIMUM (MUKnine) trial	Conference abstract
Hungria et al. 2019	Comparison of daratumumab plus bortezomib, melphalan, and prednisone (D-VMP) with standard of care for patients from latin america with newly diagnosed multiple myeloma (NDMM) who were transplant ineligible: A propensity score matching analysis	Conference abstract
Hulin et al. 2019	Stem cell (SC) yield and transplantation results from transplant-eligible newly diagnosed multiple myeloma (TE NDMM) patients (pts) receiving daratumumab (DARA) + bortezomib/thalidomide/dexamethasone (D-VTd) in the phase 3 CASSIOPEIA study	Conference abstract
Huang et al. 2020	Phase 3 study of daratumumab/bortezomib/ dexamethasone (D-Vd) versus bortezomib/ dexamethasone (VD) in chinese patients (pts) with relapsed/refractory multiple myeloma (RRMM): MMY3009 (LEPUS)	Conference abstract
Htut et al. 2020	Efficacy of daratumumab combination regimen in patients with multiple myeloma: A combined analysis of six phase III randomised controlled trials	Conference abstract
Htut et al. 2019	Incidence of second primary malignancies and peripheral sensory neuropathy in patients with multiple myeloma receiving daratumumab containing regimen	Conference abstract

Supplementary Table 1: Reasons of exclusion for individual studies in the full text assessment (continued)

Study		Reason for exclusion
Giri et al. 2020	Impact of daratumumab in the treatment of multiple myeloma according to cytogenetic risk	Conference abstract
Gajra et al. 2019	Perceptions of community hematologists/oncologists on the potential of data presented at ASH 2018 and ASCO 2019 to alter the standard of care for multiple myeloma treatment	Conference abstract
Facon et al. 2018	Phase 3 randomized study of daratumumab plus lenalidomide and dexamethasone (D-Rd) versus lenalidomide and dexamethasone (Rd) in patients with newly diagnosed multiple myeloma (NDMM) ineligible for transplant (MAIA)	Conference abstract
Facon et al. 2015	Two randomized open-label studies of daratumumab (DARA) plus standard of care treatment versus standard of care alone in patients with previously untreated multiple myeloma (MM) ineligible for high-dose therapy: 54767414MMY3007 (Alcyone) and 54767414MMY3008 (Maia)	Conference abstract
Einsele et al. 2020	The B cell maturation antigen (BCMA) chimeric antigen receptor (CAR) T cell therapy idecabtagene vicleucel (IDE-CEL; bb2121) in relapsed and refractory multiple myeloma (RRMM): Outcomes from a phase 1 study support the phase 3 Karmma-3 study design to compare IDE-CEL versus standard triplet regimens	Conference abstract
Dimopoulos et al. 2017	Daratumumab, lenalidomide, and dexamethasone (DRD) versus lenalidomide and dexamethasone (RD) in relapsed or refractory multiple myeloma (RRMM): Updated efficacy and safety analysis of pollux	Conference abstract
Dimopoulos et al. 2017	Efficacy and safety of daratumumab, lenalidomide, and dexamethasone versus Rd alone in relapsed or refractory multiple myeloma: Updated analysis of pollux	Conference abstract
Cook et al. 2020	Four-year follow-up of the phase 3 POLLUX study of daratumumab plus lenalidomide and dexamethasone versus lenalidomide and dexamethasone alone in relapsed or refractory multiple myeloma	Conference abstract
Chiu et al. 2016	Daratumumab in combination with lenalidomide plus dexamethasone induces clonality increase and T-cell expansion: Results from a phase 3 randomized study (POLLUX)	Conference abstract
Charie et al. 2019	Daratumumab plus lenalidomide, bortezomib, and dexamethasone (d-RVd) Improves Depth Of Response In Transplant-Eligible Newly Diagnosed Multiple Myeloma: GRIFFIN primary analysis	Conference abstract
Chanan-Khan et al. 2016	Daratumumab, bortezomib and dexamethasone versus bortezomib and dexamethasone alone for relapsed or refractory multiple myeloma based on prior treatment exposure: Updated efficacy analysis of castor	Conference abstract

Supplementary Table 1: Reasons of exclusion for individual studies in the full text assessment (continued)

Study		Reason for exclusion
Cavo et al. 2018	Daratumumab plus Bortezomib-Melphalan-Prednisone (VMP) in elderly (≥75 y) patients (Pts) with newly diagnosed multiple myeloma (NDMM) ineligible for transplantation (ALCYONE)	Conference abstract
Cavo et al. 2018	Impact of baseline renal function on efficacy and safety of daratumumab plus bortezomibmelphalan- prednisone (VMP) in patients (Pts) with newly diagnosed multiple myeloma (NDMM) ineligible for transplantation (ALCYONE)	Conference abstract
Cavo et al. 2018	Impact of baseline renal function on efficacy and safety of daratumumab plus bortezomib-melphalan-prednisone (VMP) in patients (Pts) with newly diagnosed multiple myeloma (NDMM) ineligible for transplantation (ALCYONE)	Conference abstract
Cavo et al. 2018	Daratumumab plus Bortezomib-Melphalan-Prednisone (VMP) in elderly (≥75 y) patients (Pts) with newly diagnosed multiple myeloma (NDMM) ineligible for transplantation (ALCYONE)	Conference abstract
Bahlis et al. 2017	Daratumumab, lenalidomide, and dexamethasone (DRd) vs lenalidomide and dexamethasone (Rd) in relapsed or refractory multiple myeloma (RRMM): Efficacy and safety update (POLLUX)	Conference abstract
Bahlis et al. 2019	Randomized phase 2 study of subcutaneous daratumumab plus carfilzomib/dexamethasone versus carfilzomib/dexamethasone alone in patients with multiple myeloma who have been previously treated with intravenous daratumumab to evaluate retreatment (LYNX)	Conference abstract
Bahlis et al. 2019	Daratumumab plus lenalidomide and dexamethasone (D-RD) versus lenalidomide and dexamethasone (RD) in patients with newly diagnosed multiple myeloma (NDMM) ineligible for transplant: Updated analysis of maia	Conference abstract
Avet-Loiseau et al. 2019	Efficacy of daratumumab (DARA) + bortezomib/thalidomide/dexamethasone (D-VTd) in transplant-eligible newly diagnosed multiple myeloma (TE NDMM) based on minimal residual disease (MRD) status: Analysis of the CASSIOPEIA trial	Conference abstract
Avet-Loiseau et al. 2016	Evaluation of minimal residual disease (MRD) in relapsed/refractory multiple myeloma (RRMM) patients treated with daratumumab in combination with lenalidomide plus dexamethasone or bortezomib plus dexamethasone	Conference abstract
Avet-Loiseau et al. 2019	Concordance of Post-consolidation Minimal Residual Disease Rates by Multiparametric Flow Cytometry and Next-generation Sequencing in CASSIOPEIA	Conference abstract
Anderson et al. 2020	A matching-adjusted indirect comparison (MAIC) of progression-free survival between elotuzumab, daratumumab, and panobinostat triplet regimens for relapsed/refractory multiple myeloma	Conference abstract

Supplementary Table 1: Reasons of exclusion for individual studies in the full text assessment (continued)

Study		Reason for exclusion
Al Hadidi et al. 2019	Safety Analysis of Five Randomized Controlled Studies of Daratumumab in Patients with Multiple Myeloma	Conference abstract
Comenzo et al. 2020	Reduction in absolute involved free light chain and difference between involved and uninvolved free light chain is associated with prolonged major organ deterioration progression-free survival in patients with newly diagnosed al amyloidosis receiving bortezomib, cyclophosphamide, and dexamethasone with or without daratumumab: results from andromeda	Conference abstract
Cook et al. 2021	Four-year follow-up of the phase 3 POLLUX study of daratumumab plus lenalidomide and dexamethasone versus lenalidomide and dexamethasone alone in relapsed or refractory multiple myeloma	Conference abstract
Dimopoulos et al. 2020	Carfilzomib, dexamethasone, and daratumumab versus carfilzomib and dexamethasone in relapsed or refractory multiple myeloma: updated effiicacy and safety results of the phase 3 candor study	Conference abstract
Dimopoulos et al. 2020	Apollo: phase 3 randomized study of subcutaneous daratumumab plus pomalidomide and dexamethasone (D-PD) versus pomalidomide and dexamethasone (PD) alone in patients (PTS) with relapsed/refractory multiple myeloma (RRMM)	Conference abstract
Htut et al. 2019	Incidence of Second Primary Malignancies and Peripheral Sensory Neuropathy in Patients with Multiple Myeloma Receiving Daratumumab Containing Regimen	Conference abstract
Htut et al. 2019	Daratumumab-Related Hematological Toxicities in Patients with Multiple Myeloma: A Combined Analysis of Five Phase III Randomized Controlled Trials	Conference abstract
Huang et al. 2020	Phase 3 study of daratumumab/bortezomib/ dexamethasone (D-Vd) versus bortezomib/ dexamethasone (VD) in chinese patients (pts) with relapsed/refractory multiple myeloma (RRMM): MMY3009 (LEPUS)	Conference abstract
Kaufman et al. 2019	Four-Year Follow-up of the Phase 3 Pollux Study of Daratumumab Plus Lenalidomide and Dexamethasone (D-Rd) Versus Lenalidomide and Dexamethasone (Rd) Alone in Relapsed or Refractory Multiple Myeloma (RRMM)	Conference abstract
Kumar et al. 2020	Updated analysis of daratumumab plus lenalidomide and dexamethasone (D-RD) versus lenalidomide and dexamethasone(RD) in patients with transplant-ineligible newly diagnosedmultiple myeloma (NDMM): the phase 3 MAIA study	Conference abstract

Supplementary Table 1: Reasons of exclusion for individual studies in the full text assessment (continued)

Study		Reason for exclusion
Landgren et al. 2020	Evaluation of minimal residual disease (MRD) negativity inpatients with relapsed or refractory multiple myeloma treated inthe candor study	Conference abstract
Lu et al. 2020	Daratumumab, bortezomib, dexamethasone (D-VD) versusbortezomib and dexamethasone (vd) in relapsed or refractory(RR) multiple myeloma (MM): Pooled subgroup analysis of lepus and castor	Conference abstract
Quach et al. 2020	MM-128: carfilzomib, Dexamethasone, and Daratumumab (KdD) Versus Carfilzomib and Dexamethasone (Kd) in Relapsed or Refractory Multiple Myeloma (RRMM): subgroup Analysis of the Phase 3 CANDOR Study by Number of Prior Lines of Therapy (pLOTs) and Prior Therapies	Conference abstract
Shah et al. 2019	Daratumumab (DARA) Plus Lenalidomide Versus Lenalidomide Alone As Maintenance Treatment in Patients with Newly Diagnosed Multiple Myeloma (NDMM) after Frontline Autologous Stem Cell Transplant (ASCT): use of Minimal Residual Disease (MRD) As a Novel Primary Endpoint in the Phase 3 Auriga Study	Conference abstract
Voorhees et al. 2020	Depth of response to daratumumab, lenalidomide, bortezomib, and dexamethasone improves over time in patients with transplant-eligible newly diagnosed multiple myeloma: griffin study update (published in Blood)	Conference abstract
Voorhees et al. 2020	Depth of Response to Daratumumab (DARA), Lenalidomide, Bortezomib, and Dexamethasone (RVd) Improves over Time in Patients (pts) with Transplant-Eligible Newly Diagnosed Multiple Myeloma (NDMM): griffin Study Update (published in Blood marrow transplantation)	Conference abstract
Weisel et al. 2020	Carfilzomib, dexamethasone, and daratumumab versus carfilzomib and dexamethasone in relapsed or refractory multiple myeloma: subgroup analysis of the phase 3 candor study in patients with early or late relapse	Conference abstract
Weisel et al. 2020	Carfilzomib 56mg/m2 twice-weekly in combination withdexamethasone and daratumumab (KDD) versus daratumumab incombination with 8 cycles of bortezomib and dexamethasone(DVD); a matching-adjusted indirect treatment comparison	Conference abstract
Weisel et al. 2020	Carfilzomib, dexamethasone (KD) and daratumumab versus KD in relapsed or refractory multiple myeloma: subgroup analysis of the candor study by number of prior lines of therapy and prior therapies	Conference abstract
Weisel et al. 2020	Health-related quality of life outcomes from the phase 3 candor study in patients with relapsed or refractory multiple myeloma	Conference abstract

Supplementary Table 1: Reasons of exclusion for individual studies in the full text assessment (continued)

Study		Reason for exclusion
Weisel et al. 2019	Efficacy and Safety of Daratumumab, Bortezomib, and Dexamethasone (D-Vd) Versus Bortezomib and Dexamethasone (Vd) in First Relapse Patients (pts) with Multiple Myeloma (MM): four-Year Update of Castor	Conference abstract
Zweegman et al. 2021	Daratumumab plus lenalidomide and dexamethasone (D-RD) versus lenalidomide and dexamethasone (rd) in transplantineligible newly diagnosed multiple myeloma (NDMM): frailty subgroup analysis of Maia	Conference abstract
Bahlis et al. 2019	Daratumumab Plus Lenalidomide and Dexamethasone (D-Rd) Versus Lenalidomide and Dexamethasone (Rd) in Patients with Newly Diagnosed Multiple Myeloma (NDMM) Ineligible for Transplant: updated Analysis of Maia	Conference abstract
Al Hadidi et al. 2019	Safety analysis of five randomized controlled studies of daratumumab in patients with multiple myeloma	Conference abstract
Bahlis et al. 2019	Randomized Phase 2 Study of Subcutaneous Daratumumab Plus Carfilzomib/Dexamethasone Versus Carfilzomib/Dexamethasone Alone in Patients with Multiple Myeloma Who Have Been Previously Treated with Intravenous Daratumumab to Evaluate Retreatment (LYNX)	Protocol
-	Efficacy of Daratumumab (dara) retreatment using a histone deacytelase-inhibitor (HDACi: panobinostat) as a dara-longevity-inducing, epigenetic agent in combination with bortezomib-dexamethasone as a quadruplet in relapsed / refractory multiple myeloma (RRMM) patients	Protocol
Euctr, S. E. 2014	A Study Comparing Daratumumab, Lenalidomide, and Dexamethasone with Lenalidomide and Dexamethasone in Relapsed or Refractory Multiple Myeloma	Protocol
Euctr, H. U. 2015	A Study of Combination of Daratumumanb and Velcade (Bortezomib) Melphalan-Prednisone (DVMP) Compared to Velcade Melphalan-Prednisone (VMP) in Participants with Previously Untreated Multiple Myeloma	Protocol
Euctr, G. R. 2018	A Study of combination of Daratumumab, VELCADE (bortezomib), Lenalidomide, and Dexamethasone (D-VRd) compared to VELCADE, Lenalidomide, and Dexamethasone (VRd) in participants with Previously Untreated Multiple Myeloma	Protocol
Euctr, F. R. 2019	IFM 2017-03	Protocol

Supplementary Table 1: Reasons of exclusion for individual studies in the full text assessment (continued)

Study		Reason for exclusion
Al Hadidi et al. 2019	Safety analysis of five randomized controlled studies of daratumumab in patients with multiple myeloma	Conference abstract
Bahlis et al. 2019	Randomized Phase 2 Study of Subcutaneous Daratumumab Plus Carfilzomib/Dexamethasone Versus Carfilzomib/Dexamethasone Alone in Patients with Multiple Myeloma Who Have Been Previously Treated with Intravenous Daratumumab to Evaluate Retreatment (LYNX)	Protocol
-	Efficacy of Daratumumab (dara) retreatment using a histone deacytelase-inhibitor (HDACi: panobinostat) as a dara-longevity-inducing, epigenetic agent in combination with bortezomib-dexamethasone as a quadruplet in relapsed / refractory multiple myeloma (RRMM) patients	Protocol
Euctr, S. E. 2014	A Study Comparing Daratumumab, Lenalidomide, and Dexamethasone with Lenalidomide and Dexamethasone in Relapsed or Refractory Multiple Myeloma	Protocol
Euctr, H. U. 2015	A Study of Combination of Daratumumanb and Velcade (Bortezomib) Melphalan-Prednisone (DVMP) Compared to Velcade Melphalan-Prednisone (VMP) in Participants with Previously Untreated Multiple Myeloma	Protocol
Euctr, G. R. 2018	A Study of combination of Daratumumab, VELCADE (bortezomib), Lenalidomide, and Dexamethasone (D-VRd) compared to VELCADE, Lenalidomide, and Dexamethasone (VRd) in participants with Previously Untreated Multiple Myeloma	Protocol
Euctr, F. R. 2019	IFM 2017-03	Protocol
Euctr, E. S. 2018	A Clinical Study to Compare Daratumumab, VELCADE (bortezomib), Lenalidomide, and Dexamethasone (D-VRd) with VELCADE, Lenalidomide, and Dexamethasone (VRd) in Subjects with Untreated Bone Marrow Cancer and for Whom Hematopoietic Stem Cell Transplant is Not Planned as Initial Therapy	Protocol
Euctr, B. E. 2017	A Randomized, Open-label, Phase 3 Study Comparing Carfilzomib, Dexamethasone, and Daratumumab to Carfilzomib and Dexamethasone for the treatment of Patients With Relapsed or Refractory Multiple Myeloma	Protocol
Euctr, A. T. 2015	Study Comparing Daratumumab, Lenalidomide, and Dexamethasone With Lenalidomide and Dexamethasone in Participants with Previously Untreated Multiple Myeloma	Protocol

Supplementary Table 1: Reasons of exclusion for individual studies in the full text assessment (continued)

Study		Reason for exclusion
Ishida et al. 2018	Therapeutic antibodies for multiple myeloma	Review
Bhatnagar et al. 2017	FDA Approval Summary: Daratumumab for Treatment of Multiple Myeloma After One Prior Therapy	Review
Al Hadidi et al. 2020	Safety Analysis of Five Randomized Controlled Studies of Daratumumab in Patients With Multiple Myeloma	Review
Cao et al. 2021	Daratumumab provides a survival benefit in relapsed and refractory Multiple Myeloma, independent of baseline clinical characteristics: A meta-analysis	Review
-	Late Breaking: 61st ASH Annual Meeting Abstracts	Review
-	17th International Myeloma Workshop	Review
-	2018 Annual Meeting of the American Society of Hematology, ASH 2018	Review
Weisel et al. 2020	Health-related quality of life of carfilzomib- and daratumumab-based therapies in patients with relapsed/refractory multiple myeloma, based on German benefit assessment data	Outcome of interest is not reported
Borelli et al. 2020	Differences in safety profiles of newly approved medications for multiple myeloma in real-world settings versus randomized controlled trials	Outcome of interest is not reported

Supplementary Figure 1: Forest plot and Trial Sequential Analysis representing that addition of daratumumab to backbone therapies increases the chance for achieving minimal residual disease negativity in newly diagnosed multiple myeloma

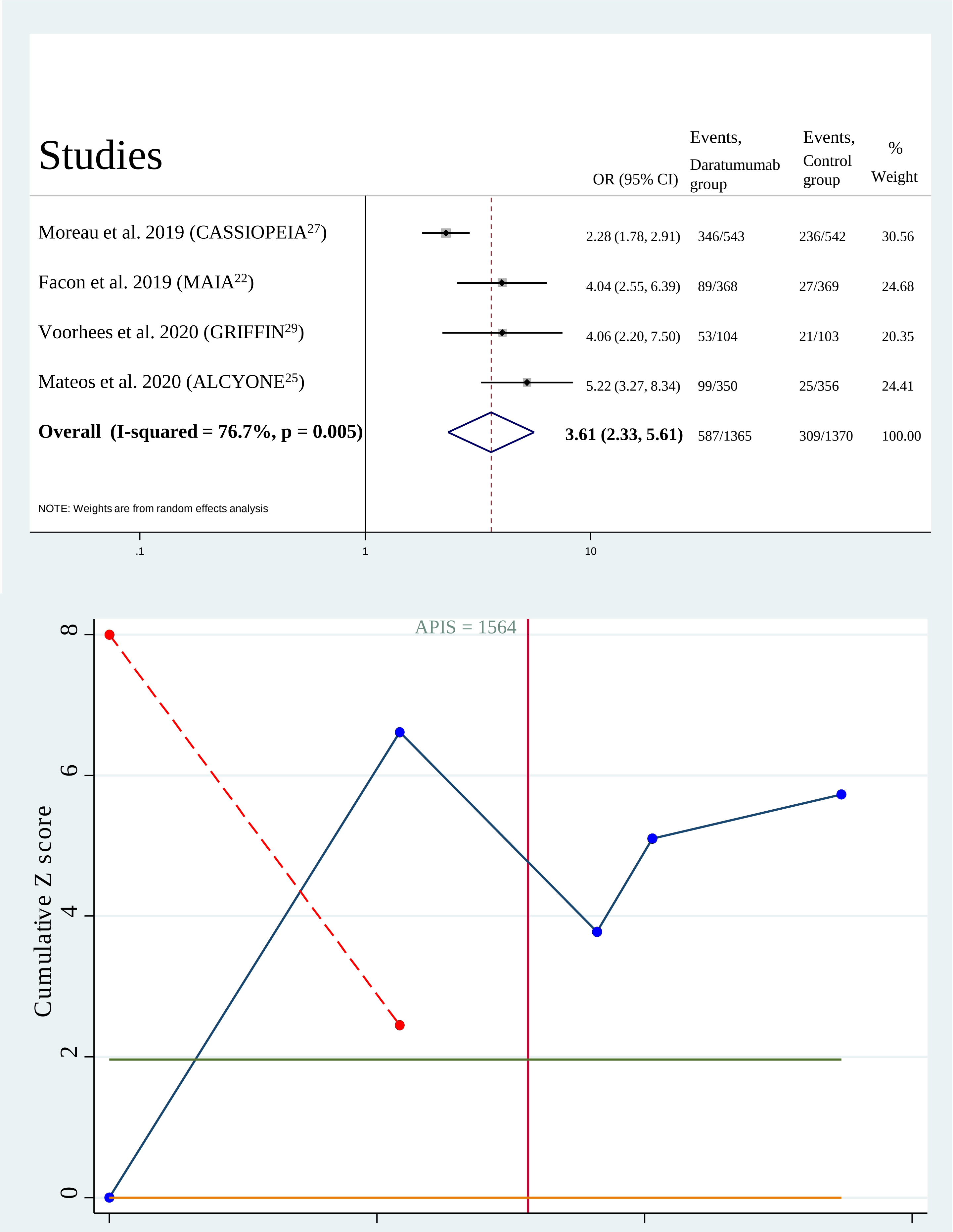

Supplementary Figure 2: Risk of bias assessment at study level and at domain level regarding minimal residual disease negativity in newly diagnosed multiple myeloma

DRVd, daratumumab, bortezomib, lenalidomide and dexamethasone; RVd, bortezomib, lenalidomide and dexamethasone; DVMP, daratumumab, bortezomib, melphalan, and prednisone; VMP, bortezomib, melphalan, and prednisone; DVTd, daratumumab, bortezomib, thalidomide, and dexamethasone; VTd, bortezomib, thalidomide, and dexamethasone; DRd, daratumumab, lenalidomide and dexamethasone; Rd, lenalidomide and dexamethasone

Study	Interventions	Randomization process	Deviations from intended interventions	Missing outcome data	Measurement of the outcome	Selection of the reported results	Overall assessment
Voorhees et al. 2020	DRVd vs RVd	?	+	?	+	+	?
Mateos et al. 2020	DVMP vs VMP	+	+	+	+	+	+
Moreau et al. 2019	DVTd vs VTd	+	+	+	+	?	+
Facon et al. 2019	DRd vs Rd	+	+	+	+	+	+

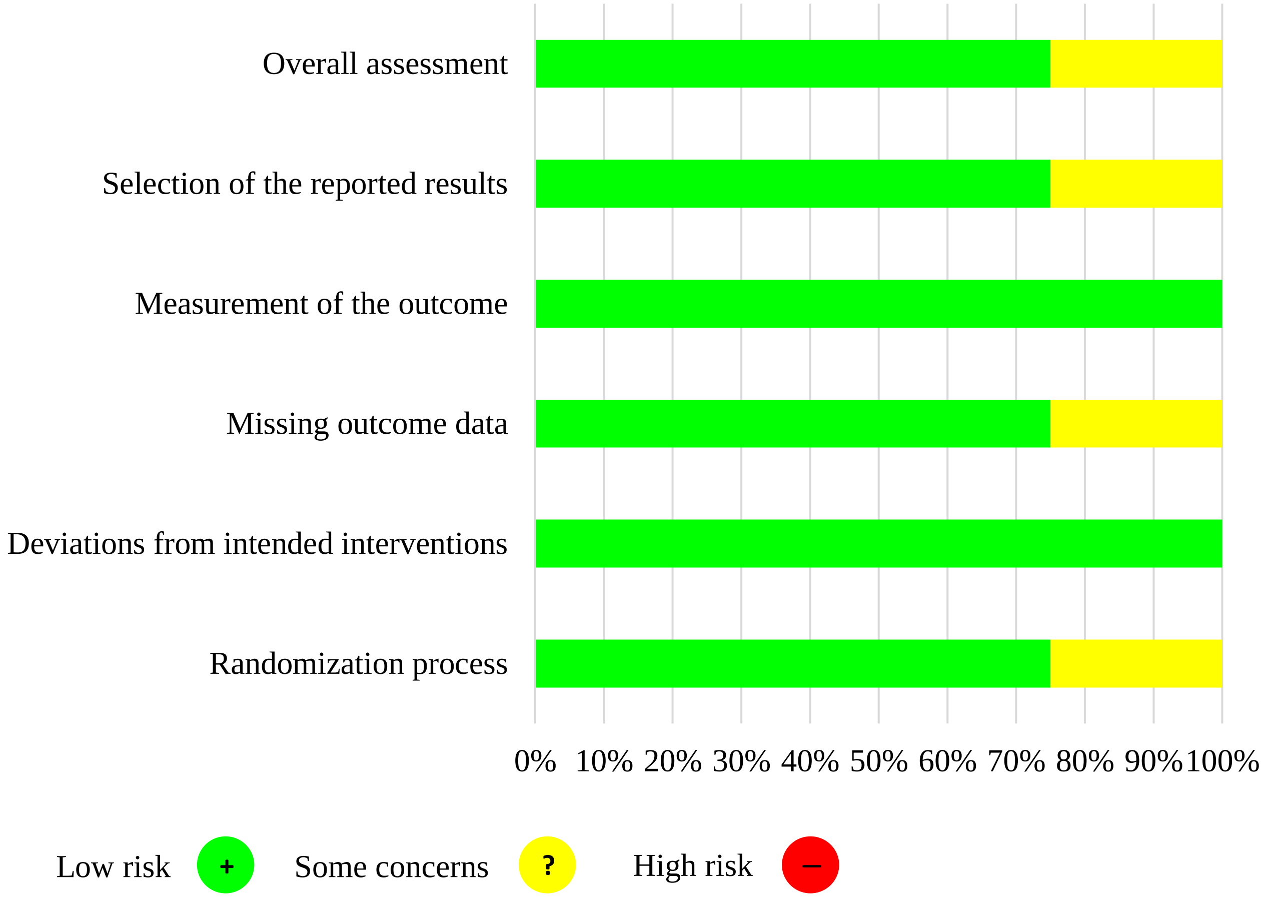

Supplementary Figure 3: Forest plot and Trial Sequential Analysis representing that addition of daratumumab to backbone therapies increases the chance for achieving stringent complete response in newly diagnosed multiple myeloma

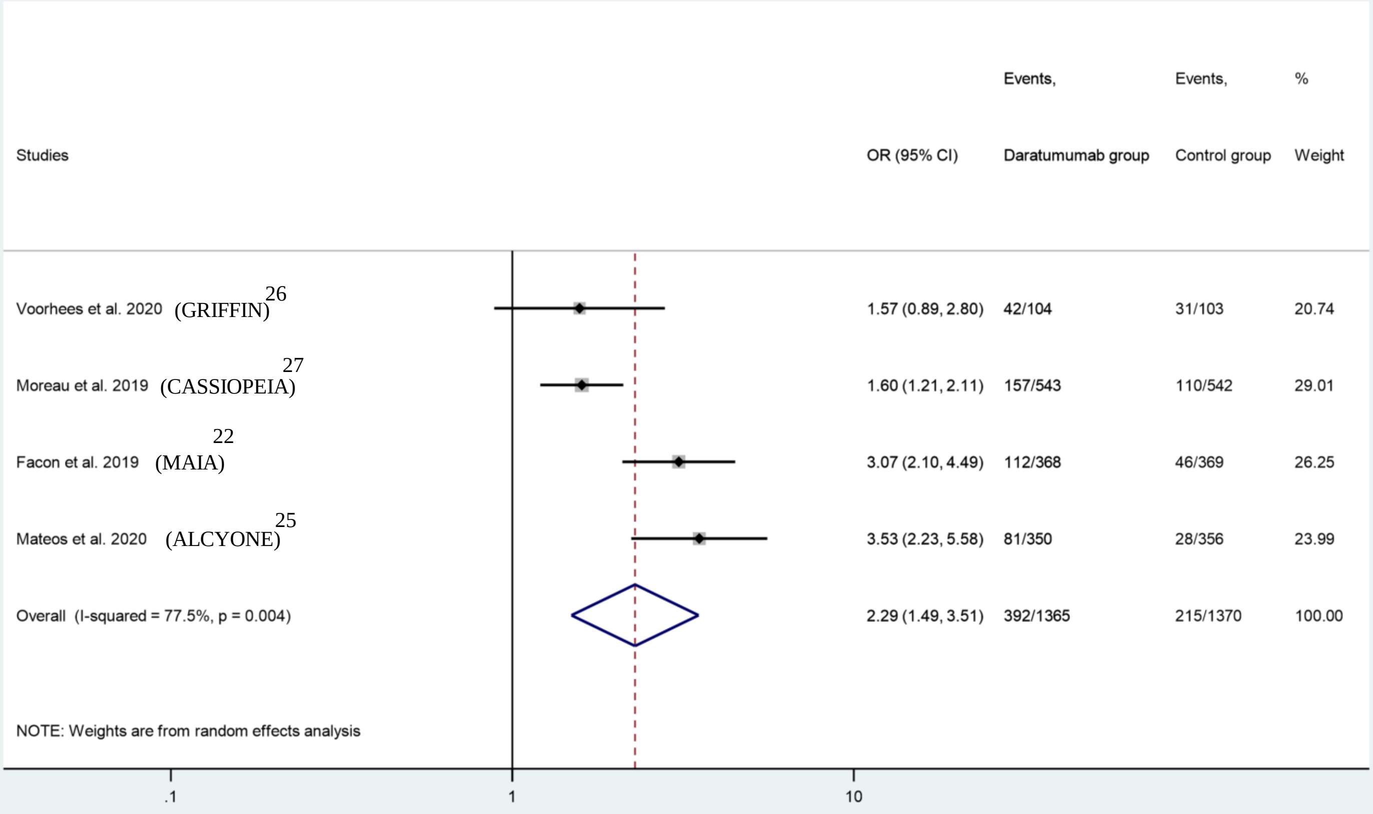

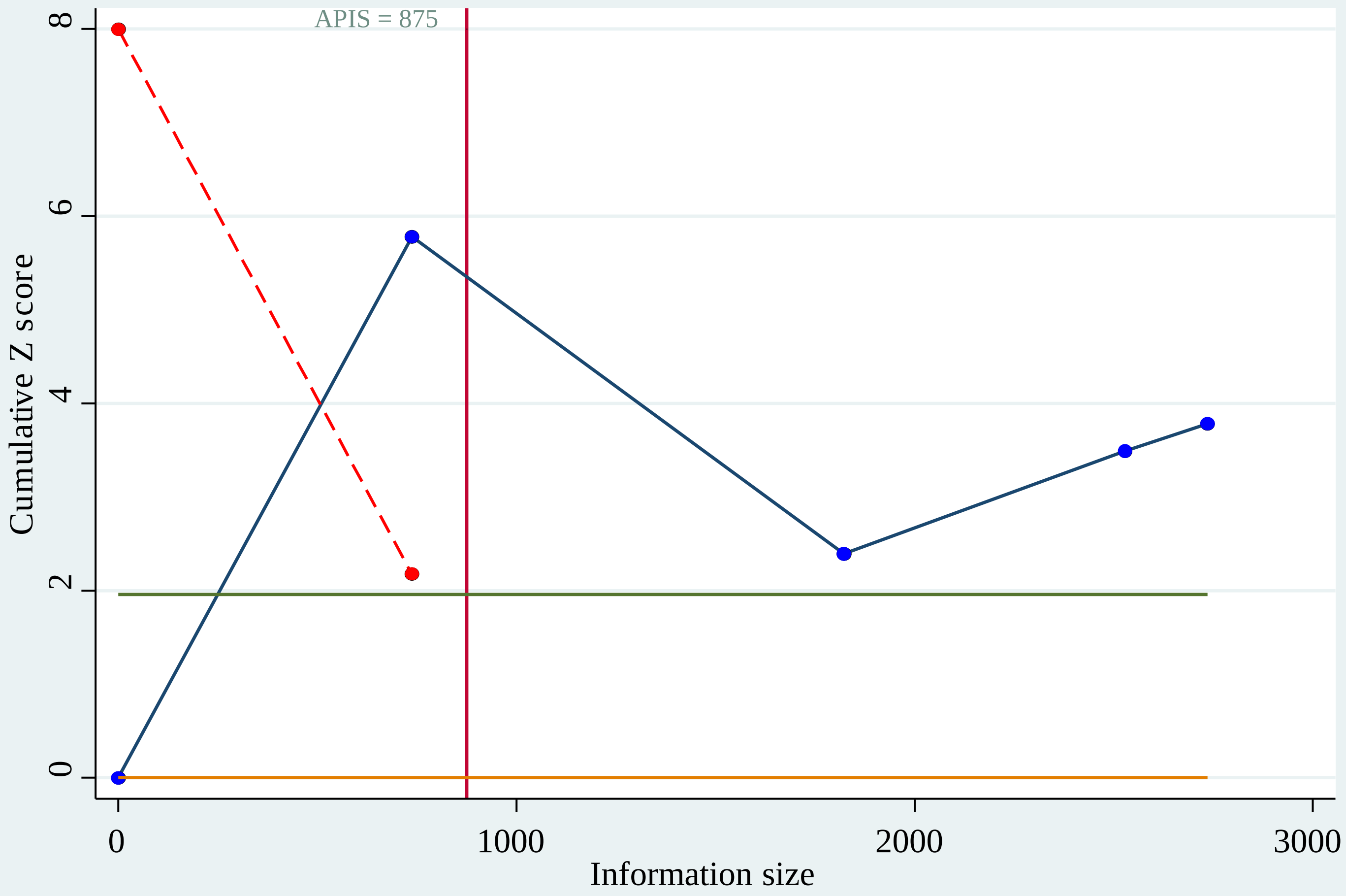

Supplementary Figure 4: Risk of bias assessment at study level and at domain level regarding stringent complete response in newly diagnosed multiple myeloma

DRVd, daratumumab, bortezomib, lenalidomide and dexamethasone; RVd, bortezomib, lenalidomide and dexamethasone; DVMP, daratumumab, bortezomib, melphalan, and prednisone; VMP, bortezomib, melphalan, and prednisone; DVTd, daratumumab, bortezomib, thalidomide, and dexamethasone; VTd, bortezomib, thalidomide, and dexamethasone; DRd, daratumumab, lenalidomide and dexamethasone; Rd, lenalidomide and dexamethasone

Study	Interventions	Randomization process	Deviations from intended interventions	Missing outcome data	Measurement of the outcome	Selection of the reported results	Overall assessment
Voorhees et al. 2020	DRVd vs RVd	?	+	+	+	+	?
Mateos et al. 2020	DVMP vs VMP	+	+	+	+	+	+
Moreau et al. 2019	DVTd vs VTd	+	+	+	+	?	+
Facon et al. 2019	DRd vs Rd	+	+	+	+	+	+

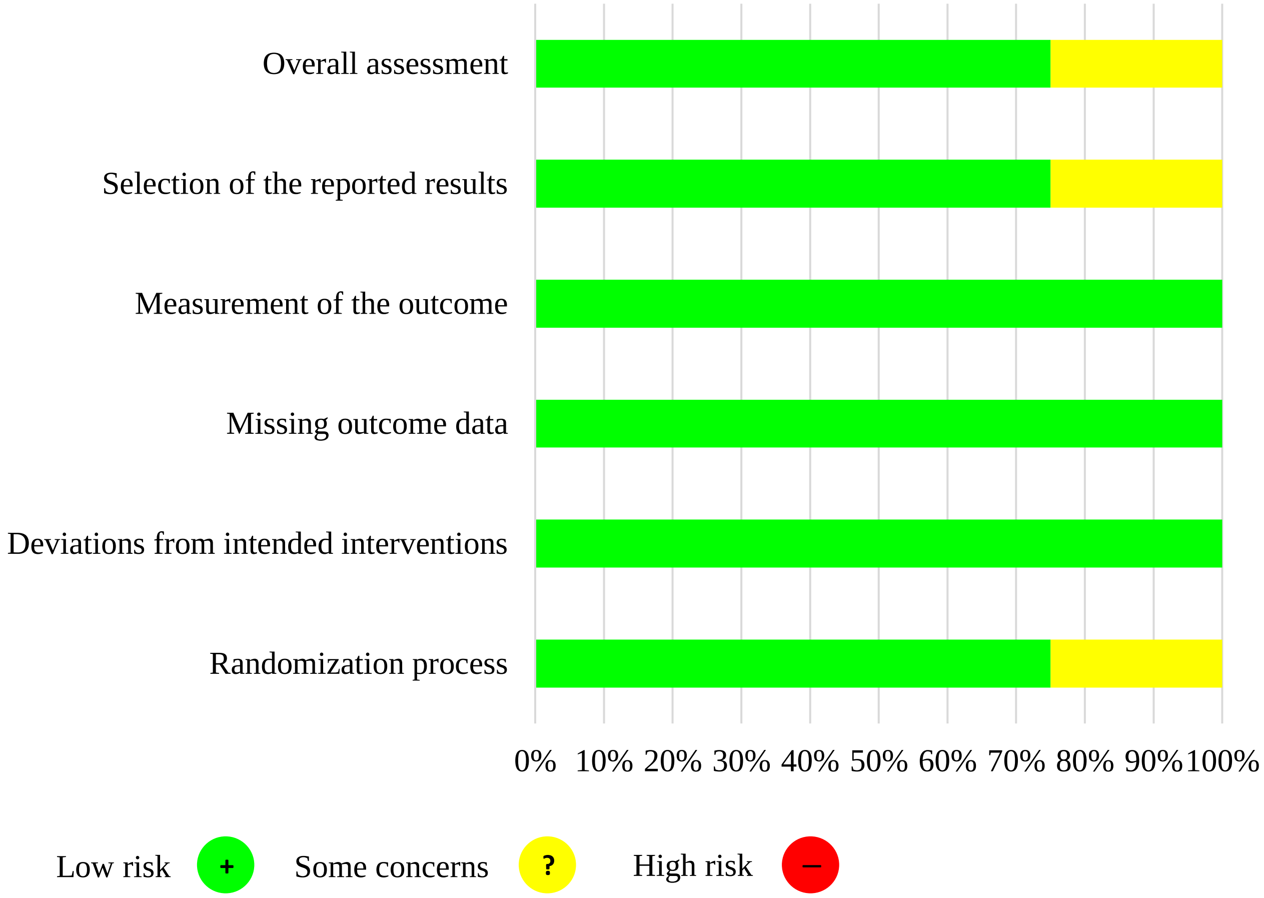

Supplementary Figure 5: Forest plot and Trial Sequential Analysis representing that addition of daratumumab to backbone therapies increases the chance for achieving complete response or better in newly diagnosed multiple myeloma

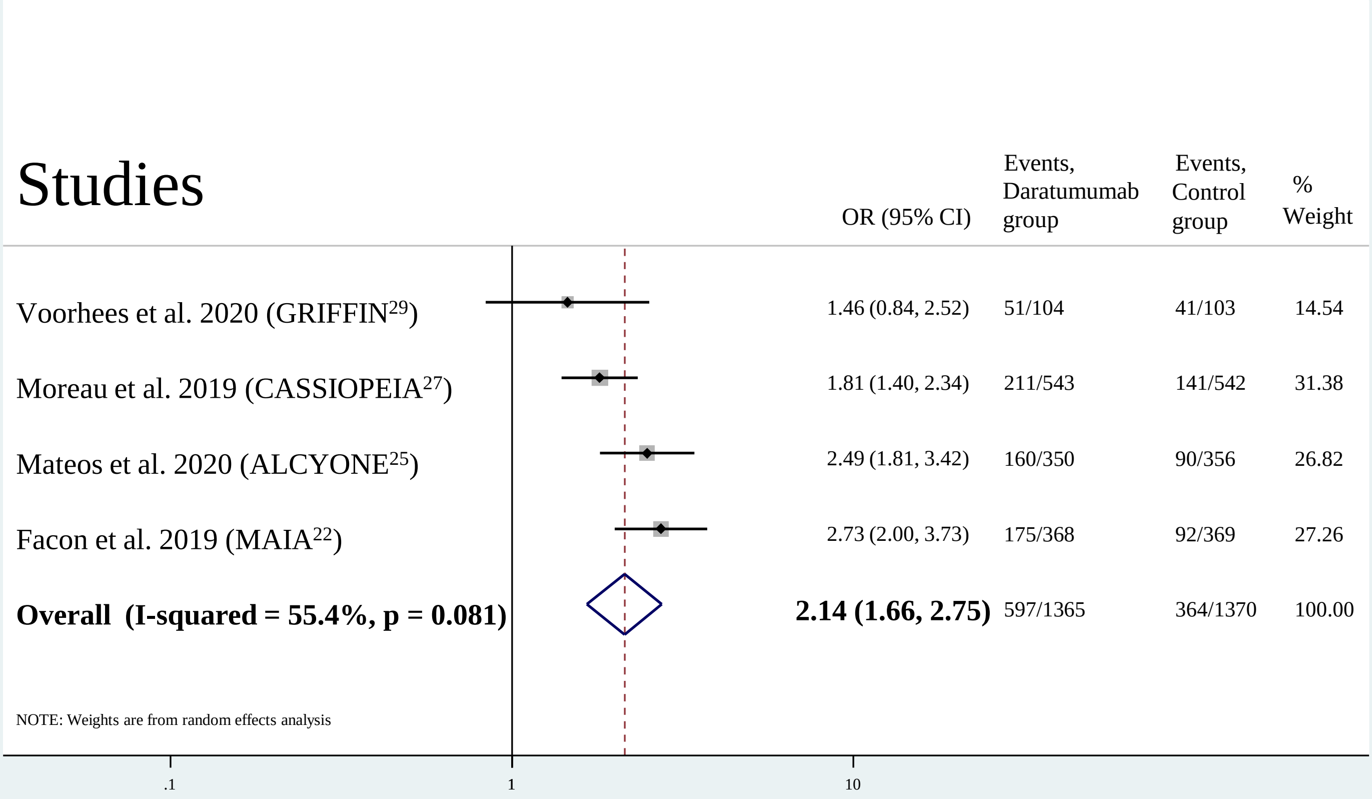

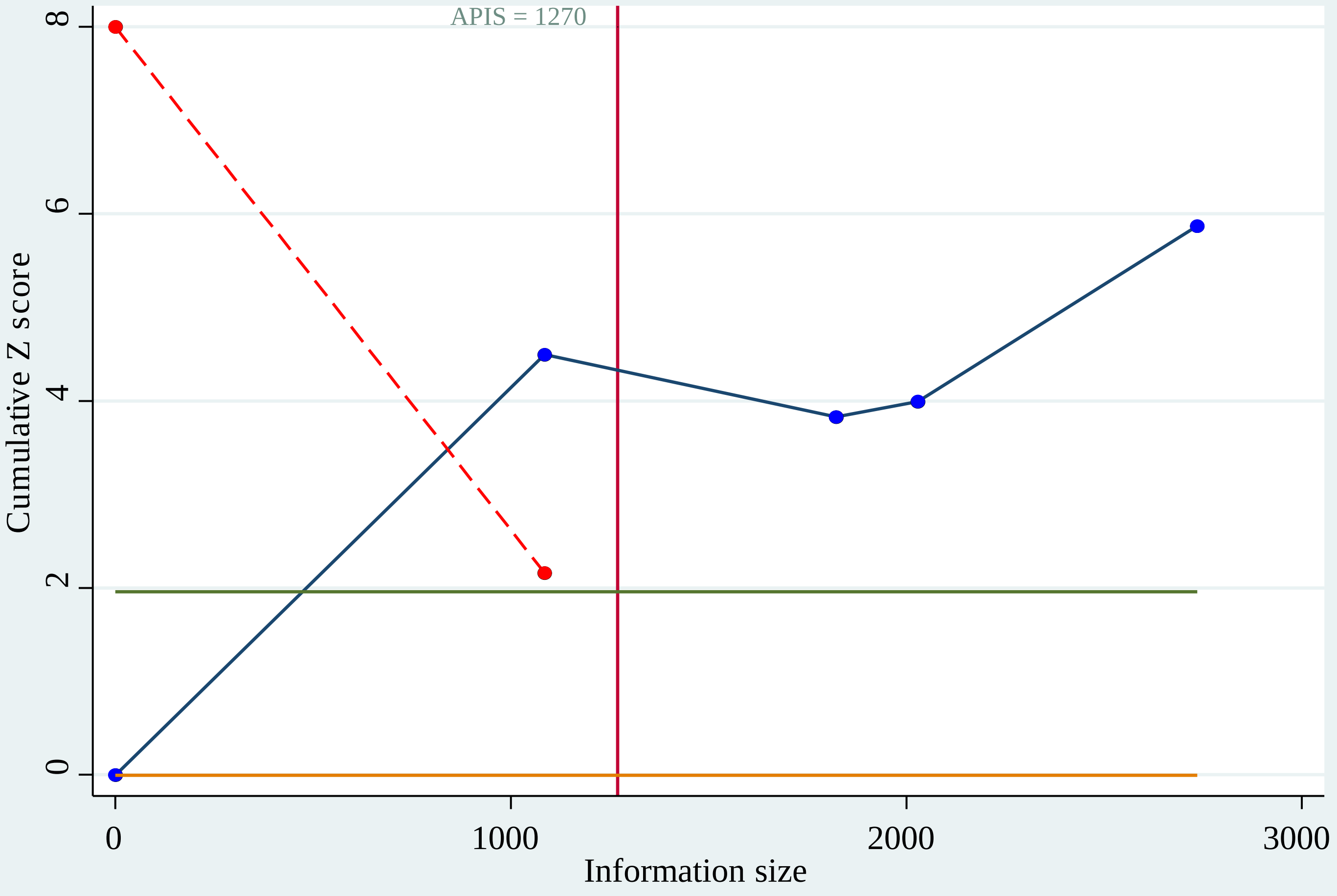

Supplementary Figure 6: Risk of bias assessment at study level and at domain level regarding complete response or better in newly diagnosed multiple myeloma

DRVd, daratumumab, bortezomib, lenalidomide and dexamethasone; RVd, bortezomib, lenalidomide and dexamethasone; DVMP, daratumumab, bortezomib, melphalan, and prednisone; VMP, bortezomib, melphalan, and prednisone; DVTd, daratumumab, bortezomib, thalidomide, and dexamethasone; VTd, bortezomib, thalidomide, and dexamethasone; DRd, daratumumab, lenalidomide and dexamethasone; Rd, lenalidomide and dexamethasone

**Study**	**Interventions**	**Randomization process**	**Deviations from intended interventions**	**Missing outcome data**	**Measurement of the outcome**	**Selection of the reported results**	**Overall assessment**
Voorhees et al. 2020	DRVd vs RVd	?	+	+	+	+	?
Mateos et al. 2020	DVMP vs VMP	+	+	+	+	+	+
Moreau et al. 2019	DVTd vs VTd	+	+	+	+	?	+
Facon et al. 2019	DRd vs Rd	+	+	+	+	+	+

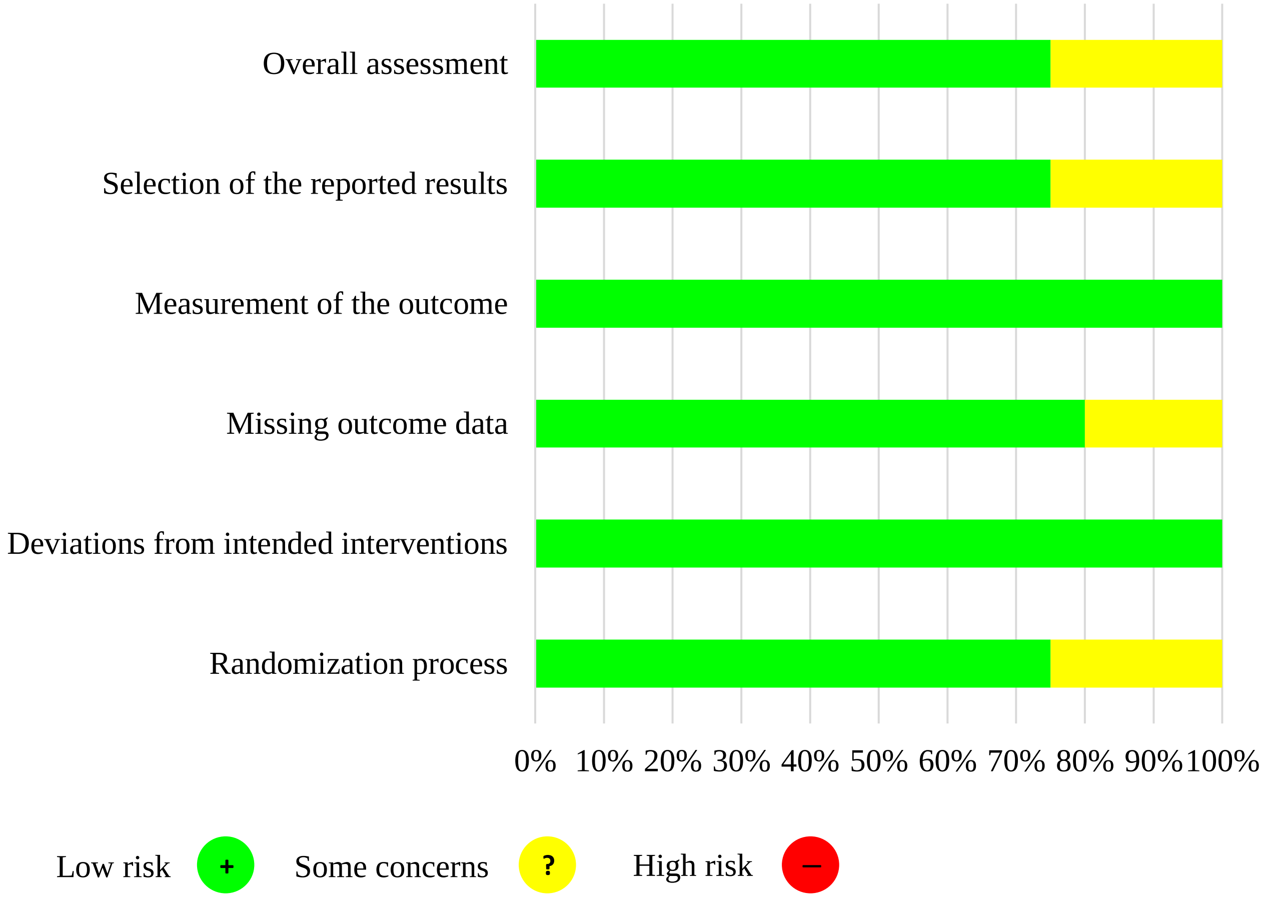

Supplementary Figure 7: Forest plot and Trial Sequential Analysis representing that addition of daratumumab to backbone therapies decreases the chance for death or disease progression in newly diagnosed multiple myeloma

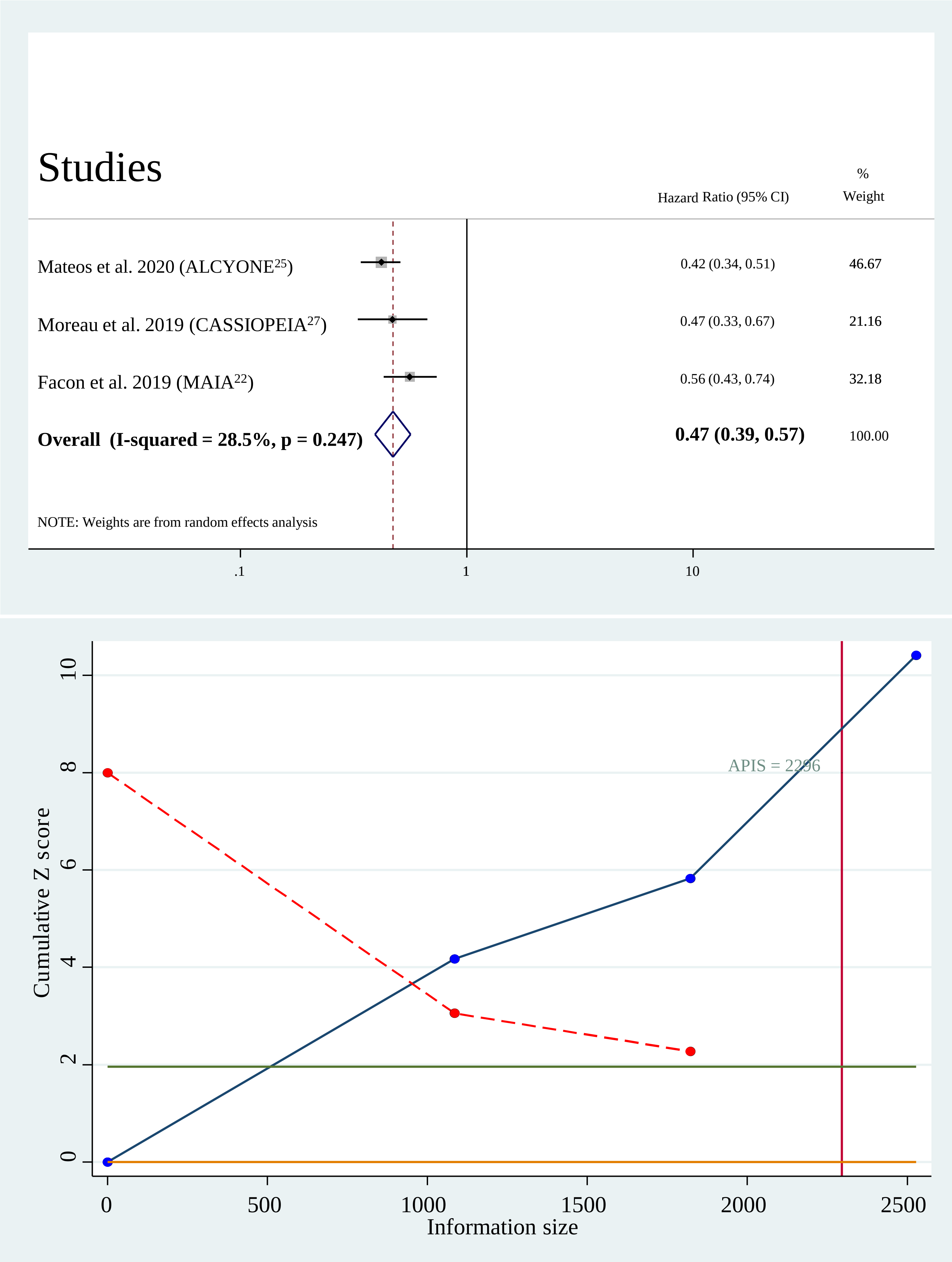

Supplementary Figure 8: Risk of bias assessment at study level and at domain level regarding death or disease progression in newly diagnosed multiple myeloma

DVMP, daratumumab, bortezomib, melphalan, and prednisone; VMP, bortezomib, melphalan, and prednisone; DVTd, daratumumab, bortezomib, thalidomide, and dexamethasone; VTd, bortezomib, thalidomide, and dexamethasone; DRd, daratumumab, lenalidomide and dexamethasone; Rd, lenalidomide and dexamethasone

Study	Interventions	Randomization process	Deviations from intended interventions	Missing outcome data	Measurement of the outcome	Selection of the reported results	Overall assessment
Mateos et al. 2020	DVMP vs VMP	+	+	+	+	+	+
Moreau et al. 2019	DVTd vs VTd	+	+	+	+	?	+
Facon et al. 2019	DRd vs Rd	+	+	+	+	+	+

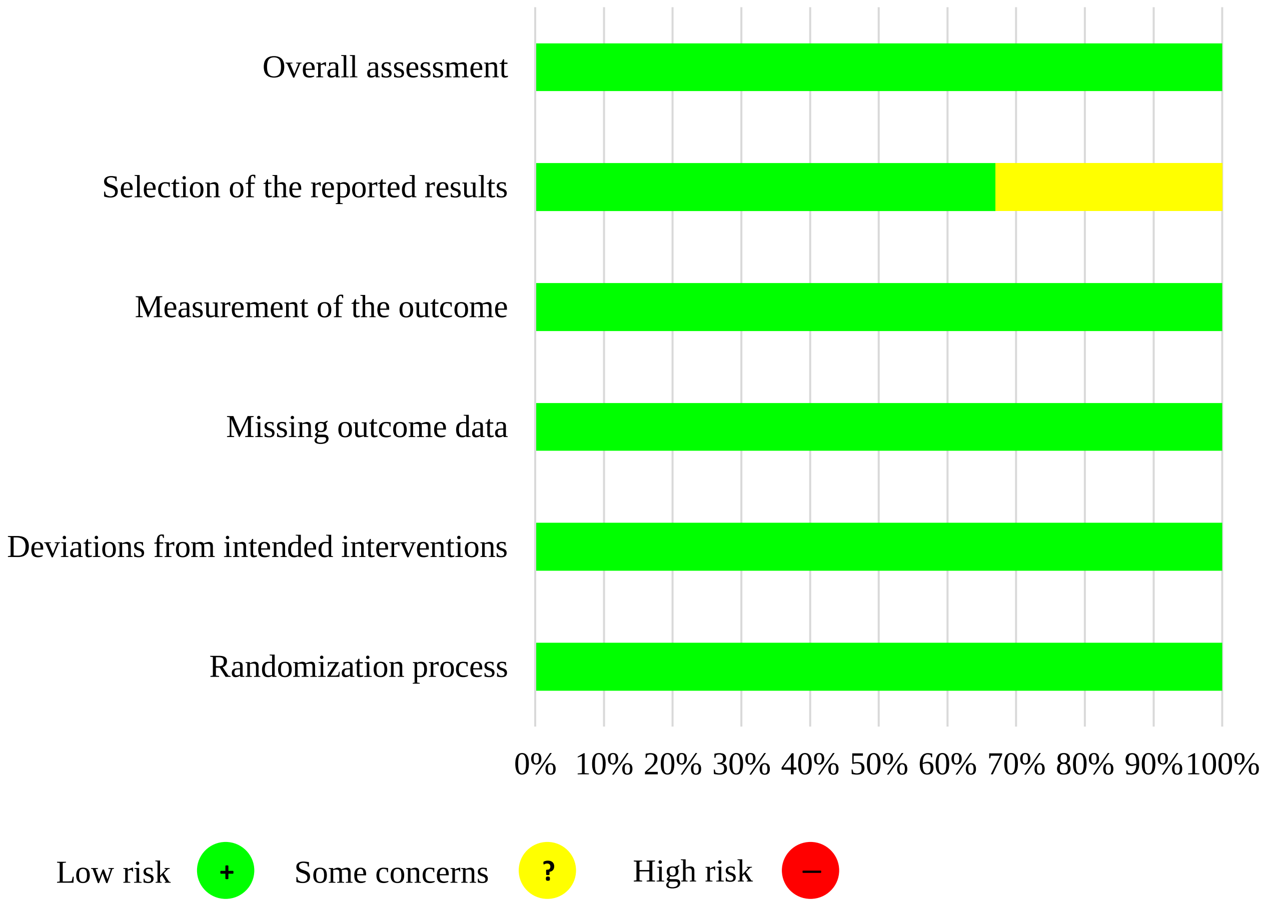

Supplementary Figure 9: Forest plot and Trial Sequential Analysis representing that addition of daratumumab to backbone therapies decreases the chance for death or disease progression in high cytogenetic risk newly diagnosed multiple myeloma

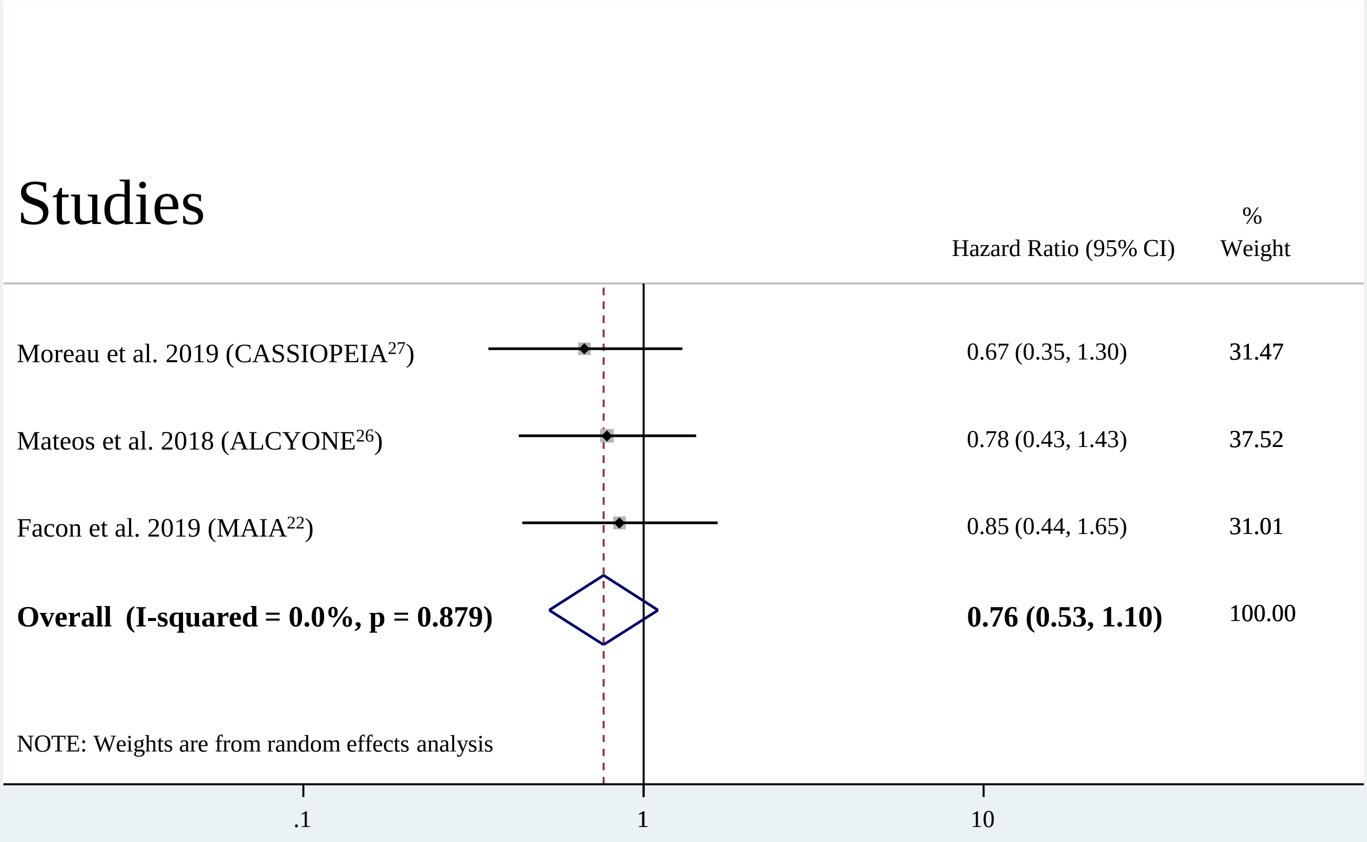

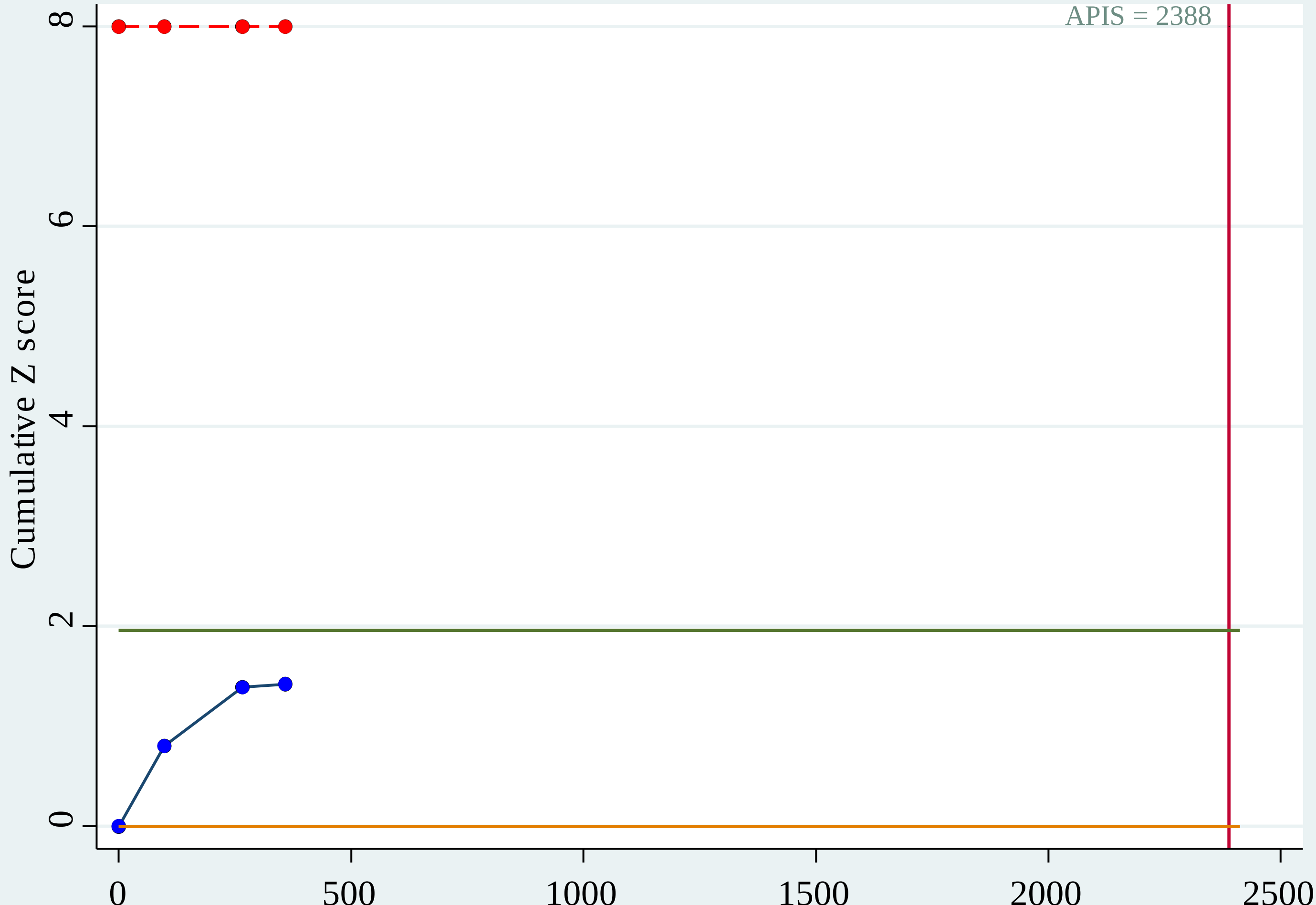

Supplementary Figure 10: Risk of bias assessment at study level and at domain level regarding death or disease progression in high cytogenetic risk newly diagnosed multiple myeloma
DVMP, daratumumab, bortezomib, melphalan, and prednisone; VMP, bortezomib, melphalan, and prednisone; DVTd, daratumumab, bortezomib, thalidomide, and dexamethasone; VTd, bortezomib, thalidomide, and dexamethasone; DRd, daratumumab, lenalidomide and dexamethasone; Rd, lenalidomide and dexamethasone

Study	Interventions	Randomization process	Deviations from intended interventions	Missing outcome data	Measurement of the outcome	Selection of the reported results	Overall assessment
Mateos et al. 2020	DVMP vs VMP	+	+	+	+	+	+
Moreau et al. 2019	DVTd vs VTd	+	+	+	+	?	+
Facon et al. 2019	DRd vs Rd	+	+	+	+	+	+

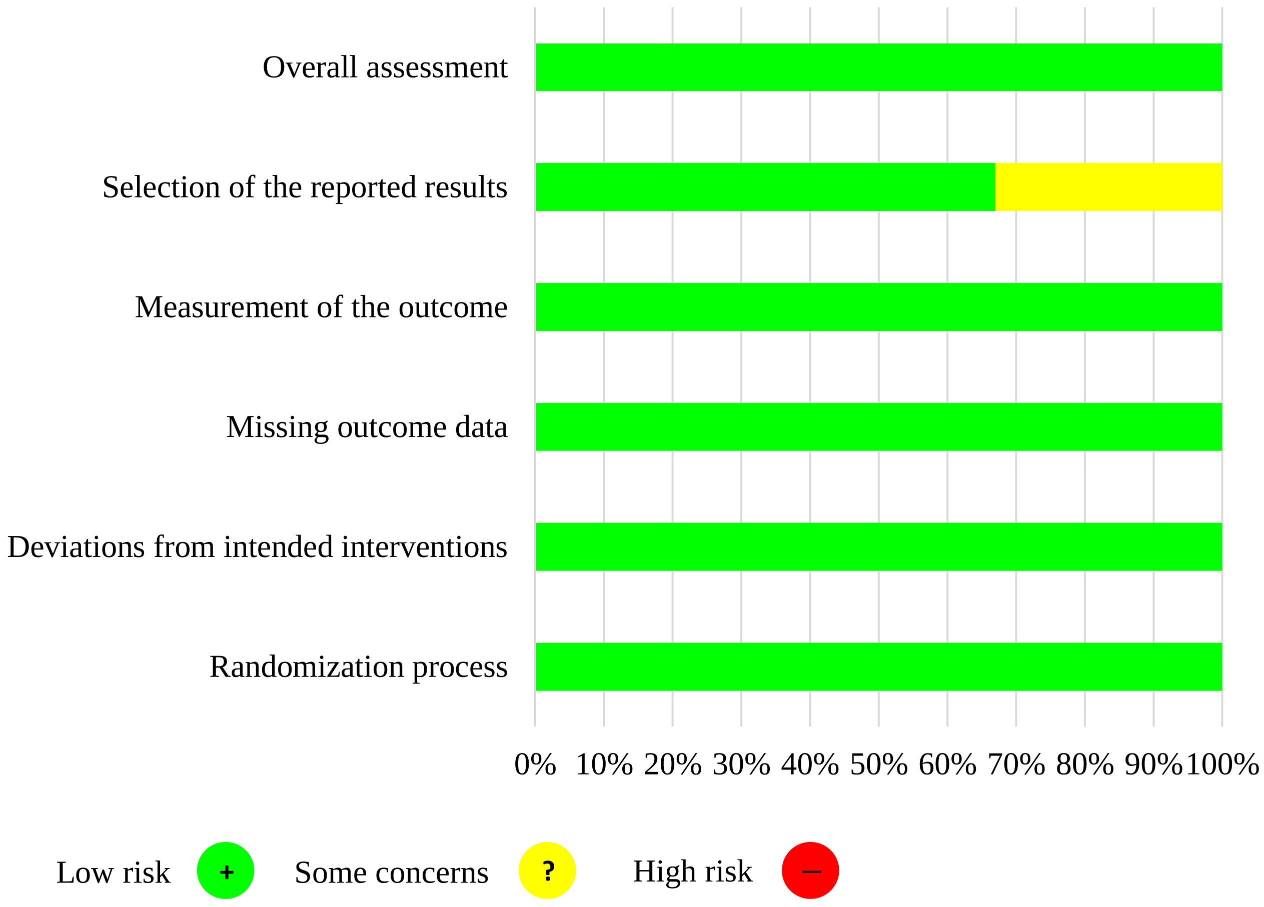

Supplementary Figure 11: Forest plot and Trial Sequential Analysis representing that addition of daratumumab to backbone therapies decreases the chance for death or disease progression in standard cytogenetic risk newly diagnosed multiple myeloma

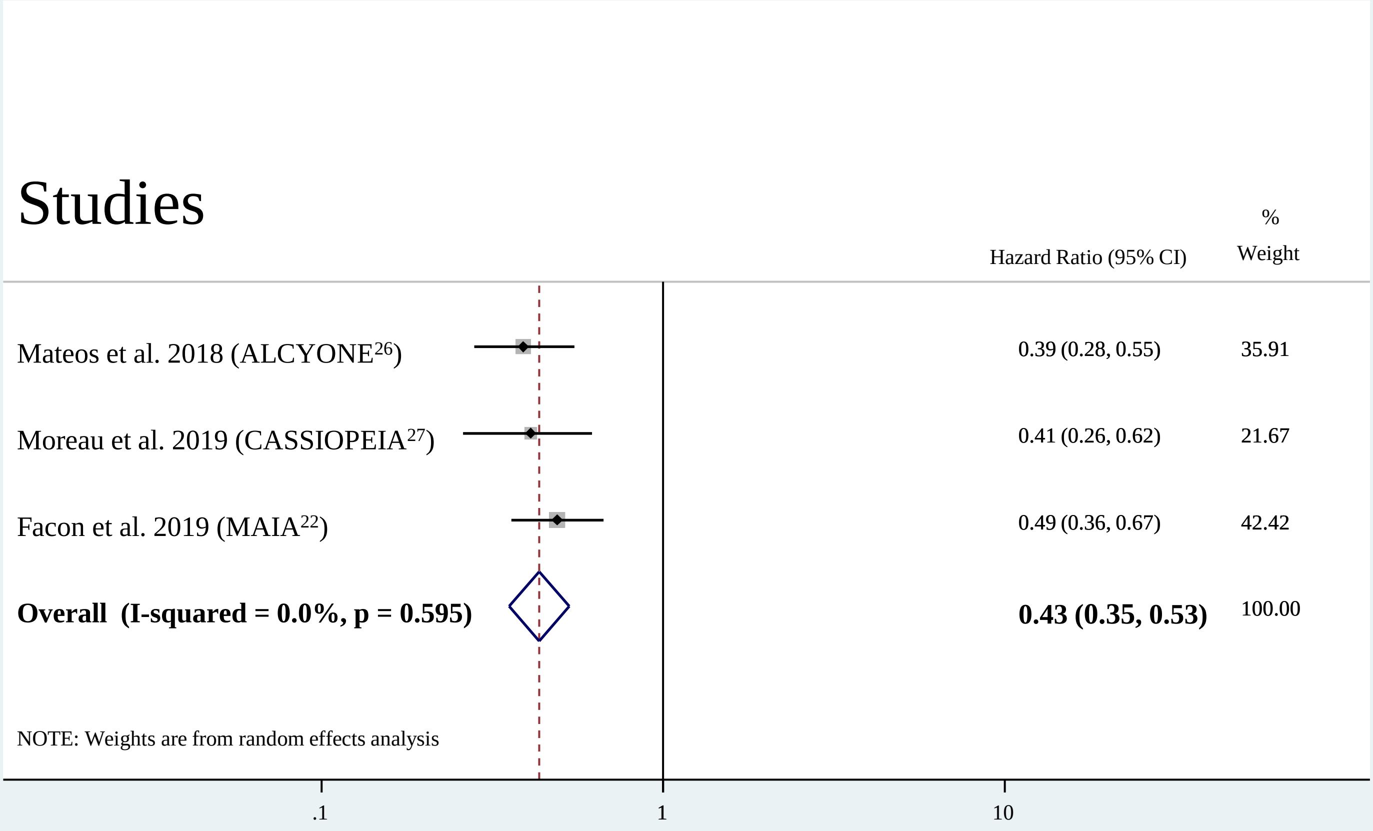

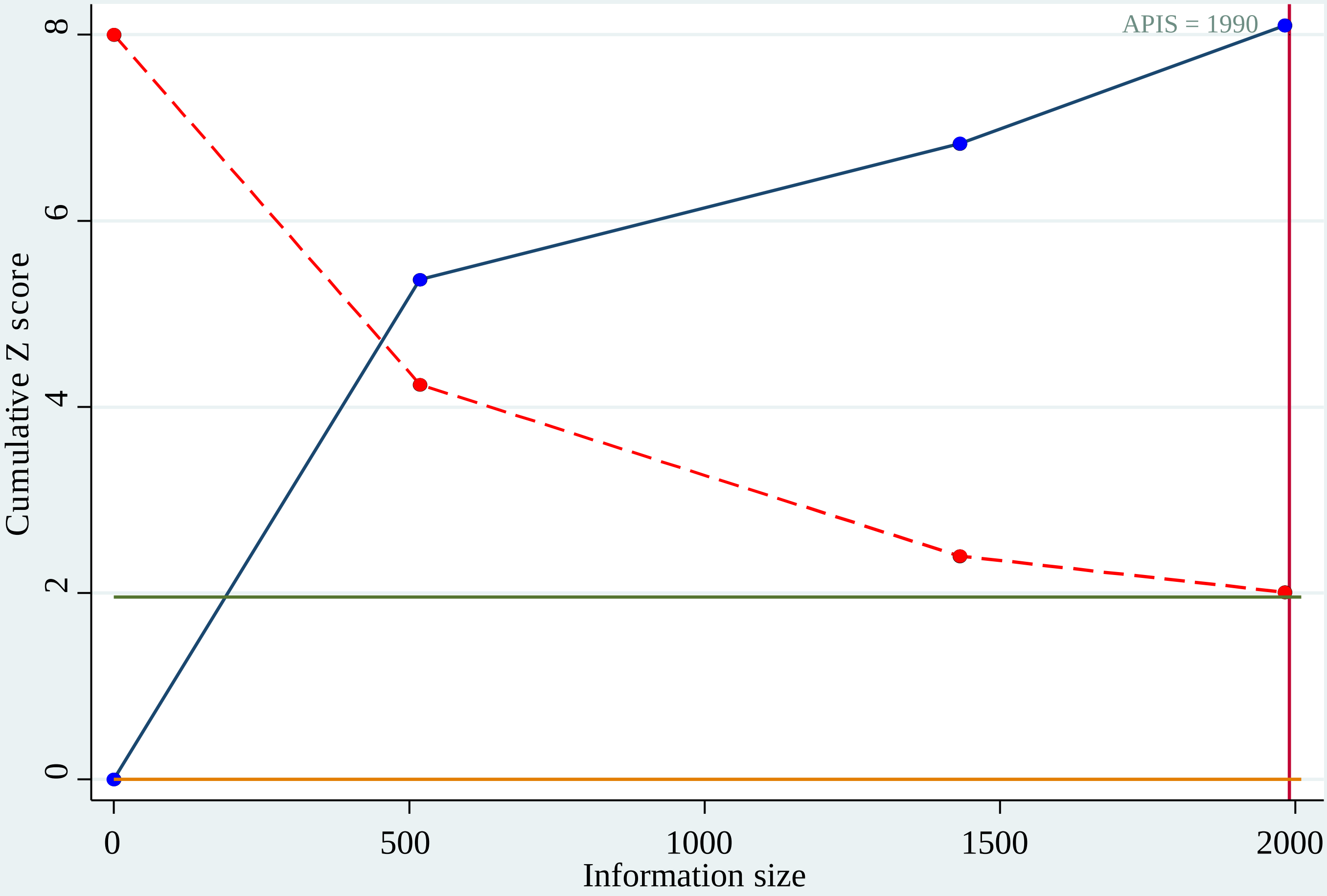

Supplementary Figure 12: Risk of bias assessment at study level and at domain level regarding death or disease progression in standard cytogenetic risk newly diagnosed multiple myeloma
DVMP, daratumumab, bortezomib, melphalan, and prednisone; VMP, bortezomib, melphalan, and prednisone; DVTd, daratumumab, bortezomib, thalidomide, and dexamethasone; VTd, bortezomib, thalidomide, and dexamethasone; DRd, daratumumab, lenalidomide and dexamethasone; Rd, lenalidomide and dexamethasone

Study	Interventions	Randomization process	Deviations from intended interventions	Missing outcome data	Measurement of the outcome	Selection of the reported results	Overall assessment
Mateos et al. 2020	DVMP vs VMP	+	+	+	+	+	+
Moreau et al. 2019	DVTd vs VTd	+	+	+	+	?	+
Facon et al. 2019	DRd vs Rd	+	+	+	+	+	+

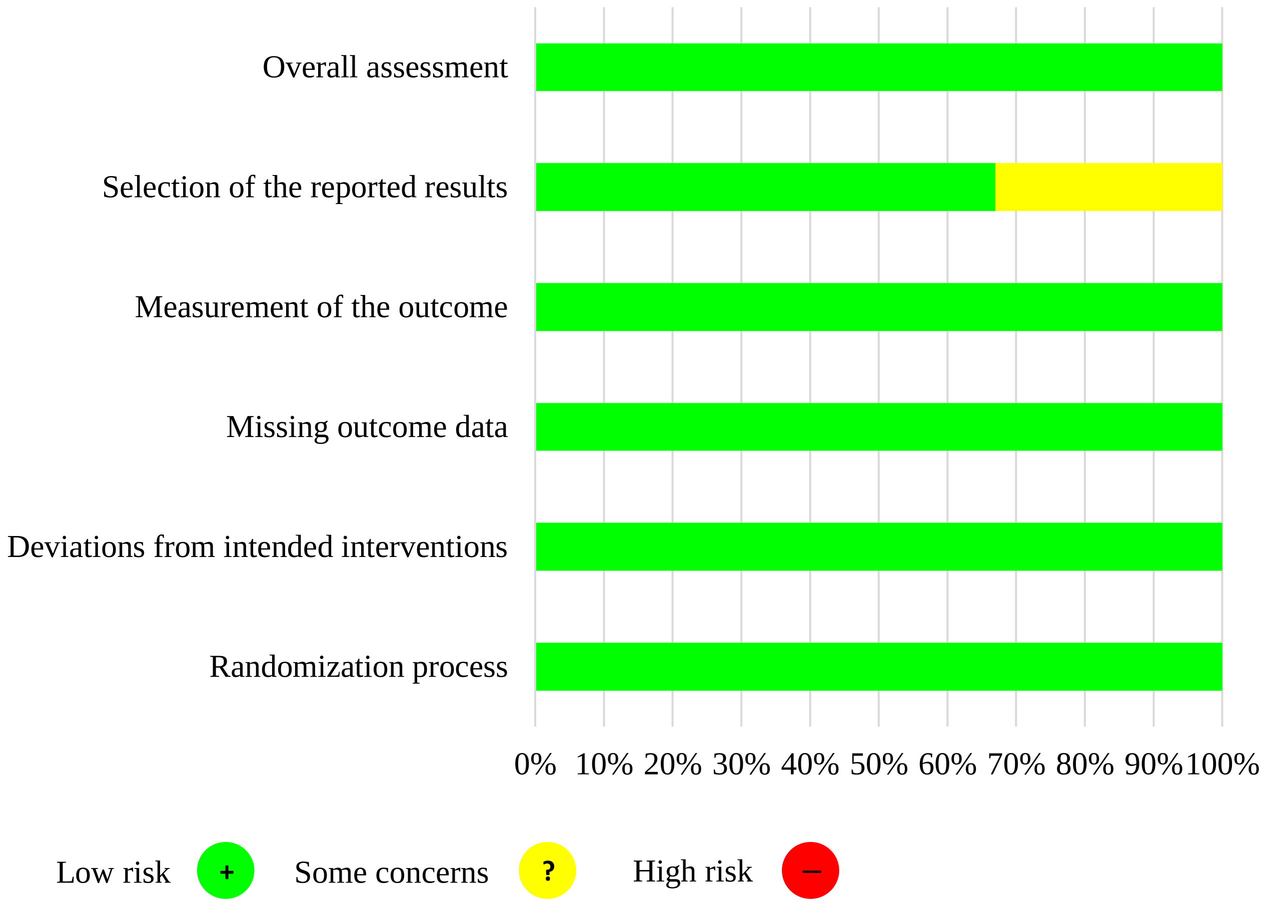

Supplementary Figure 13: Forest plot and Trial Sequential Analysis representing that addition of daratumumab to backbone therapies does not increase the chance for all grade thrombocytopenia in newly diagnosed multiple myeloma

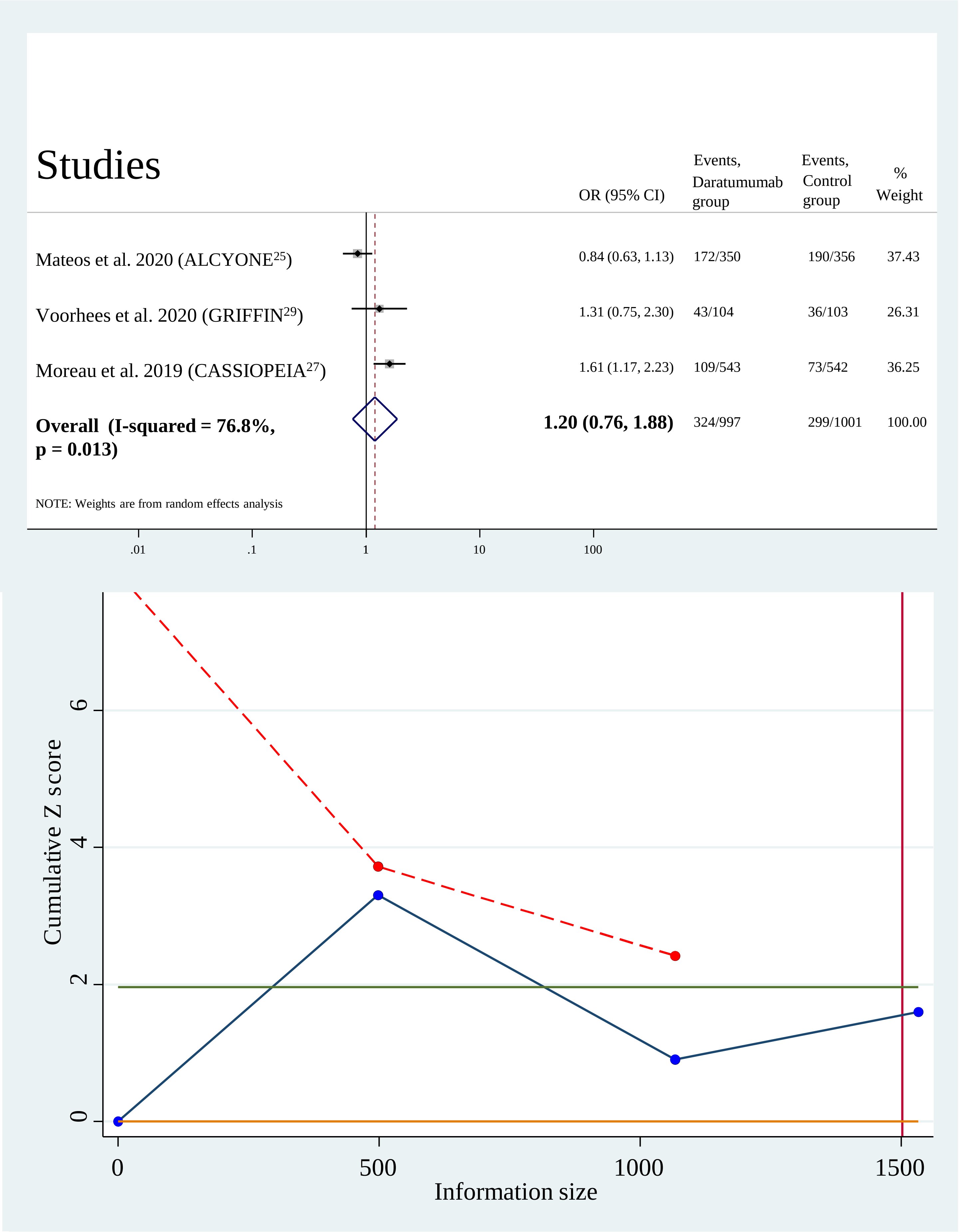

Supplementary Figure 14: Risk of bias assessment at study level and at domain level regarding all grade thrombocytopenia in newly diagnosed multiple myeloma
DVMP, daratumumab, bortezomib, melphalan, and prednisone; VMP, bortezomib, melphalan, and prednisone; DVTd, daratumumab, bortezomib, thalidomide, and dexamethasone; VTd, bortezomib, thalidomide, and dexamethasone; DRd, daratumumab, lenalidomide and dexamethasone; Rd, lenalidomide and dexamethasone

Study	Interventions	Randomization process	Deviations from intended interventions	Missing outcome data	Measurement of the outcome	Selection of the reported results	Overall assessment
Voorhees et al. 2020	DRVd vs RVd	?	+	+	+	+	?
Moreau et al. 2019	DVTd vs VTd	+	+	+	+	?	+
Mateos et al. 2020	DVMP vs VMP	+	+	+	+	+	+

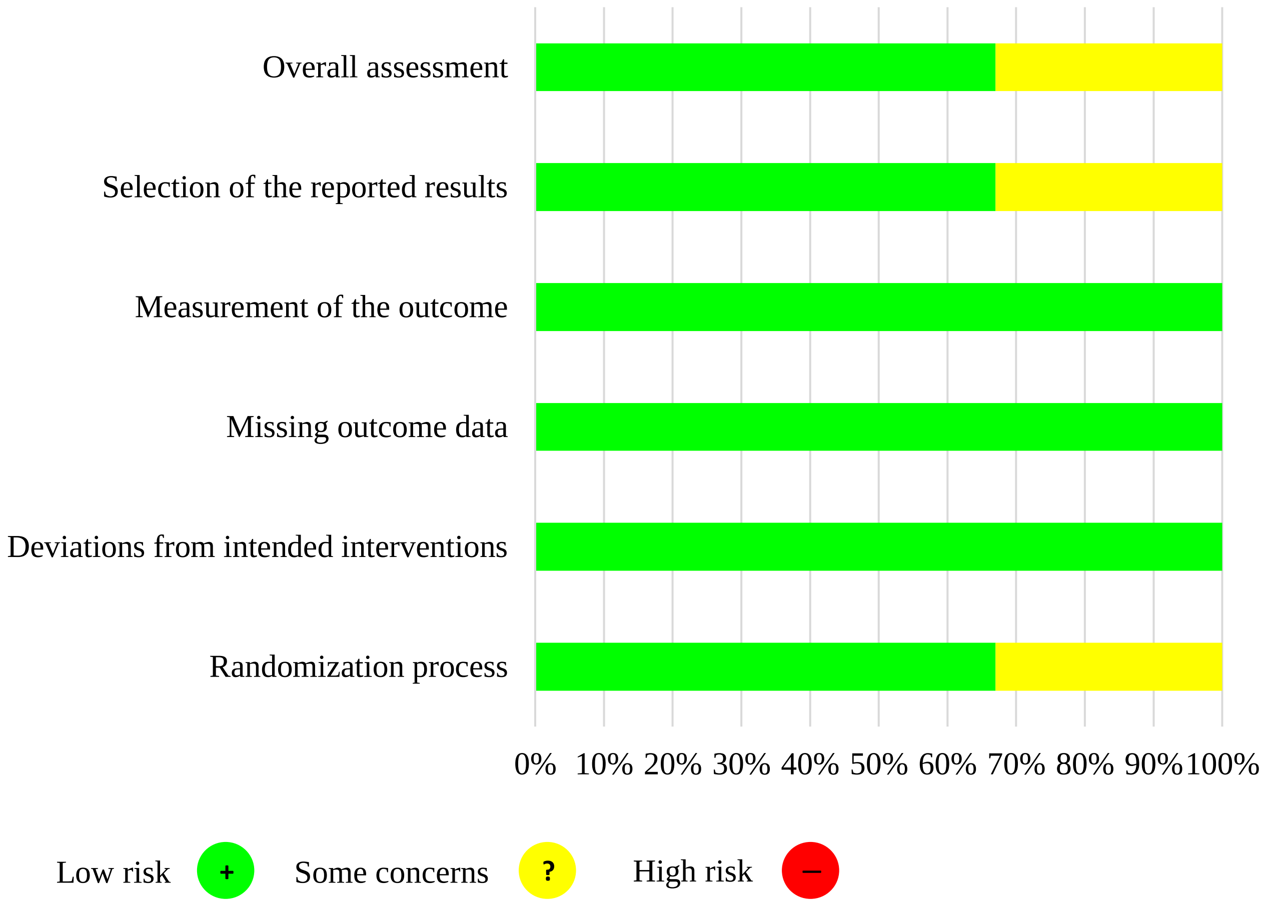

Supplementary Figure 15: Forest plot and Trial Sequential Analysis representing that addition of daratumumab to backbone therapies does not increase the chance for grade 3-4 thrombocytopenia in newly diagnosed multiple myeloma

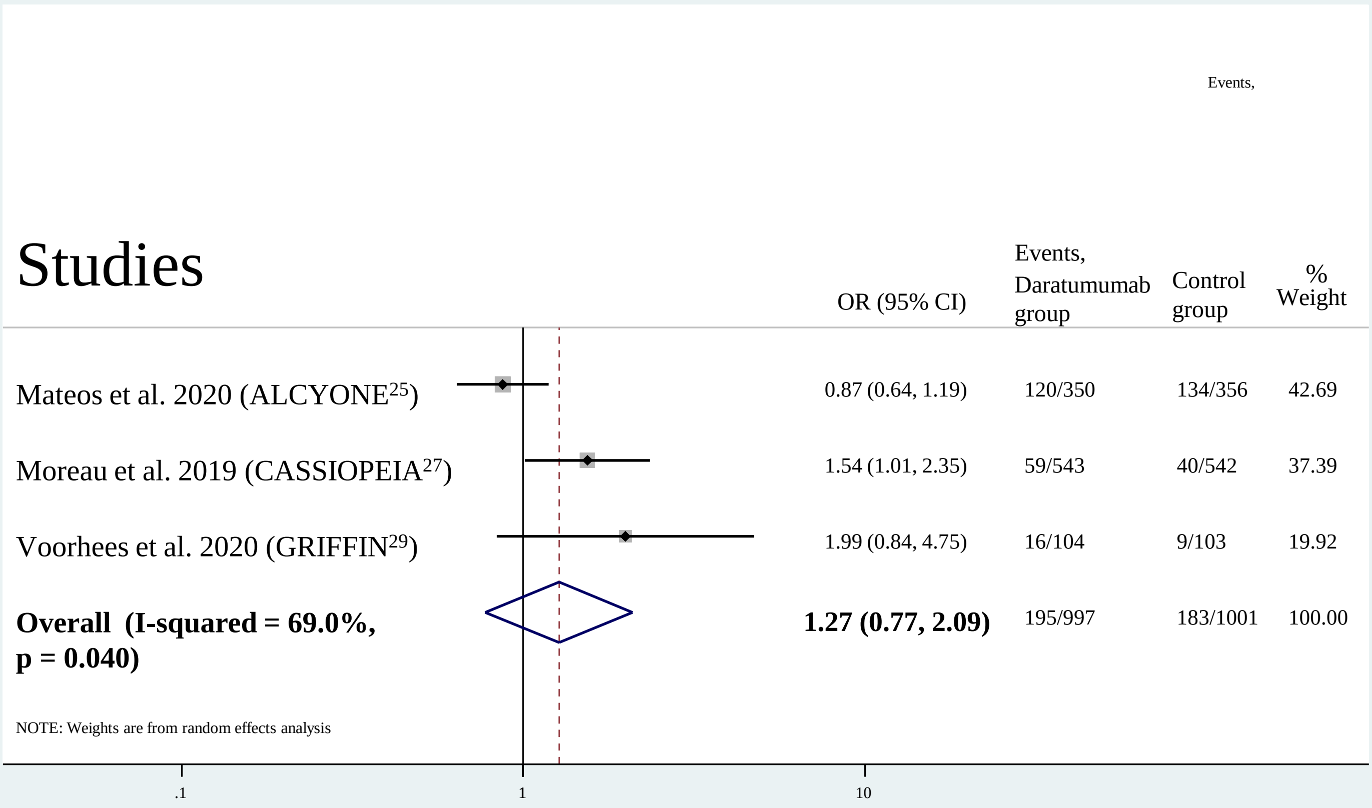

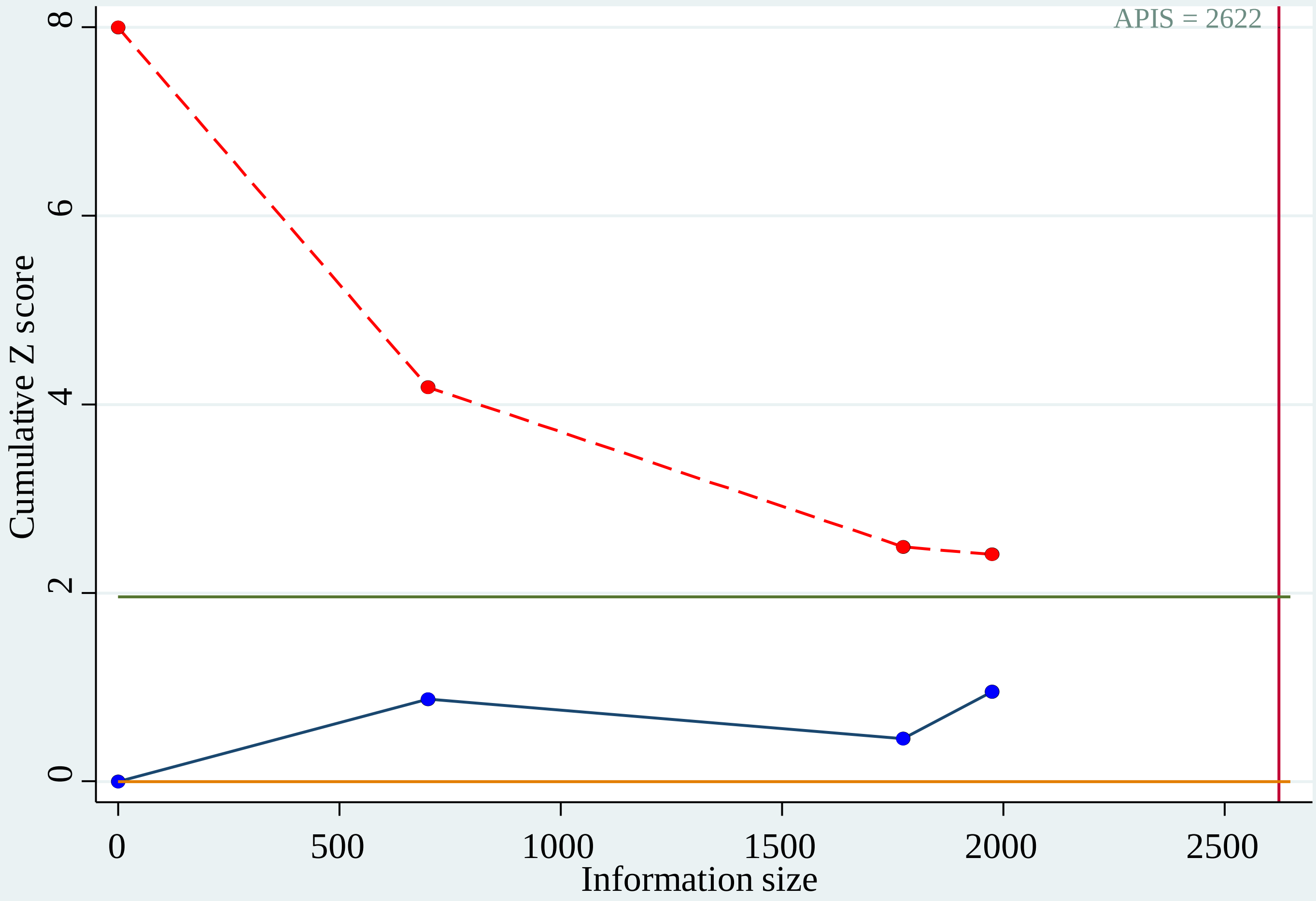

Supplementary Figure 16: Risk of bias assessment at study level and at domain level regarding grade 3-4 thrombocytopenia in newly diagnosed multiple myeloma
DVMP, daratumumab, bortezomib, melphalan, and prednisone; VMP, bortezomib, melphalan, and prednisone; DVTd, daratumumab, bortezomib, thalidomide, and dexamethasone; VTd, bortezomib, thalidomide, and dexamethasone; DRd, daratumumab, lenalidomide and dexamethasone; Rd, lenalidomide and dexamethasone

Study	Interventions	Randomization process	Deviations from intended interventions	Missing outcome data	Measurement of the outcome	Selection of the reported results	Overall assessment
Voorhees et al. 2020	DRVd vs RVd	?	+	+	+	+	?
Moreau et al. 2019	DVTd vs VTd	+	+	+	+	?	+
Mateos et al. 2020	DVMP vs VMP	+	+	+	+	+	+

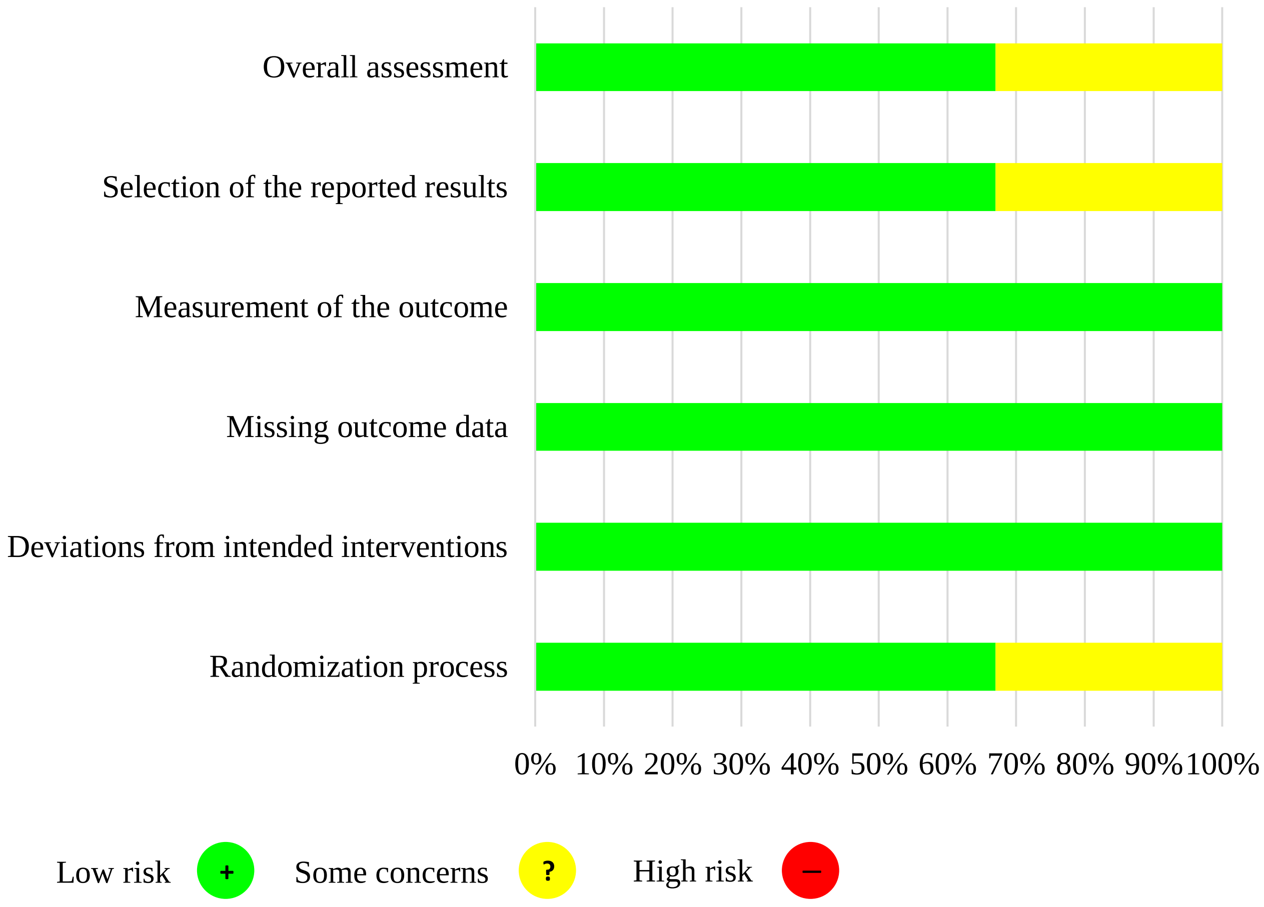

Supplementary Figure 17: Forest plot and Trial Sequential Analysis representing that addition of daratumumab to backbone therapies increases the chance for all grade lymphopenia in newly diagnosed multiple myeloma

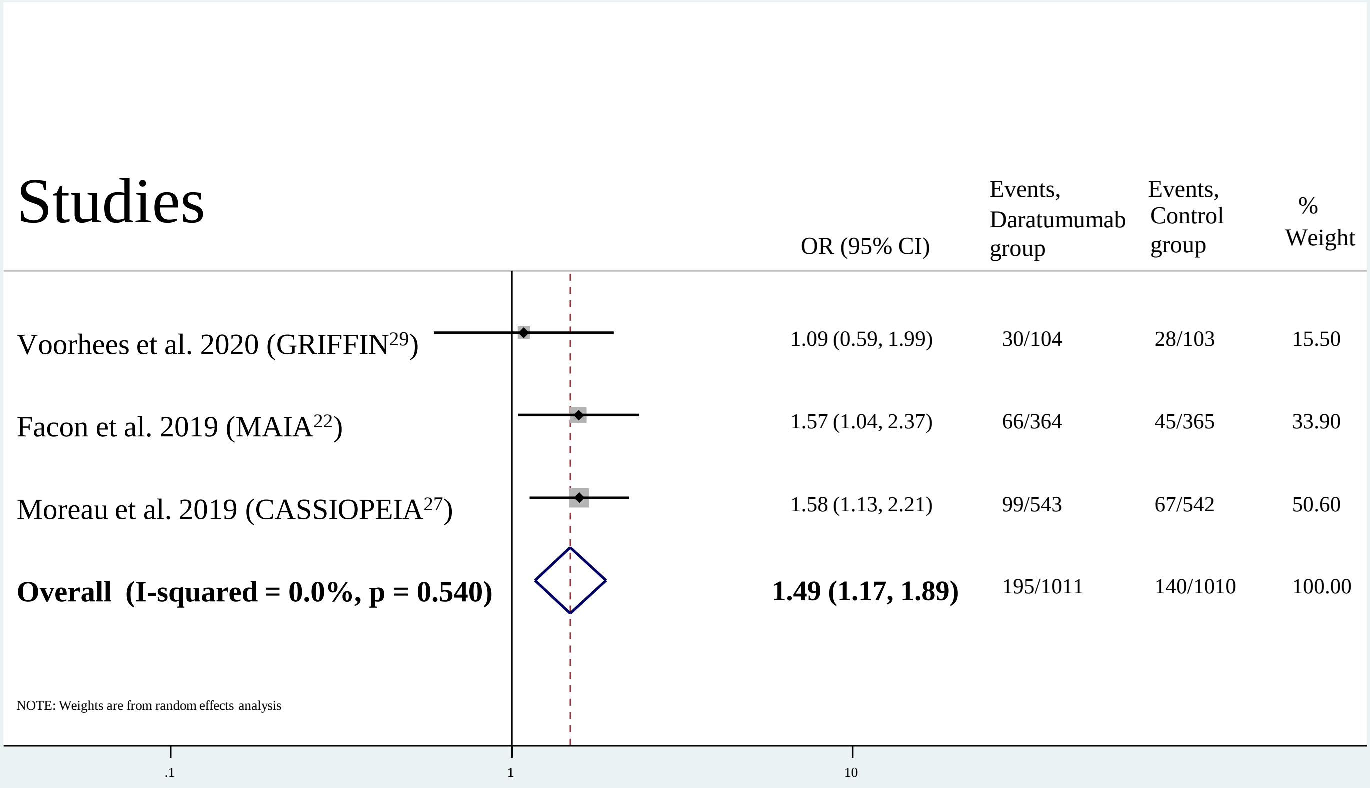

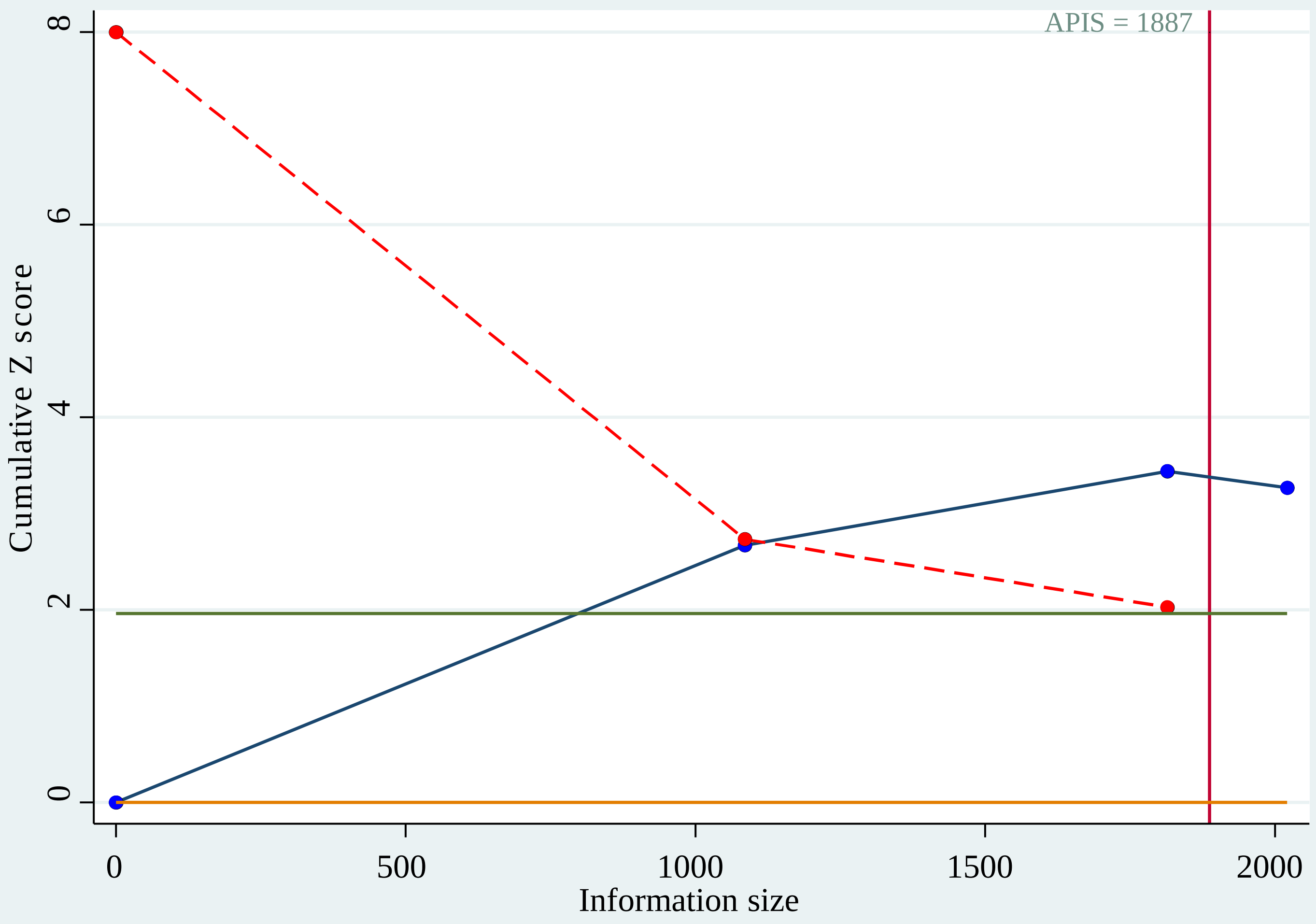

Supplementary Figure 18: Risk of bias assessment at study level and at domain level regarding all grade lymphopenia in newly diagnosed multiple myeloma

DVMP, daratumumab, bortezomib, melphalan, and prednisone; VMP, bortezomib, melphalan, and prednisone; DVTd, daratumumab, bortezomib, thalidomide, and dexamethasone; VTd, bortezomib, thalidomide, and dexamethasone; DRd, daratumumab, lenalidomide and dexamethasone; Rd, lenalidomide and dexamethasone

Study	Interventions	Randomization process	Deviations from intended interventions	Missing outcome data	Measurement of the outcome	Selection of the reported results	Overall assessment
Voorhees et al. 2020	DRVd vs RVd	?	+	+	+	+	?
Moreau et al. 2019	DVTd vs VTd	+	+	+	+	?	+
Facon et al. 2019	DRd vs Rd	+	+	+	+	+	+

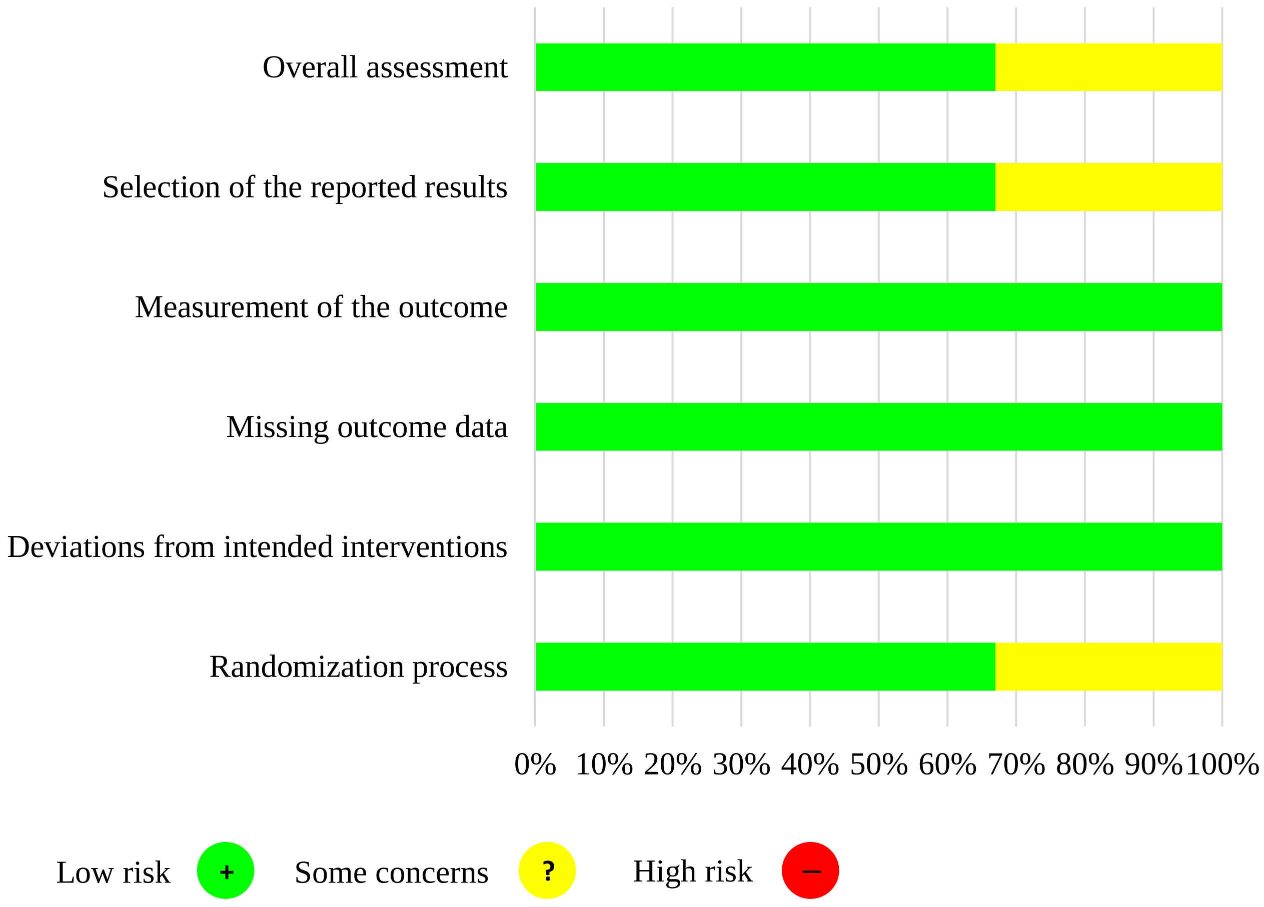

Supplementary Figure 19: Forest plot and Trial Sequential Analysis representing that addition of daratumumab to backbone therapies increases the chance for grade 3-4 lymphopenia in newly diagnosed multiple myeloma

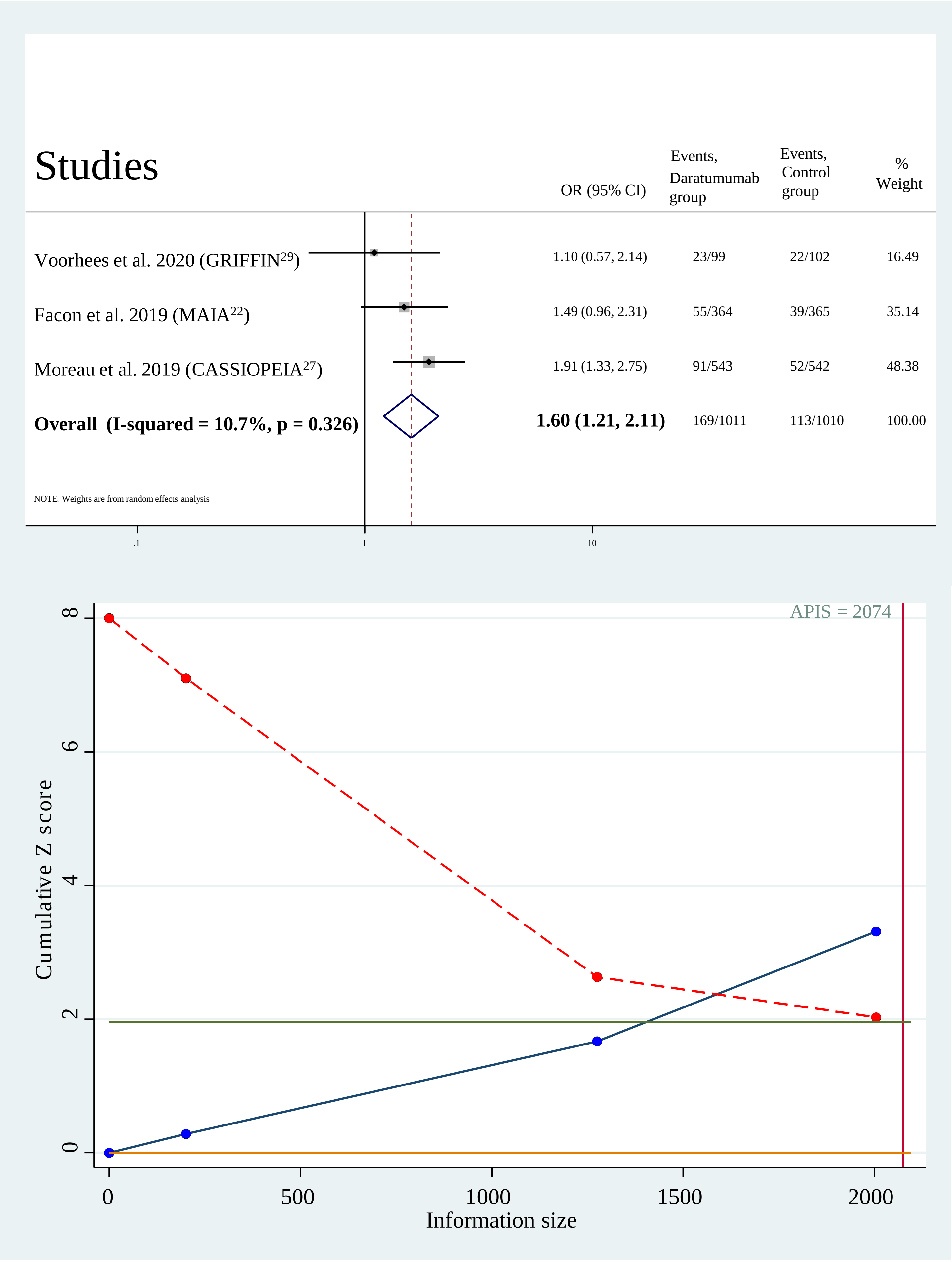

Supplementary Figure 20: Risk of bias assessment at study level and at domain level regarding grade 3-4 lymphopenia in newly diagnosed multiple myeloma
DVMP, daratumumab, bortezomib, melphalan, and prednisone; VMP, bortezomib, melphalan, and prednisone; DVTd, daratumumab, bortezomib, thalidomide, and dexamethasone; VTd, bortezomib, thalidomide, and dexamethasone; DRd, daratumumab, lenalidomide and dexamethasone; Rd, lenalidomide and dexamethasone

**Study**	**Interventions**	**Randomization process**	**Deviations from intended interventions**	**Missing outcome data**	**Measurement of the outcome**	**Selection of the reported results**	**Overall assessment**
Voorhees et al. 2020	DRVd vs RVd	?	+	+	+	+	?
Moreau et al. 2019	DVTd vs VTd	+	+	+	+	?	+
Facon et al. 2019	DRd vs Rd	+	+	+	+	+	+

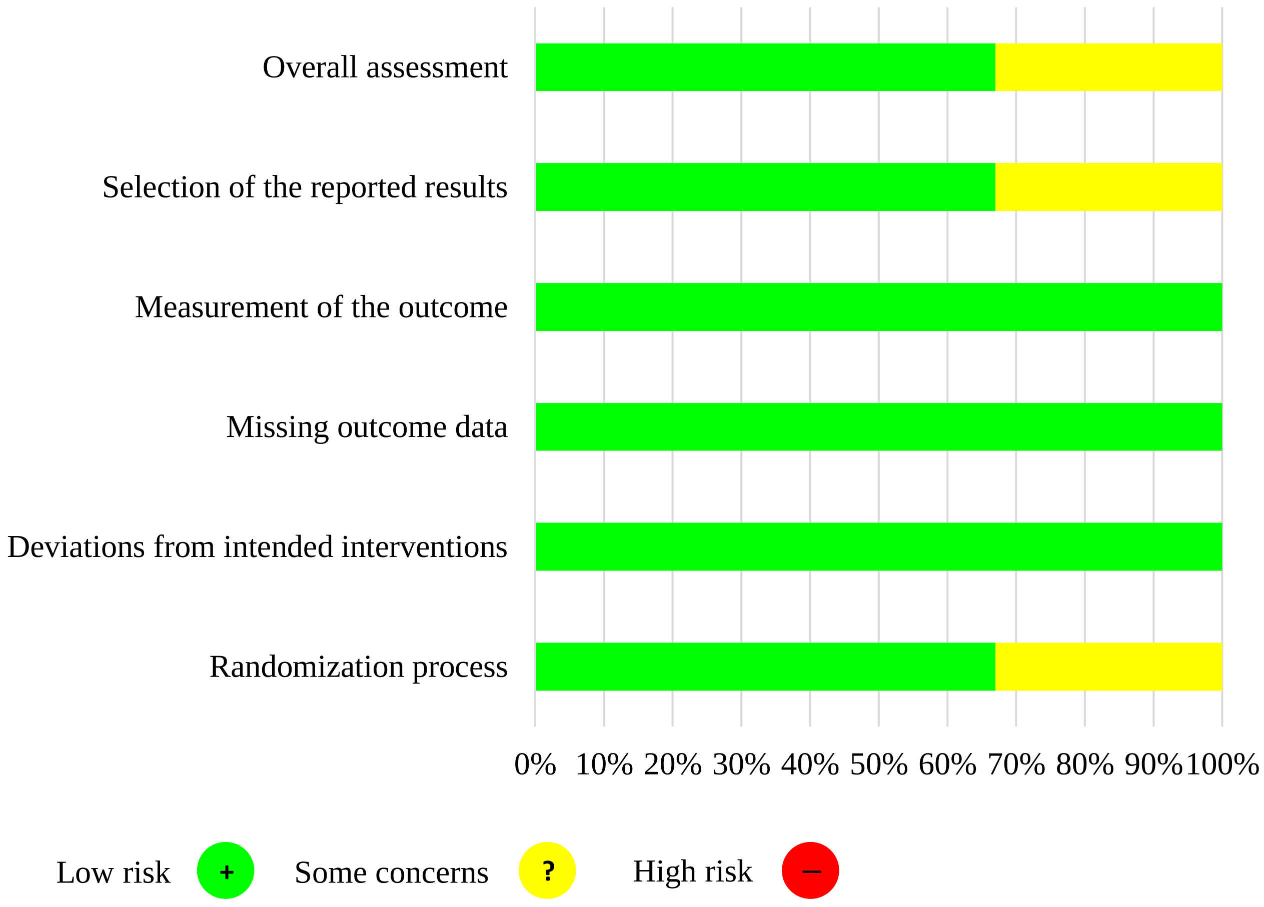

Supplementary Figure 21: Forest plot and Trial Sequential Analysis representing that addition of daratumumab to backbone therapies increases the chance for all grade neutropenia in newly diagnosed multiple myeloma

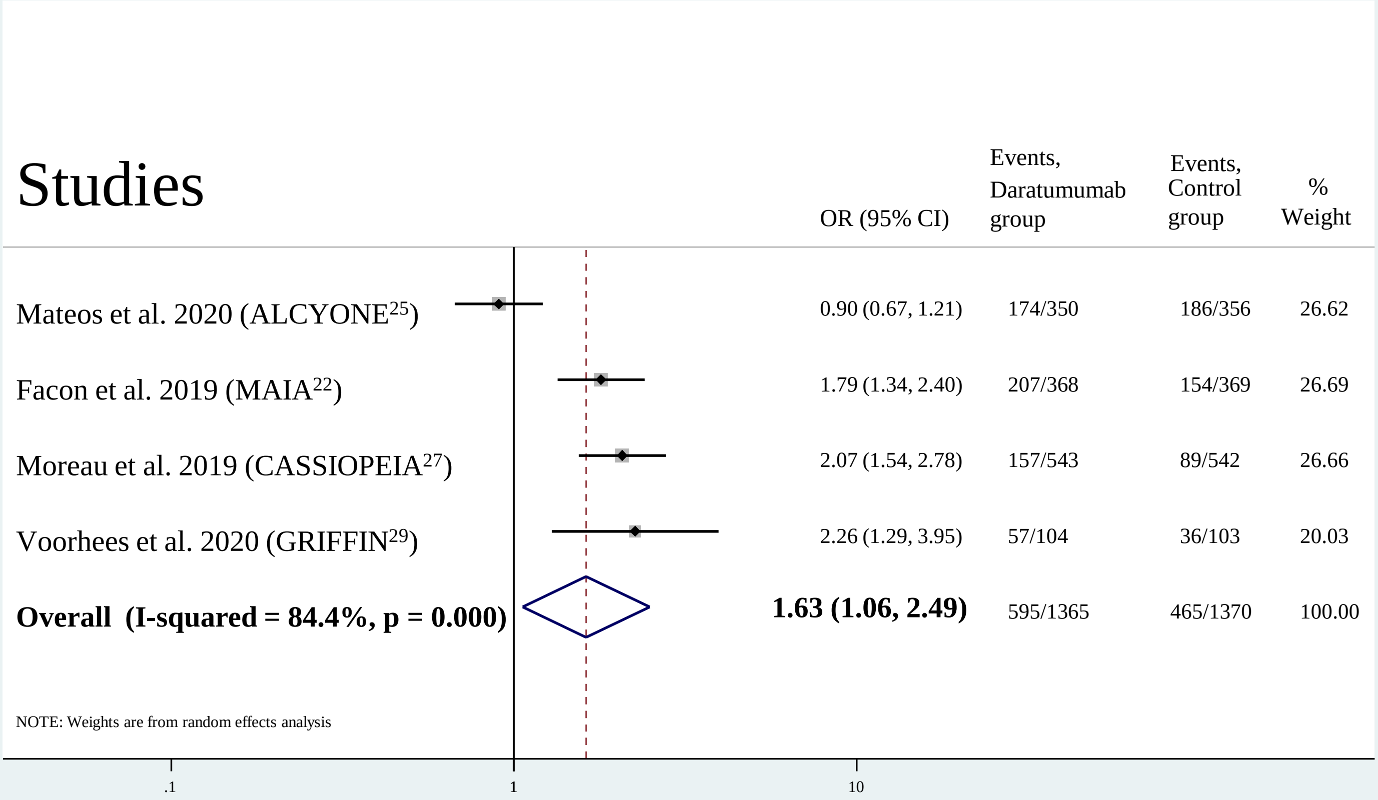

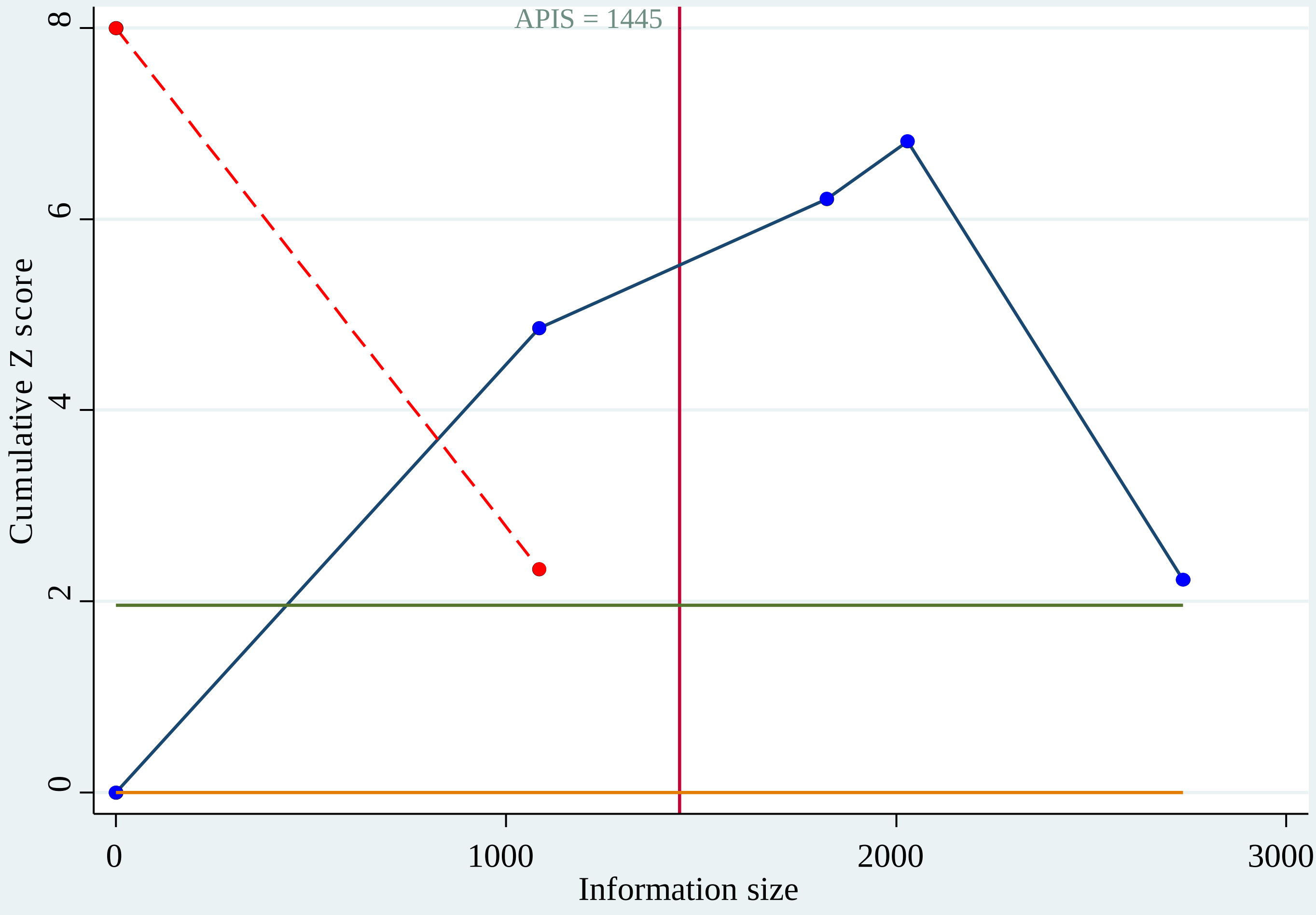

Supplementary Figure 22: Risk of bias assessment at study level and at domain level all grade neutropenia in newly diagnosed multiple myeloma

DRVd, daratumumab, bortezomib, lenalidomide and dexamethasone; RVd, bortezomib, lenalidomide and dexamethasone; DVMP, daratumumab, bortezomib, melphalan, and prednisone; VMP, bortezomib, melphalan, and prednisone; DVTd, daratumumab, bortezomib, thalidomide, and dexamethasone; VTd, bortezomib, thalidomide, and dexamethasone; DRd, daratumumab, lenalidomide and dexamethasone; Rd, lenalidomide and dexamethasone

Study	Interventions	Randomization process	Deviations from intended interventions	Missing outcome data	Measurement of the outcome	Selection of the reported results	Overall assessment
Voorhees et al. 2020	DRVd vs RVd	?	+	+	+	+	?
Mateos et al. 2020	DVMP vs VMP	+	+	+	+	+	+
Moreau et al. 2019	DVTd vs VTd	+	+	+	+	?	+
Facon et al. 2019	DRd vs Rd	+	+	+	+	+	+

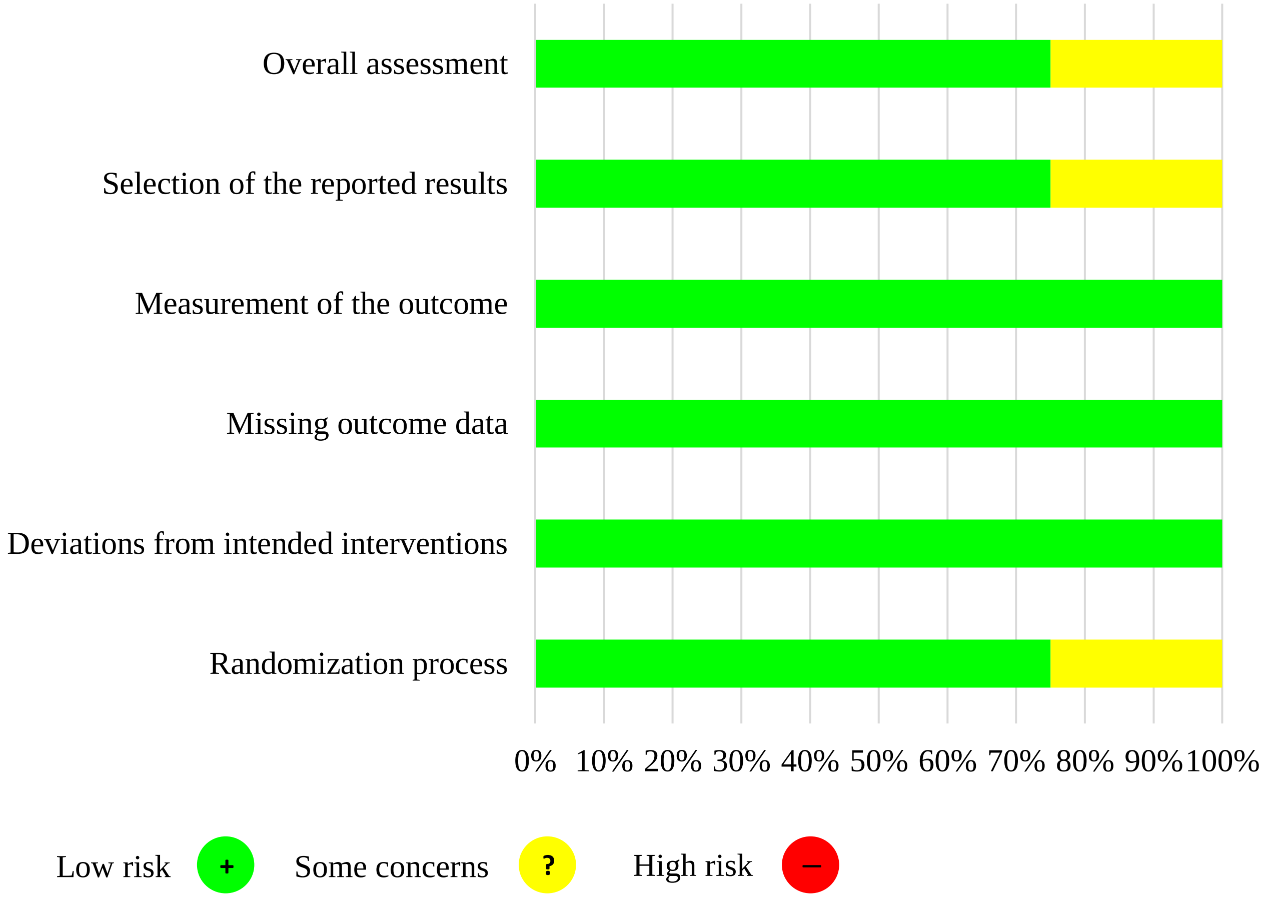

Supplementary Figure 23: Forest plot and Trial Sequential Analysis representing that addition of daratumumab to backbone therapies increases the chance for grade 3-4 neutropenia in newly diagnosed multiple myeloma

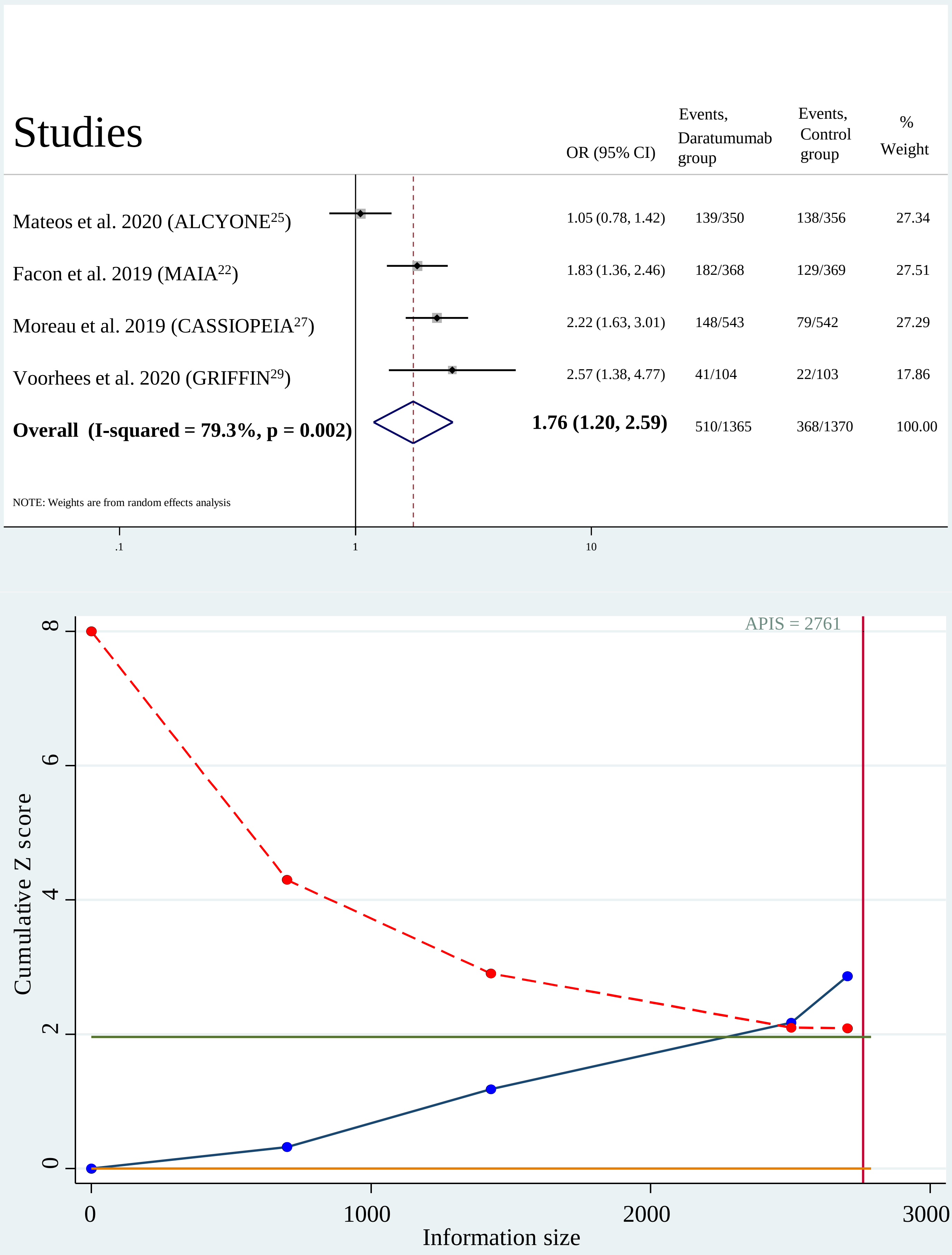

Supplementary Figure 24: Risk of bias assessment at study level and at domain level grade 3-4 neutropenia in newly diagnosed multiple myeloma

DRVd, daratumumab, bortezomib, lenalidomide and dexamethasone; RVd, bortezomib, lenalidomide and dexamethasone; DVMP, daratumumab, bortezomib, melphalan, and prednisone; VMP, bortezomib, melphalan, and prednisone; DVTd, daratumumab, bortezomib, thalidomide, and dexamethasone; VTd, bortezomib, thalidomide, and dexamethasone; DRd, daratumumab, lenalidomide and dexamethasone; Rd, lenalidomide and dexamethasone

Study	Interventions	Randomization process	Deviations from intended interventions	Missing outcome data	Measurement of the outcome	Selection of the reported results	Overall assessment
Voorhees et al. 2020	DRVd vs RVd	?	+	+	+	+	?
Mateos et al. 2020	DVMP vs VMP	+	+	+	+	+	+
Moreau et al. 2019	DVTd vs VTd	+	+	+	+	?	+
Facon et al. 2019	DRd vs Rd	+	+	+	+	+	+

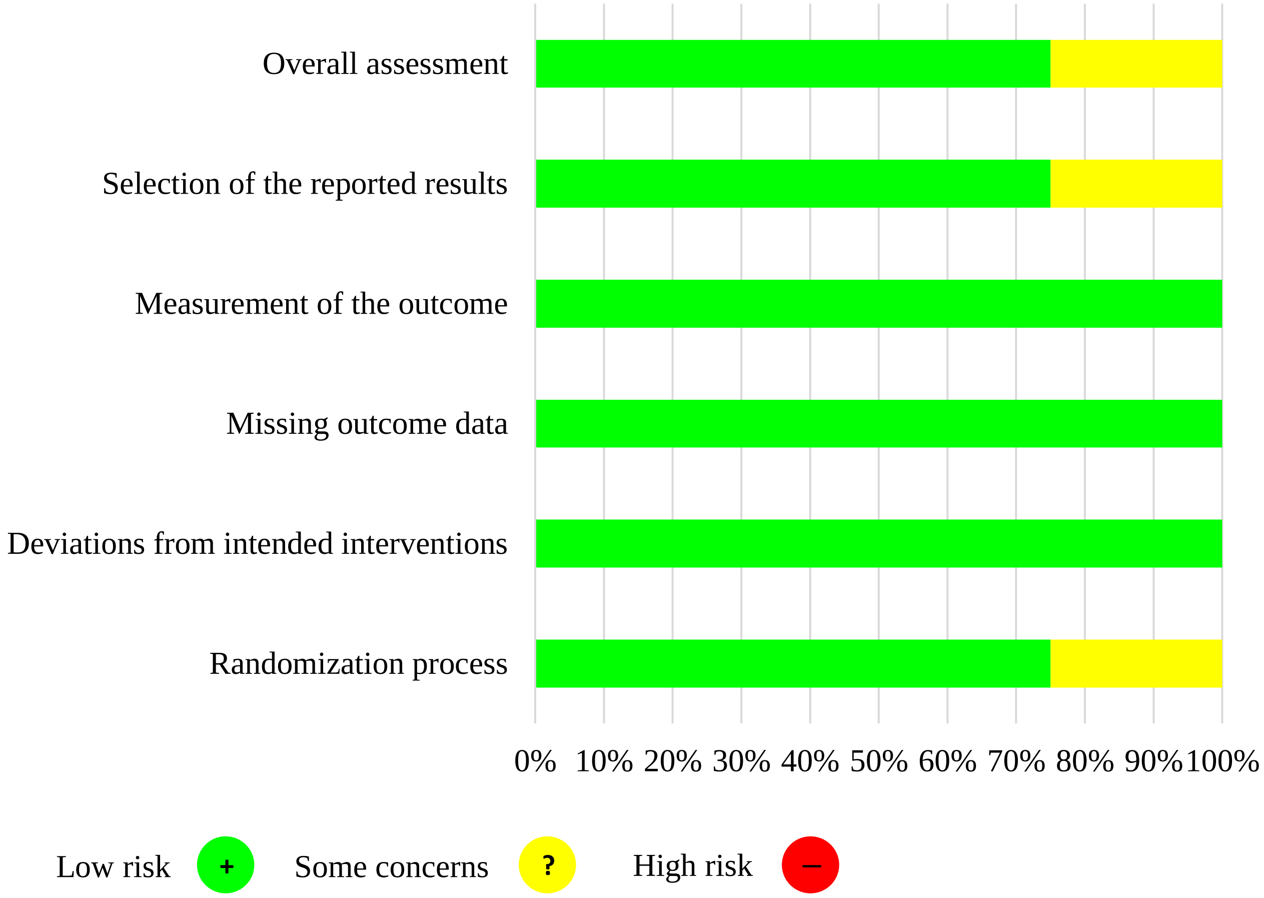

Supplementary Figure 25: Forest plot and Trial Sequential Analysis representing that addition of daratumumab to backbone therapies does not increase the chance for all grade anaemia in newly diagnosed multiple myeloma

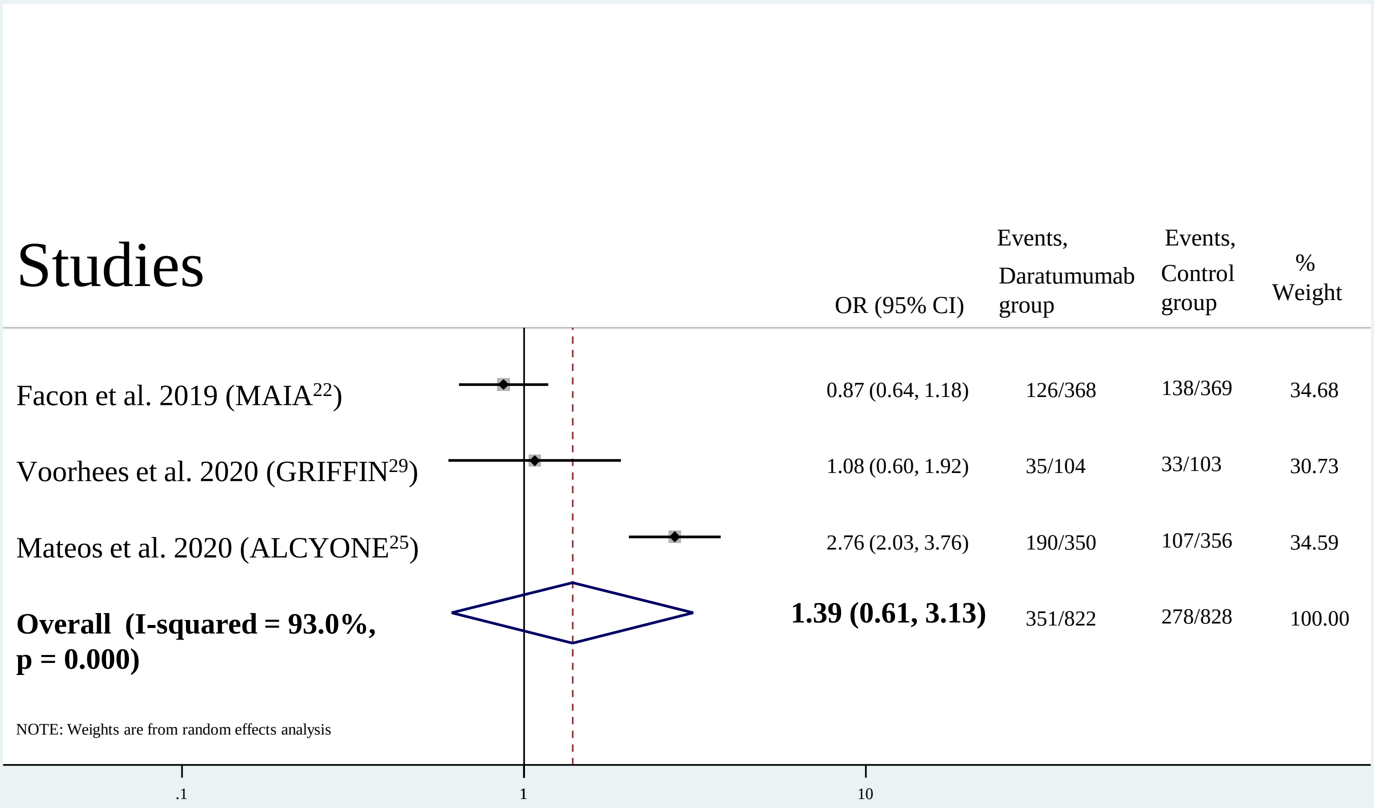

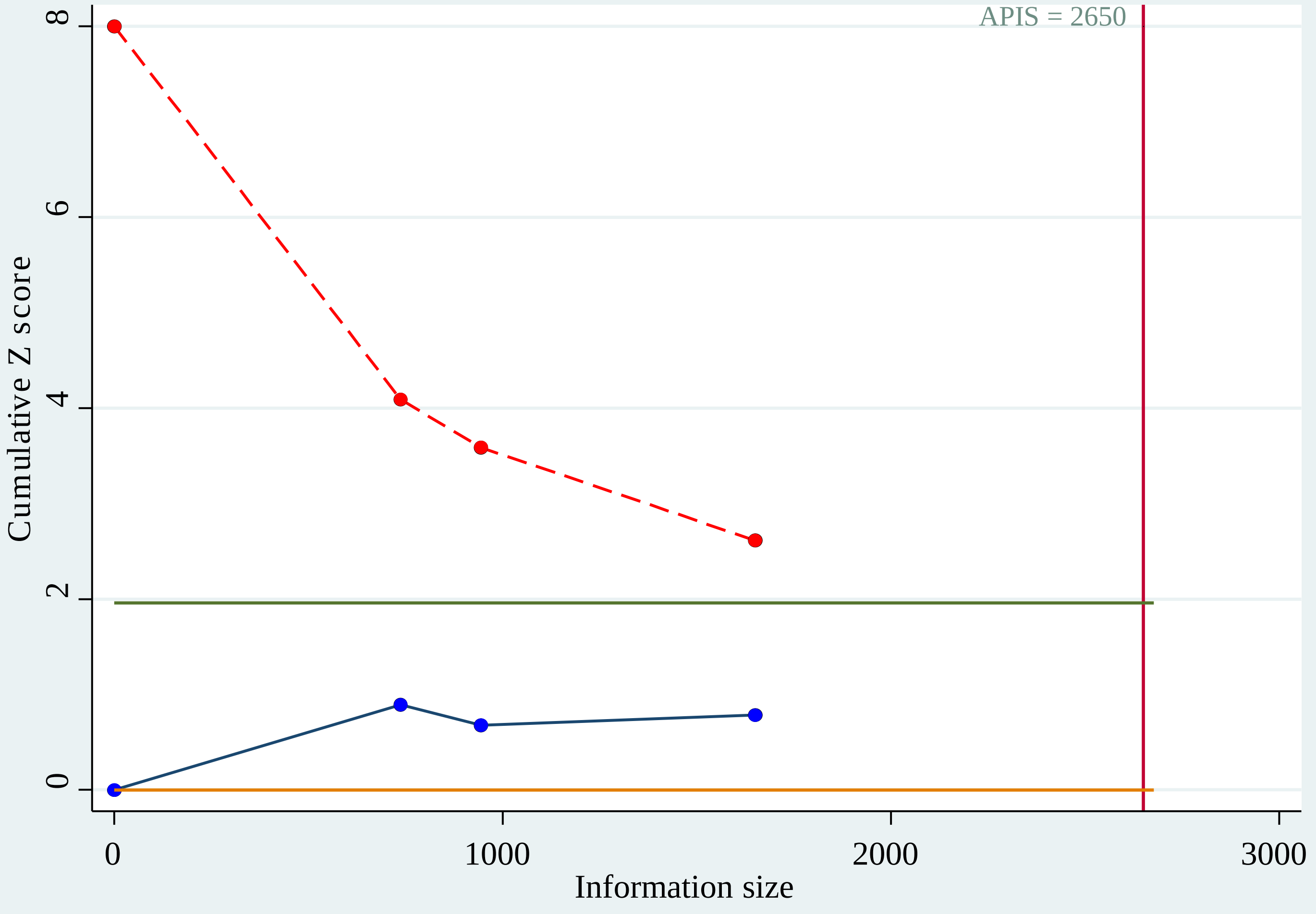

Supplementary Figure 26: Risk of bias assessment at study level and at domain level regarding all grade anaemia in newly diagnosed multiple myeloma

DRVd, daratumumab, bortezomib, lenalidomide and dexamethasone; RVd, bortezomib, lenalidomide and dexamethasone; DVMP, daratumumab, bortezomib, melphalan, and prednisone; VMP, bortezomib, melphalan, and prednisone; DRd, daratumumab, lenalidomide and dexamethasone; Rd, lenalidomide and dexamethasone

Study	Interventions	Randomization process	Deviations from intended interventions	Missing outcome data	Measurement of the outcome	Selection of the reported results	Overall assessment
Voorhees et al. 2020	DRVd vs RVd	?	+	+	+	+	?
Mateos et al. 2020	DVMP vs VMP	+	+	+	+	+	+
Facon et al. 2019	DRd vs Rd	+	+	+	+	+	+

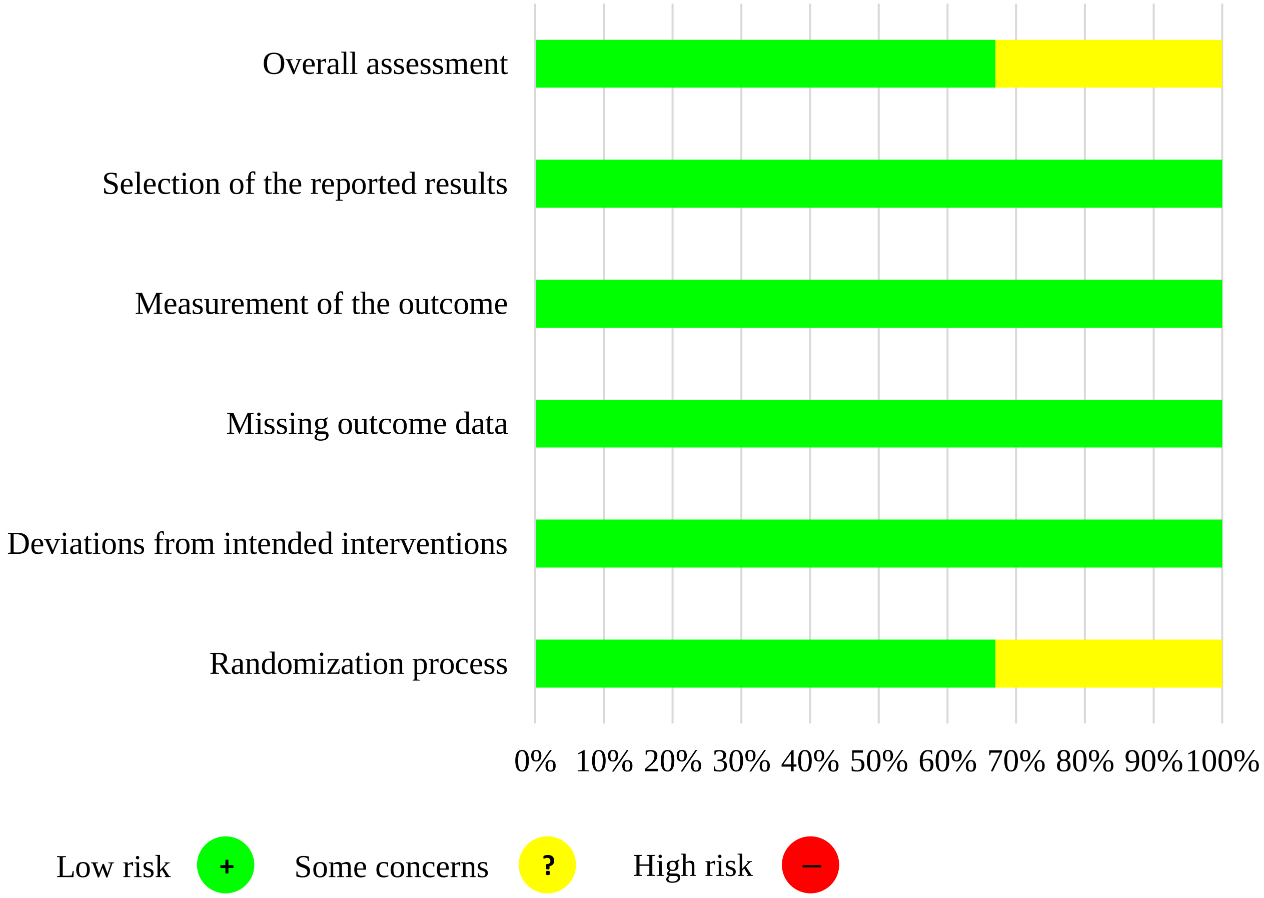

Supplementary Figure 27: Forest plot and Trial Sequential Analysis representing that addition of daratumumab to backbone therapies does not increase the chance for grade 3-4 anaemia in newly diagnosed multiple myeloma

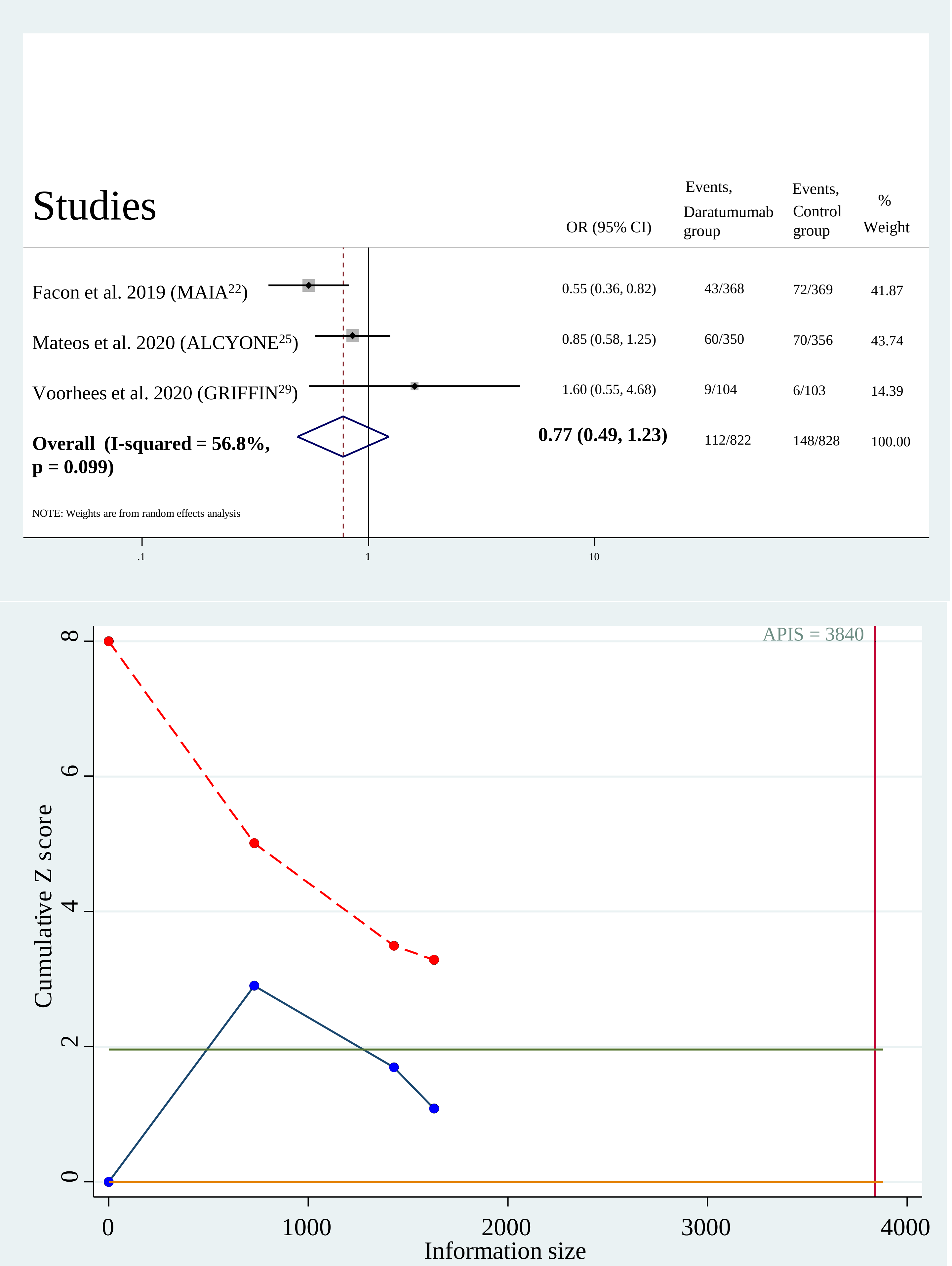

Supplementary Figure 28: Risk of bias assessment at study level and at domain level regarding all grade anaemia in newly diagnosed multiple myeloma

DRVd, daratumumab, bortezomib, lenalidomide and dexamethasone; RVd, bortezomib, lenalidomide and dexamethasone; DVMP, daratumumab, bortezomib, melphalan, and prednisone; VMP, bortezomib, melphalan, and prednisone; DRd, daratumumab, lenalidomide and dexamethasone; Rd, lenalidomide and dexamethasone

Study	Interventions	Randomization process	Deviations from intended interventions	Missing outcome data	Measurement of the outcome	Selection of the reported results	Overall assessment
Voorhees et al. 2020	DRVd vs RVd	?	+	+	+	+	?
Mateos et al. 2020	DVMP vs VMP	+	+	+	+	+	+
Facon et al. 2019	DRd vs Rd	+	+	+	+	+	+

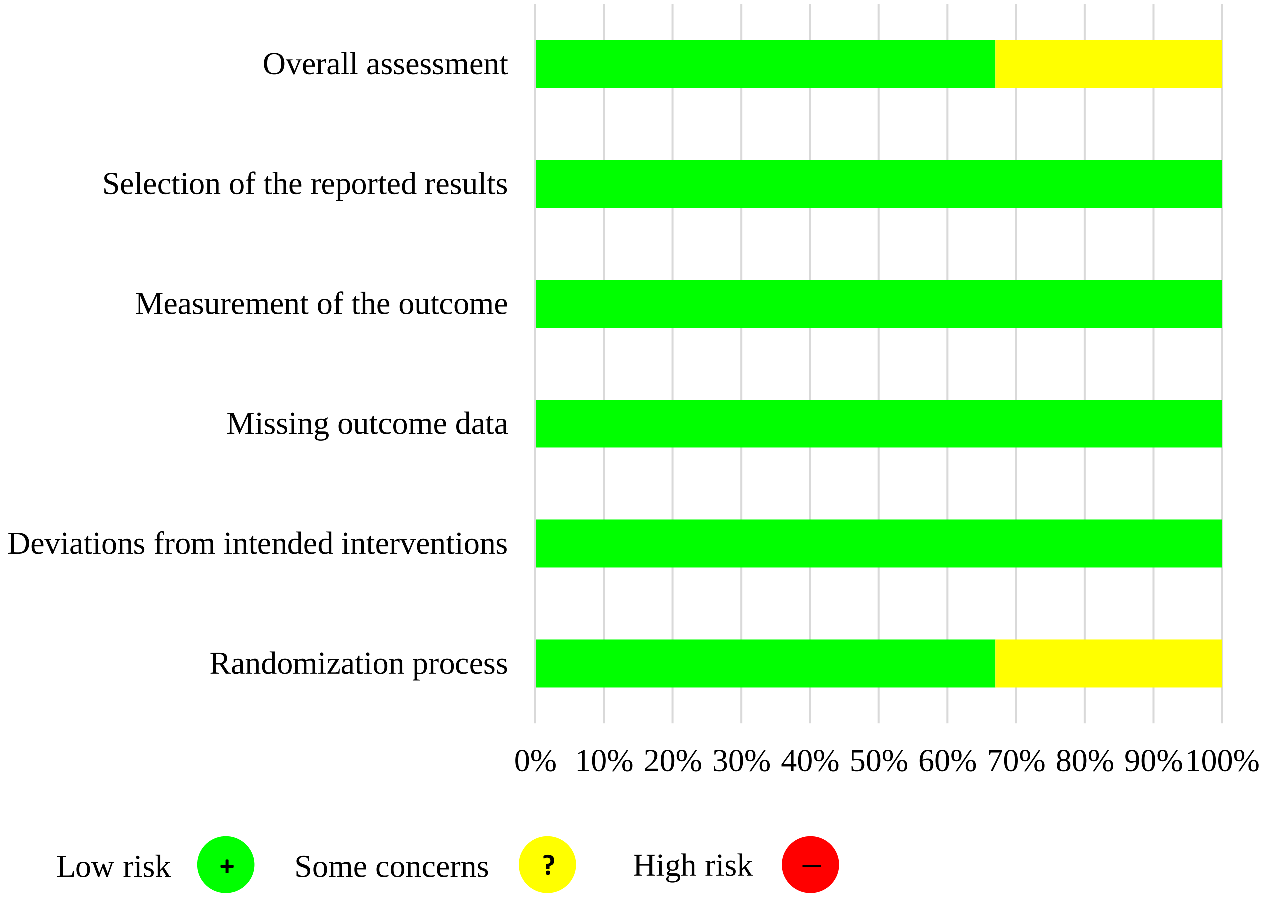

Supplementary Figure 29: Forest plot and Trial Sequential Analysis representing that addition of daratumumab to backbone therapies is associated with decreased chance for all grade peripheral neuropathy in newly diagnosed multiple myeloma

Studies	OR (95% CI)	Events, Daratumumab group	Events, Control group	% Weight
Voorhees et al. 2020 (GRIFFIN[29])	0.51 (0.29, 0.92)	59/104	74/103	10.50
Mateos et al. 2020 (ALCYONE[25])	0.77 (0.56, 1.06)	100/350	122/356	33.58
Moreau et al. 2019 (CASSIOPEIA[27])	0.81 (0.64, 1.04)	314/543	340/542	55.92
**Overall (I-squared = 3.5%, p = 0.355)**	**0.76 (0.63, 0.92)**	473/997	536/1001	100.00

NOTE: Weights are from random effects analysis

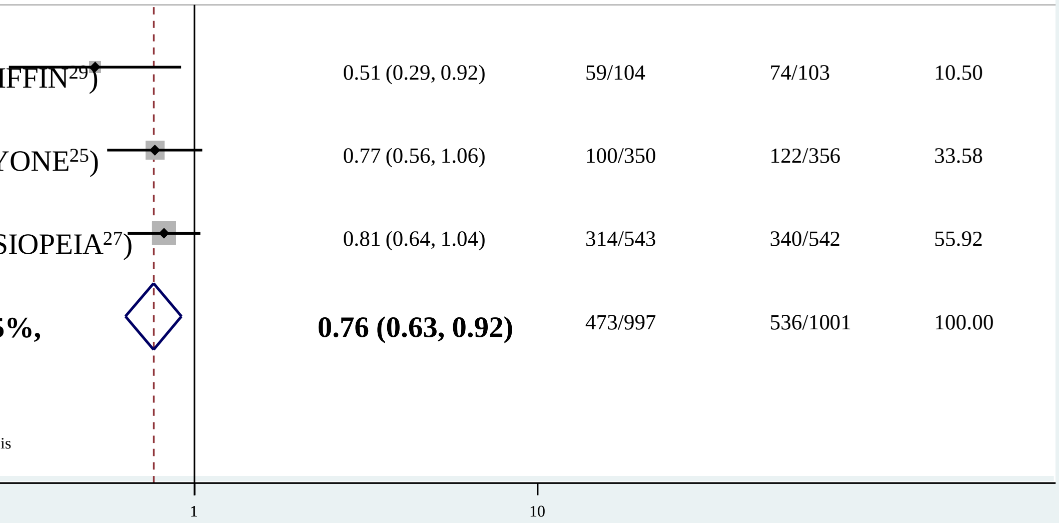

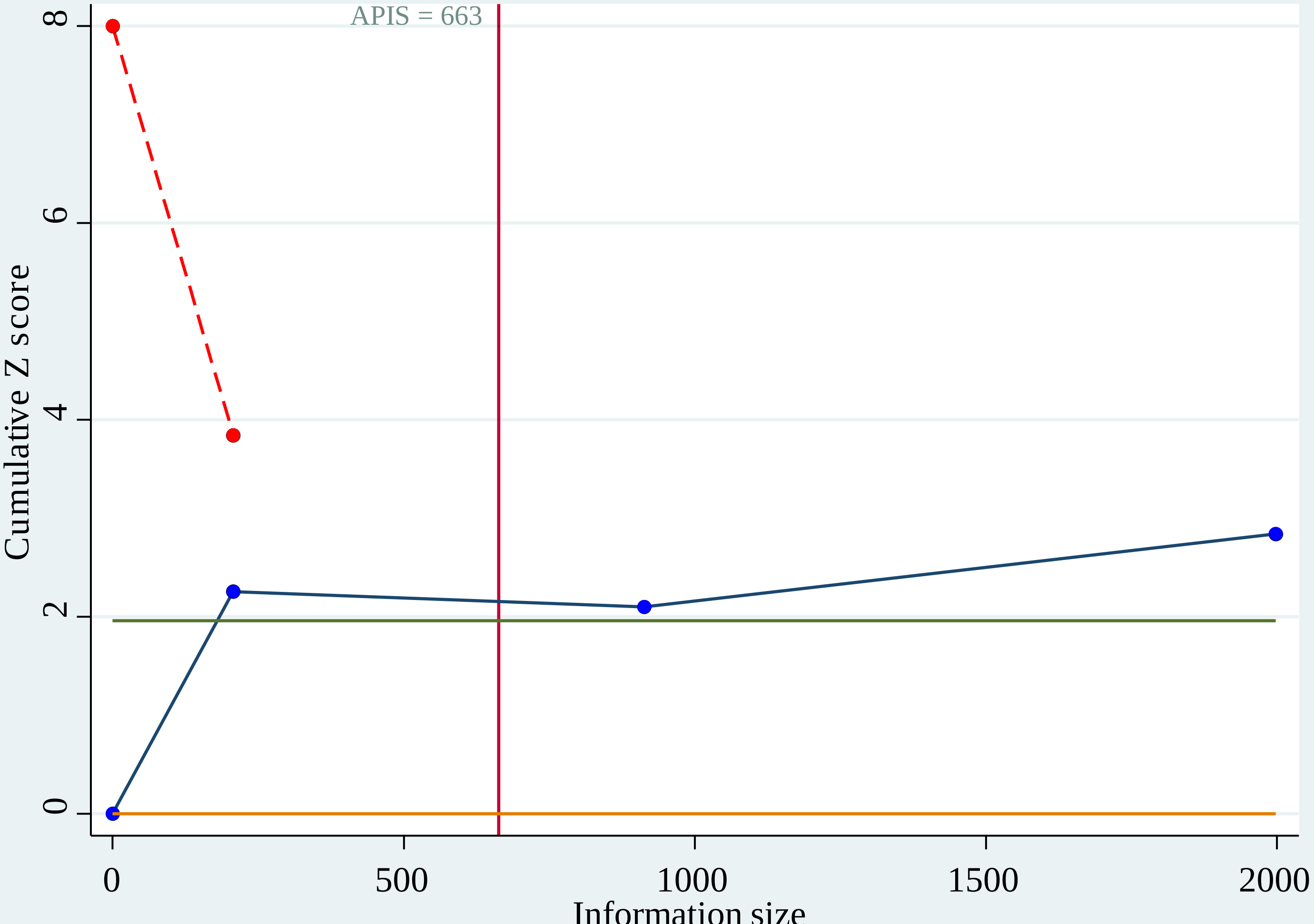

Supplementary Figure 30: Risk of bias assessment at study level and at domain level regarding all grade peripheral neuropathy in newly diagnosed multiple myeloma
DRVd, daratumumab, bortezomib, lenalidomide and dexamethasone; RVd, bortezomib, lenalidomide and dexamethasone; DVMP, daratumumab, bortezomib, melphalan, and prednisone; VMP, bortezomib, melphalan, and prednisone; DVTd, daratumumab, bortezomib, thalidomide, and dexamethasone; VTd, bortezomib, thalidomide, and dexamethasone

**Study**	**Interventions**	**Randomization process**	**Deviations from intended interventions**	**Missing outcome data**	**Measurement of the outcome**	**Selection of the reported results**	**Overall assessment**
Voorhees et al. 2020	DRVd vs RVd	?	+	+	+	+	?
Mateos et al. 2020	DVMP vs VMP	+	+	+	+	+	+
Moreau et al. 2019	DVTd vs VTd	+	+	+	+	?	+

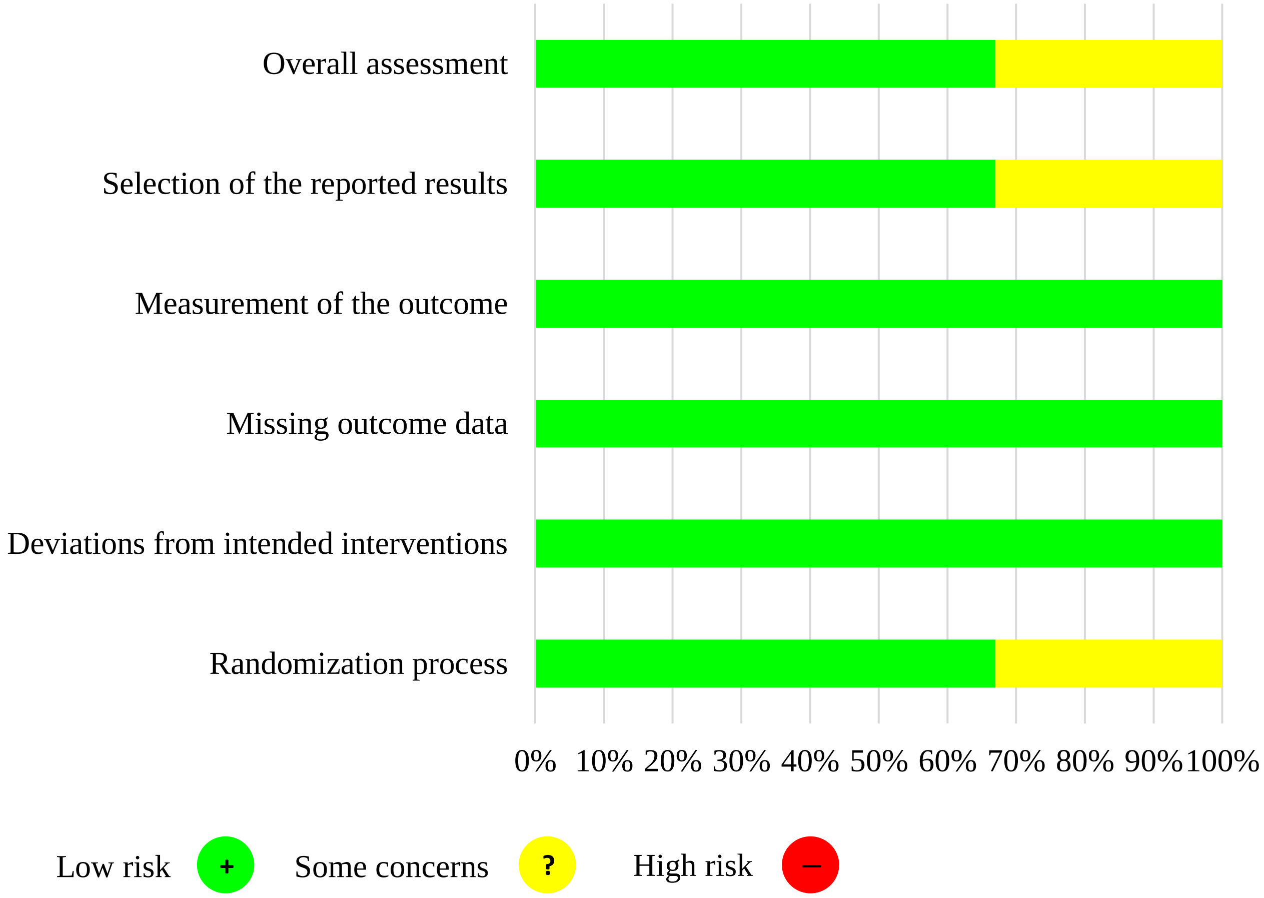

Supplementary Figure 31: Forest plot and Trial Sequential Analysis representing that addition of daratumumab to backbone therapies does not increase the chance for grade 3-4 peripheral neuropathy in newly diagnosed multiple myeloma

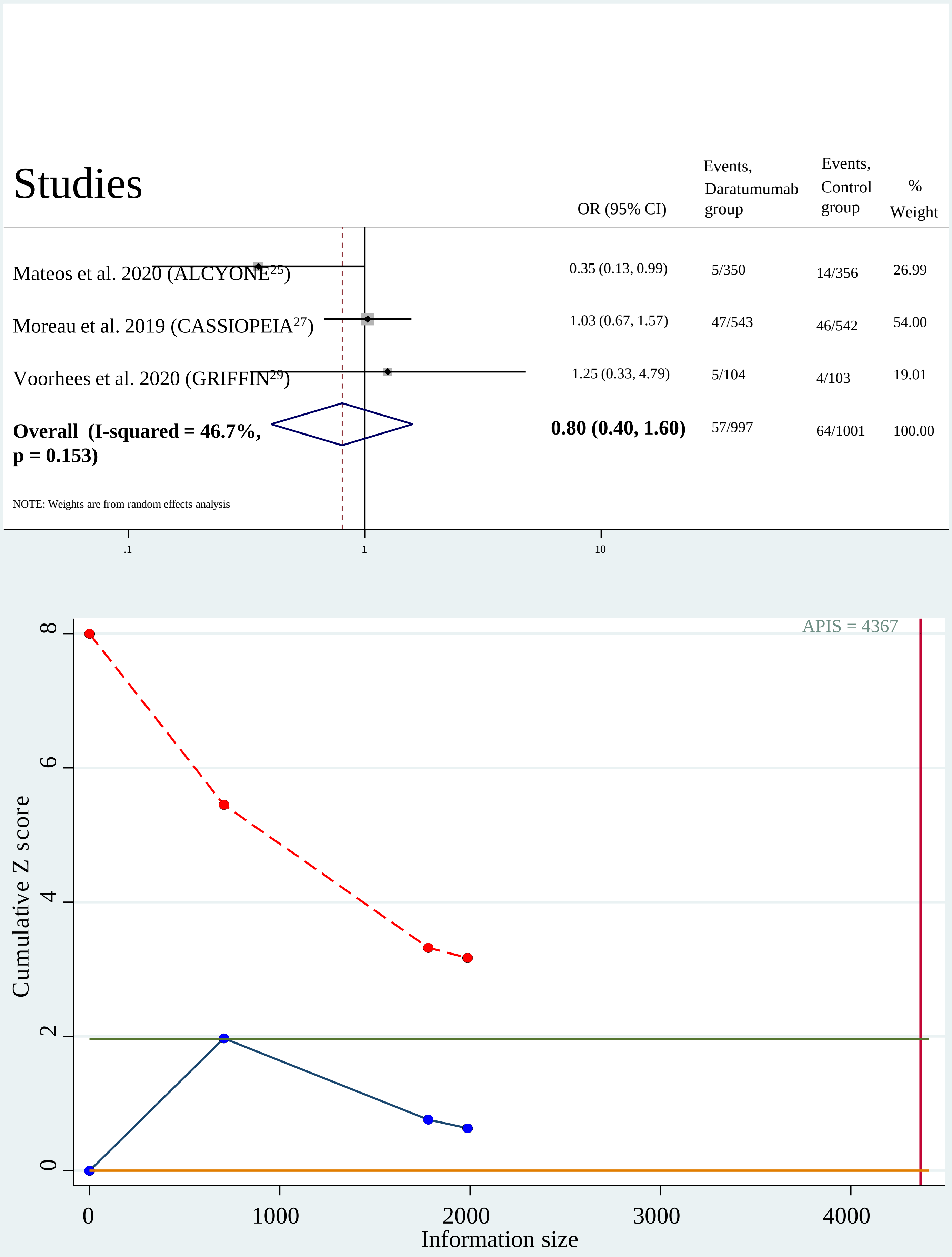

Supplementary Figure 32: Risk of bias assessment at study level and at domain level regarding grade 3-4 peripheral neuropathy in newly diagnosed multiple myeloma
DRVd, daratumumab, bortezomib, lenalidomide and dexamethasone; RVd, bortezomib, lenalidomide and dexamethasone; DVMP, daratumumab, bortezomib, melphalan, and prednisone; VMP, bortezomib, melphalan, and prednisone; DVTd, daratumumab, bortezomib, thalidomide, and dexamethasone; VTd, bortezomib, thalidomide, and dexamethasone

**Study**	**Interventions**	**Randomization process**	**Deviations from intended interventions**	**Missing outcome data**	**Measurement of the outcome**	**Selection of the reported results**	**Overall assessment**
Voorhees et al. 2020	DRVd vs RVd	?	+	+	+	+	?
Mateos et al. 2020	DVMP vs VMP	+	+	+	+	+	+
Moreau et al. 2019	DVTd vs VTd	+	+	+	+	?	+

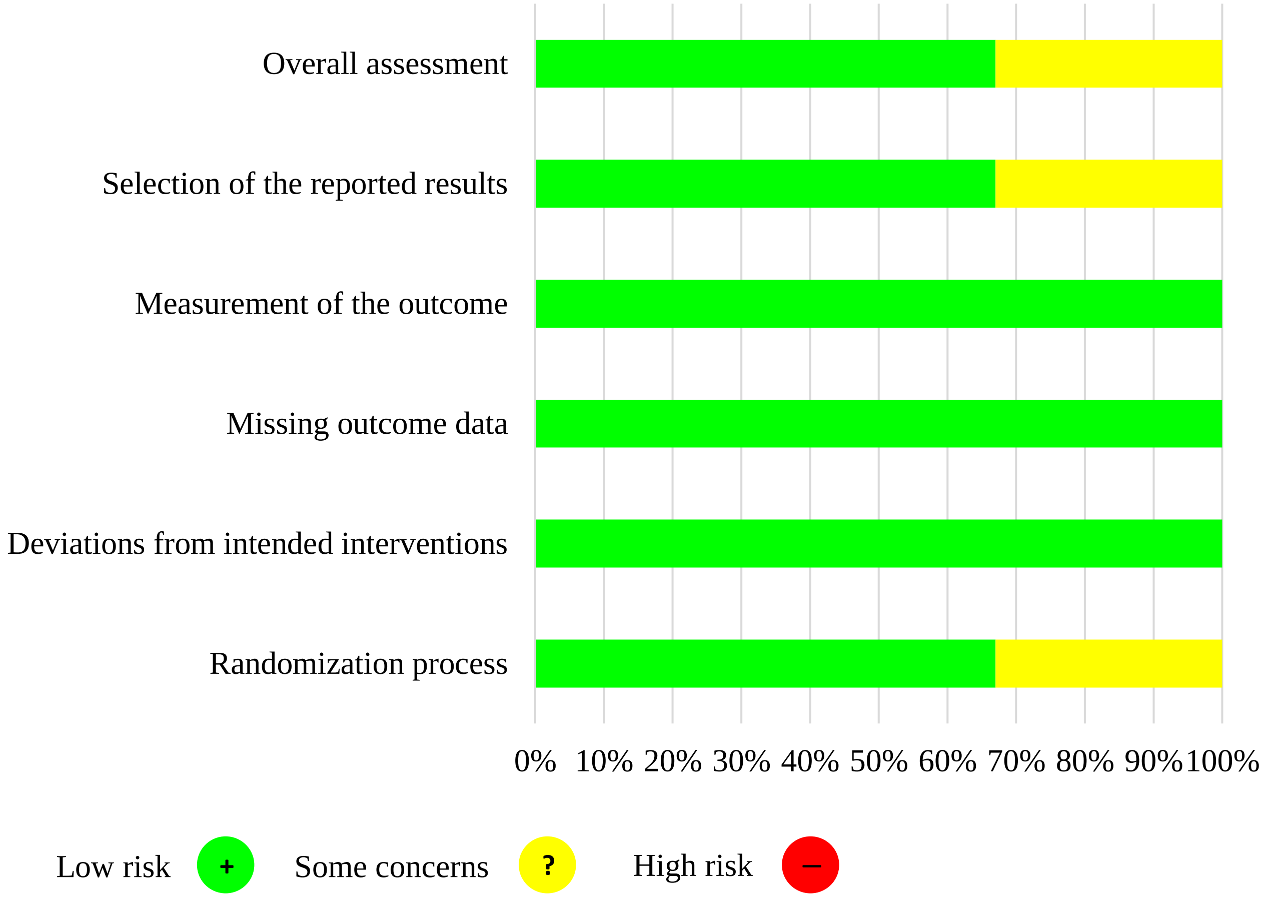

Supplementary Figure 33: Forest plot representing that addition of daratumumab to backbone therapies is not associated with increased chance for second primary cancer in newly diagnosed multiple myeloma. Trial Sequential Analysis could not be carried out due to low event number.

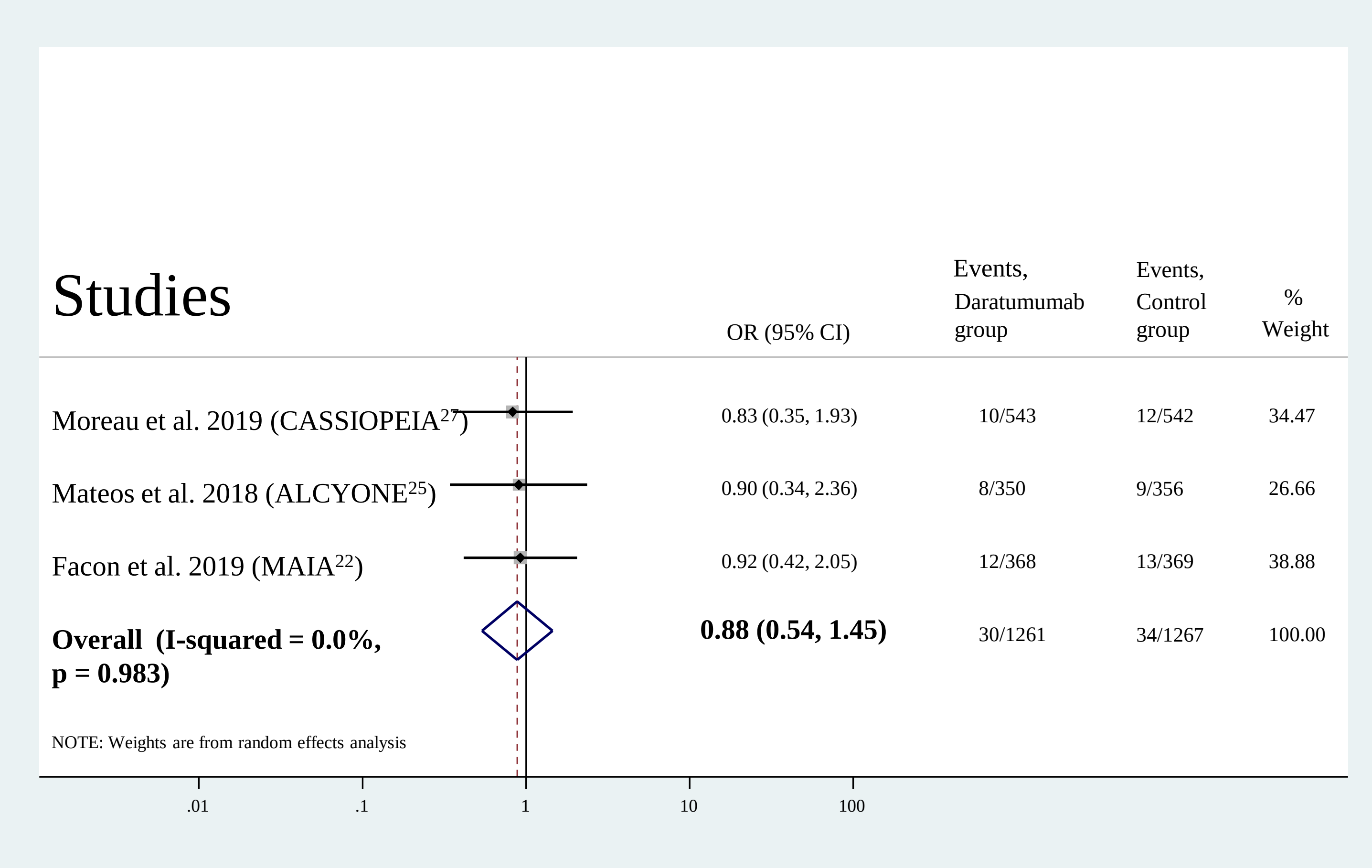

Supplementary Figure 34: Risk of bias assessment at study level and at domain level regarding second primary cancer in newly diagnosed multiple myeloma
DVMP, daratumumab, bortezomib, melphalan, and prednisone; VMP, bortezomib, melphalan, and prednisone; DVTd, daratumumab, bortezomib, thalidomide, and dexamethasone; VTd, bortezomib, thalidomide, and dexamethasone; DRd, daratumumab, lenalidomide and dexamethasone; Rd, lenalidomide and dexamethasone

Study	Interventions	Randomization process	Deviations from intended interventions	Missing outcome data	Measurement of the outcome	Selection of the reported results	Overall assessment
Mateos et al. 2020	DVMP vs VMP	+	+	+	+	+	+
Moreau et al. 2019	DVTd vs VTd	+	+	+	+	?	+
Facon et al. 2019	DRd vs Rd	+	+	+	+	+	+

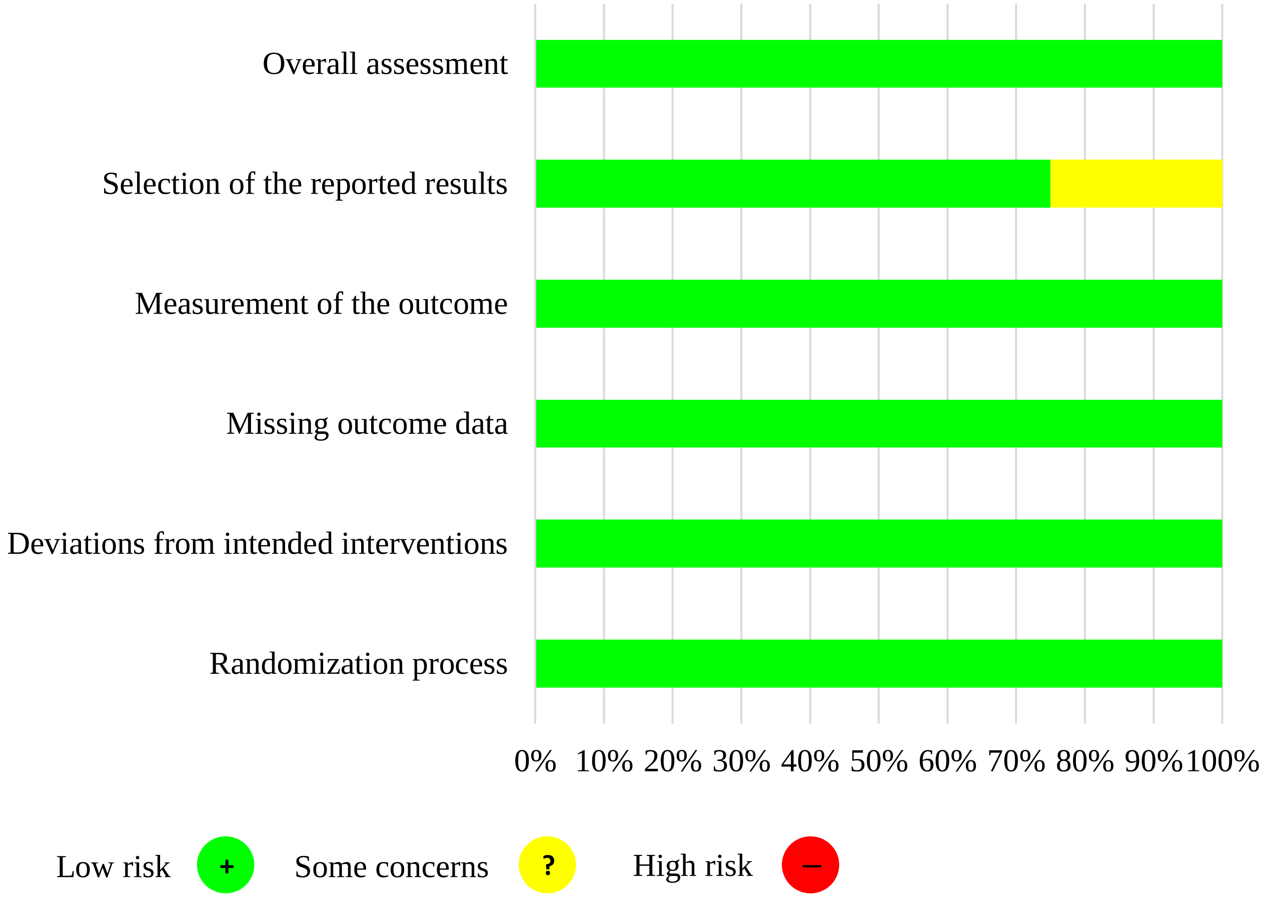

Supplementary Figure 35: Forest plot and Trial Sequential Analysis representing that addition of daratumumab to backbone therapies increases the chance for achieving minimal residual disease negativity in relapsed/refractory multiple myeloma

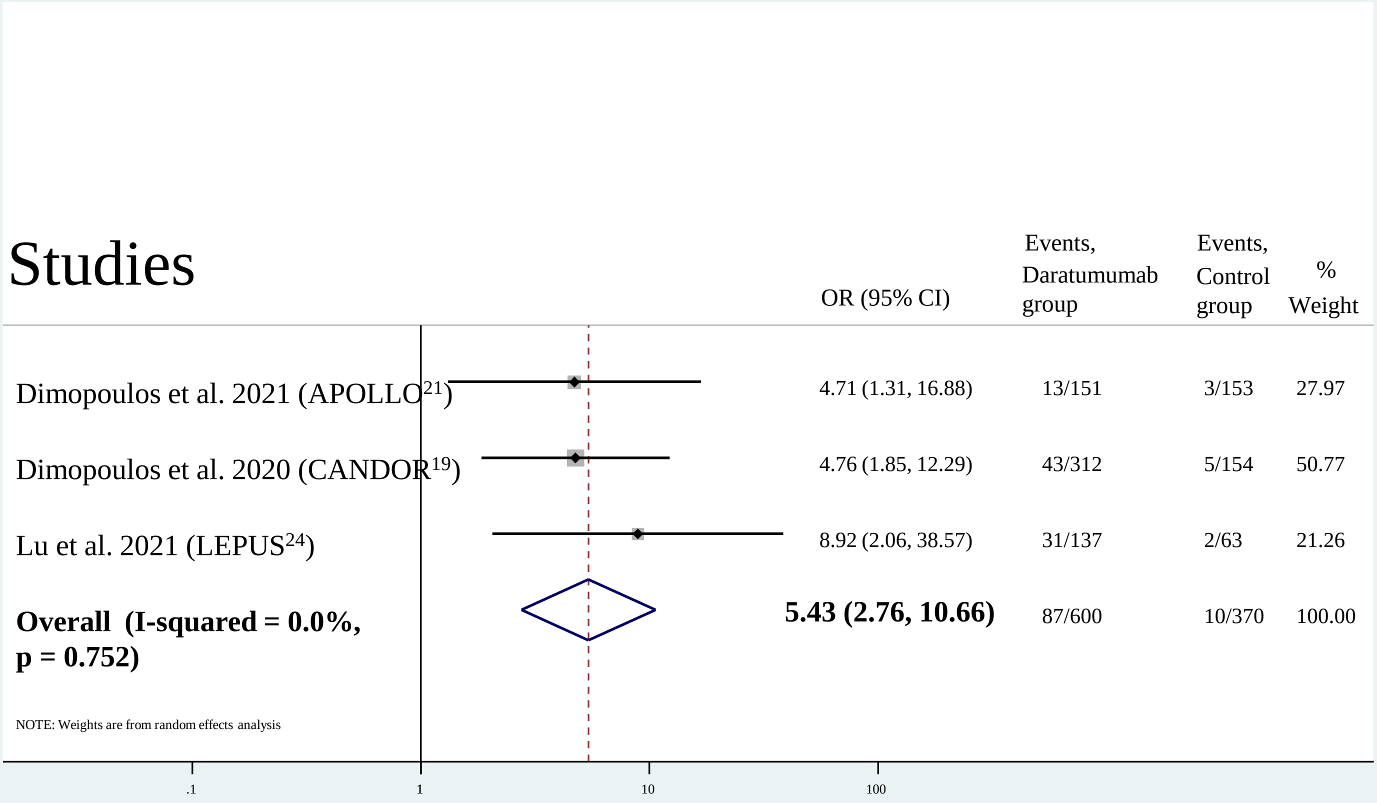

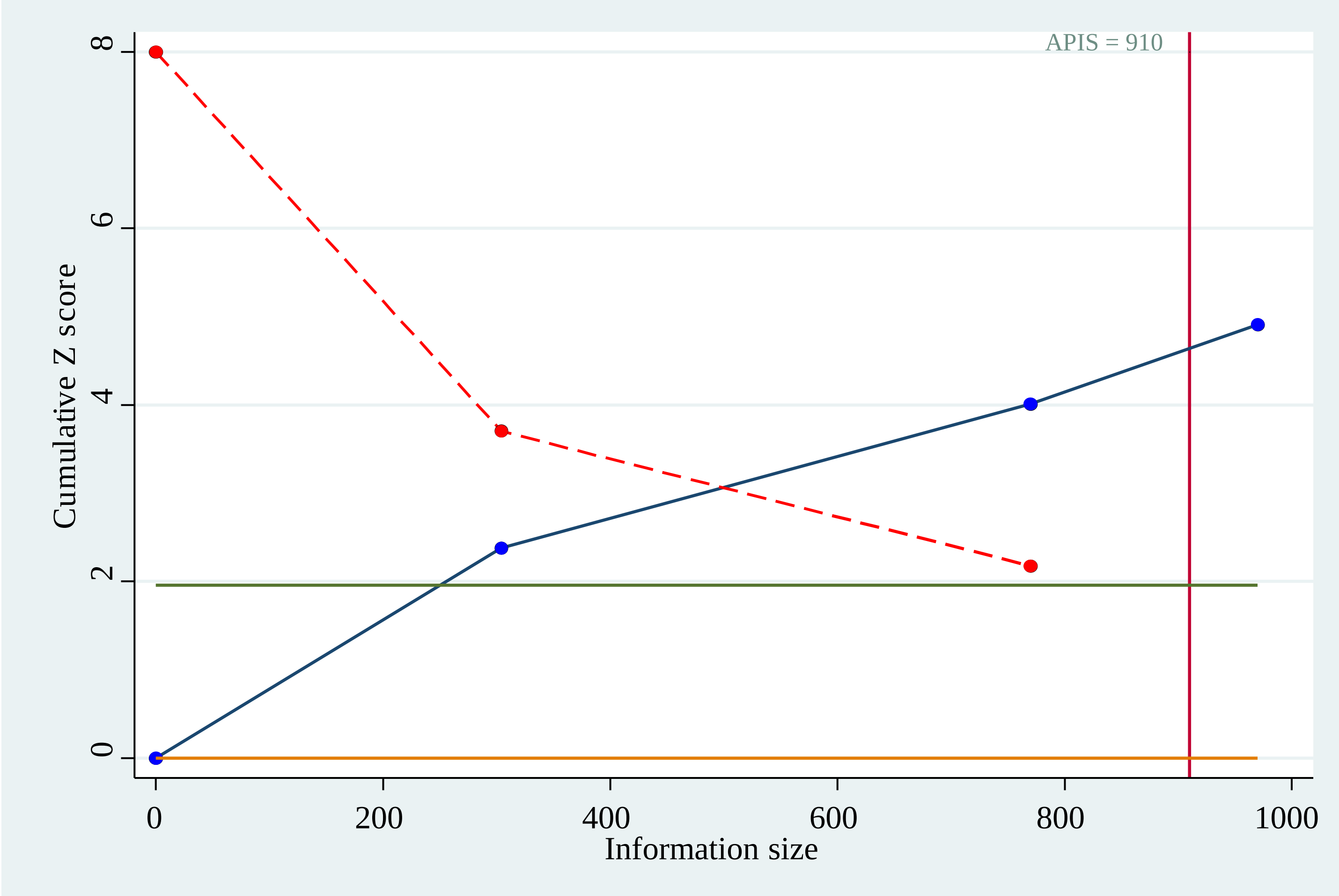

Supplementary Figure 36: Risk of bias assessment at study level and at domain level regarding minimal residual disease negativity in relapsed/refractory multiple myeloma
DPd, daratumumab, pomalidomide, and dexamethasone; Pd, pomalidomide and dexamethasone; DVd, daratumumab, bortezomib, and dexamethasone; Vd, bortezomib and dexamethasone; DRd, daratumumab, lenalidomide and dexamethasone; Rd, lenalidomide and dexamethasone

Study	Interventions	Randomization process	Deviations from intended interventions	Missing outcome data	Measurement of the outcome	Selection of the reported results	Overall assessment
Dimopoulos et al. 2021	DPd vs Pd	+	+	+	+	?	+
Lu et al. 2021	DVd vs Vd	?	+	+	+	?	?
Dimopoulos et al. 2016	DRd vs Rd	+	+	+	+	?	+

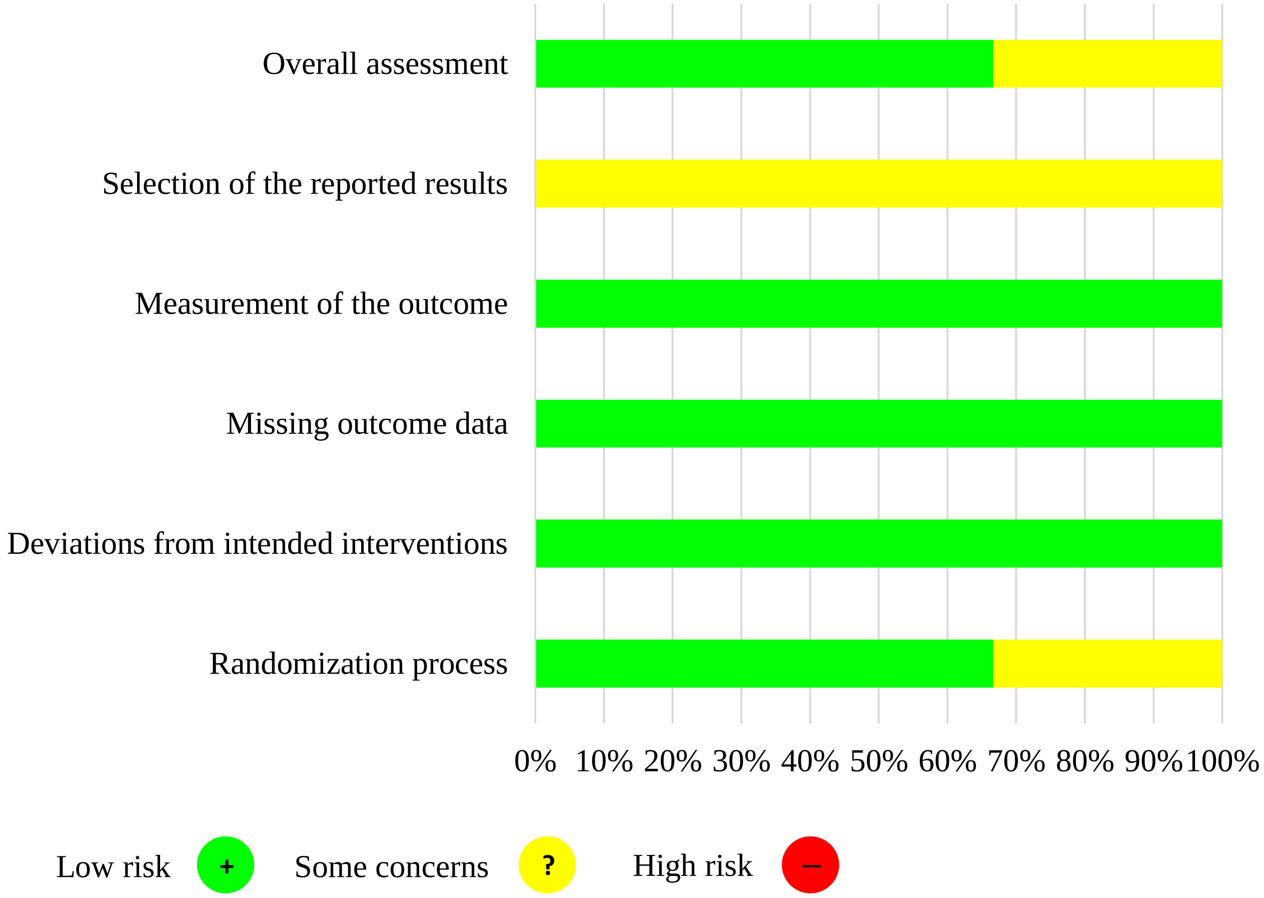

Supplementary Figure 37: Forest plot and Trial Sequential Analysis representing that addition of daratumumab to backbone therapies increases the chance for achieving stringent complete response in relapsed/refractory multiple myeloma

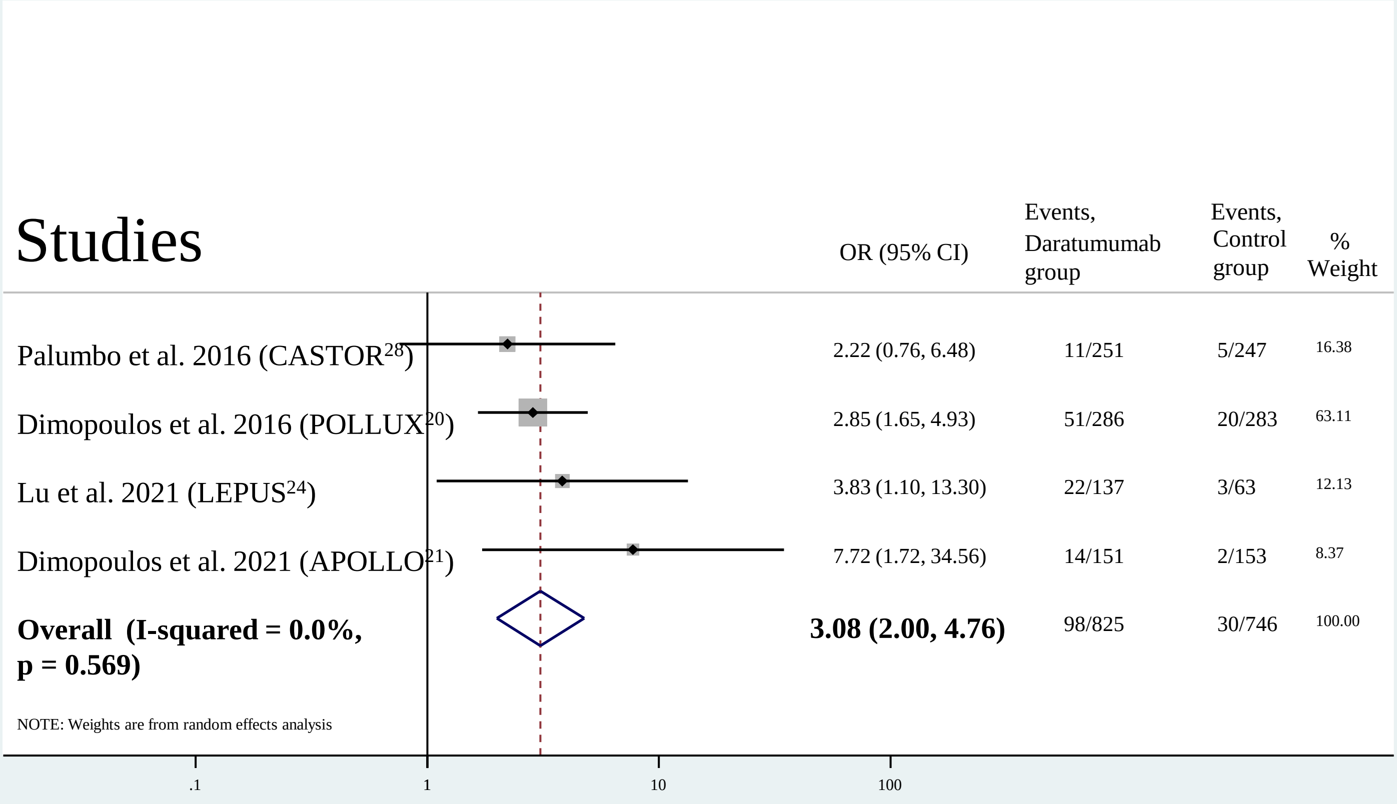

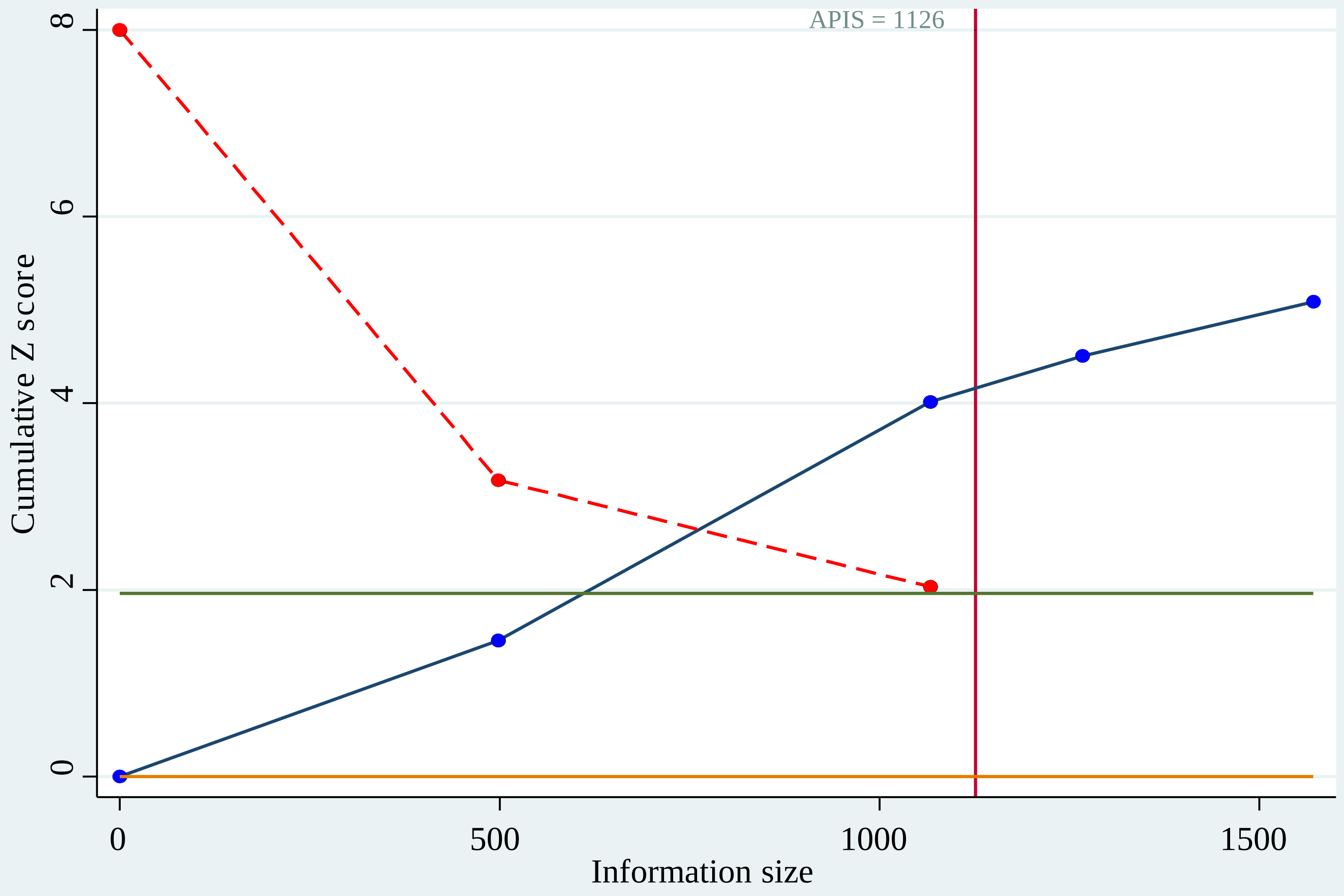

Supplementary Figure 38: Risk of bias assessment at study level and at domain level regarding stringent complete response in relapsed/refractory multiple myeloma
DVd, daratumumab, bortezomib, and dexamethasone; Vd, bortezomib and dexamethasone; KdD, carfilzomib, dexamethasone, and daratumumab; Kd, carfilzomib and dexamethasone; DRd, daratumumab, lenalidomide and dexamethasone; Rd, lenalidomide and dexamethasone

Study	Interventions	Randomization process	Deviations from intended interventions	Missing outcome data	Measurement of the outcome	Selection of the reported results	Overall assessment
Lu et al. 2021	DVd vs Vd	?	+	+	+	?	?
Dimopoulos et al. 2020	KdD vs Kd	+	+	+	+	+	+
Palumbo et al. 2016	DVd vs Vd	?	+	+	+	?	?
Dimopoulos et al. 2016	DRd vs Rd	+	+	+	+	?	+

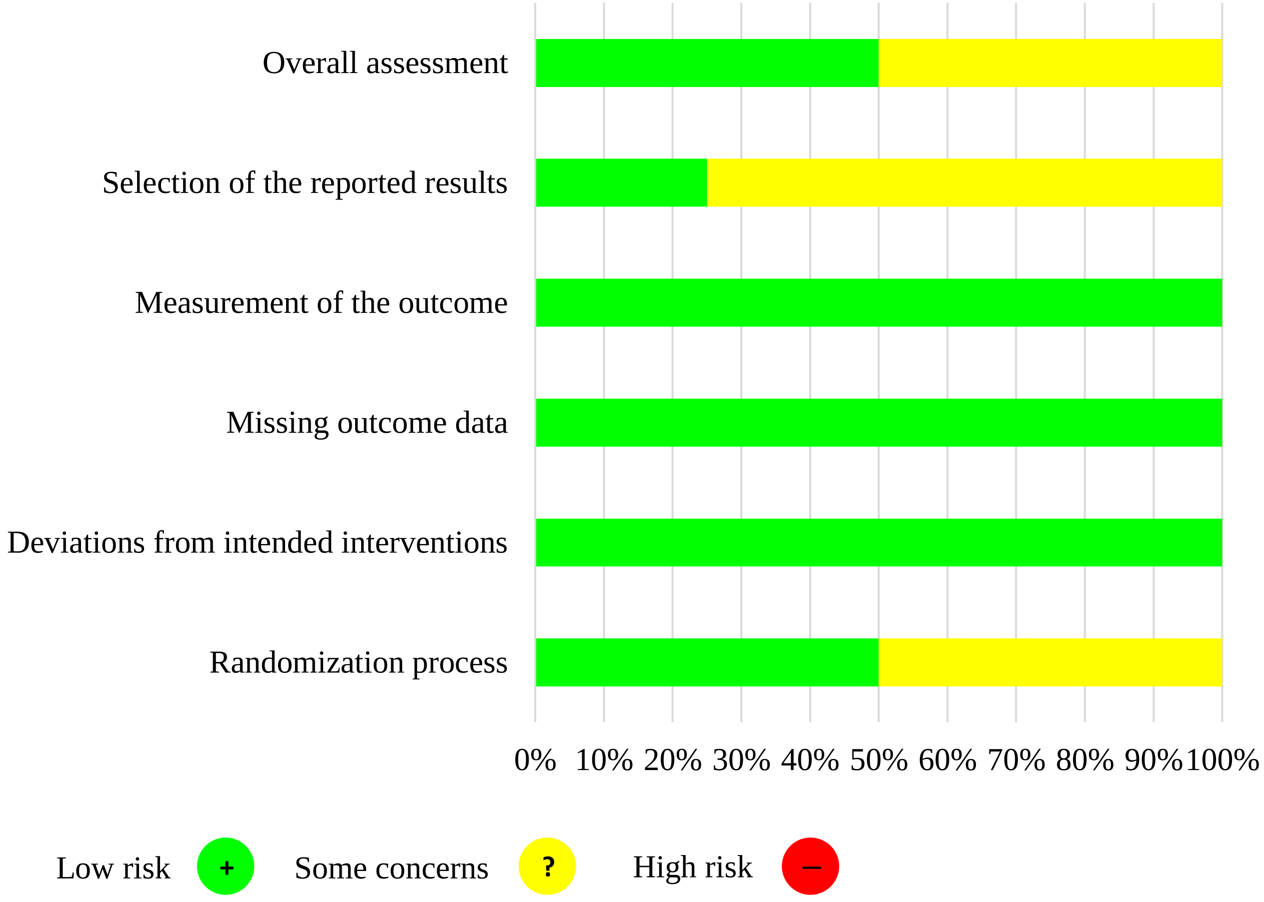

Supplementary Figure 39: Forest plot and Trial Sequential Analysis representing that addition of daratumumab to backbone therapies increases the chance for achieving complete response or better in relapsed/refractory multiple myeloma

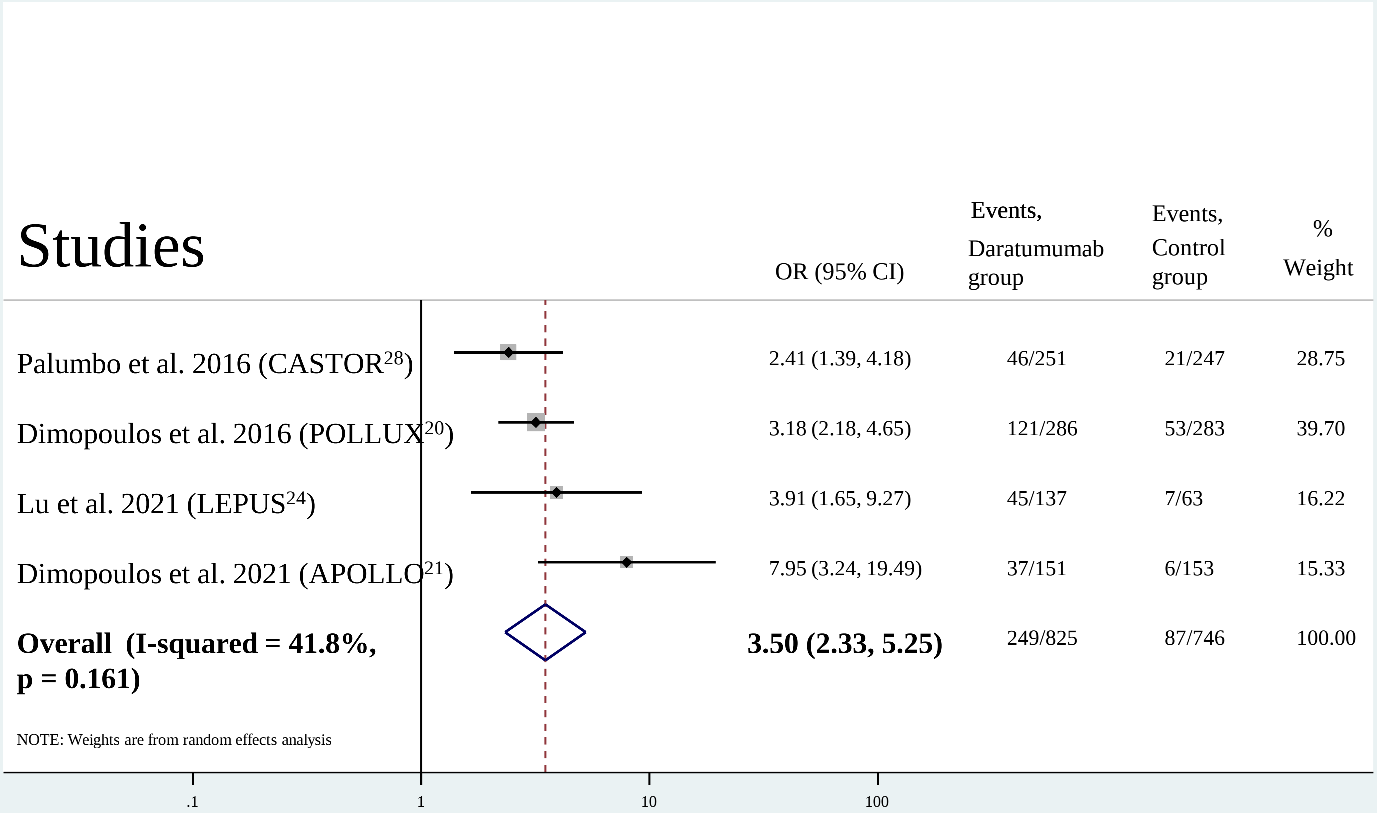

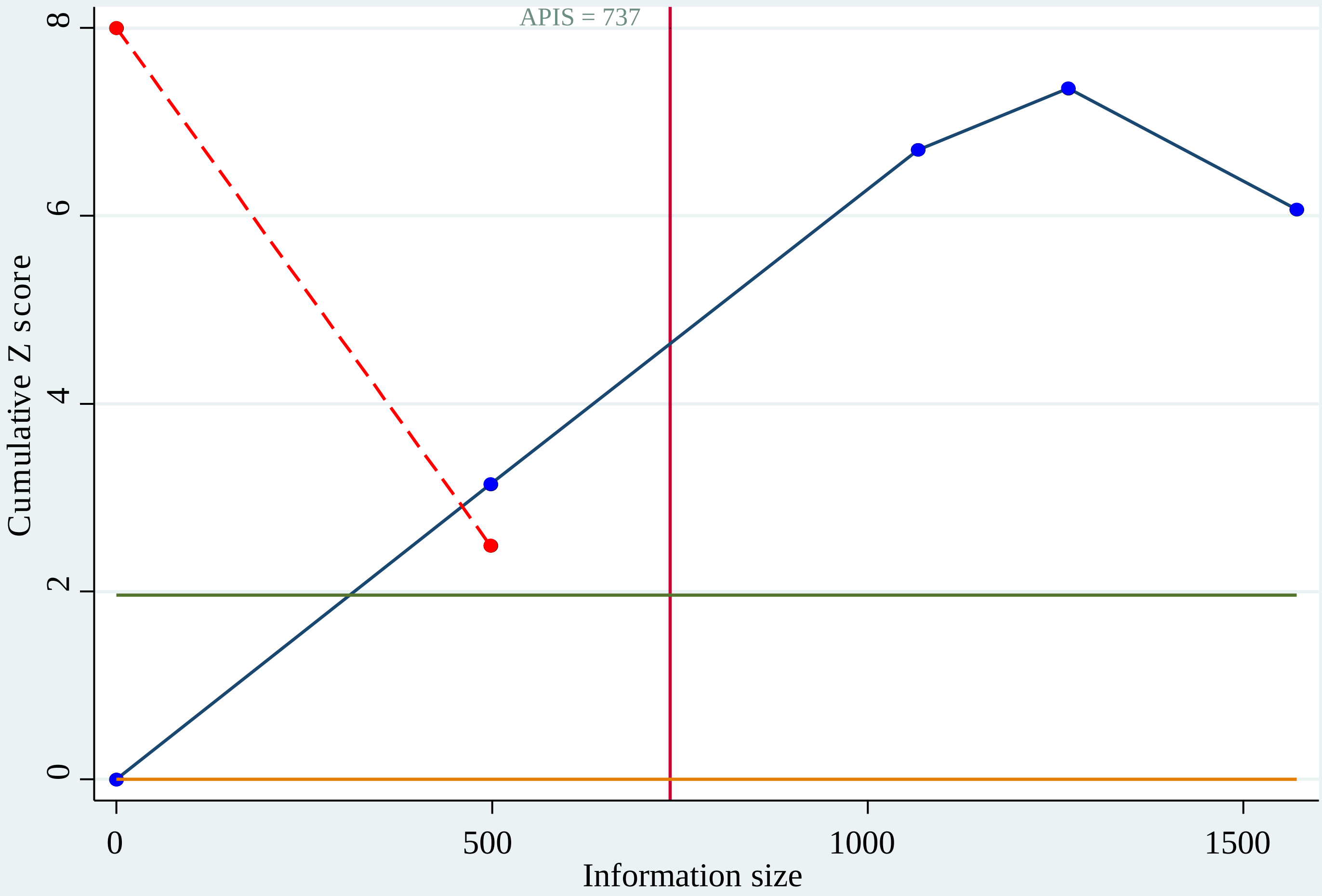

Supplementary Figure 40: Risk of bias assessment at study level and at domain level regarding complete response or better in relapsed/refractory multiple myeloma
DPd, daratumumab, pomalidomide, and dexamethasone; Pd, pomalidomide and dexamethasone; DVd, daratumumab, bortezomib, and dexamethasone; Vd, bortezomib and dexamethasone; DRd, daratumumab, lenalidomide and dexamethasone; Rd, lenalidomide and dexamethasone

Study	Interventions	Randomization process	Deviations from intended interventions	Missing outcome data	Measurement of the outcome	Selection of the reported results	Overall assessment
Dimopoulos et al. 2021	DPd vs Pd	+	+	+	+	?	+
Lu et al. 2021	DVd vs Vd	?	+	+	+	?	?
Palumbo et al. 2016	DVd vs Vd	?	+	+	+	?	?
Dimopoulos et al. 2016	DRd vs Rd	+	+	+	+	?	+

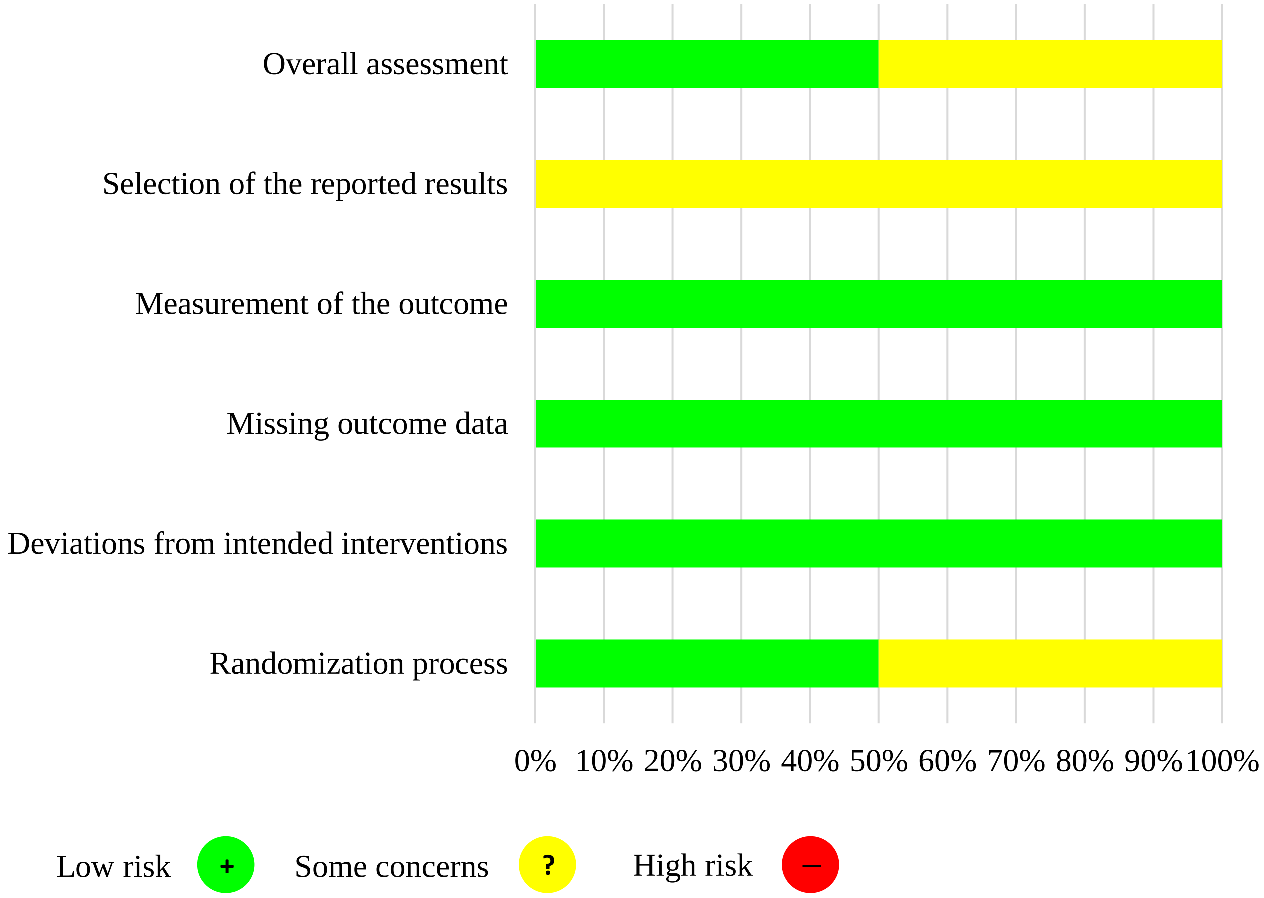

Supplementary Figure 41: Forest plot and Trial Sequential Analysis representing that addition of daratumumab to backbone therapies decreases the chance for death or disease progression in relapsed/refractory multiple myeloma

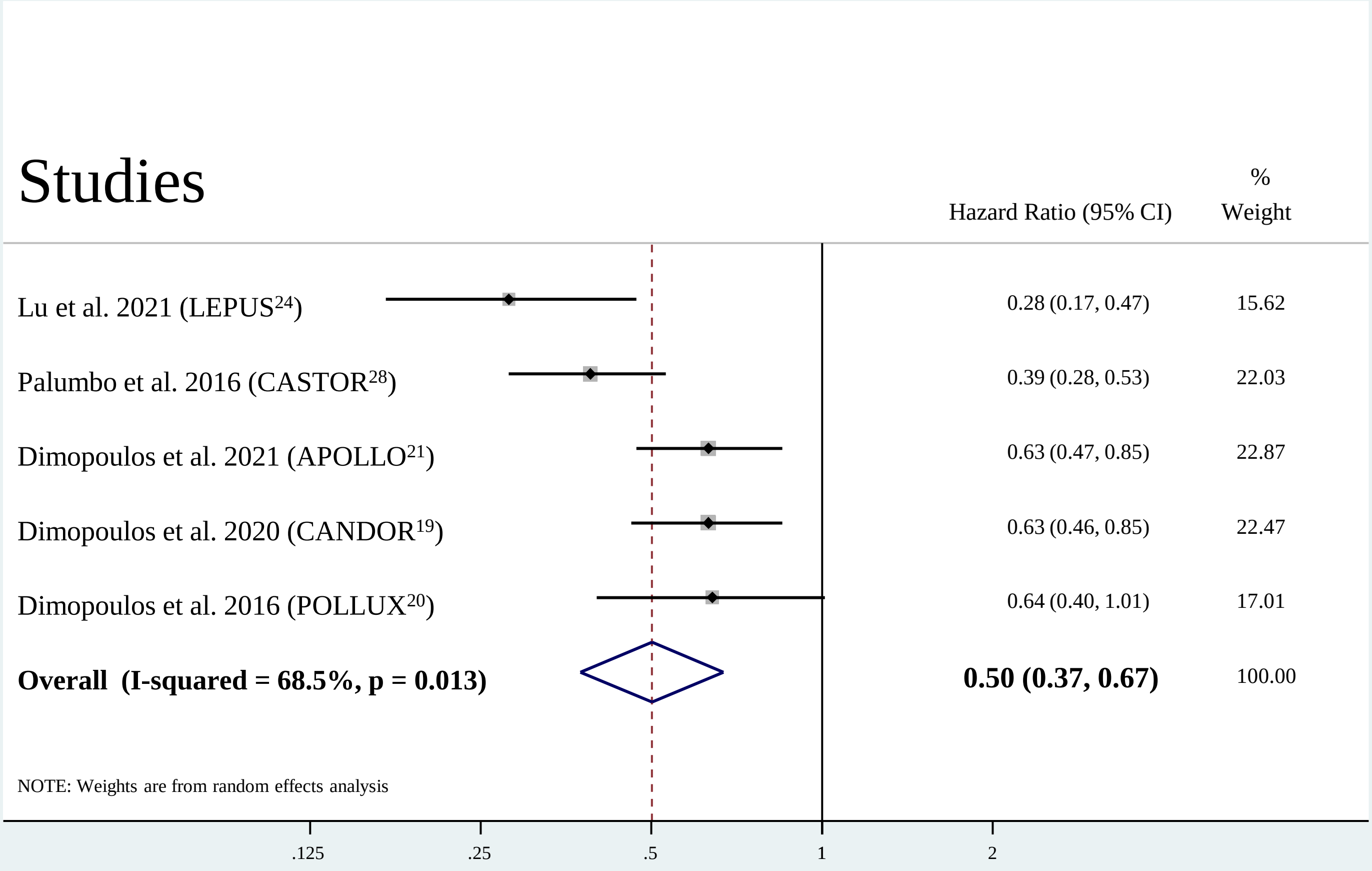

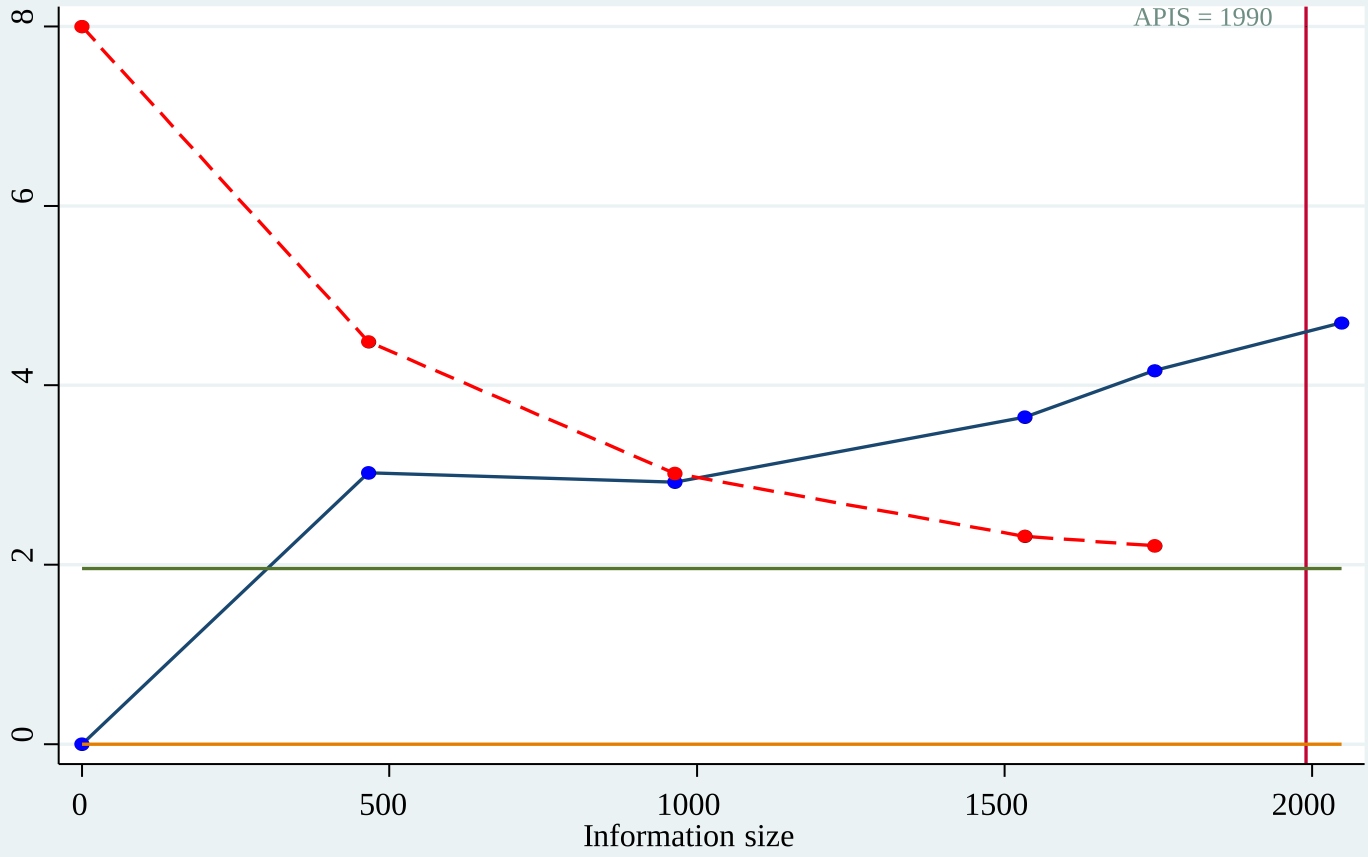

Supplementary Figure 42: Risk of bias assessment at study level and at domain level regarding death or disease progression in relapsed/refractory multiple myeloma
DPd, daratumumab, pomalidomide, and dexamethasone; Pd, pomalidomide and dexamethasone; DVd, daratumumab, bortezomib, and dexamethasone; Vd, bortezomib and dexamethasone; KdD, carfilzomib, dexamethasone, and daratumumab; Kd, carfilzomib and dexamethasone; DRd, daratumumab, lenalidomide and dexamethasone; Rd, lenalidomide and dexamethasone

**Study**	**Interventions**	**Randomization process**	**Deviations from intended interventions**	**Missing outcome data**	**Measurement of the outcome**	**Selection of the reported results**	**Overall assessment**
Dimopoulos et al. 2021	DPd vs Pd	+	+	+	+	?	+
Lu et al. 2021	DVd vs Vd	?	+	+	+	?	?
Dimopoulos et al. 2020	KdD vs Kd	+	+	+	+	+	+
Palumbo et al. 2016	DVd vs Vd	?	+	+	+	?	?
Dimopoulos et al. 2016	DRd vs Rd	+	+	+	+	?	+

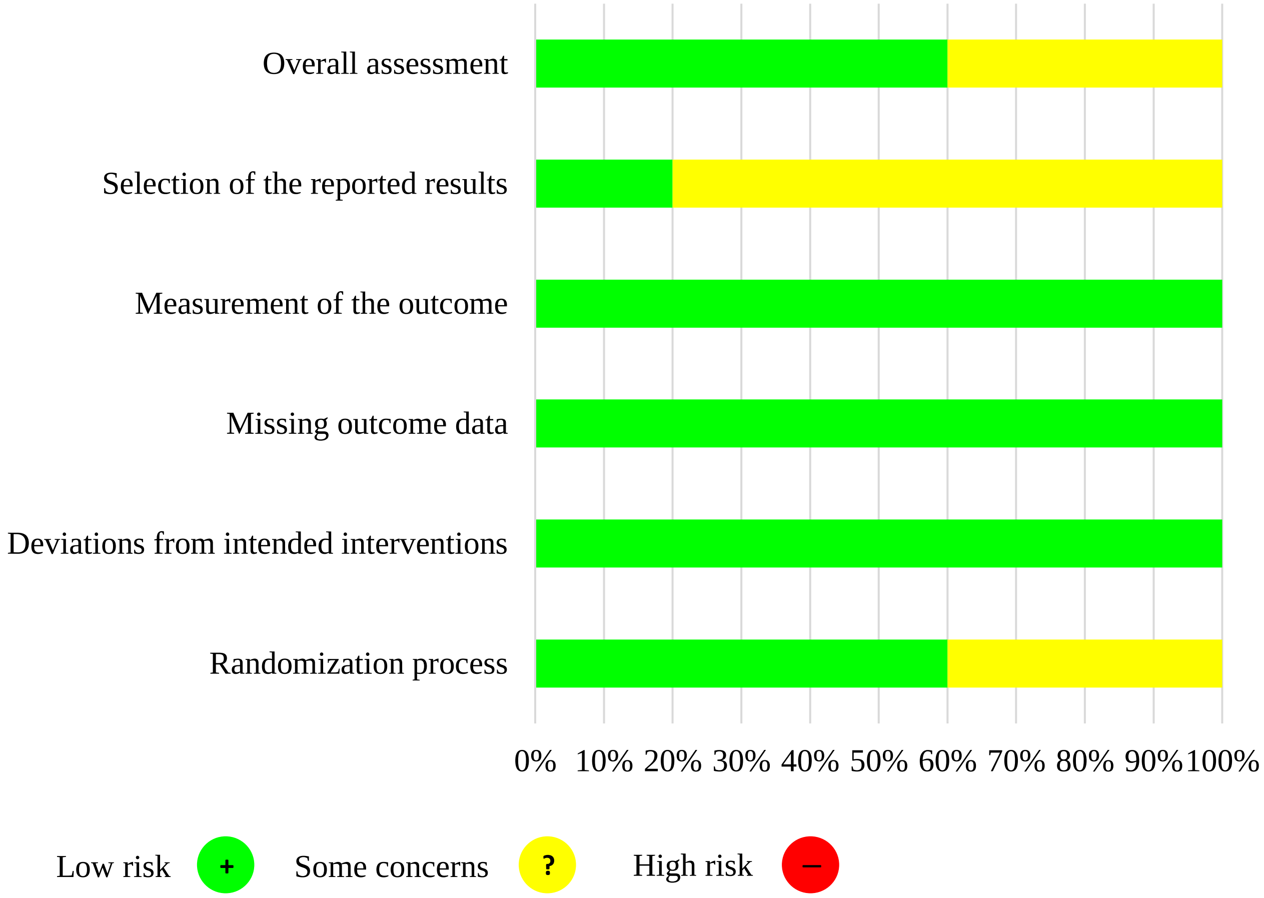

Supplementary Figure 43: Forest plot and Trial Sequential Analysis representing that addition of daratumumab to backbone therapies decreases the chance for death or disease progression in high cytogenetic risk relapsed/refractory multiple myeloma

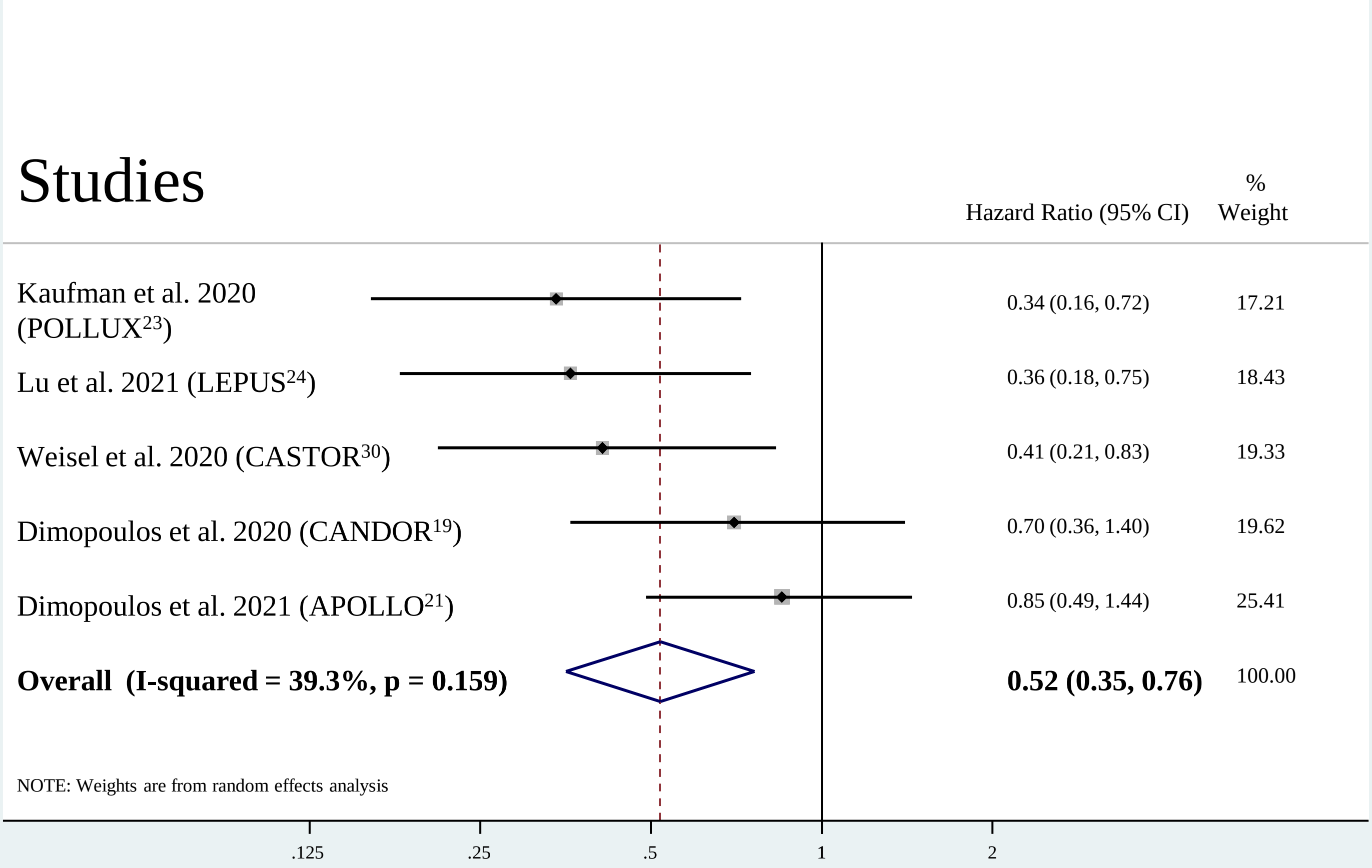

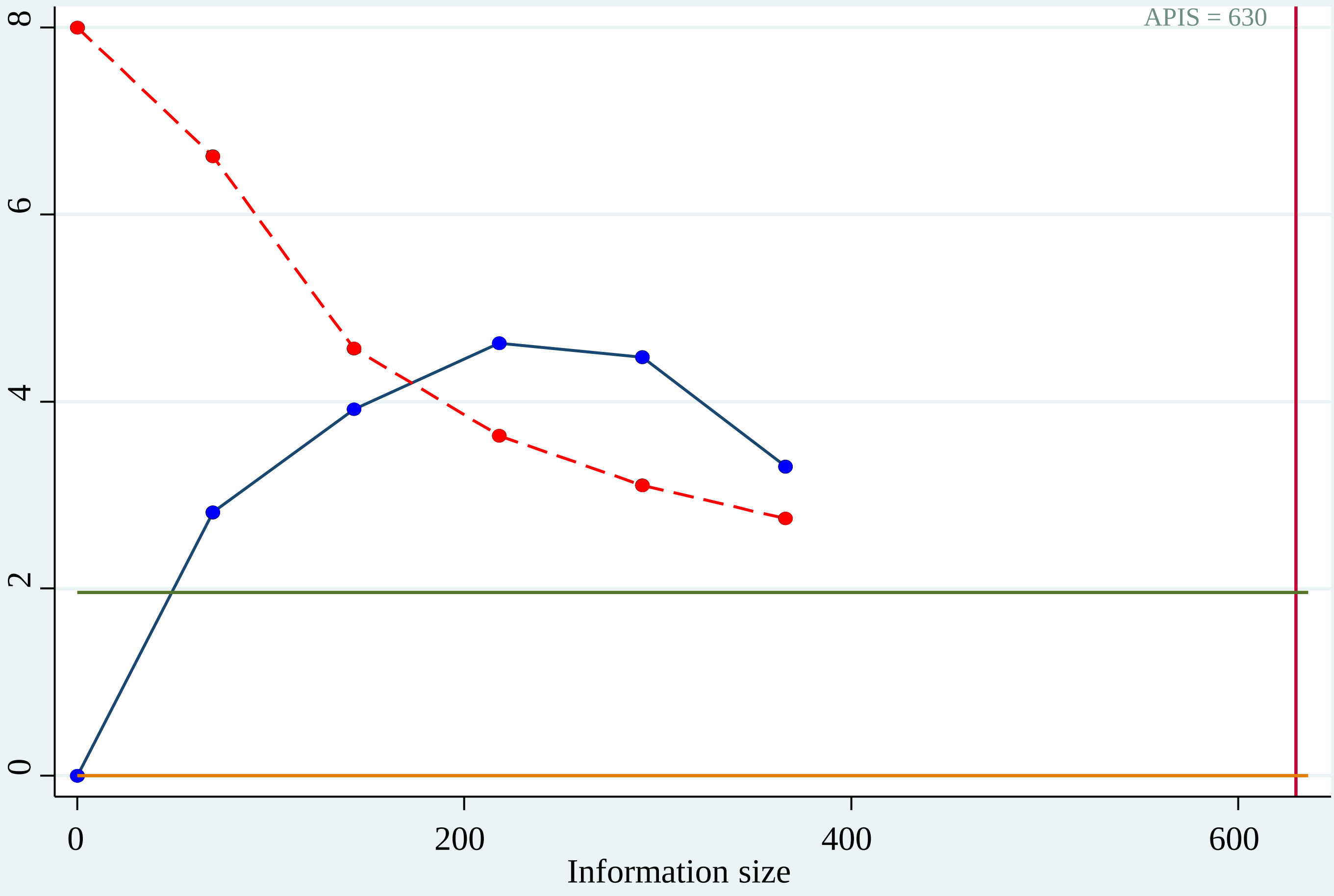

Supplementary Figure 44: Risk of bias assessment at study level and at domain level regarding death or disease progression in high cytogenetic risk relapsed/refractory multiple myeloma
DPd, daratumumab, pomalidomide, and dexamethasone; Pd, pomalidomide and dexamethasone; DVd, daratumumab, bortezomib, and dexamethasone; Vd, bortezomib and dexamethasone; KdD, carfilzomib, dexamethasone, and daratumumab; Kd, carfilzomib and dexamethasone; DRd, daratumumab, lenalidomide and dexamethasone; Rd, lenalidomide and dexamethasone

Study	Interventions	Randomization process	Deviations from intended interventions	Missing outcome data	Measurement of the outcome	Selection of the reported results	Overall assessment
Dimopoulos et al. 2021	DPd vs Pd	+	+	+	+	?	+
Kaufman et al. 2021	DRd vs Rd	+	+	+	+	?	+
Lu et al. 2021	DVd vs Vd	?	+	+	+	?	?
Dimopoulos et al. 2020	KdD vs Kd	+	+	+	+	+	+
Weisel et al. 2020	DVd vs Vd	?	+	+	+	?	?

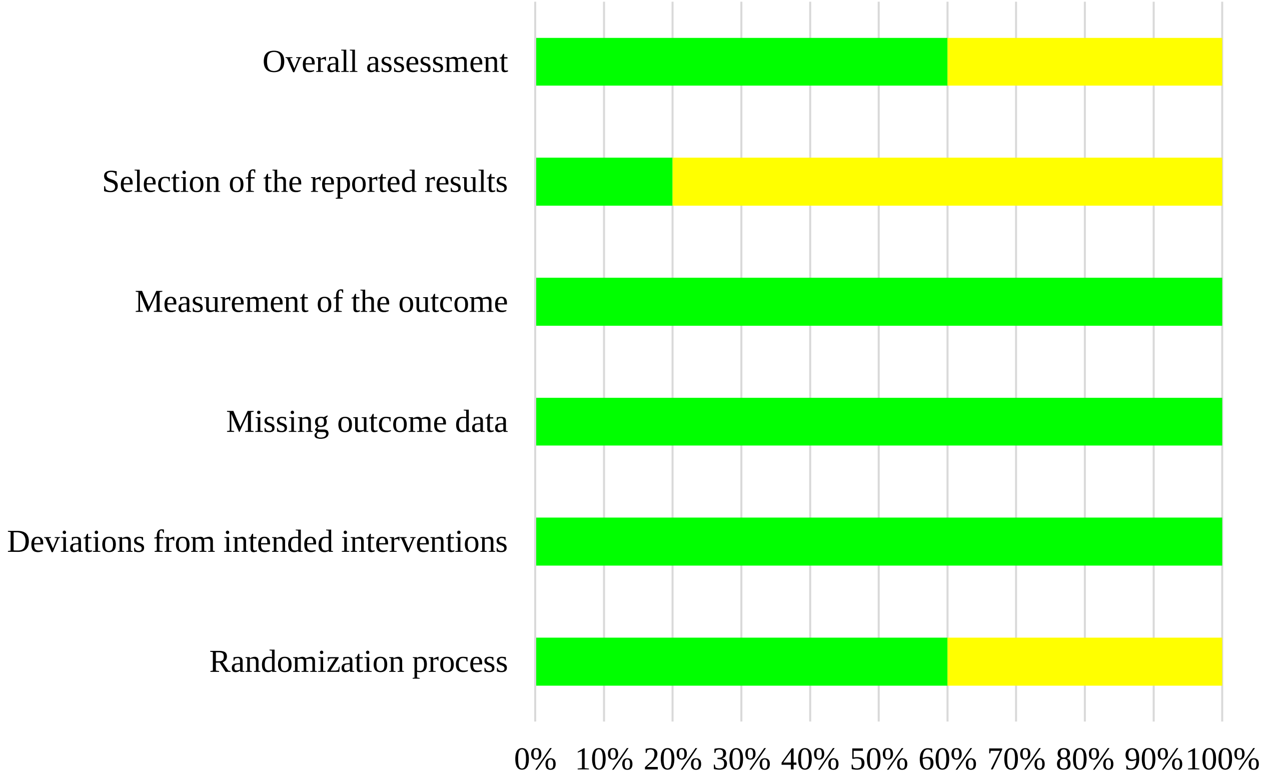

Low risk + Some concerns ? High risk –

Supplementary Figure 45: Forest plot and Trial Sequential Analysis representing that addition of daratumumab to backbone therapies decreases the chance for death or disease progression in standard cytogenetic risk relapsed/refractory multiple myeloma

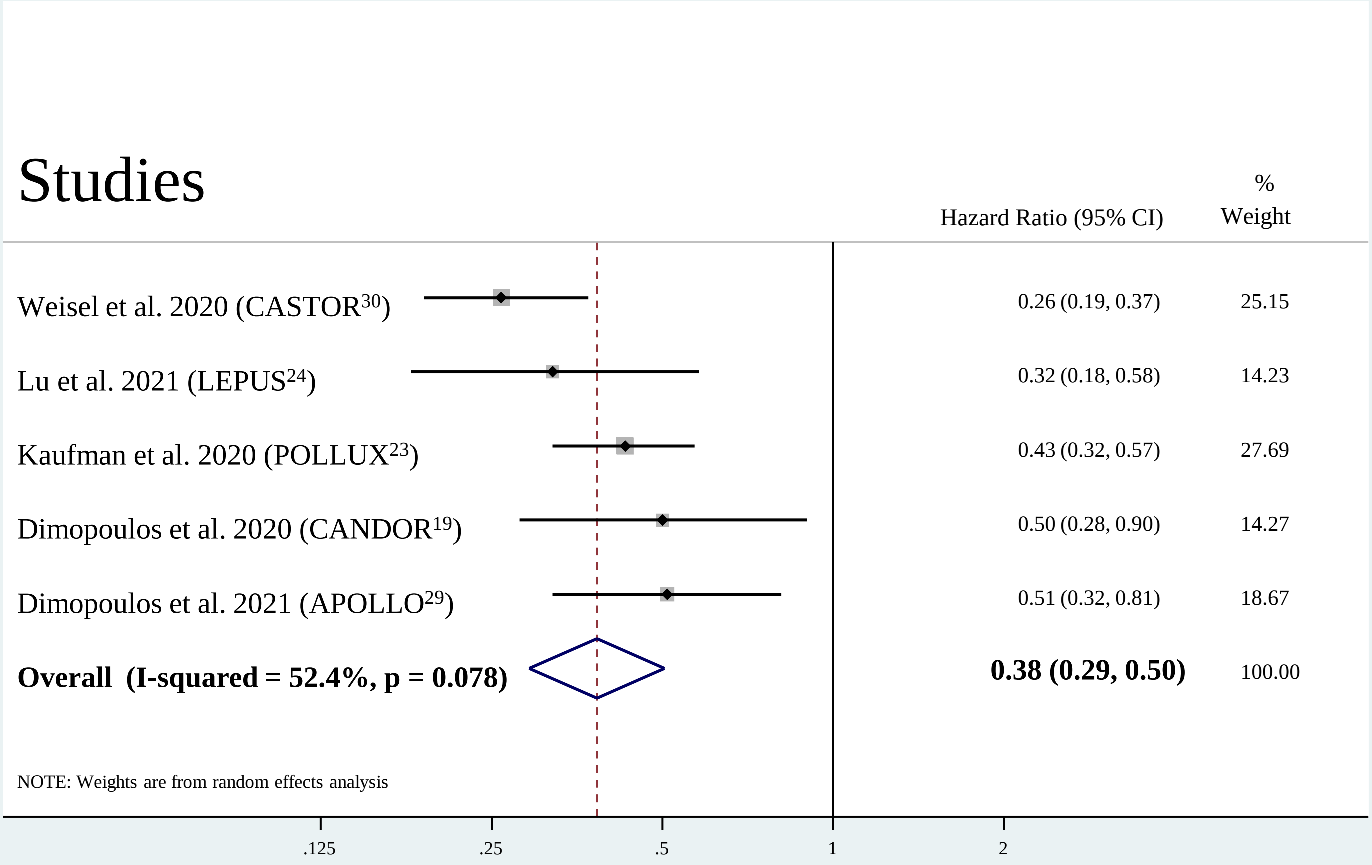

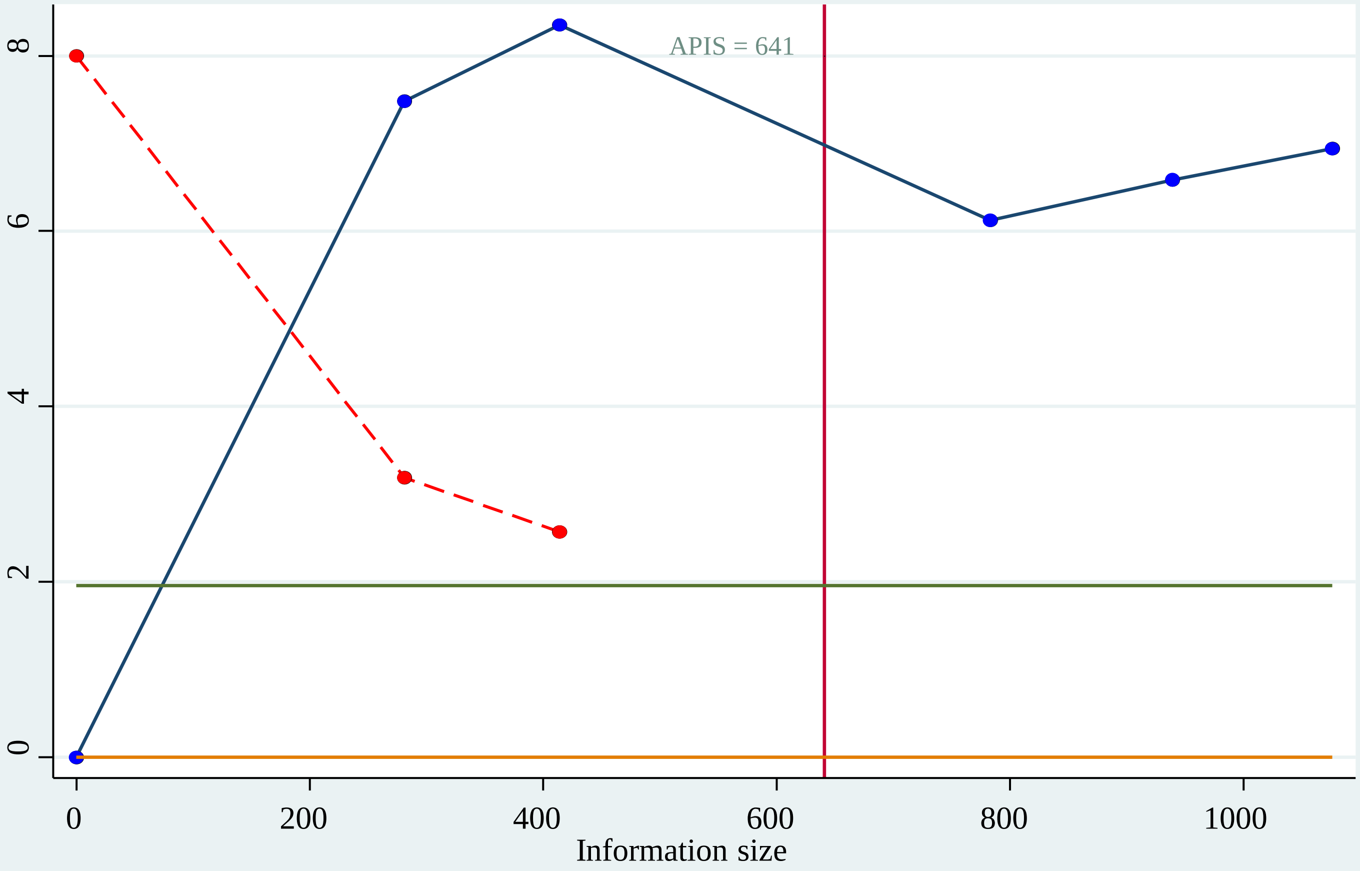

Supplementary Figure 46: Risk of bias assessment at study level and at domain level regarding death or disease progression in standard cytogenetic risk relapsed/refractory multiple myeloma
DPd, daratumumab, pomalidomide, and dexamethasone; Pd, pomalidomide and dexamethasone; DVd, daratumumab, bortezomib, and dexamethasone; Vd, bortezomib and dexamethasone; KdD, carfilzomib, dexamethasone, and daratumumab; Kd, carfilzomib and dexamethasone; DRd, daratumumab, lenalidomide and dexamethasone; Rd, lenalidomide and dexamethasone

Study	Interventions	Randomization process	Deviations from intended interventions	Missing outcome data	Measurement of the outcome	Selection of the reported results	Overall assessment
Dimopoulos et al. 2021	DPd vs Pd	+	+	+	+	?	+
Kaufman et al. 2021	DRd vs Rd	+	+	+	+	?	+
Lu et al. 2021	DVd vs Vd	?	+	+	+	?	?
Dimopoulos et al. 2020	KdD vs Kd	+	+	+	+	+	+
Weisel et al. 2020	DVd vs Vd	?	+	+	+	?	?

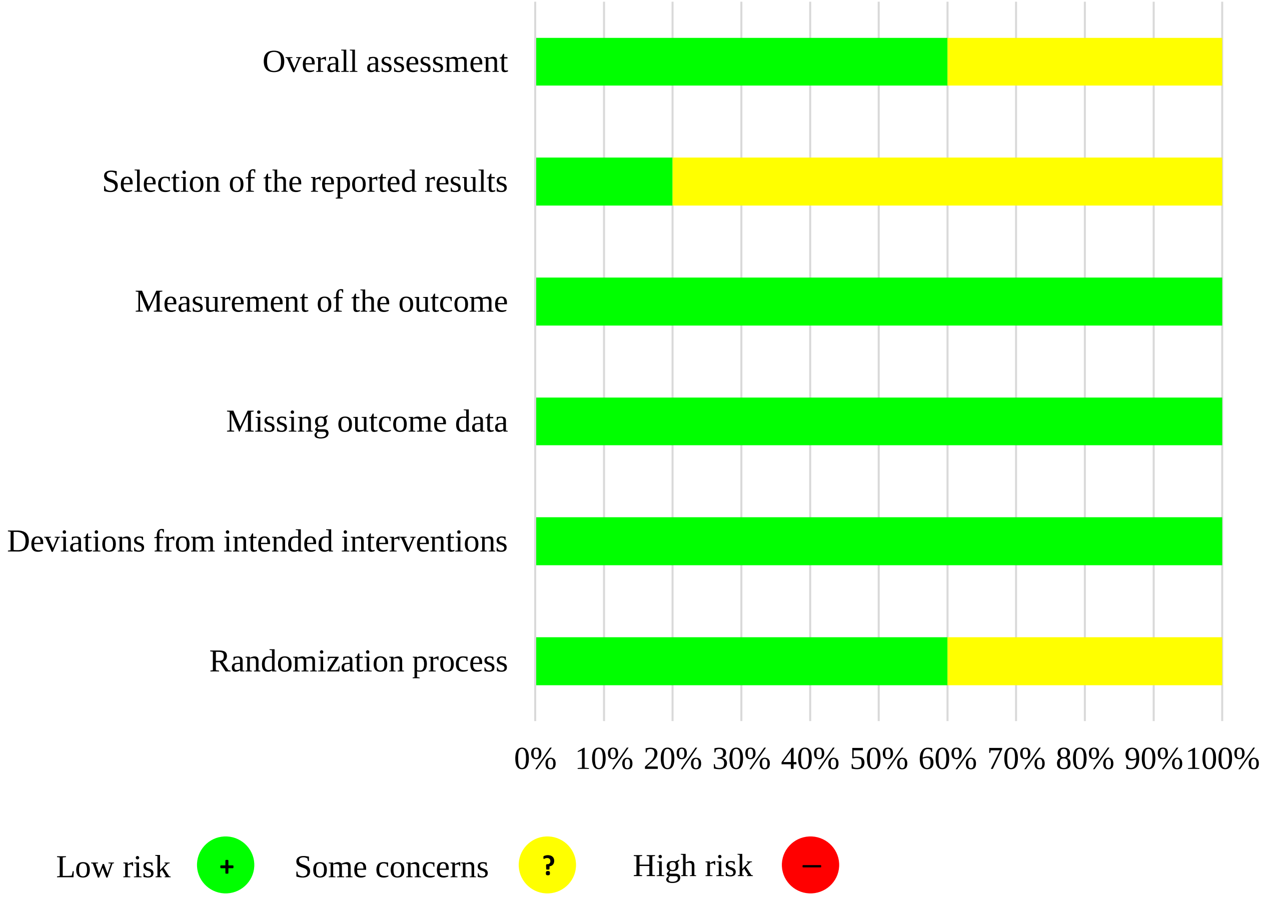

Supplementary Figure 47: Forest plot and Trial Sequential Analysis representing that addition of daratumumab to backbone therapies increases the chance for all grade thrombocytopenia in relapsed/refractory diagnosed multiple myeloma

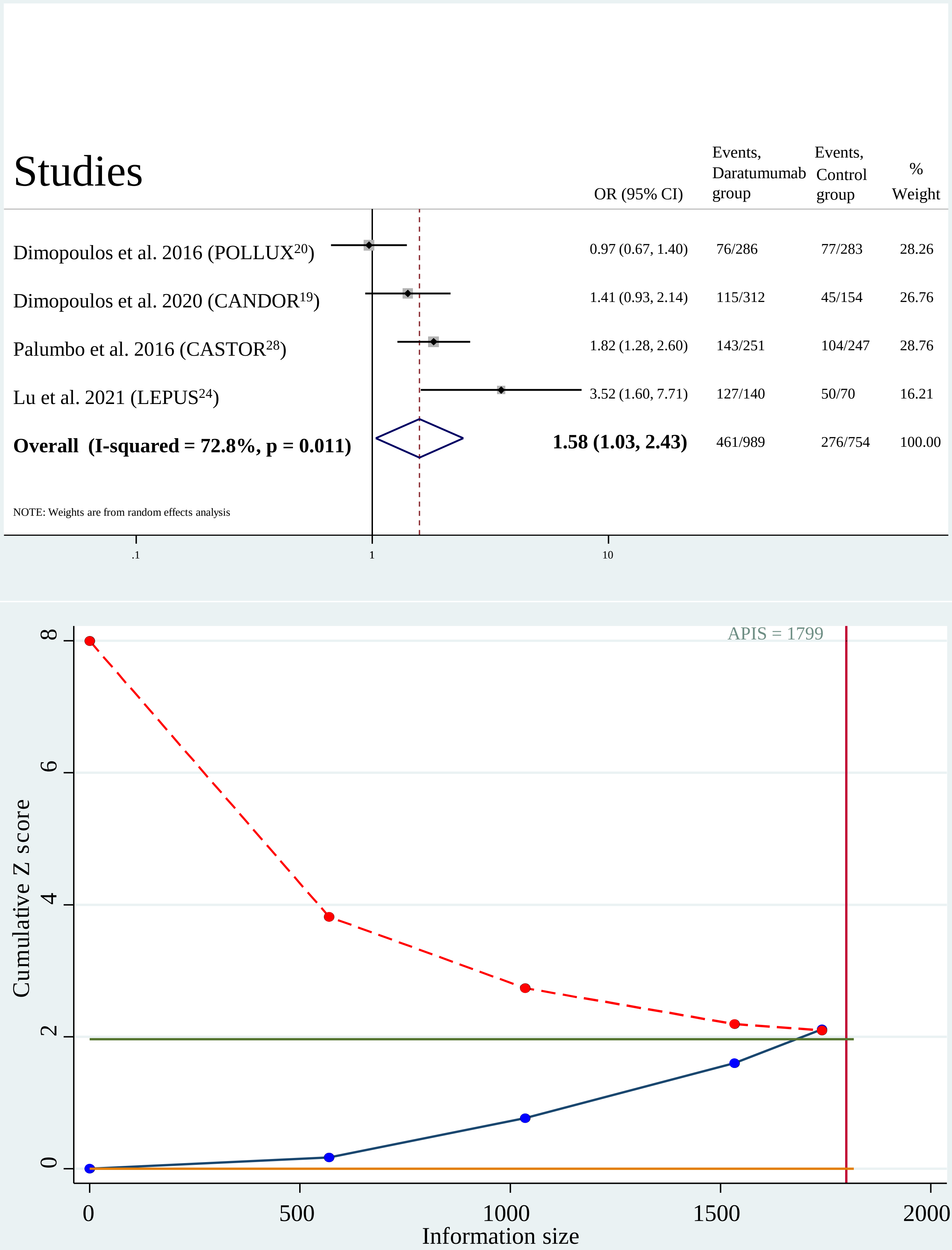

Supplementary Figure 48: Risk of bias assessment at study level and at domain level regarding all grade thrombocytopenia in relapsed/refractory multiple myeloma
KdD, carfilzomib, dexamethasone, and daratumumab; Kd, carfilzomib and dexamethasone; DVd, daratumumab, bortezomib, and dexamethasone; Vd, bortezomib and dexamethasone; DRd, daratumumab, lenalidomide and dexamethasone; Rd, lenalidomide and dexamethasone

**Study**	**Interventions**	**Randomization process**	**Deviations from intended interventions**	**Missing outcome data**	**Measurement of the outcome**	**Selection of the reported results**	**Overall assessment**
Lu et al. 2021	DVd vs Vd	?	+	+	+	?	?
Dimopoulos et al. 2020	KdD vs Kd	+	+	+	+	+	+
Palumbo et al. 2016	DVd vs Vd	?	+	+	+	?	?
Dimopoulos et al. 2016	DRd vs Rd	+	+	+	+	?	+

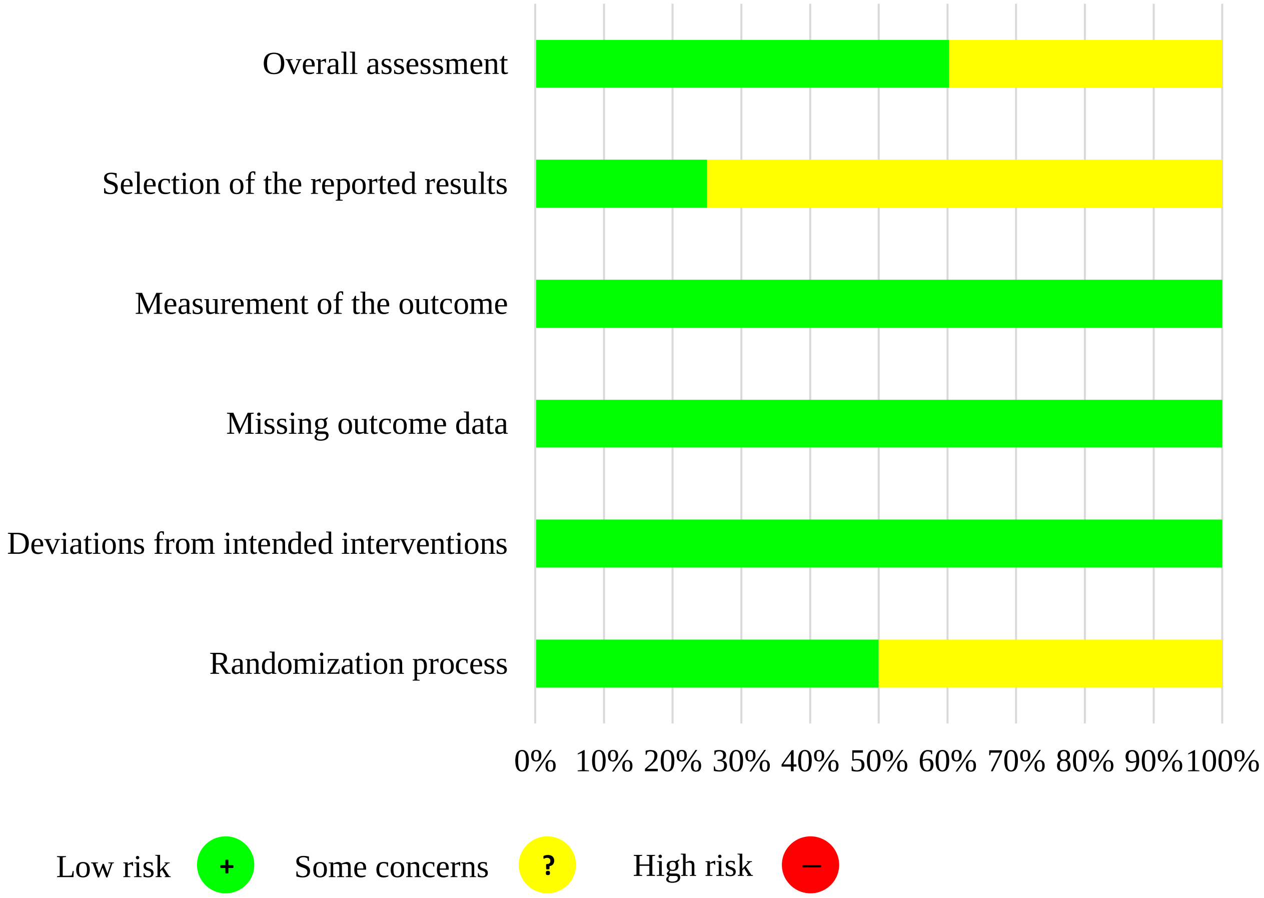

Supplementary Figure 49: Forest plot and Trial Sequential Analysis representing that addition of daratumumab to backbone therapies increases the chance for grade 3-4 thrombocytopenia in relapsed/refractory diagnosed multiple myeloma

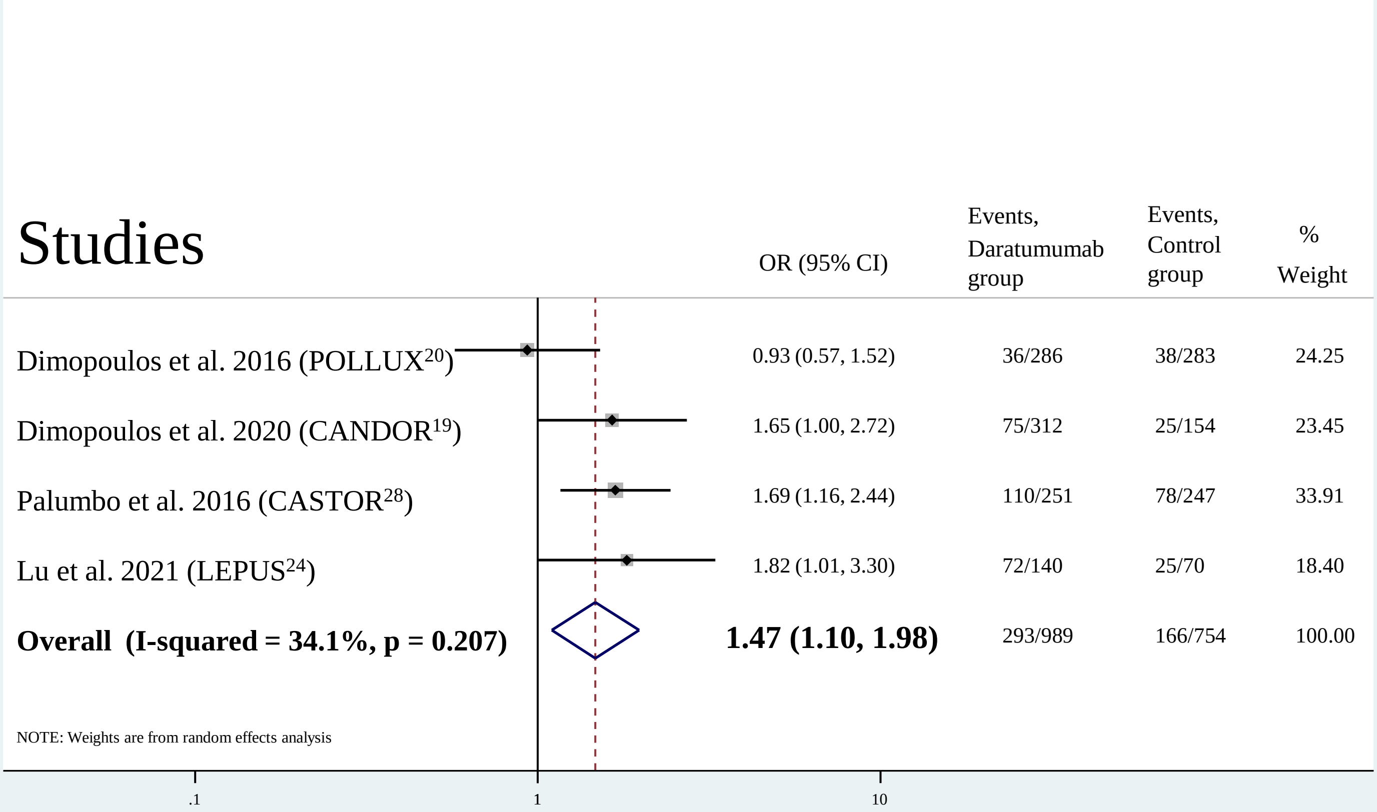

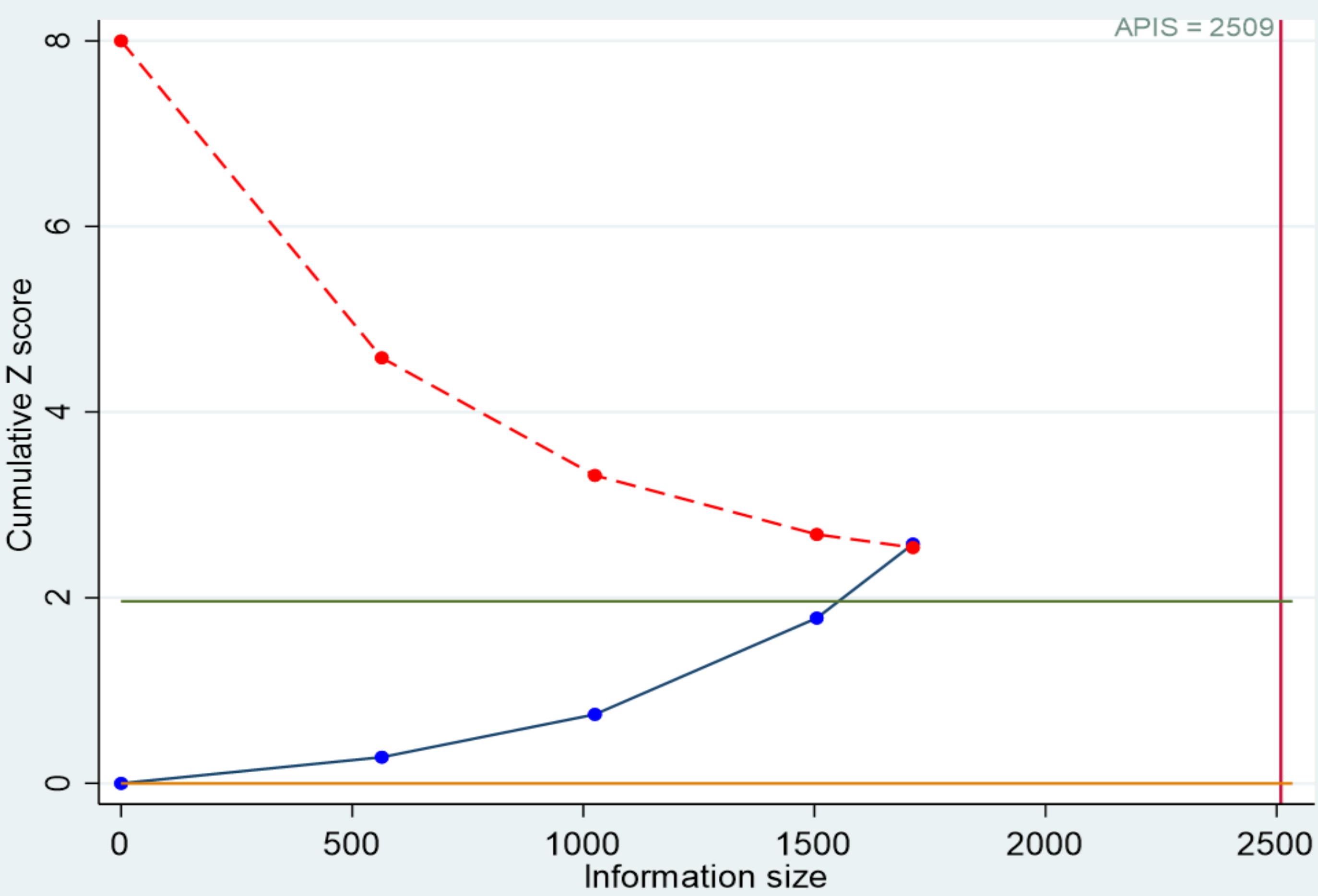

Supplementary Figure 50: Risk of bias assessment at study level and at domain level regarding grade 3-4 thrombocytopenia in relapsed/refractory multiple myeloma

KdD, carfilzomib, dexamethasone, and daratumumab; Kd, carfilzomib and dexamethasone; DVd, daratumumab, bortezomib, and dexamethasone; Vd, bortezomib and dexamethasone; DRd, daratumumab, lenalidomide and dexamethasone; Rd, lenalidomide and dexamethasone

Study	Interventions	Randomization process	Deviations from intended interventions	Missing outcome data	Measurement of the outcome	Selection of the reported results	Overall assessment
Lu et al. 2021	DVd vs Vd	?	+	+	+	?	?
Dimopoulos et al. 2020	KdD vs Kd	+	+	+	+	+	
Palumbo et al. 2016	DVd vs Vd	?	+	+	+	?	?
Dimopoulos et al. 2016	DRd vs Rd	+	+	+	+	?	+

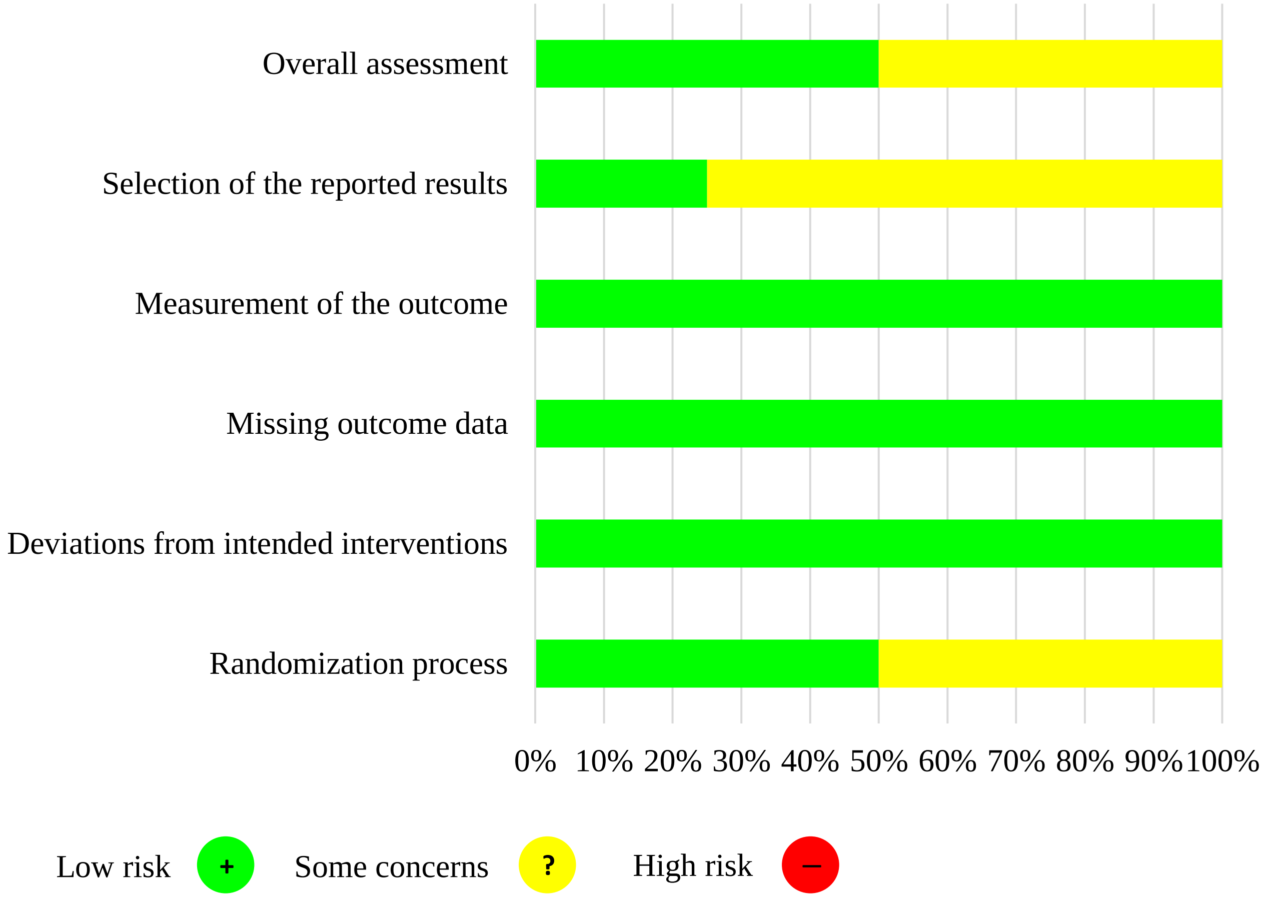

Supplementary Figure 51: Forest plot and Trial Sequential Analysis representing that addition of daratumumab to backbone therapies does not increase the chance for all grade lymphopenia in relapsed/refractory multiple myeloma

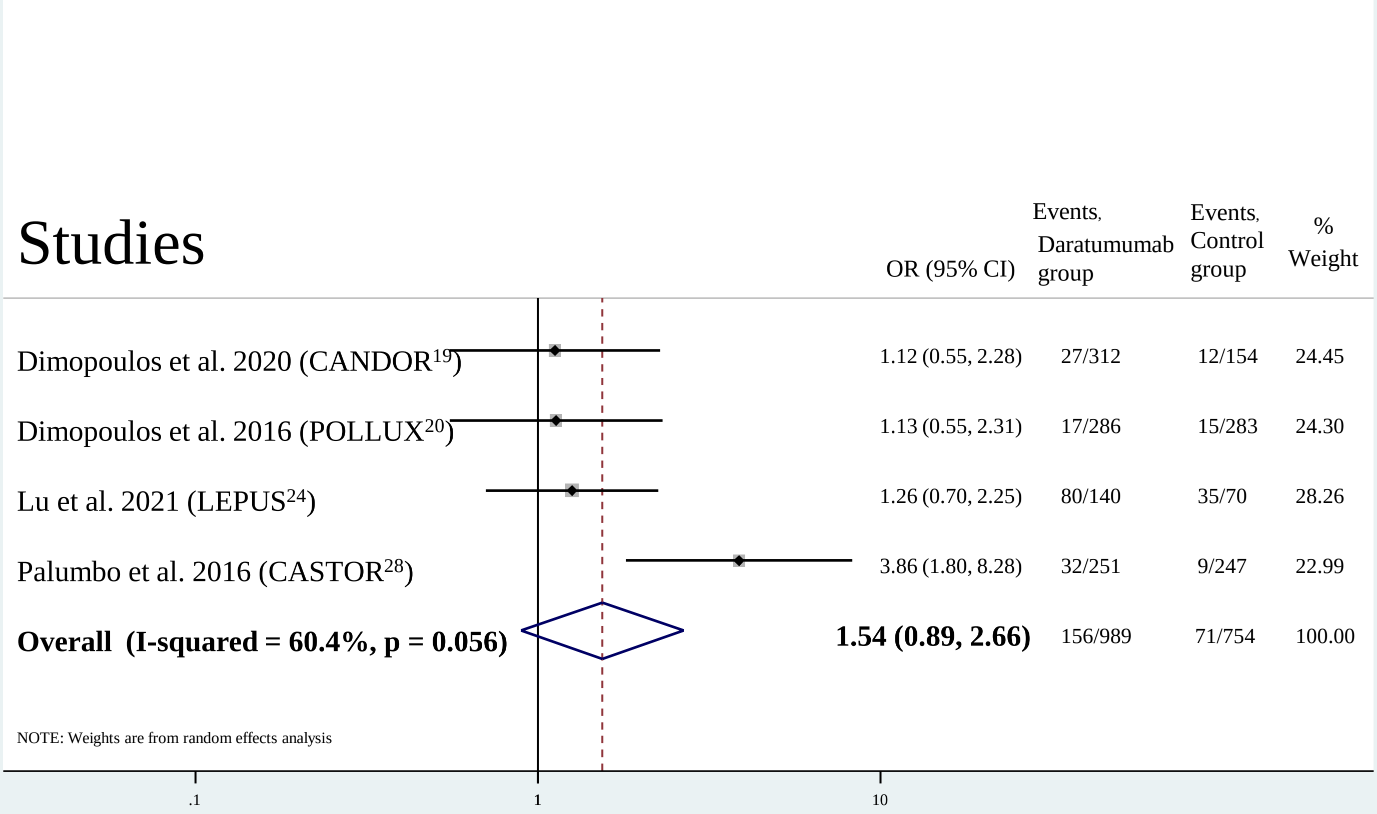

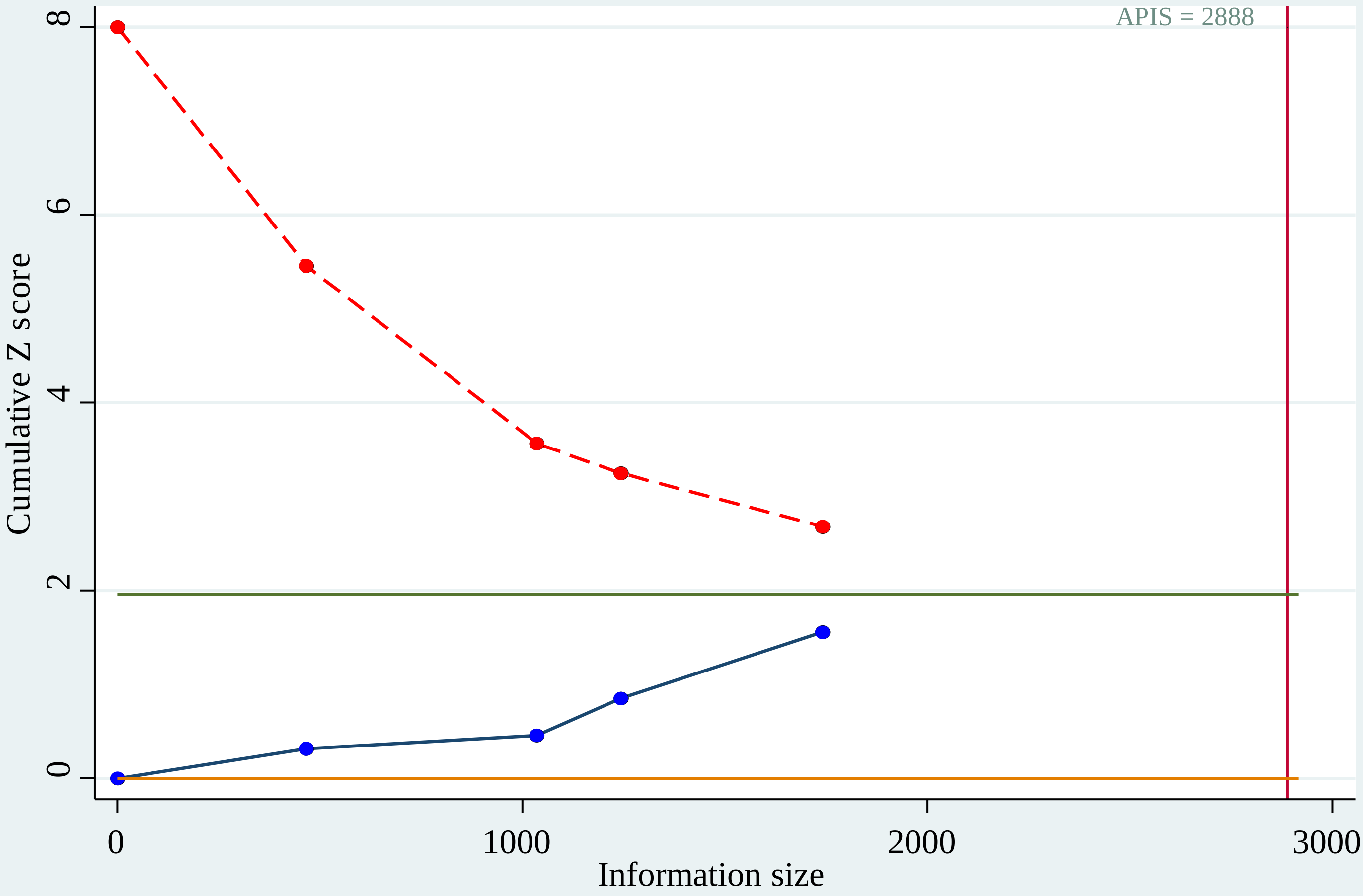

Supplementary Figure 52: Risk of bias assessment at study level and at domain level regarding all grade lymphopenia in relapsed/refractory multiple myeloma
KdD, carfilzomib, dexamethasone, and daratumumab; Kd, carfilzomib and dexamethasone; DVd, daratumumab, bortezomib, and dexamethasone; Vd, bortezomib and dexamethasone; DRd, daratumumab, lenalidomide and dexamethasone; Rd, lenalidomide and dexamethasone

Study	Interventions	Randomization process	Deviations from intended interventions	Missing outcome data	Measurement of the outcome	Selection of the reported results	Overall assessment
Lu et al. 2021	DVd vs Vd	?	+	+	+	?	?
Dimopoulos et al. 2020	KdD vs Kd	+	+	+	+	+	+
Palumbo et al. 2016	DVd vs Vd	?	+	+	+	?	?
Dimopoulos et al. 2016	DRd vs Rd	+	+	+	+	?	+

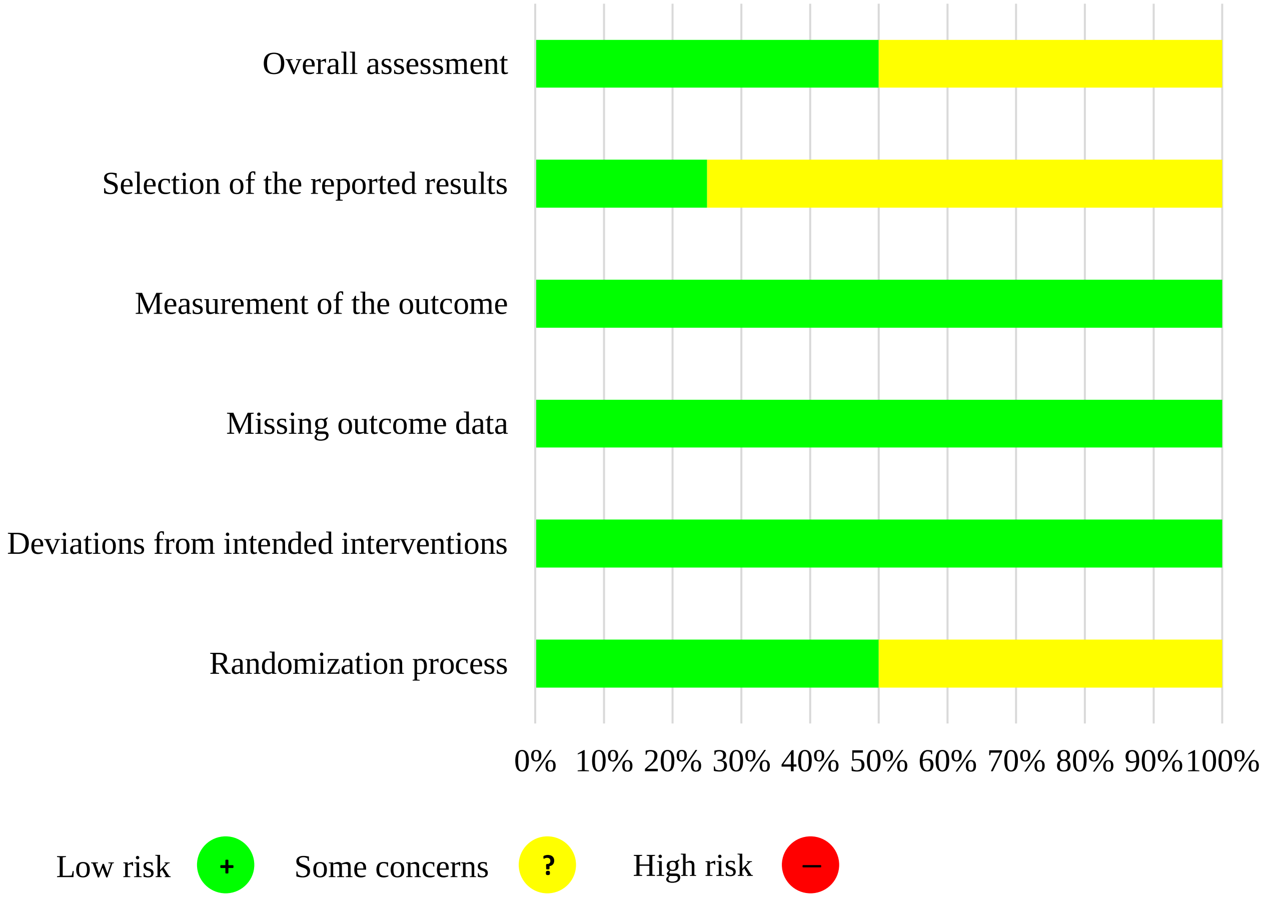

Supplementary Figure 53: Forest plot and Trial Sequential Analysis representing that addition of daratumumab to backbone therapies does not increase the chance for grade 3-4 lymphopenia in relapsed/refractory multiple myeloma

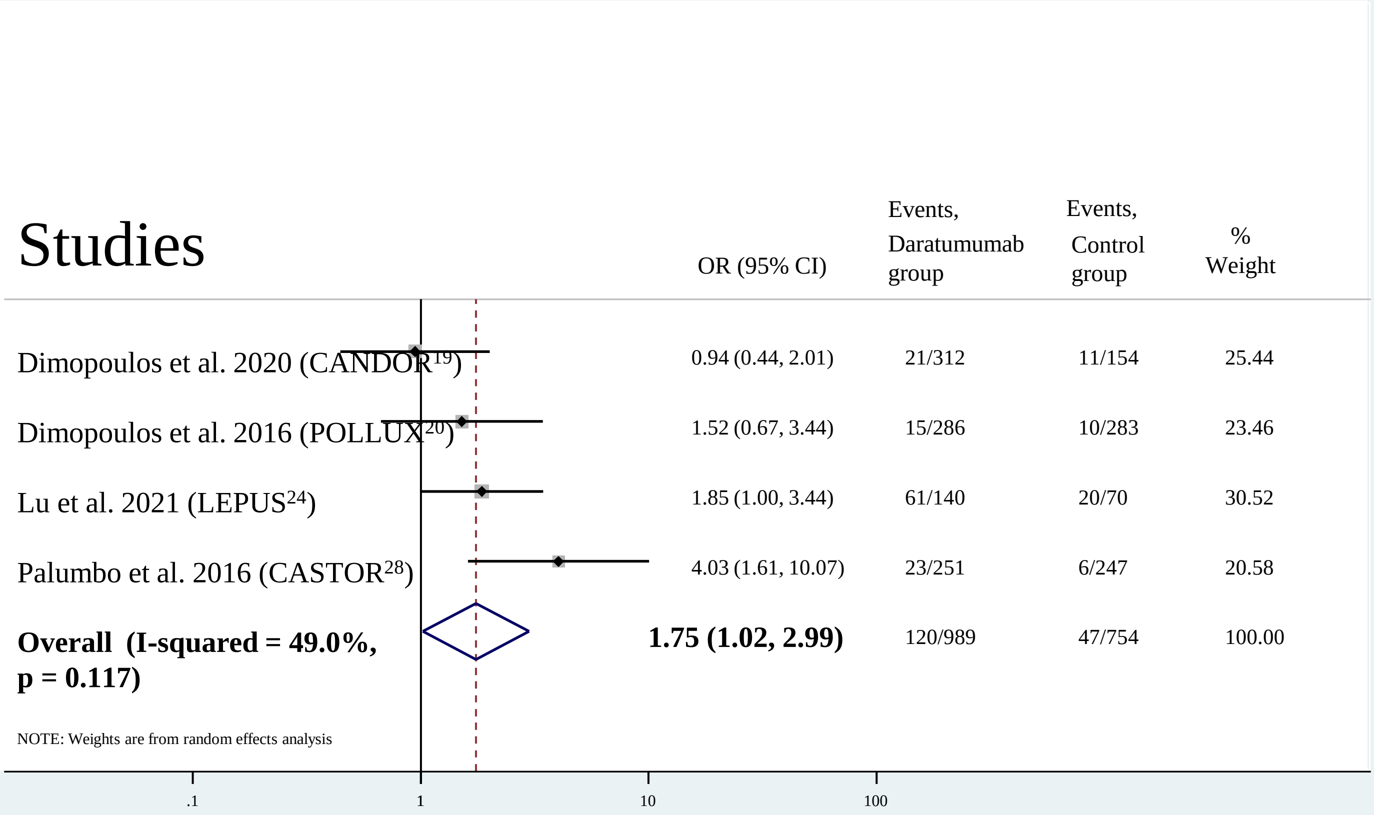

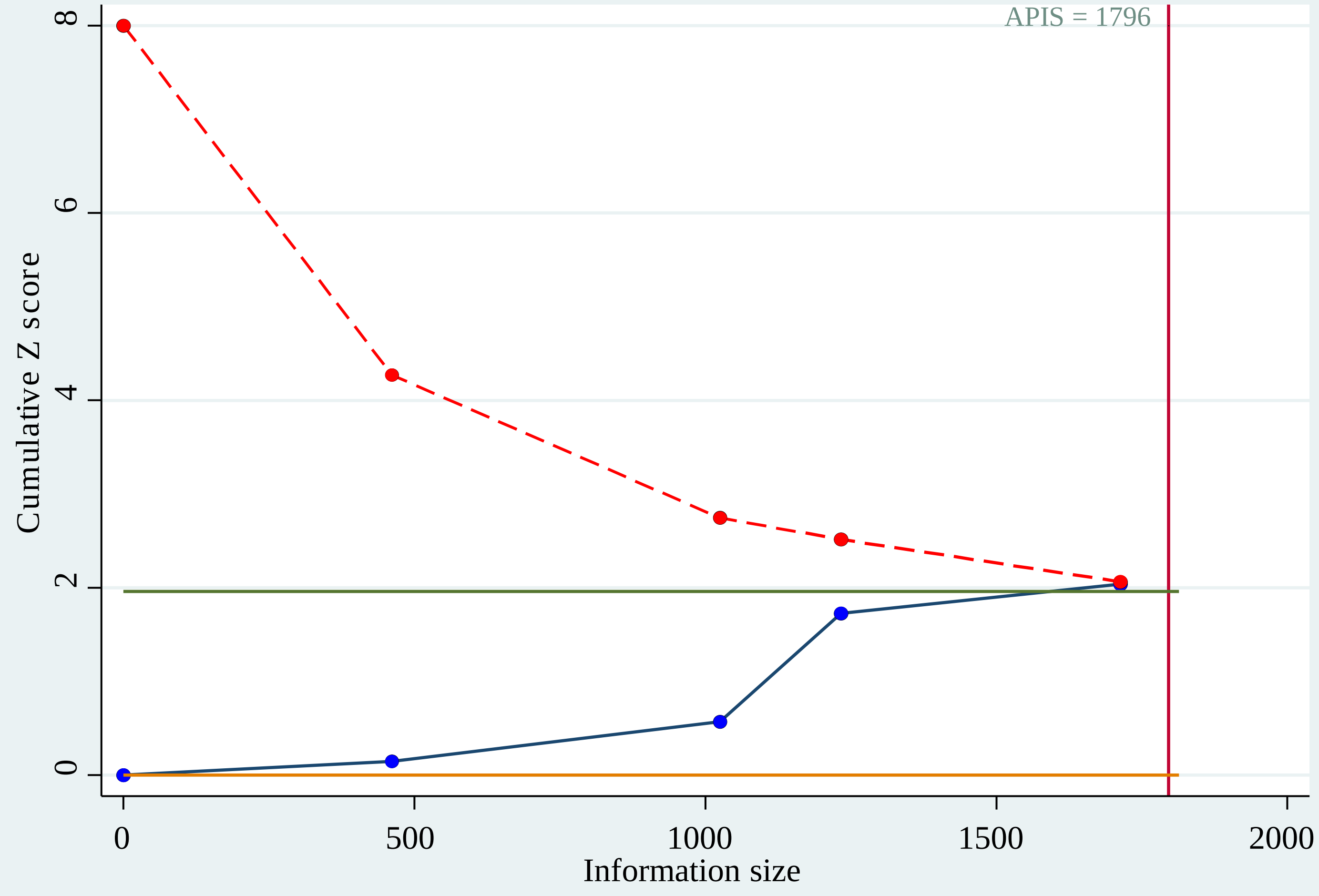

Supplementary Figure 54: Risk of bias assessment at study level and at domain level regarding grade 3-4 lymphopenia in relapsed/refractory multiple myeloma

KdD, carfilzomib, dexamethasone, and daratumumab; Kd, carfilzomib and dexamethasone; DVd, daratumumab, bortezomib, and dexamethasone; Vd, bortezomib and dexamethasone; DRd, daratumumab, lenalidomide and dexamethasone; Rd, lenalidomide and dexamethasone

Study	Interventions	Randomization process	Deviations from intended interventions	Missing outcome data	Measurement of the outcome	Selection of the reported results	Overall assessment
Lu et al. 2021	DVd vs Vd	?	+	+	+	?	?
Dimopoulos et al. 2020	KdD vs Kd	+	+	+	+	+	+
Palumbo et al. 2016	DVd vs Vd	?	+	+	+	?	?
Dimopoulos et al. 2016	DRd vs Rd	+	+	+	+	?	+

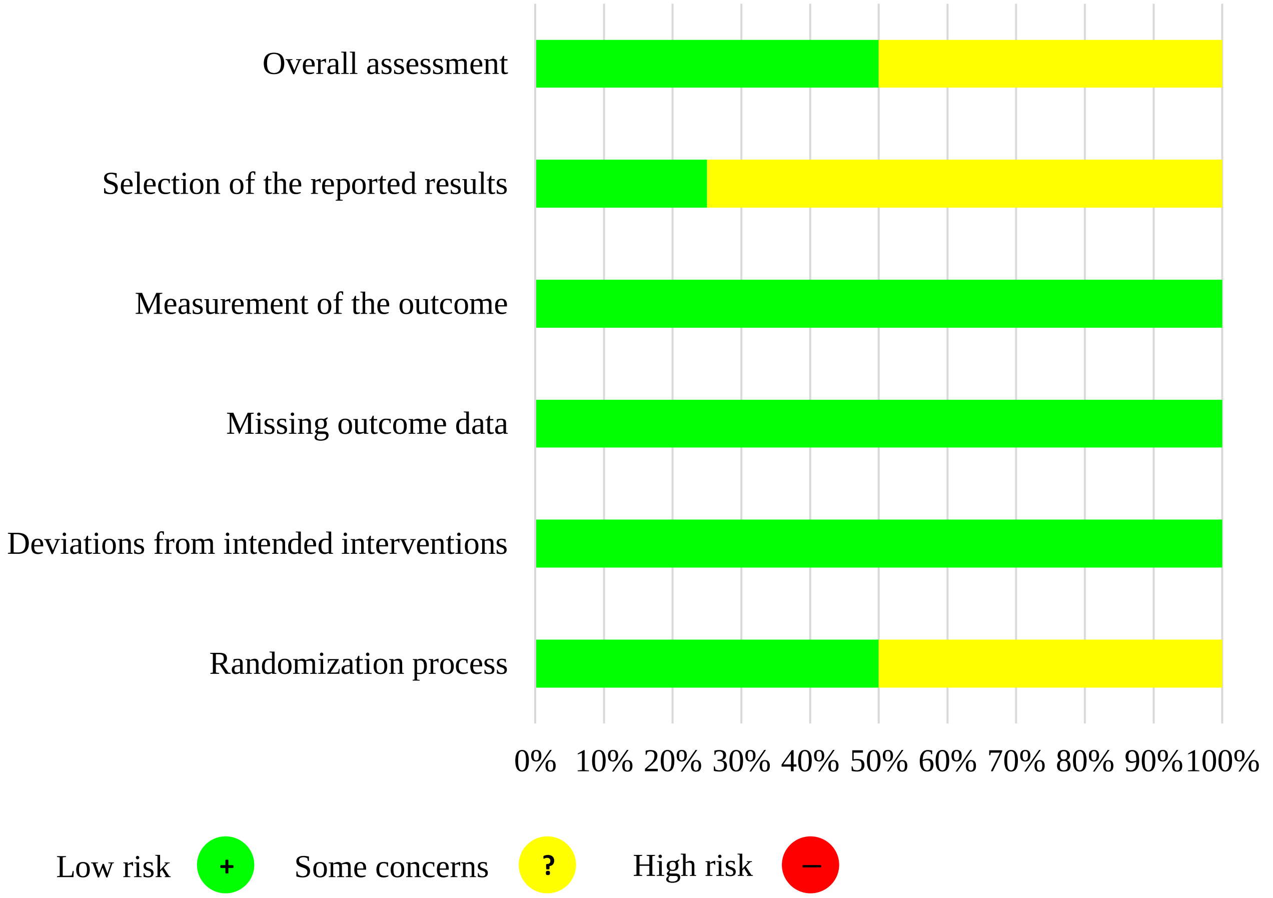

Supplementary Figure 55: Forest plot and Trial Sequential Analysis representing that addition of daratumumab to backbone therapies increases the chance for all grade neutropenia in relapsed/refractory multiple myeloma

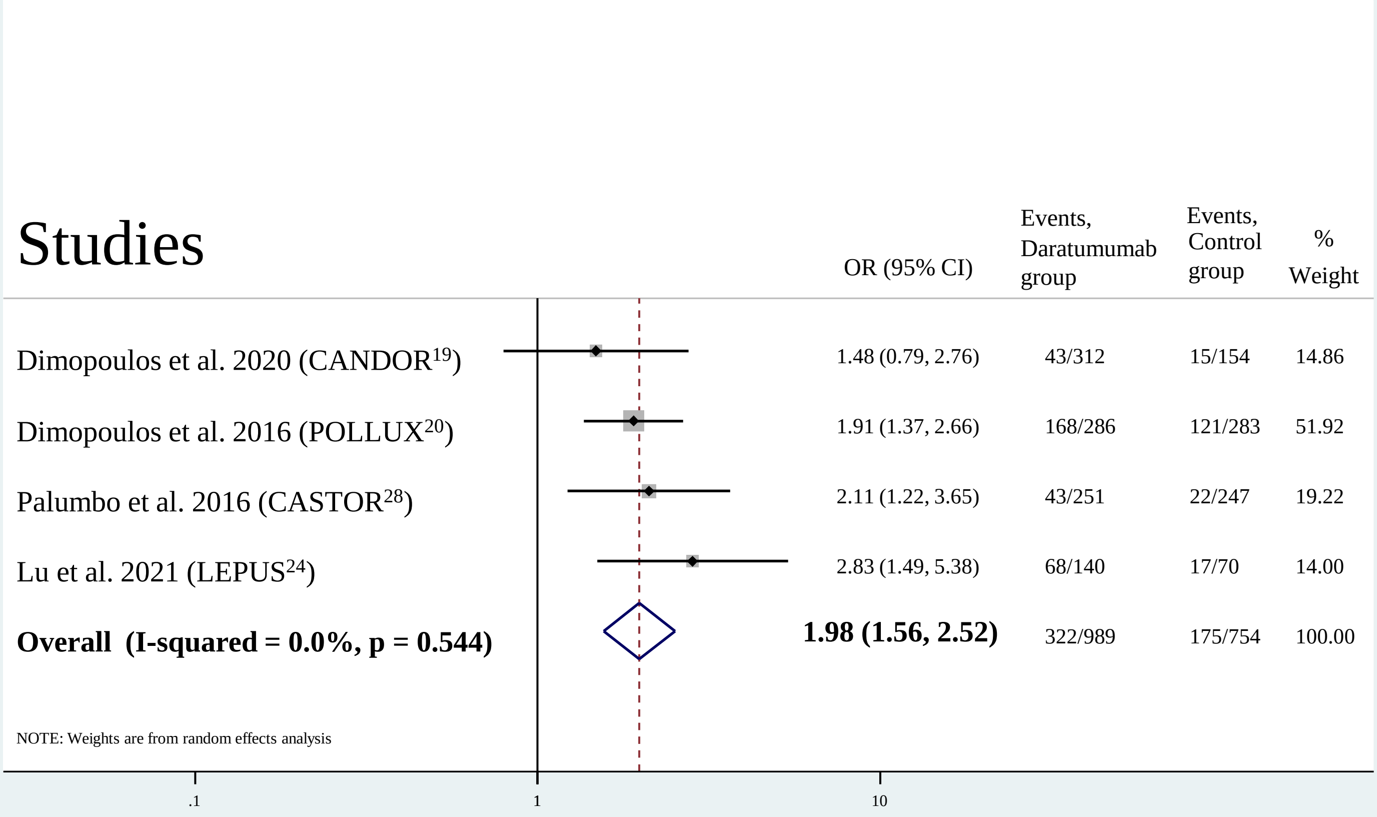

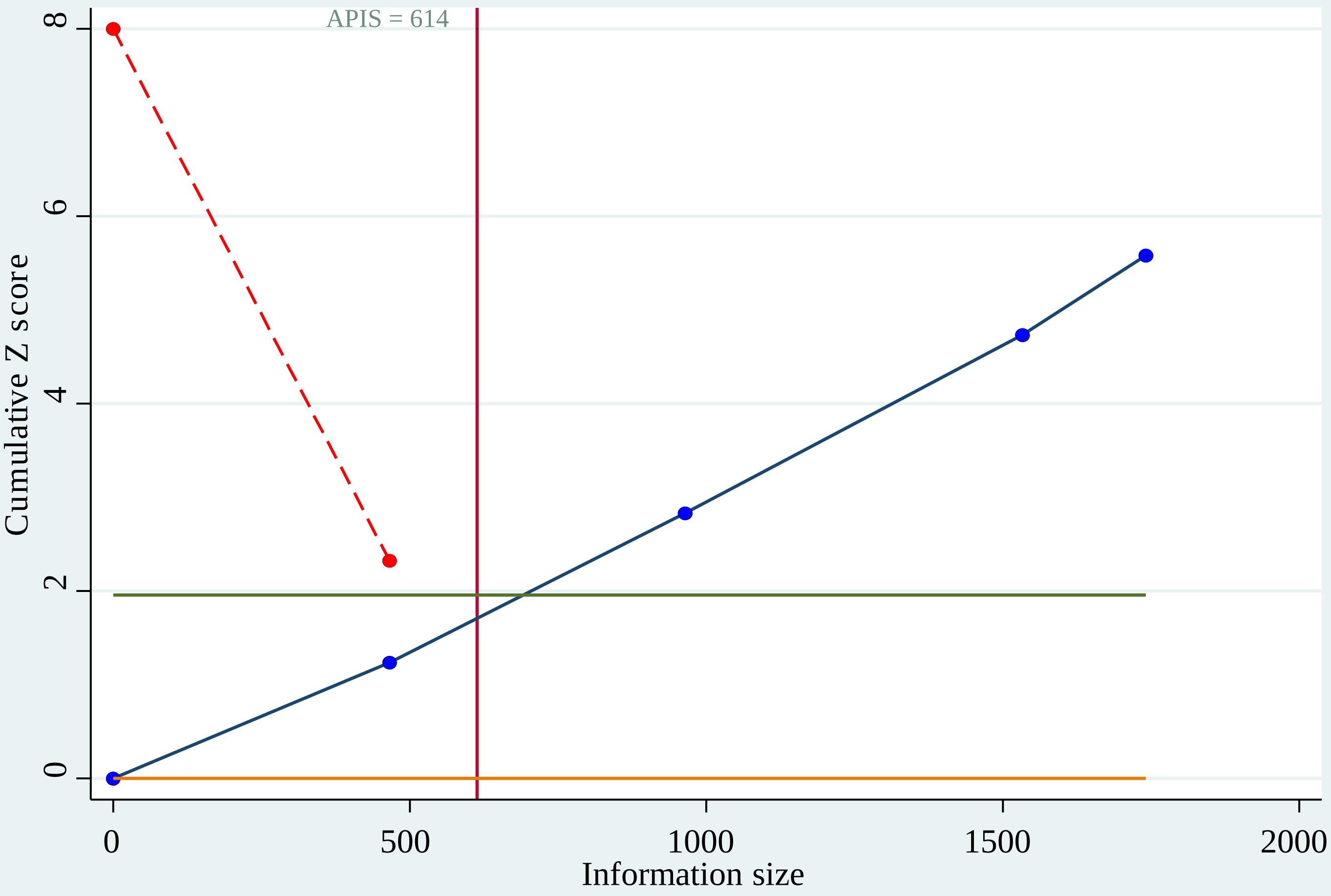

Supplementary Figure 56: Risk of bias assessment at study level and at domain level regarding all grade neutropenia in relapsed/refractory multiple myeloma
KdD, carfilzomib, dexamethasone, and daratumumab; Kd, carfilzomib and dexamethasone; DVd, daratumumab, bortezomib, and dexamethasone; Vd, bortezomib and dexamethasone; DRd, daratumumab, lenalidomide and dexamethasone; Rd, lenalidomide and dexamethasone

**Study**	**Interventions**	**Randomization process**	**Deviations from intended interventions**	**Missing outcome data**	**Measurement of the outcome**	**Selection of the reported results**	**Overall assessment**
Lu et al. 2021	DVd vs Vd	?	+	+	+	?	?
Dimopoulos et al. 2020	KdD vs Kd	+	+	+	+	+	+
Palumbo et al. 2016	DVd vs Vd	?	+	+	+	?	?
Dimopoulos et al. 2016	DRd vs Rd	+	+	+	+	?	+

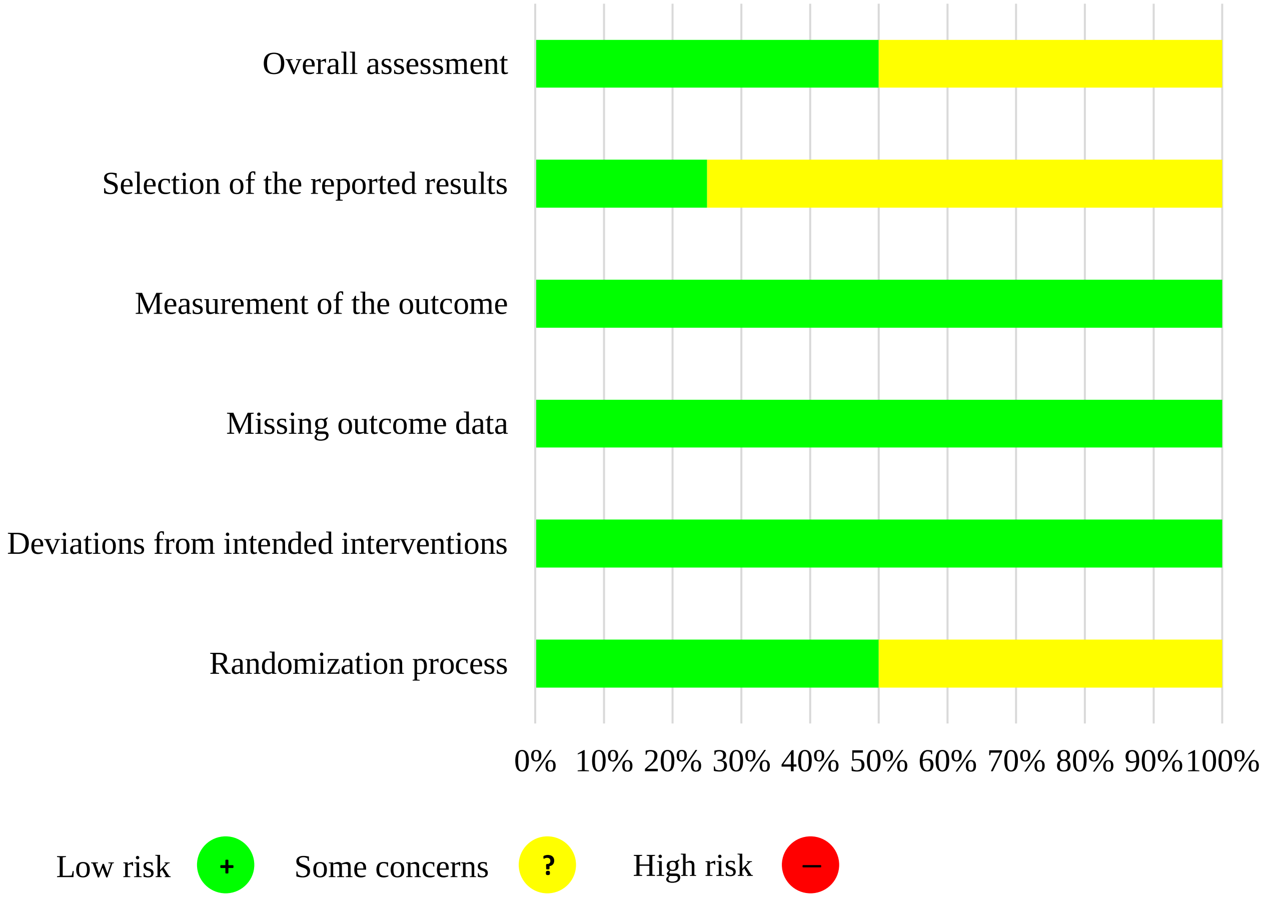

Supplementary Figure 57: Forest plot and Trial Sequential Analysis representing that addition of daratumumab to backbone therapies does not increase the chance for grade 3-4 neutropenia in relapsed/refractory multiple myeloma

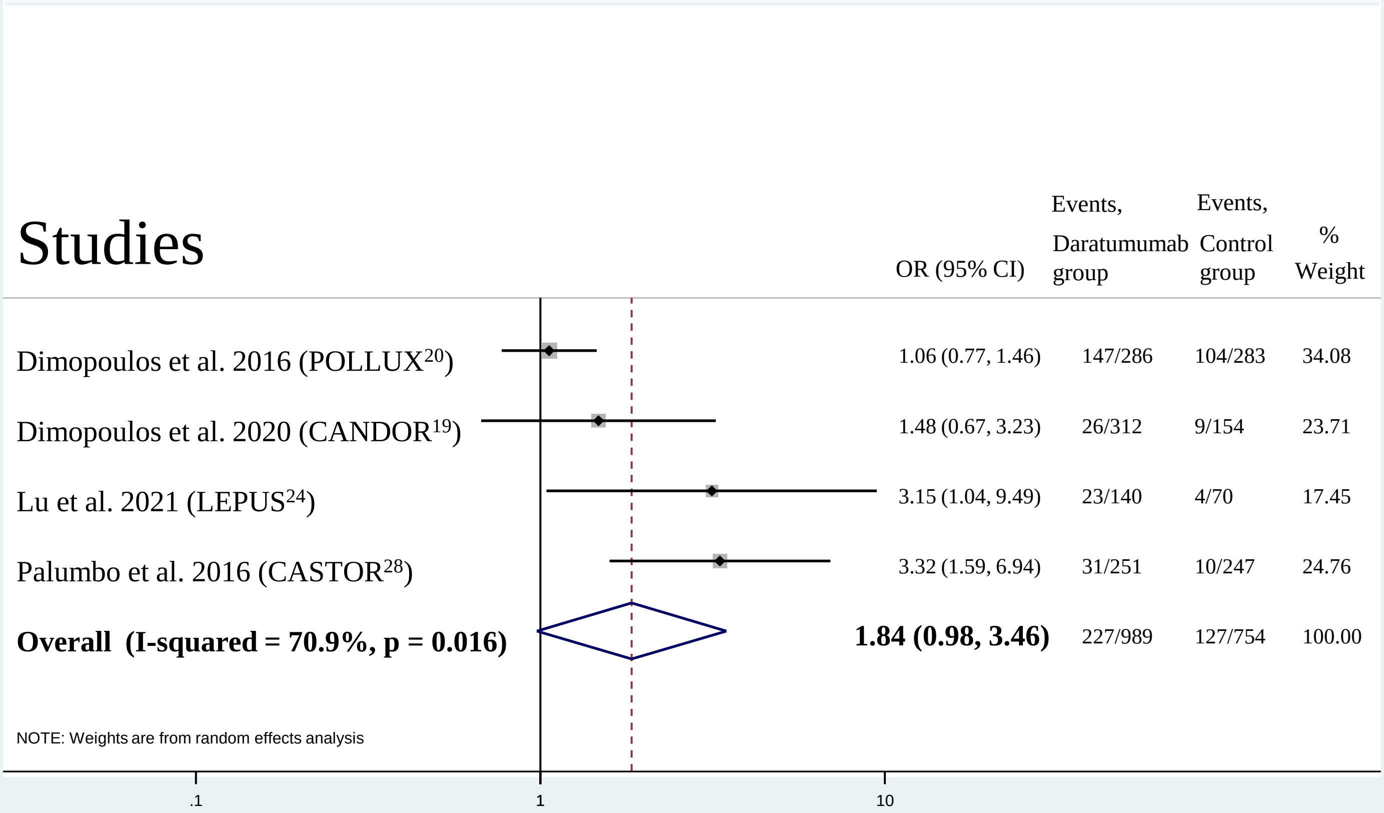

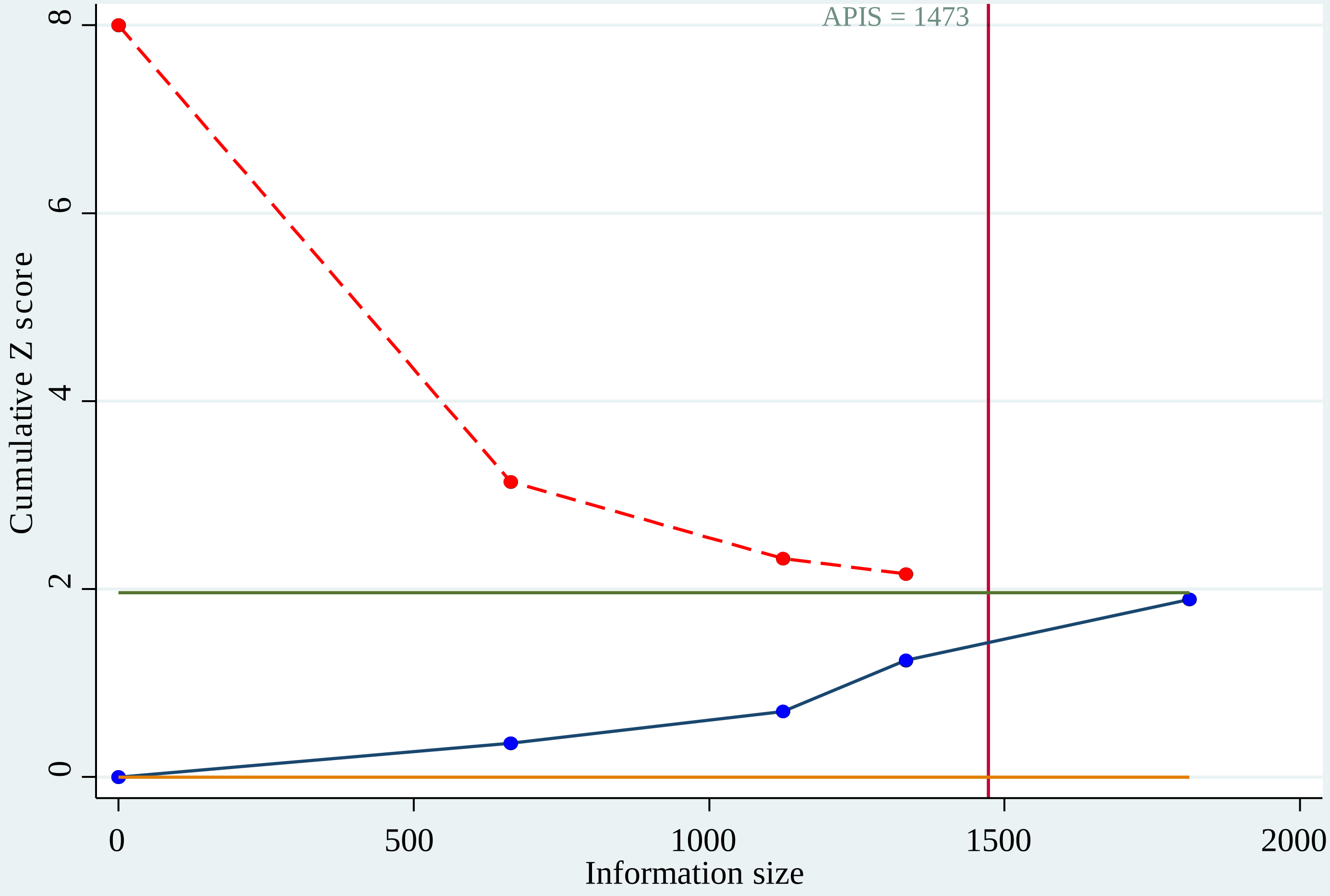

Supplementary Figure 58: Risk of bias assessment at study level and at domain level regarding grade 3-4 neutropenia in relapsed/refractory multiple myeloma
KdD, carfilzomib, dexamethasone, and daratumumab; Kd, carfilzomib and dexamethasone; DVd, daratumumab, bortezomib, and dexamethasone; Vd, bortezomib and dexamethasone; DRd, daratumumab, lenalidomide and dexamethasone; Rd, lenalidomide and dexamethasone

Study	Interventions	Randomization process	Deviations from intended interventions	Missing outcome data	Measurement of the outcome	Selection of the reported results	Overall assessment
Lu et al. 2021	DVd vs Vd	?	+	+	+	?	?
Dimopoulos et al. 2020	KdD vs Kd	+	+	+	+	+	+
Palumbo et al. 2016	DVd vs Vd	?	+	+	+	?	?
Dimopoulos et al. 2016	DRd vs Rd	+	+	+	+	?	+

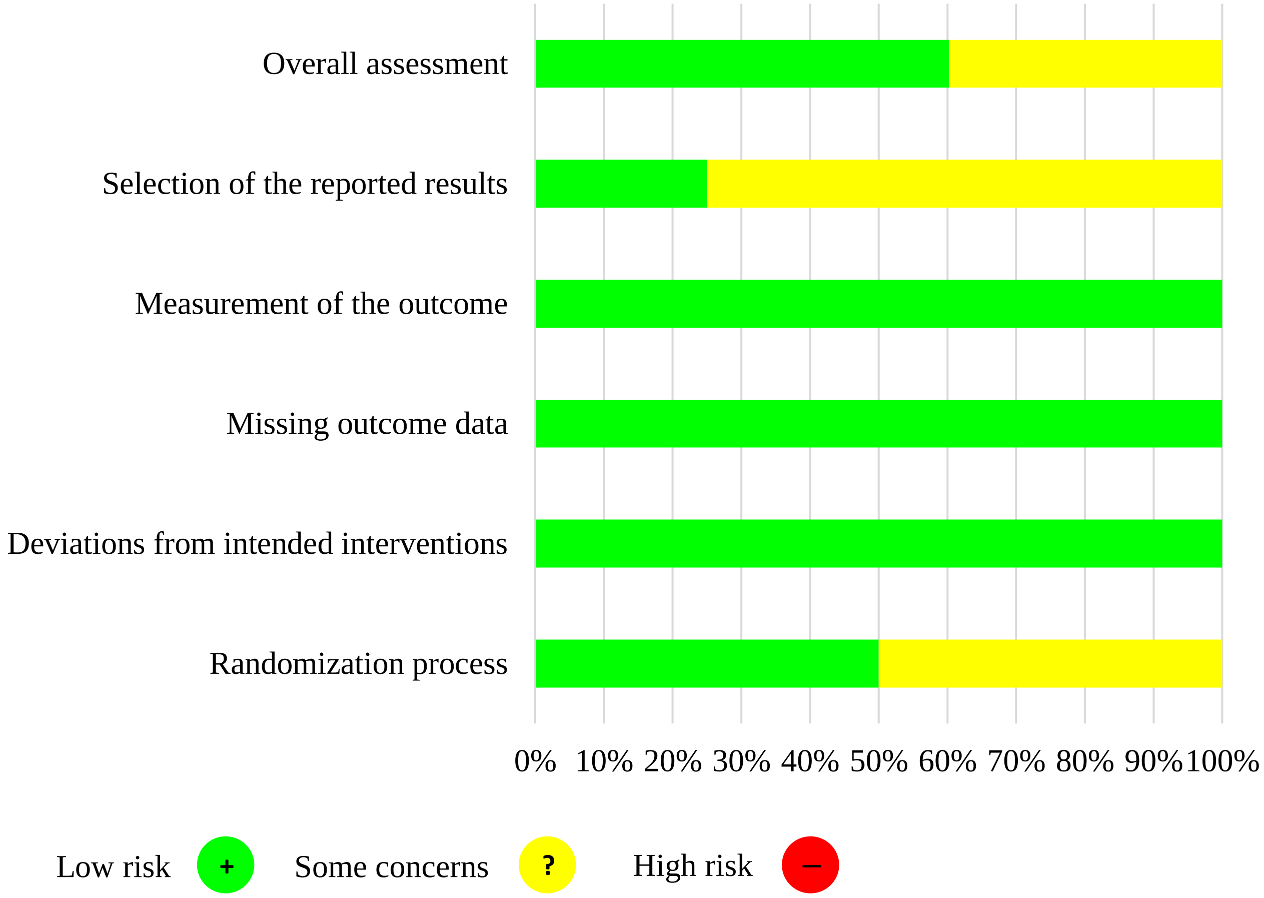

Supplementary Figure 59: Forest plot and Trial Sequential Analysis representing that addition of daratumumab to backbone therapies does not increase the chance for all grade anaemia relapsed/refractory multiple myeloma

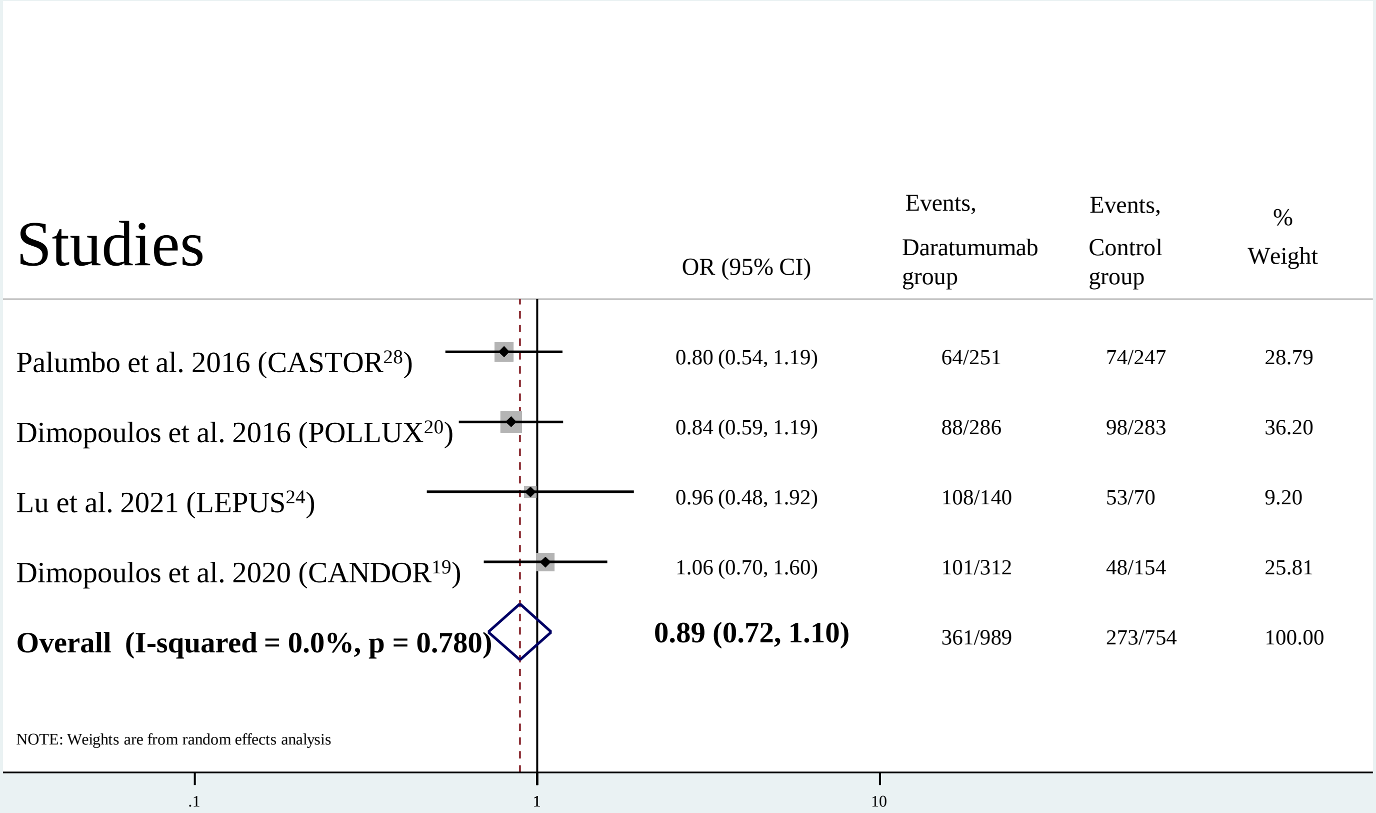

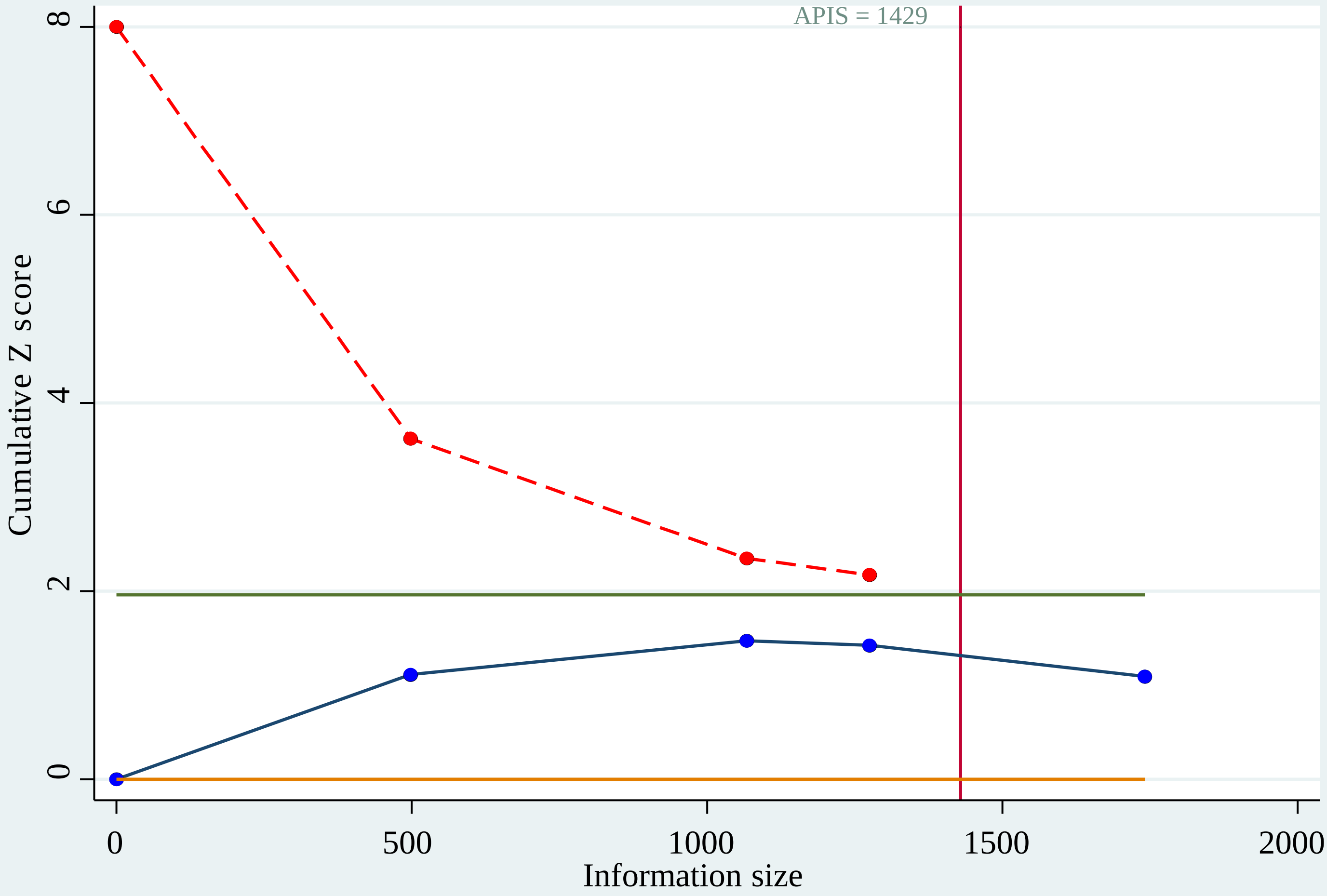

Supplementary Figure 60: Risk of bias assessment at study level and at domain level regarding all grade anaemia in relapsed/refractory multiple myeloma
KdD, carfilzomib, dexamethasone, and daratumumab; Kd, carfilzomib and dexamethasone; DVd, daratumumab, bortezomib, and dexamethasone; Vd, bortezomib and dexamethasone; DRd, daratumumab, lenalidomide and dexamethasone; Rd, lenalidomide and dexamethasone

Study	Interventions	Randomization process	Deviations from intended interventions	Missing outcome data	Measurement of the outcome	Selection of the reported results	Overall assessment
Lu et al. 2021	DVd vs Vd	?	+	+	+	?	?
Dimopoulos et al. 2020	KdD vs Kd	+	+	+	+	+	+
Palumbo et al. 2016	DVd vs Vd	?	+	+	+	?	?
Dimopoulos et al. 2016	DRd vs Rd	+	+	+	+	?	+

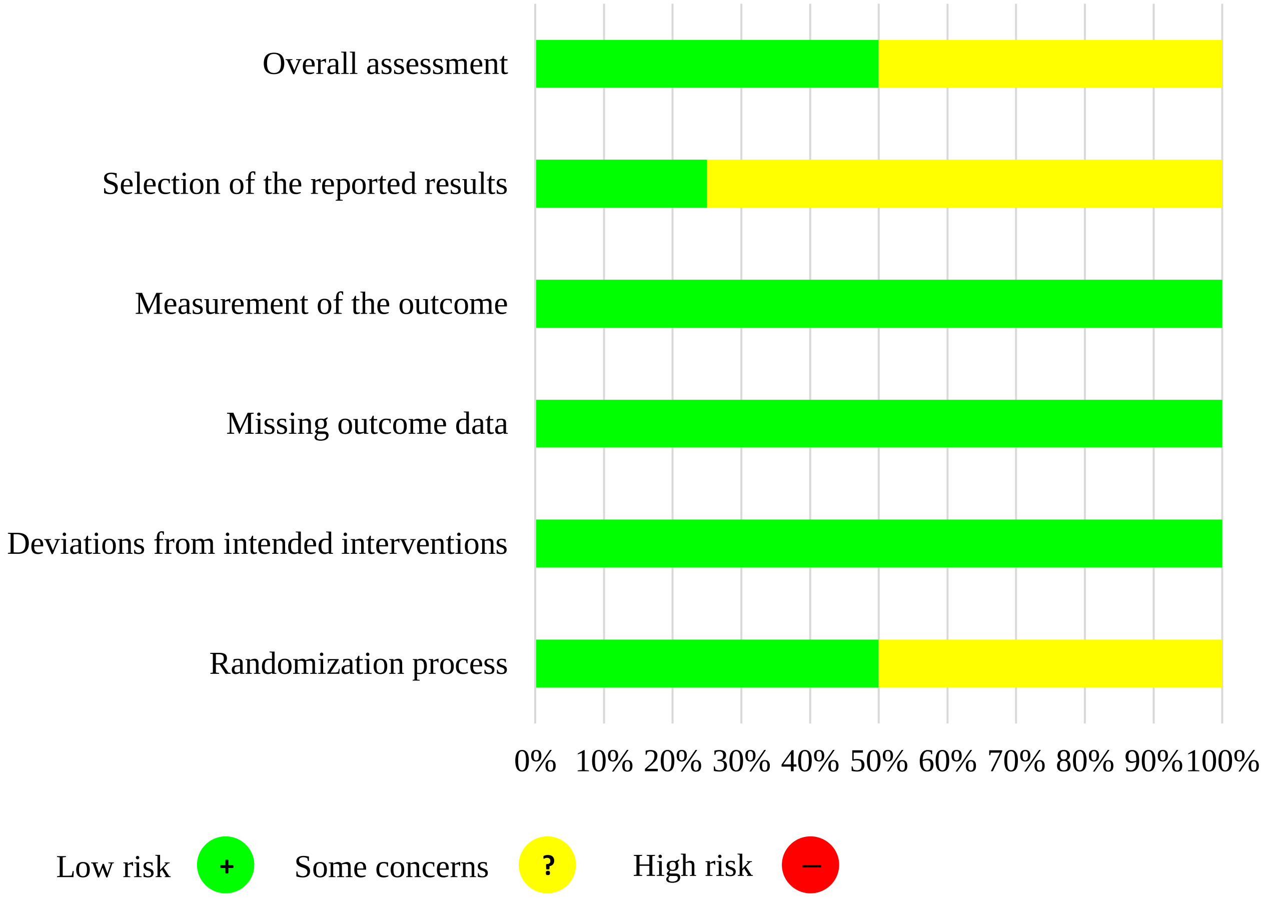

Supplementary Figure 61: Forest plot and Trial Sequential Analysis representing that addition of daratumumab to backbone therapies does not increase the chance for grade 3-4 anaemia relapsed/refractory multiple myeloma

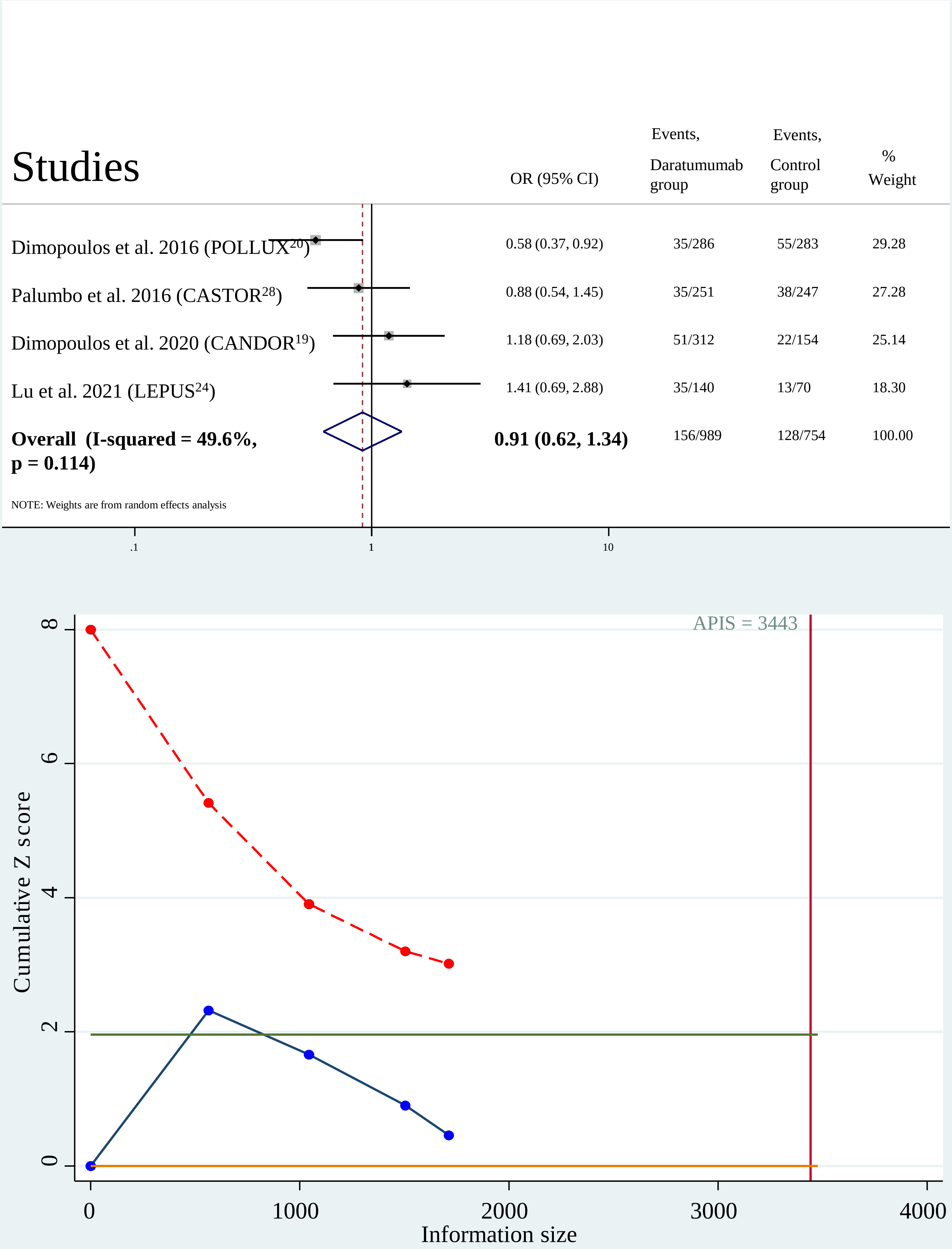

Supplementary Figure 62: Risk of bias assessment at study level and at domain level regarding grade 3-4 anaemia in relapsed/refractory multiple myeloma

KdD, carfilzomib, dexamethasone, and daratumumab; Kd, carfilzomib and dexamethasone; DVd, daratumumab, bortezomib, and dexamethasone; Vd, bortezomib and dexamethasone; DRd, daratumumab, lenalidomide and dexamethasone; Rd, lenalidomide and dexamethasone

Study	Interventions	Randomization process	Deviations from intended interventions	Missing outcome data	Measurement of the outcome	Selection of the reported results	Overall assessment
Lu et al. 2021	DVd vs Vd	?	+	+	+	?	?
Dimopoulos et al. 2020	KdD vs Kd	+	+	+	+	+	+
Palumbo et al. 2016	DVd vs Vd	?	+	+	+	?	?
Dimopoulos et al. 2016	DRd vs Rd	+	+	+	+	?	+

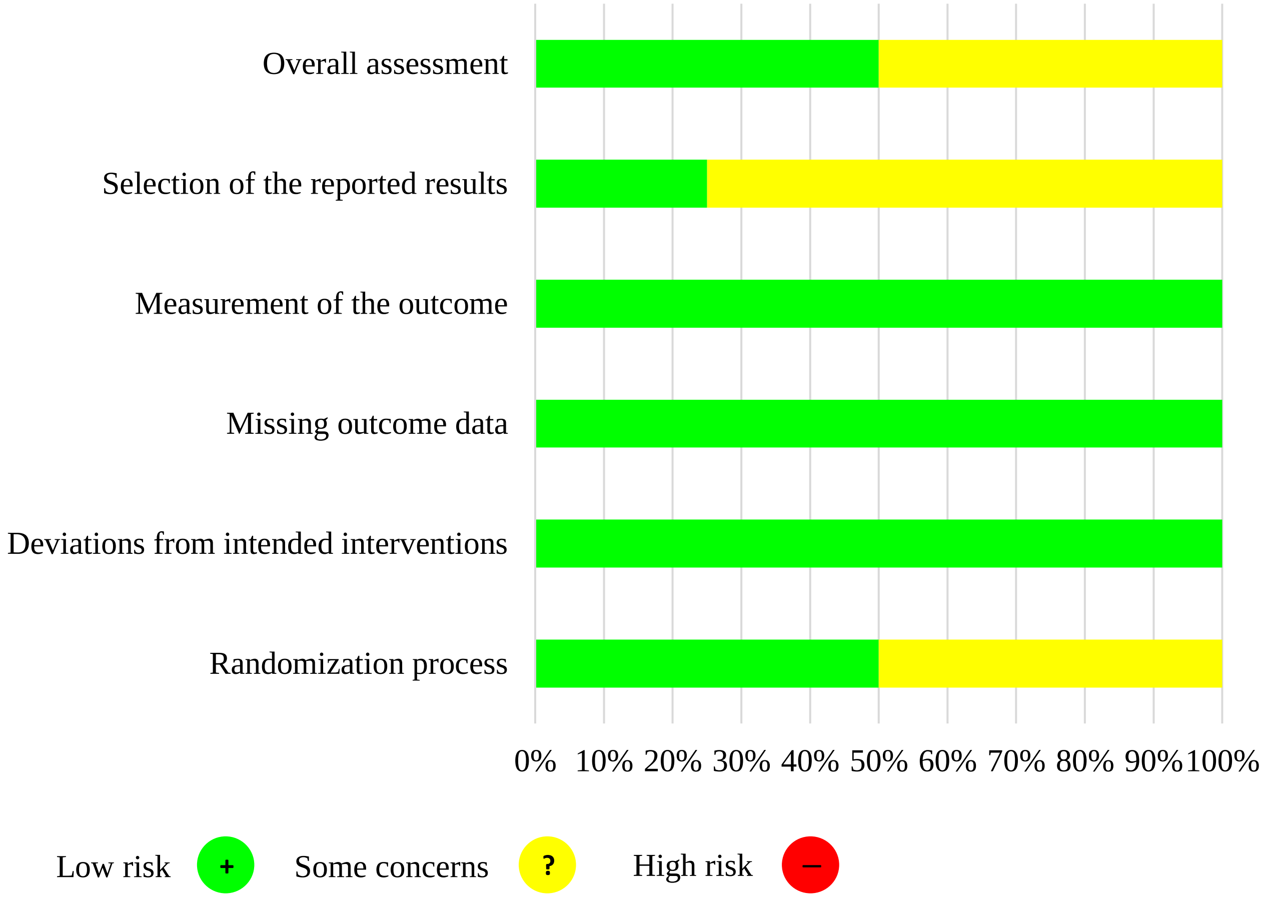

Supplementary Figure 63: Forest plot and Trial Sequential Analysis representing that addition of daratumumab to backbone therapies does not increase the chance for all grade hypertension in relapsed/refractory multiple myeloma

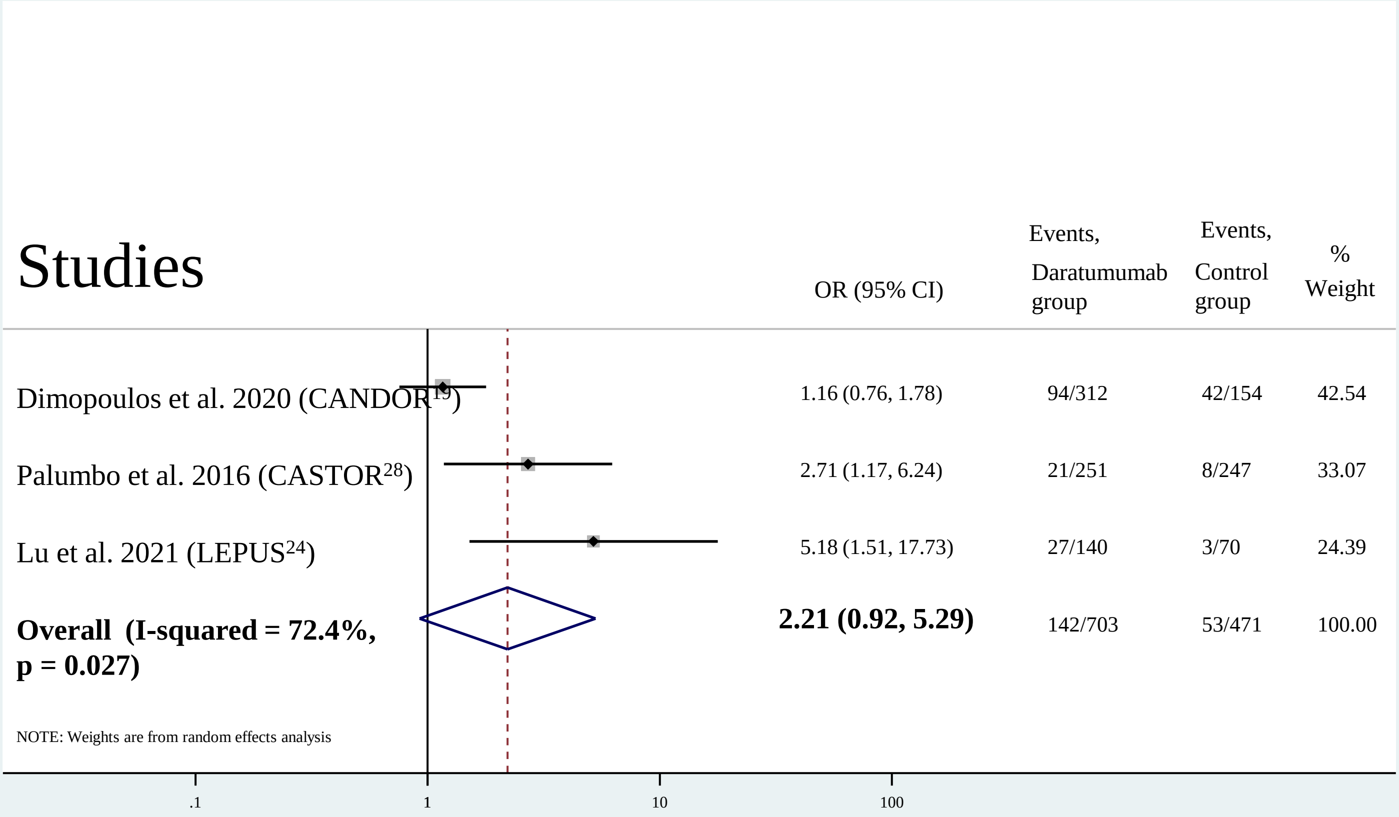

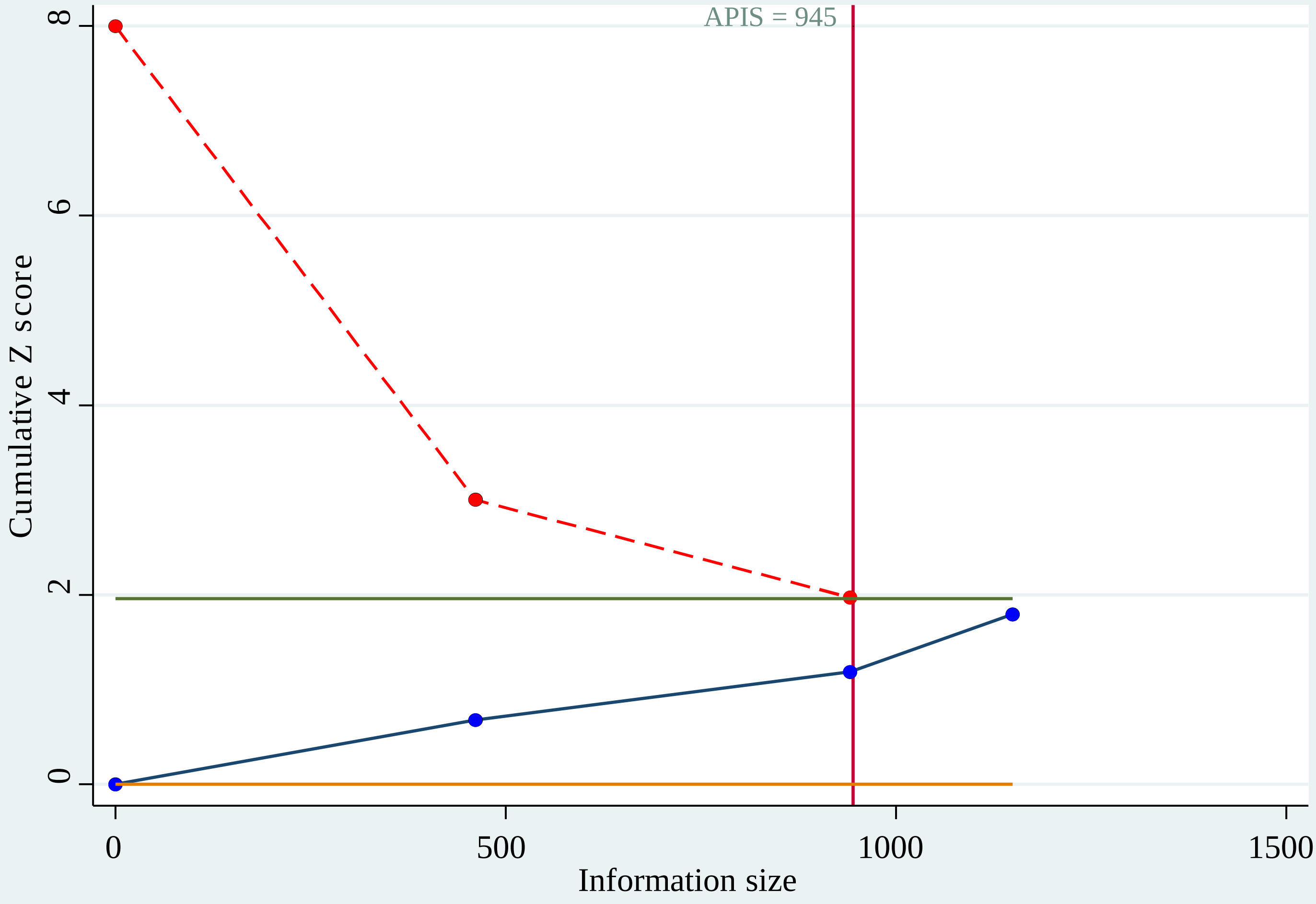

Supplementary Figure 64: Risk of bias assessment at study level and at domain level regarding all grade hypertension in relapsed/refractory multiple myeloma
DVd, daratumumab, bortezomib, and dexamethasone; Vd, bortezomib and dexamethasone; DRd, daratumumab, lenalidomide and dexamethasone; Rd, lenalidomide and dexamethasone

Study	Interventions	Randomization process	Deviations from intended interventions	Missing outcome data	Measurement of the outcome	Selection of the reported results	Overall assessment
Lu et al. 2021	DVd vs Vd	?	+	+	+	?	?
Dimopoulos et al. 2020	DRd vs Rd	+	+	+	+	+	+
Palumbo et al. 2016	DVd vs Vd	?	+	+	+	?	?

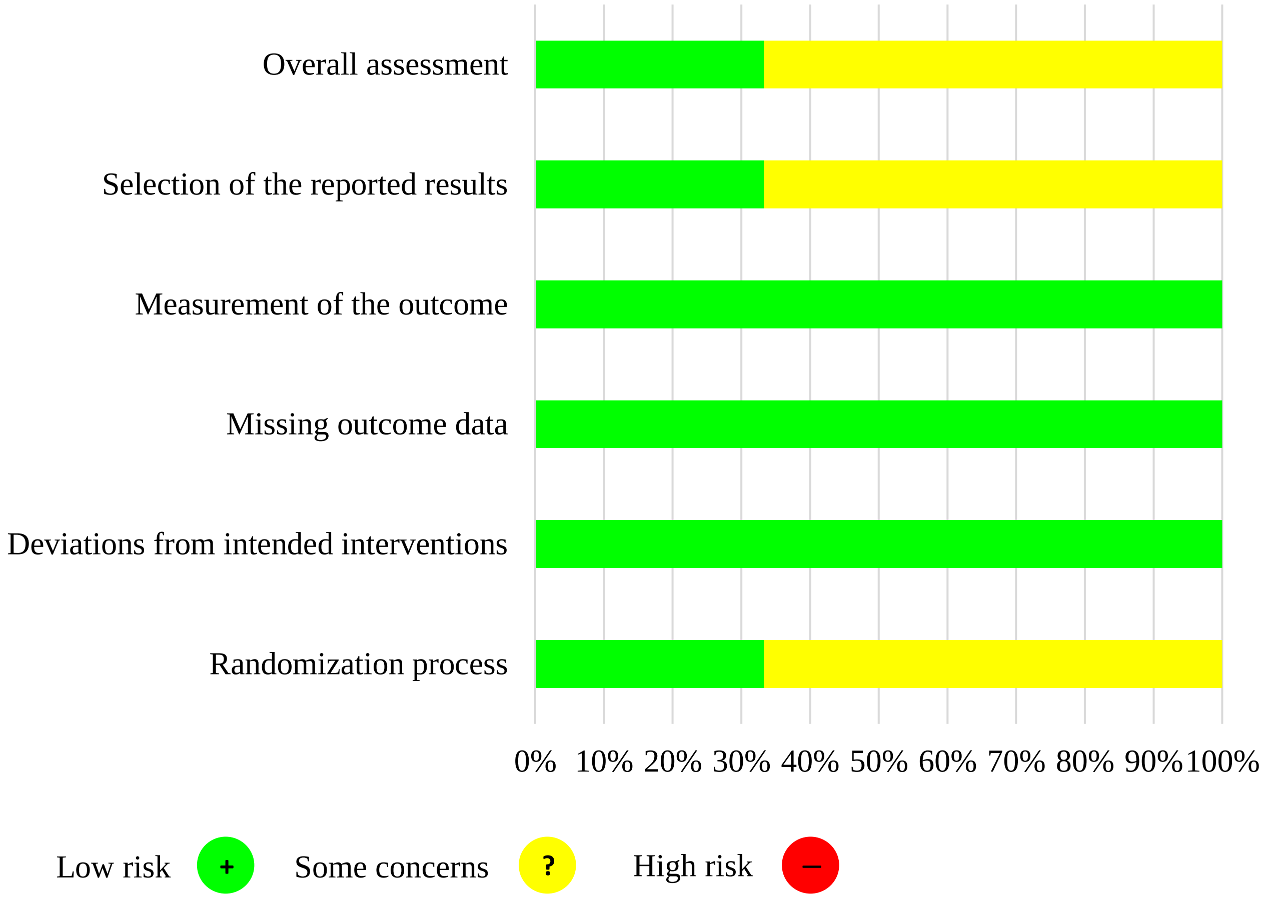

Supplementary Figure 65: Forest plot and Trial Sequential Analysis representing that addition of daratumumab to backbone therapies does not increase the chance for grade 3-4 hypertension in relapsed/refractory multiple myeloma

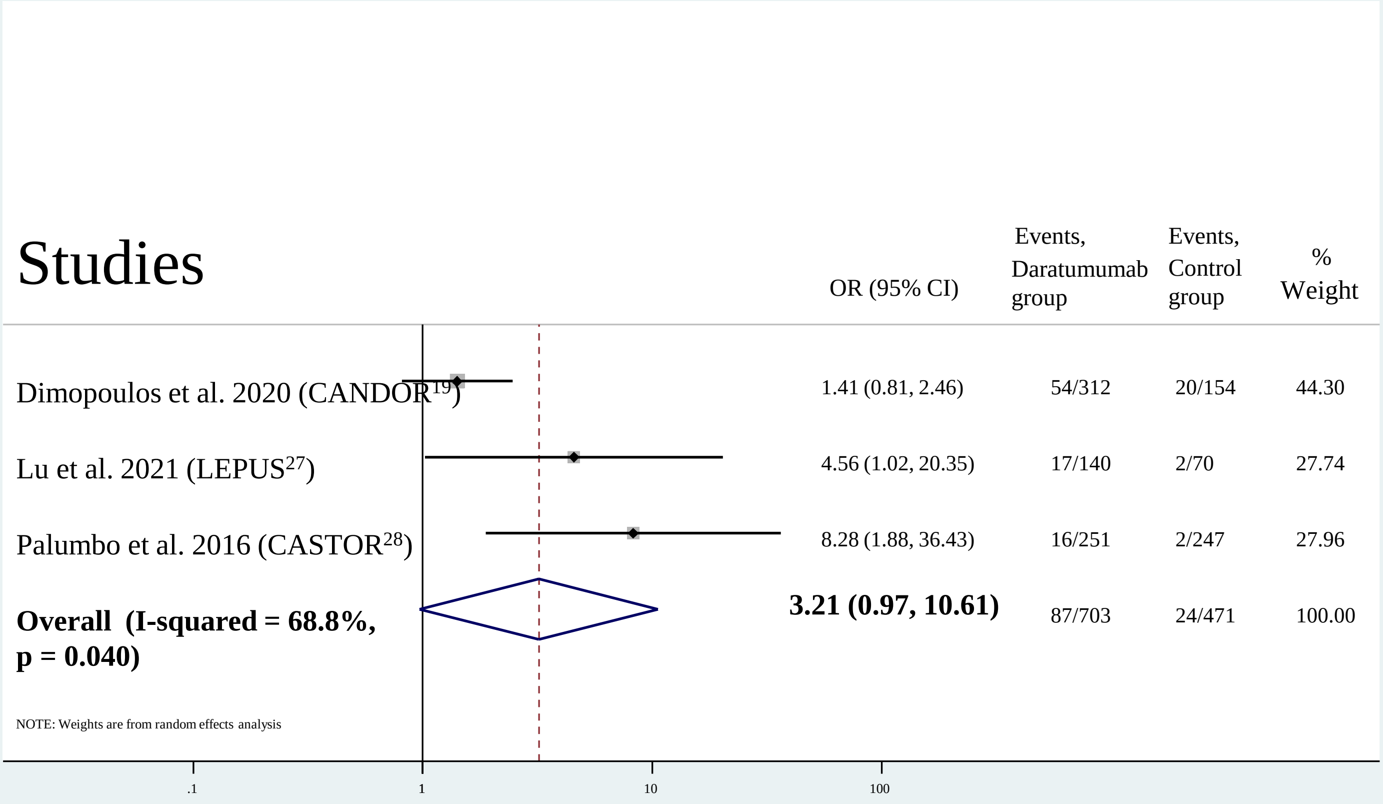

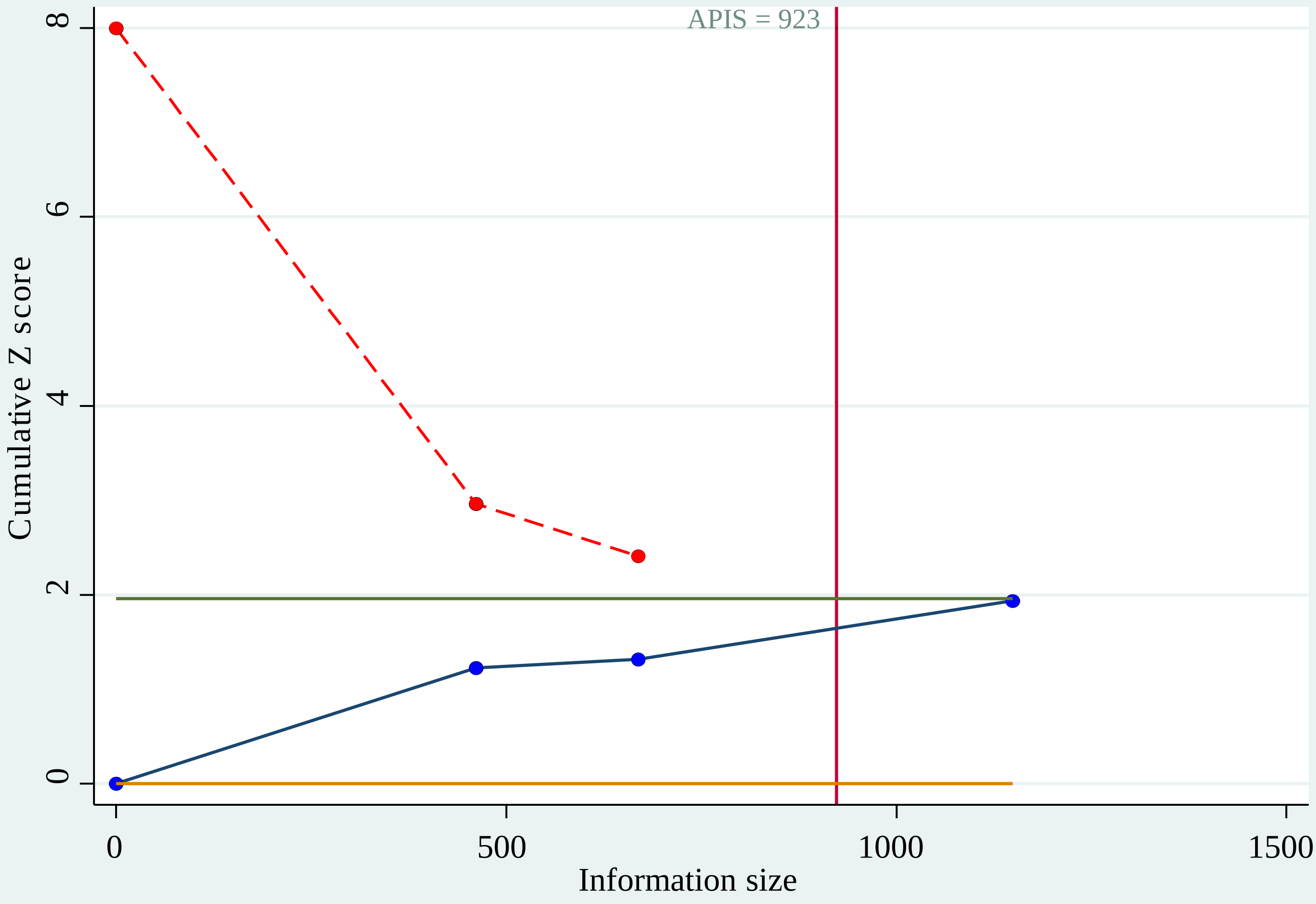

Supplementary Figure 66: Risk of bias assessment at study level and at domain level regarding grade 3-4 hypertension in relapsed/refractory multiple myeloma
DVd, daratumumab, bortezomib, and dexamethasone; Vd, bortezomib and dexamethasone; DRd, daratumumab, lenalidomide and dexamethasone; Rd, lenalidomide and dexamethasone

Study	Interventions	Randomization process	Deviations from intended interventions	Missing outcome data	Measurement of the outcome	Selection of the reported results	Overall assessment
Lu et al. 2021	DVd vs Vd	?	+	+	+	?	?
Dimopoulos et al. 2020	DRd vs Rd	+	+	+	+	+	+
Palumbo et al. 2016	DVd vs Vd	?	+	+	+	?	?

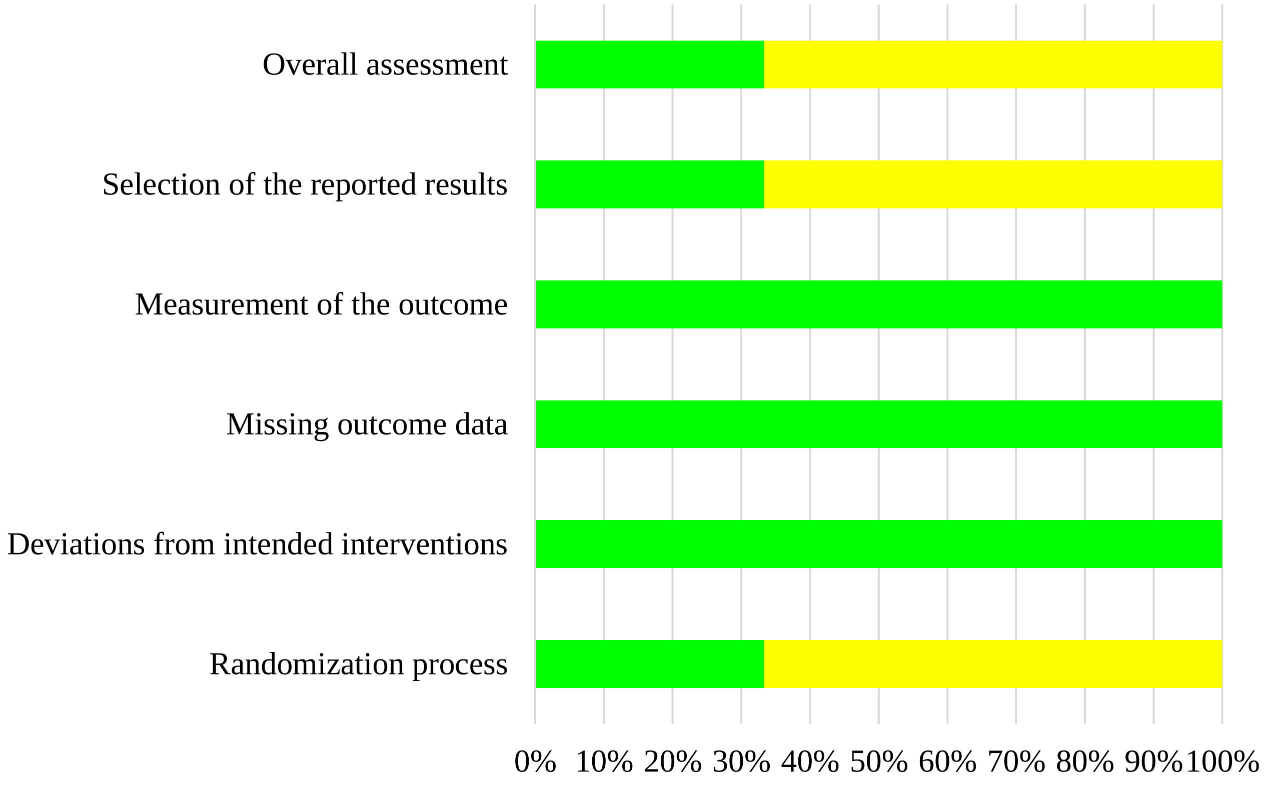

Low risk + Some concerns ? High risk –
