# Supplementary material for: Addition of daratumumab to multiple myeloma backbone regimens significantly improves clinical outcomes: a systematic review and meta-analysis of randomised controlled trials
Source: Sci Rep. 2021 Nov 9;11:21916. doi: 10.1038/s41598-021-01440-x (PMC8578422; doi:10.1038/s41598-021-01440-x)
Supplement: Supplementary file 2 — Supplementary Information 2. [file 41598_2021_1440_MOESM2_ESM.pdf]

Appendix B

Summary: This document contains certainty of evidence assessment using the GRADE Approach.

Addition of daratumumab to multiple myeloma backbone regimens: A systematic review and meta-analysis of randomised controlled trials

Szabolcs Kiss[1], Noémi Gede[2], Péter Hegyi[3], Bettina Nagy[2], Rita Deák[2], Fanni Dembrovszky[2], Stefania Bunduc[4], Bálint Erőss[2], Tamás Leiner[5], Zsolt Szakács[6 #], Hussain Alizadeh[6 # *]

[1]Doctoral School of Clinical Medicine, University of Szeged, Szeged, Hungary
Address: H-6720 Szeged, Korányi fasor 8-10., Hungary

[2]Institute for Translational Medicine, Medical School, University of Pécs, Pécs, Hungary
Address: H-7624 Pécs, Szigeti út 12. 2nd floor, Hungary

[3]Centre for Translational Medicine, Semmelweis University, Budapest, Hungary
Address: H-1085 Budapest, Üllői út 26., Hungary

[4]Doctoral school, Carol Davila University of Medicine and Pharmacy, Bucharest, Romania
Address: RO-050474 Bulevardul Eroii Sanitari 8, București, Romania

[5]North West Anglia NHS Foundation Trust, United Kingdom
Address: Parkway Hinchingbrooke, Huntingdon PE29 6NT, United Kingdom

[6]Division of Haematology, First Department of Medicine, Medical School, University of Pécs, Pécs, Hungary
Address: H-7624 Pécs, Ifjúság útja 13., Hungary

# equally contributed

*** Correspondence:**
Hussain Alizadeh M.D., Ph.D., Address: Division of Haematology, First Department of Medicine, Medical School, University of Pécs, H-7624 Pécs, Ifjúság Street 13., Hungary;
Tel: +36306436099
E-mail: alizadeh.hussain@pte.hu

Patient or population: newly diagnosed multiple myeloma

Intervention: daratumumab + backbone therapy

Comparison: backbone therapy

Outcomes	№ of participants (studies)	Certainty of the evidence (GRADE)	Relative effect (95% CI)	Anticipated absolute effects	
				Risk with backbone therapy	Risk difference with daratumumab + backbone therapy
**minimal residual disease negativity (MRD negativity)**	2735 (4 RCTs)	⊕⊕◯◯ LOW	OR 3.61 (2.33 to 5.61)	226 per 1 000	287 more per 1 000 (179 more to 395 more)
**stringent complete response (sCR)**	2735 (4 RCTs)	⊕⊕◯◯ LOW	OR 2.29 (1.49 to 3.51)	157 per 1 000	142 more per 1 000 (60 more to 238 more)
**complete response or better (CR or better)**	2735 (4 RCTs)	⊕⊕◯◯ LOW	OR 2.14 (1.66 to 2.75)	266 per 1 000	171 more per 1 000 (110 more to 233 more)
**death or disease progression**	2528 (3 RCTs)	⊕⊕⊕◯ MODERATE	HR 0.47 (0.39 to 0.57)	394 per 1 000	184 fewer per 1 000 (216 fewer to 146 fewer)

***The risk in the intervention group (and its 95% confidence interval) is based on the assumed risk in the comparison group and the relative effect of the intervention (and its 95% CI).**

CI: Confidence interval; OR: Odds ratio; HR: Hazard Ratio

GRADE Working Group grades of evidence
High certainty: We are very confident that the true effect lies close to that of the estimate of the effect
Moderate certainty: We are moderately confident in the effect estimate: The true effect is likely to be close to the estimate of the effect, but there is a possibility that it is substantially different
Low certainty: Our confidence in the effect estimate is limited: The true effect may be substantially different from the estimate of the effect
Very low certainty: We have very little confidence in the effect estimate: The true effect is likely to be substantially different from the estimate of effect

Patient or population: newly diagnosed multiple myeloma
Intervention: daratumumab + backbone therapy
Comparison: backbone therapy

Outcomes	№ of participants (studies)	Certainty of the evidence (GRADE)	Relative effect (95% CI)	Anticipated absolute effects	
				Risk with backbone therapy	Risk difference with daratumumab + backbone therapy
**all grade thrombocytopenia**	1998 (3 RCTs)	⊕⊕◯◯ LOW	OR 1.20 (0.76 to 1.88)	299 per 1 000	40 more per 1 000 (54 fewer to 146 more)
**grade 3-4 thrombocytopenia**	1998 (3 RCTs)	⊕◯◯◯ VERY LOW	OR 1.27 (0.77 to 2.09)	184 per 1 000	39 more per 1 000 (36 fewer to 136 more)
**all grade lymphopenia**	2021 (3 RCTs)	⊕⊕⊕◯ MODERATE	OR 1.49 (1.17 to 1.89)	139 per 1 000	55 more per 1 000 (20 more to 95 more)
**grade 3-4 lymphopenia**	2021 (3 RCTs)	⊕⊕⊕◯ MODERATE	OR 1.60 (1.21 to 2.11)	112 per 1 000	56 more per 1 000 (20 more to 98 more)
**all grade neutropenia**	2735 (4 RCTs)	⊕⊕◯◯ LOW	OR 1.63 (1.06 to 2.49)	339 per 1 000	116 more per 1 000 (13 more to 222 more)
**grade 3-4 neutropenia**	2735 (4 RCTs)	⊕⊕◯◯ LOW	OR 1.76 (1.20 to 2.59)	271 per 1 000	124 more per 1 000 (37 more to 219 more)
**all grade anaemia**	1650 (3 RCTs)	⊕◯◯◯ VERY LOW	OR 1.39 (0.61 to 3.13)	336 per 1 000	77 more per 1 000 (100 fewer to 277 more)

***The risk in the intervention group (and its 95% confidence interval) is based on the assumed risk in the comparison group and the relative effect of the intervention (and its 95% CI).**
CI: Confidence interval; OR: Odds ratio; HR: Hazard Ratio

GRADE Working Group grades of evidence
High certainty: We are very confident that the true effect lies close to that of the estimate of the effect
Moderate certainty: We are moderately confident in the effect estimate: The true effect is likely to be close to the estimate of the effect, but there is a possibility that it is substantially different. Low certainty: Our confidence in the effect estimate is limited: The true effect may be substantially different from the estimate of the effect. Very low certainty: We have very little confidence in the effect estimate: The true effect is likely to be substantially different from the estimate of effect

Patient or population: <u>newly diagnosed multiple myeloma</u>

Intervention: daratumumab + backbone therapy

Comparison: backbone therapy

Outcomes	№ of participants (studies)	Certainty of the evidence (GRADE)	Relative effect (95% CI)	Anticipated absolute effects	
				Risk with backbone therapy	Risk difference with daratumumab + backbone therapy
**grade 3-4 anaemia**	1650 (3 RCTs)	⊕◯◯◯ VERY LOW	OR 0.77 (0.49 to 1.23)	180 per 1 000	35 fewer per 1 000 (83 fewer to 33 more)
**all grade peripheral neuropathy**	1998 (3 RCTs)	⊕⊕⊕◯ MODERATE	OR 0.76 (0.63 to 0.92)	535 per 1 000	69 fewer per 1 000 (115 fewer to 21 fewer)
**grade 3-4 peripheral neuropathy**	1998 (3 RCTs)	⊕◯◯◯ VERY LOW	OR 0.8 (0.4 to 1.6)	64 per 1 000	12 fewer per 1 000 (37 fewer to 35 more)
**second primary cancer**	2528 (3 RCTs)	⊕◯◯◯ VERY LOW	OR 0.88 (0.54 to 1.45)	27 per 1 000	3 fewer per 1 000 (12 fewer to 12 more)

***The risk in the intervention group (and its 95% confidence interval) is based on the assumed risk in the comparison group and the relative effect of the intervention (and its 95% CI).**
CI: Confidence interval; OR: Odds ratio; HR: Hazard Ratio

GRADE Working Group grades of evidence
High certainty: We are very confident that the true effect lies close to that of the estimate of the effect
Moderate certainty: We are moderately confident in the effect estimate: The true effect is likely to be close to the estimate of the effect, but there is a possibility that it is substantially different
Low certainty: Our confidence in the effect estimate is limited: The true effect may be substantially different from the estimate of the effect
Very low certainty: We have very little confidence in the effect estimate: The true effect is likely to be substantially different from the estimate of effect

Patient or population: newly diagnosed multiple myeloma

Intervention: daratumumab + backbone therapy

Comparison: backbone therapy

Outcomes	№ of participants (studies)	Certainty of the evidence (GRADE)	Relative effect (95% CI)	Anticipated absolute effects	
				Risk with backbone therapy	Risk difference with daratumumab + backbone therapy
**All grade hypertension**	Not reported.				
**Grade 3-4 hypertension**	Not reported.				
**Acute heart failure**	Not reported.				
**Ischemic heart disease**	Not reported.				
**Renal failure**	Not reported.				

***The risk in the intervention group (and its 95% confidence interval) is based on the assumed risk in the comparison group and the relative effect of the intervention (and its 95% CI).**
CI: Confidence interval; OR: Odds ratio; HR: Hazard Ratio

GRADE Working Group grades of evidence
High certainty: We are very confident that the true effect lies close to that of the estimate of the effect
Moderate certainty: We are moderately confident in the effect estimate: The true effect is likely to be close to the estimate of the effect, but there is a possibility that it is substantially different. Low certainty: Our confidence in the effect estimate is limited: The true effect may be substantially different from the estimate of the effect. Very low certainty: We have very little confidence in the effect estimate: The true effect is likely to be substantially different from the estimate of effect .

Patient or population: newly diagnosed multiple myeloma

Intervention: daratumumab + backbone therapy

Comparison: backbone therapy

Certainty assessment							№ of patients		Effect		Certainty	Importance
**№ of studies**	Study design	Risk of bias	Inconsistency	Indirectness	Imprecision	Other considerations	daratumumab + backbone therapy	backbone therapy	Relative (95% CI)	Absolute (95% CI)		
**minimal residual disease negativity**												
4	randomised trials	not serious	serious	serious	not serious	none	587/1365 (43.0%)	309/1370 (22.6%)	OR 3.61 (2.33 to 5.61)	287 more per 1 000 (from 179 more to 395 more)	⊕⊕◯◯ LOW	IMPORTANT
**stringent complete response**												
4	randomised trials	not serious	serious	serious	not serious	none	392/1365 (28.7%)	215/1370 (15.7%)	OR 2.29 (1.49 to 3.51)	142 more per 1 000 (from 60 more to 238 more)	⊕⊕◯◯ LOW	IMPORTANT
**Complete response or better**												
4	randomised trials	not serious	serious	serious	not serious	none	597/1365 (43.7%)	364/1370 (26.6%)	OR 2.14 (1.66 to 2.75)	171 more per 1 000 (from 110 more to 233 more)	⊕⊕◯◯ LOW	IMPORTANT
**Death or disease progression**												
3	randomised trials	not serious	not serious	serious	not serious	none	318/1261 (25.2%)	499/1267 (39.4%)	HR 0.47 (0.39 to 0.57)	184 fewer per 1 000 (from 216 fewer to 146 fewer)	⊕⊕⊕◯ MODERATE	CRITICAL
**All grade thrombocytopenia**												
3	randomised trials	not serious	serious	serious	not serious	none	324/997 (32.5%)	299/1001 (29.9%)	OR 1.20 (0.76 to 1.88)	40 more per 1 000 (from 54 fewer to 146 more)	⊕⊕◯◯ LOW	IMPORTANT
**Grade 3-4 thrombocytopenia**												
3	randomised trials	not serious	serious	serious	serious	none	198/997 (19.9%)	183/1001 (18.3%)	OR 1.27 (0.77 to 2.09)	39 more per 1 000 (from 36 fewer to 136 more)	⊕◯◯◯ VERY LOW	IMPORTANT
**All grade lymphopenia**												
3	randomised trials	not serious	not serious	serious	not serious	none	195/1011 (19.3%)	140/1010 (13.9%)	OR 1.49 (1.17 to 1.89)	55 more per 1 000 (from 20 more to 95 more)	⊕⊕⊕◯ MODERATE	IMPORTANT
**Grade 3-4 lymphopenia**												
3	randomised trials	not serious	not serious	serious	not serious	none	169/1011 (16.7%)	113/1010 (11.2%)	OR 1.60 (1.21 to 2.11)	56 more per 1 000 (from 20 more to 98 more)	⊕⊕⊕◯ MODERATE	IMPORTANT

Patient or population: newly diagnosed multiple myeloma

Intervention: daratumumab + backbone therapy

Comparison: backbone therapy

Certainty assessment							№ of patients		Effect		Certainty	Importance
**№ of studies**	Study design	Risk of bias	Inconsistency	Indirectness	Imprecision	Other considerations	daratumumab + backbone therapy	backbone therapy	Relative (95% CI)	Absolute (95% CI)		
**All grade neutropenia**												
4	randomised trials	not serious	serious	serious	not serious	none	595/1365 (43.6%)	465/1370 (33.9%)	OR 1.63 (1.06 to 2.49)	116 more per 1 000 (from 13 more to 222 more)	⨁⨁◯◯ LOW	IMPORTANT
**Grade 3-4 neutropenia**												
4	randomised trials	not serious	serious	serious	not serious	none	510/1365 (37.4%)	368/1370 (26.9%)	OR 1.76 (1.20 to 2.59)	124 more per 1 000 (from 37 more to 219 more)	⨁⨁◯◯ LOW	IMPORTANT
**All grade anaemia**												
3	randomised trials	not serious	serious	serious	serious	none	351/822 (42.7%)	278/828 (33.6%)	OR 1.39 (0.61 to 3.13)	77 more per 1 000 (from 100 fewer to 277 more)	⨁◯◯◯ VERY LOW	IMPORTANT
**Grade 3-4 anaemia**												
3	randomised trials	not serious	serious	serious	serious	none	112/822 (13.6%)	148/828 (17.9%)	OR 0.77 (0.49 to 1.23)	35 fewer per 1 000 (from 83 fewer to 33 more)	⨁◯◯◯ VERY LOW	IMPORTANT
**All grade peripheral neuropathy**												
3	randomised trials	not serious	not serious	serious	not serious	none	473/997 (47.4%)	536/1001 (53.5%)	OR 0.76 (0.63 to 0.92)	69 fewer per 1 000 (from 115 fewer to 21 fewer)	⨁⨁⨁◯ MODERATE	IMPORTANT
**Grade 3-4 peripheral neuropathy**												
3	randomised trials	not serious	serious	serious	serious	none	57/997 (5.7%)	64/1001 (6.4%)	OR 0.8 (0.4 to 1.6)	12 fewer per 1 000 (from 37 fewer to 35 more)	⨁◯◯◯ VERY LOW	IMPORTANT
**Second primary cancer**												
3	randomised trials	not serious	serious	serious	serious	none	30/1261 (2.4%)	34/1267 (2.7%)	OR 0.88 (0.54 to 1.45)	3 fewer per 1 000 (from 12 fewer to 12 more)	⨁◯◯◯ VERY LOW	IMPORTANT

All grade hypertension, grade 3-4 hypertension, acute heart failure, ischemic heart failure, and renal failure were not reported.

Patient or population: newly diagnosed multiple myeloma with standard cytogenetic risk

Intervention: daratumumab + backbone therapy

Comparison: backbone therapy

Outcomes	№ of participants (studies)	Certainty of the evidence (GRADE)	Relative effect (95% CI)	Anticipated absolute effects	
				Risk with backbone therapy	Risk difference with daratumumab + backbone therapy
**death or disease progression**	1982 (3 RCTs)	⊕⊕◯◯ LOW	HR 0.43 (0.35 to 0.53)	291 per 1 000	153 fewer per 1 000 (178 fewer to 124 fewer)
**minimal residual disease negativity (MRD negativity)**	165 (1 RCT)	⊕⊕◯◯ LOW	OR 4.72 (2.37 to 9.40)	205 per 1 000	344 more per 1 000 (174 more to 503 more)
**stringent complete response (sCR)**	677 (2 RCTs)	⊕⊕◯◯ LOW	OR 2.51 (1.72 to 3.65)	151 per 1 000	158 more per 1 000 (83 more to 243 more)
**complete response or better (CR or better)**	518 (1 RCT)	⊕⊕◯◯ LOW	OR 2.70 (1.86 to 3.91)	257 per 1 000	226 more per 1 000 (134 more to 318 more)

***The risk in the intervention group (and its 95% confidence interval) is based on the assumed risk in the comparison group and the relative effect of the intervention (and its 95% CI).**

CI: Confidence interval; HR: Hazard Ratio; OR: Odds ratio

GRADE Working Group grades of evidence
High certainty: We are very confident that the true effect lies close to that of the estimate of the effect
Moderate certainty: We are moderately confident in the effect estimate: The true effect is likely to be close to the estimate of the effect, but there is a possibility that it is substantially different
Low certainty: Our confidence in the effect estimate is limited: The true effect may be substantially different from the estimate of the effect
Very low certainty: We have very little confidence in the effect estimate: The true effect is likely to be substantially different from the estimate of effect

Patient or population: newly diagnosed multiple myeloma with standard cytogenetic risk
Intervention: daratumumab + backbone therapy
Comparison: backbone therapy

Outcomes	№ of participants (studies)	Certainty of the evidence (GRADE)	Relative effect (95% CI)	Anticipated absolute effects	
				Risk with backbone therapy	Risk difference with daratumumab + backbone therapy
**All grade thrombocytopenia - not reported**	Not reported.				
**Grade 3-4 thrombocytopenia - not reported**	Not reported.				
**All grade lymphopenia - not reported**	Not reported.				
**Grade 3-4 lymphopenia - not reported**	Not reported.				
**All grade neutropenia - not reported**	Not reported.				
**Grade 3-4 neutropenia - not reported**	Not reported.				
**All grade anaemia - not reported**	Not reported.				
**Grade 3-4 anaemia - not reported**	Not reported.				
**All grade peripheral neuropathy - not reported**	Not reported.				
**Grade 3-4 peripheral neuropathy - not reported**	Not reported.				
**All grade hypertension - not reported**	Not reported.				
**Grade 3-4 hypertension - not reported**	Not reported.				
**Acute cardiac failure - not reported**	Not reported.				
**Ischaemic heart diease - not reported**	Not reported.				
**Renal failure - not reported**	Not reported.				

***The risk in the intervention group (and its 95% confidence interval) is based on the assumed risk in the comparison group and the relative effect of the intervention (and its 95% CI).**
CI: Confidence interval; HR: Hazard Ratio; OR: Odds ratio

GRADE Working Group grades of evidence
High certainty: We are very confident that the true effect lies close to that of the estimate of the effect
Moderate certainty: We are moderately confident in the effect estimate: The true effect is likely to be close to the estimate of the effect, but there is a possibility that it is substantially different
Low certainty: Our confidence in the effect estimate is limited: The true effect may be substantially different from the estimate of the effect
Very low certainty: We have very little confidence in the effect estimate: The true effect is likely to be substantially different from the estimate of effect

Patient or population: newly diagnosed multiple myeloma with standard cytogenetic risk

Intervention: daratumumab + backbone therapy

Comparison: backbone therapy

Certainty assessment							№ of patients		Effect		Certainty	Importance
**№ of studies**	Study design	Risk of bias	Inconsistency	Indirectness	Imprecision	Other considerations	daratumumab + backbone therapy	backbone therapy	Relative (95% CI)	Absolute (95% CI)		
**death or disease progression**												
3	randomised trials	not serious	not serious	serious	serious	none	992	990	HR 0.43 (0.35 to 0.53)	153 fewer per 1 000 (from 178 fewer to 124 fewer)	⨁⨁◯◯ LOW	CRITICAL
**Minimal residual disease negativity**												
1	randomised trials	serious	not serious	not serious	serious	none	45/82 (54.9%)	17/83 (20.5%)	OR 4.72 (2.37 to 9.40)	344 more per 1 000 (from 174 more to 503 more)	⨁⨁◯◯ LOW	IMPORTANT
**Stringent complete response**												
2	randomised trials	serious	not serious	not serious	serious	none	105/340 (30.9%)	51/337 (15.1%)	OR 2.51 (1.72 to 3.65)	158 more per 1 000 (from 83 more to 243 more)	⨁⨁◯◯ LOW	IMPORTANT
**Complete response or better**												
1	randomised trials	serious	not serious	not serious	serious	none	126/261 (48.3%)	66/257 (25.7%)	OR 2.70 (1.86 to 3.91)	226 more per 1 000 (from 134 more to 318 more)	⨁⨁◯◯ LOW	IMPORTANT

All grade thrombocytopenia, grade 3-4 thrombocytopenia, all grade lymphopenia, grade 3-4 lymphopenia, all grade neutropenia, grade 3-4 neutropenia, all grade anaemia, grade 3-4 anaemia, all grade peripheral neuropathy, grade 3-4 peripheral neuropathy, all grade hypertension, grade 3-4 hypertension, acute cardiac failure, ischaemic heart failure, and renal failure were not reported.

Patient or population: newly diagnosed multiple myeloma with high cytogenetic risk

Intervention: daratumumab + backbone therapy

Comparison: backbone therapy

**Outcomes**	№ of participants (studies)	Certainty of the evidence (GRADE)	Relative effect (95% CI)	Anticipated absolute effects	
				Risk with backbone therapy	Risk difference with daratumumab + backbone therapy
**death or disease progression**	358 (3 RCTs)	⊕⊕◯◯ LOW	HR 0.76 (0.53 to 1.10)	331 per 1 000	68 fewer per 1 000 (139 fewer to 26 more)
**minimal residual disease negativity (MRD negativity)**	30 (1 RCT)	⊕◯◯◯ VERY LOW	OR 1.50 (0.32 to 6.99)	286 per 1 000	89 more per 1 000 (172 fewer to 451 more)
**stringent complete response (sCR)**	312 (2 RCTs)	⊕◯◯◯ VERY LOW	OR 1.63 (0.91 to 2.92)	148 per 1 000	73 more per 1 000 (11 fewer to 188 more)
**complete response or better (CR or better)**	98 (1 RCT)	⊕⊕◯◯ LOW	OR 2.19 (0.92 to 5.25)	244 per 1 000	170 more per 1 000 (15 fewer to 385 more)

***The risk in the intervention group (and its 95% confidence interval) is based on the assumed risk in the comparison group and the relative effect of the intervention (and its 95% CI).**

CI: Confidence interval; HR: Hazard Ratio; OR: Odds ratio

GRADE Working Group grades of evidence
High certainty: We are very confident that the true effect lies close to that of the estimate of the effect
Moderate certainty: We are moderately confident in the effect estimate: The true effect is likely to be close to the estimate of the effect, but there is a possibility that it is substantially different
Low certainty: Our confidence in the effect estimate is limited: The true effect may be substantially different from the estimate of the effect
Very low certainty: We have very little confidence in the effect estimate: The true effect is likely to be substantially different from the estimate of effect

Patient or population: newly diagnosed multiple myeloma with high cytogenetic risk

Intervention: daratumumab + backbone therapy

Comparison: backbone therapy

Outcomes	№ of participants (studies)	Certainty of the evidence (GRADE)	Relative effect (95% CI)	Anticipated absolute effects	
				Risk with backbone therapy	Risk difference with daratumumab + backbone therapy
**All grade thrombocytopenia - not reported**	Not reported.				
**Grade 3-4 thrombocytopenia - not reported**	Not reported.				
**All grade lymphopenia - not reported**	Not reported.				
**Grade 3-4 lymphopenia - not reported**	Not reported.				
**All grade neutropenia - not reported**	Not reported.				
**Grade 3-4 neutropenia - not reported**	Not reported.				
**All grade anaemia - not reported**	Not reported.				
**Grade 3-4 anaemia - not reported**	Not reported.				
**All grade peripheral neuropathy - not reported**	Not reported.				
**Grade 3-4 peripheral neuropathy - not reported**	Not reported.				
**All grade hypertension - not reported**	Not reported.				
**Grade 3-4 hypertension - not reported**	Not reported.				
**Acute cardiac failure - not reported**	Not reported.				
**Ischaemic heart diease - not reported**	Not reported.				
**Renal failure - not reported**	Not reported.				

***The risk in the intervention group (and its 95% confidence interval) is based on the assumed risk in the comparison group and the relative effect of the intervention (and its 95% CI).**
CI: Confidence interval; HR: Hazard Ratio; OR: Odds ratio

GRADE Working Group grades of evidence
High certainty: We are very confident that the true effect lies close to that of the estimate of the effect
Moderate certainty: We are moderately confident in the effect estimate: The true effect is likely to be close to the estimate of the effect, but there is a possibility that it is substantially different
Low certainty: Our confidence in the effect estimate is limited: The true effect may be substantially different from the estimate of the effect
Very low certainty: We have very little confidence in the effect estimate: The true effect is likely to be substantially different from the estimate of effect

Patient or population: newly diagnosed multiple myeloma with high cytogenetic risk

Intervention: daratumumab + backbone therapy

Comparison: backbone therapy

Certainty assessment							№ of patients		Effect		Certainty	Importance
**№ of studies**	Study design	Risk of bias	Inconsistency	Indirectness	Imprecision	Other considerations	daratumumab + backbone therapy	backbone therapy	Relative (95% CI)	Absolute (95% CI)		
**death or disease progression**												
3	randomised trials	not serious	not serious	serious	serious	none	183	175	HR 0.76 (0.53 to 1.10)	68 fewer per 1 000 (from 139 fewer to 26 more)	⊕⊕◯◯ LOW	CRITICAL
**minimal residual disease negativity**												
1	randomised trials	serious	not serious	not serious	very serious	none	6/16 (37.5%)	4/14 (28.6%)	OR 1.50 (0.32 to 6.99)	89 more per 1 000 (from 172 fewer to 451 more)	⊕◯◯◯ VERY LOW	IMPORTANT
**stringent complete response**												
2	randomised trials	not serious	not serious	serious	very serious	none	30/136 (22.1%)	26/176 (14.8%)	OR 1.63 (0.91 to 2.92)	73 more per 1 000 (from 11 fewer to 188 more)	⊕◯◯◯ VERY LOW	IMPORTANT
**complete response or better**												
1	randomised trials	not serious	not serious	not serious	very serious	none	22/53 (41.5%)	11/45 (24.4%)	OR 2.19 (0.92 to 5.25)	170 more per 1 000 (from 15 fewer to 385 more)	⊕⊕◯◯ LOW	IMPORTANT

All grade thrombocytopenia, grade 3-4 thrombocytopenia, all grade lymphopenia, grade 3-4 lymphopenia, all grade neutropenia, grade 3-4 neutropenia, all grade anaemia, grade 3-4 anaemia, all grade peripheral neuropathy, grade 3-4 peripheral neuropathy, all grade hypertension, grade 3-4 hypertension, acute cardiac failure, ischaemic heart failure, and renal failure were not reported.

Patient or population: relapsed/refractory multiple myeloma

Intervention: daratumumab + backbone therapy

Comparison: backbone therapy

Outcomes	№ of participants (studies)	Certainty of the evidence (GRADE)	Relative effect (95% CI)	Anticipated absolute effects	
				Risk with backbone therapy	Risk difference with daratumumab + backbone therapy
**minimal residual disease negativity**	970 (3 RCTs)	⨁⨁⨁◯ MODERATE	OR 5.43 (2.76 to 10.66)	27 per 1 000	104 more per 1 000 (44 more to 201 more)
**stringent complete respone**	1571 (4 RCTs)	⨁⨁⨁◯ MODERATE	OR 3.08 (2.00 to 4.76)	40 per 1 000	74 more per 1 000 (37 more to 126 more)
**complete response or better**	1571 (4 RCTs)	⨁⨁⨁◯ MODERATE	OR 3.50 (2.33 to 5.25)	117 per 1 000	199 more per 1 000 (119 more to 293 more)
**death or disease progression**	2048 (5 RCTs)	⨁⨁◯◯ LOW	HR 0.50 (0.37 to 0.67)	559 per 1 000	223 fewer per 1 000 (298 fewer to 137 fewer)

***The risk in the intervention group (and its 95% confidence interval) is based on the assumed risk in the comparison group and the relative effect of the intervention (and its 95% CI).**

CI: Confidence interval; OR: Odds ratio; HR: Hazard Ratio

GRADE Working Group grades of evidence
High certainty: We are very confident that the true effect lies close to that of the estimate of the effect
Moderate certainty: We are moderately confident in the effect estimate: The true effect is likely to be close to the estimate of the effect, but there is a possibility that it is substantially different
Low certainty: Our confidence in the effect estimate is limited: The true effect may be substantially different from the estimate of the effect
Very low certainty: We have very little confidence in the effect estimate: The true effect is likely to be substantially different from the estimate of effect

Patient or population: relapsed/refractory multiple myeloma

Intervention: daratumumab + backbone therapy

Comparison: backbone therapy

Outcomes	№ of participants (studies)	Certainty of the evidence (GRADE)	Relative effect (95% CI)	Anticipated absolute effects	
				Risk with backbone therapy	Risk difference with daratumumab + backbone therapy
**all grade thrombocytopenia**	1743 (4 RCTs)	⊕⊕◯◯ LOW	OR 1.58 (1.03 to 2.43)	366 per 1 000	111 more per 1 000 (7 more to 218 more)
**grade 3-4 thrombocytopenia**	1743 (4 RCTs)	⊕⊕⊕◯ MODERATE	OR 1.47 (1.10 to 1.98)	220 per 1 000	73 more per 1 000 (17 more to 138 more)
**all grade lymphopenia**	1743 (4 RCTs)	⊕◯◯◯ VERY LOW	OR 1.54 (0.89 to 2.66)	94 per 1 000	44 more per 1 000 (9 fewer to 122 more)
**grade 3-4 lymphopenia**	1743 (4 RCTs)	⊕⊕◯◯ LOW	OR 1.75 (1.02 to 2.99)	62 per 1 000	42 more per 1 000 (1 more to 103 more)
**all grade neutropenia**	1743 (4 RCTs)	⊕⊕⊕◯ MODERATE	OR 1.98 (1.56 to 2.52)	232 per 1 000	142 more per 1 000 (88 more to 200 more)
**grade 3-4 neutropenia**	1743 (4 RCTs)	⊕⊕◯◯ LOW	OR 1.84 (0.98 to 3.46)	168 per 1 000	102 more per 1 000 (3 fewer to 244 more)

***The risk in the intervention group (and its 95% confidence interval) is based on the assumed risk in the comparison group and the relative effect of the intervention (and its 95% CI).**
CI: Confidence interval; OR: Odds ratio; HR: Hazard Ratio

GRADE Working Group grades of evidence
High certainty: We are very confident that the true effect lies close to that of the estimate of the effect
Moderate certainty: We are moderately confident in the effect estimate: The true effect is likely to be close to the estimate of the effect, but there is a possibility that it is substantially different. Low certainty: Our confidence in the effect estimate is limited: The true effect may be substantially different from the estimate of the effect. Very low certainty: We have very little confidence in the effect estimate: The true effect is likely to be substantially different from the estimate of effect

Patient or population: relapsed/refractory multiple myeloma

Intervention: daratumumab + backbone therapy

Comparison: backbone therapy

Outcomes	№ of participants (studies)	Certainty of the evidence (GRADE)	Relative effect (95% CI)	Anticipated absolute effects	
				Risk with backbone therapy	Risk difference with daratumumab + backbone therapy
**all grade anaemia**	1743 (4 RCTs)	⊕⊕⊕◯ MODERATE	OR 0.89 (0.72 to 1.10)	362 per 1 000	26 fewer per 1 000 (72 fewer to 22 more)
**grade 3-4 anaemia**	1743 (4 RCTs)	⊕◯◯◯ VERY LOW	OR 0.91 (0.62 to 1.34)	170 per 1 000	13 fewer per 1 000 (57 fewer to 45 more)
**all grade hypertension**	1174 (3 RCTs)	⊕⊕◯◯ LOW	OR 2.21 (0.92 to 5.29)	113 per 1 000	106 more per 1 000 (8 fewer to 289 more)
**grade 3-4 hypertension**	1174 (3 RCTs)	⊕⊕◯◯ LOW	OR 3.21 (0.97 to 10.61)	51 per 1 000	96 more per 1 000 (1 fewer to 312 more)
**all grade neuropathy**	941 (2 RCTs)	⊕⊕◯◯ LOW	OR 1.24 (0.93 to 1.65)	262 per 1 000	44 more per 1 000 (14 fewer to 107 more)
**grade 3-4 neuropathy**	941 (2 RCTs)	⊕⊕◯◯ LOW	OR 0.48 (0.22 to 1.04)	41 per 1 000	21 fewer per 1 000 (32 fewer to 2 more)

***The risk in the intervention group (and its 95% confidence interval) is based on the assumed risk in the comparison group and the relative effect of the intervention (and its 95% CI). CI: Confidence interval; OR: Odds ratio; HR: Hazard Ratio**

GRADE Working Group grades of evidence
High certainty: We are very confident that the true effect lies close to that of the estimate of the effect
Moderate certainty: We are moderately confident in the effect estimate: The true effect is likely to be close to the estimate of the effect, but there is a possibility that it is substantially different. Low certainty: Our confidence in the effect estimate is limited: The true effect may be substantially different from the estimate of the effect. Very low certainty: We have very little confidence in the effect estimate: The true effect is likely to be substantially different from the estimate of effect

Patient or population: relapsed/refractory multiple myeloma

Intervention: daratumumab + backbone therapy

Comparison: backbone therapy

Outcomes	№ of participants (studies)	Certainty of the evidence (GRADE)	Relative effect (95% CI)	Anticipated absolute effects	
				Risk with backbone therapy	Risk difference with daratumumab + backbone therapy
**second primary cancer**	1048 (2 RCTs)	⨁⨁◯◯ LOW	OR 1.26 (0.57 to 2.80)	21 per 1 000	5 more per 1 000 (9 fewer to 36 more)
**acute cardiac failure**	461 (1 RCT)	⨁⨁◯◯ LOW	OR 0.69 (0.35 to 1.35)	105 per 1 000	30 fewer per 1 000 (65 fewer to 32 more)
**ischaemic heart disease**	461 (1 RCT)	⨁⨁◯◯ LOW	OR 1.30 (0.46 to 3.73)	33 per 1 000	9 more per 1 000 (17 fewer to 79 more)
**renal failure**	461 (1 RCT)	⨁⨁◯◯ LOW	OR 0.73 (0.34 to 1.56)	78 per 1 000	20 fewer per 1 000 (50 fewer to 39 more)

***The risk in the intervention group (and its 95% confidence interval) is based on the assumed risk in the comparison group and the relative effect of the intervention (and its 95% CI).**
CI: Confidence interval; OR: Odds ratio; HR: Hazard Ratio

GRADE Working Group grades of evidence
High certainty: We are very confident that the true effect lies close to that of the estimate of the effect Moderate certainty: We are moderately confident in the effect estimate: The true effect is likely to be close to the estimate of the effect, but there is a possibility that it is substantially different. Low certainty: Our confidence in the effect estimate is limited: The true effect may be substantially different from the estimate of the effect. Very low certainty: We have very little confidence in the effect estimate: The true effect is likely to be substantially different from the estimate of effect

Patient or population: relapsed/refractory multiple myeloma

_Intervention: daratumumab + backbone therapy

Comparison: backbone therapy

Certainty assessment							№ of patients		Effect		Certainty	Importance
**№ of studies**	Study design	Risk of bias	Inconsistency	Indirectness	Imprecision	Other considerations	daratumumab + backbone therapy	backbone therapy	Relative (95% CI)	Absolute (95% CI)		
**minimal residual disease negativity**												
3	randomised trials	not serious	not serious	serious	not serious	none	87/600 (14.5%)	10/370 (2.7%)	OR 5.43 (2.76 to 10.66)	104 more per 1 000 (from 44 more to 201 more)	⨁⨁⨁◯ MODERATE	IMPORTANT
**stringent complete respone**												
4	randomised trials	not serious	not serious	serious	not serious	none	98/825 (11.9%)	30/746 (4.0%)	OR 3.08 (2.00 to 4.76)	74 more per 1 000 (from 37 more to 126 more)	⨁⨁⨁◯ MODERATE	IMPORTANT
**complete response or better**												
4	randomised trials	not serious	not serious	serious	not serious	none	249/825 (30.2%)	87/746 (11.7%)	OR 3.50 (2.33 to 5.25)	199 more per 1 000 (from 119 more to 293 more)	⨁⨁⨁◯ MODERATE	IMPORTANT
**death or disease progression**												
5	randomised trials	not serious	serious	serious	not serious	none	476/1141 (41.7%)	507/907 (55.9%)	HR 0.50 (0.37 to 0.67)	223 fewer per 1 000 (from 298 fewer to 137 fewer)	⨁⨁◯◯ LOW	CRITICAL
**all grade thrombocytopenia**												
4	randomised trials	not serious	serious	serious	not serious	none	461/989 (46.6%)	276/752 (36.7%)	OR 1.58 (1.03 to 2.43)	111 more per 1 000 (from 7 more to 218 more)	⨁⨁◯◯ LOW	IMPORTANT
**grade 3-4 thrombocytopenia**												
4	randomised trials	not serious	not serious	serious	not serious	none	293/989 (29.6%)	166/754 (22.0%)	OR 1.47 (1.10 to 1.98)	74 more per 1 000 (from 17 more to 140 more)	⨁⨁⨁◯ MODERATE	IMPORTANT

Patient or population: relapsed/refractory multiple myeloma

_Intervention: daratumumab + backbone therapy

Comparison: backbone therapy

Certainty assessment							№ of patients		Effect		Certainty	Importance
**№ of studies**	Study design	Risk of bias	Inconsistency	Indirectness	Imprecision	Other considerations	daratumumab + backbone therapy	backbone therapy	Relative (95% CI)	Absolute (95% CI)		
**all grade lymphopenia**												
4	randomised trials	not serious	serious	serious	serious	none	156/989 (15.8%)	71/754 (9.4%)	OR 1.54 (0.89 to 2.66)	44 more per 1 000 (from 10 fewer to 123 more)	⨁◯◯◯ VERY LOW	IMPORTANT
**grade 3-4 lymphopenia**												
4	randomised trials	not serious	serious	serious	not serious	none	120/989 (12.1%)	47/754 (6.2%)	OR 1.75 (1.02 to 2.99)	43 more per 1 000 (from 1 more to 105 more)	⨁⨁◯◯ LOW	IMPORTANT
**all grade neutropenia**												
4	randomised trials	not serious	not serious	serious	not serious	none	322/989 (32.6%)	175/754 (23.3%)	OR 1.98 (1.56 to 2.52)	142 more per 1 000 (from 88 more to 200 more)	⨁⨁⨁◯ MODERATE	IMPORTANT
**grade 3-4 neutropenia**												
4	randomised trials	not serious	serious	serious	not serious	none	227/989 (23.0%)	127/754 (16.8%)	OR 1.84 (0.98 to 3.46)	104 more per 1 000 (from 3 fewer to 246 more)	⨁⨁◯◯ LOW	IMPORTANT
**all grade anaemia**												
4	randomised trials	not serious	not serious	serious	not serious	none	361/989 (36.5%)	273/754 (36.3%)	OR 0.89 (0.72 to 1.10)	26 fewer per 1 000 (from 72 fewer to 22 more)	⨁⨁⨁◯ MODERATE	IMPORTANT
**grade 3-4 anaemia**												
4	randomised trials	not serious	serious	serious	serious	none	156/989 (15.8%)	128/754 (17.0%)	OR 0.91 (0.62 to 1.34)	13 fewer per 1 000 (from 58 fewer to 46 more)	⨁◯◯◯ VERY LOW	IMPORTANT

Patient or population: relapsed/refractory multiple myeloma

Intervention: daratumumab + backbone therapy

Comparison: backbone therapy

Certainty assessment							№ of patients		Effect		Certainty	Importance
**№ of studies**	Study design	Risk of bias	Inconsistency	Indirectness	Imprecision	Other considerations	daratumumab + backbone therapy	backbone therapy	Relative (95% CI)	Absolute (95% CI)		
**all grade hypertension**												
3	randomised trials	not serious	serious	serious	not serious	none	142/703 (20.2%)	53/471 (11.3%)	OR 2.21 (0.92 to 5.29)	109 more per 1 000 (from 8 fewer to 293 more)	⨁⨁◯◯ LOW	IMPORTANT
**grade 3-4 hypertension**												
3	randomised trials	not serious	serious	serious	not serious	none	87/703 (12.4%)	24/471 (5.1%)	OR 3.21 (0.97 to 10.61)	98 more per 1 000 (from 1 fewer to 317 more)	⨁⨁◯◯ LOW	IMPORTANT
**all grade neuropathy**												
2	randomised trials	not serious	not serious	serious	serious	none	168/551 (30.5%)	102/390 (26.2%)	OR 1.24 (0.93 to 1.65)	44 more per 1 000 (from 14 fewer to 107 more)	⨁⨁◯◯ LOW	IMPORTANT
**grade 3-4 neuropathy**												
2	randomised trials	not serious	not serious	serious	serious	none	11/551 (2.0%)	16/390 (4.1%)	OR 0.48 (0.22 to 1.04)	21 fewer per 1 000 (from 32 fewer to 2 more)	⨁⨁◯◯ LOW	IMPORTANT

Patient or population: relapsed/refractory multiple myeloma

Intervention: daratumumab + backbone therapy

Comparison: backbone therapy

Certainty assessment							№ of patients		Effect		Certainty	Importance
**№ of studies**	Study design	Risk of bias	Inconsistency	Indirectness	Imprecision	Other considerations	daratumumab + backbone therapy	backbone therapy	Relative (95% CI)	Absolute (95% CI)		
**second primary cancer**												
2	randomised trials	not serious	not serious	serious	serious	none	14/528 (2.7%)	11/520 (2.1%)	OR 1.26 (0.57 to 2.80)	5 more per 1 000 (from 9 fewer to 36 more)	⊕⊕◯◯ LOW	IMPORTANT
**Acute cardiac failure**												
1	randomised trials	not serious	not serious	not serious	very serious	none	23/308 (7.5%)	16/153 (10.5%)	OR 0.69 (0.35 to 1.35)	30 fewer per 1 000 (from 65 fewer to 32 more)	⊕⊕◯◯ LOW	IMPORTANT
**Ischaemic heart disease**												
1	randomised trials	not serious	not serious	not serious	very serious	none	13/308 (4.2%)	5/153 (3.3%)	OR 1.30 (0.46 to 3.73)	9 more per 1 000 (from 17 fewer to 79 more)	⊕⊕◯◯ LOW	IMPORTANT
**Renal failure**												
1	randomised trials	not serious	not serious	not serious	very serious	none	18/308 (5.8%)	12/153 (7.8%)	OR 0.73 (0.34 to 1.56)	20 fewer per 1 000 (from 50 fewer to 39 more)	⊕⊕◯◯ LOW	IMPORTANT

Patient or population: relapsed/refractory multiple myeloma with standard cytogenetic risk

Intervention: daratumumab + backbone therapy

Comparison: backbone therapy

Outcomes	№ of participants (studies)	Certainty of the evidence (GRADE)	Relative effect (95% CI)	Anticipated absolute effects	
				Risk with backbone therapy	Risk difference with daratumumab + backbone therapy
**minimal residual disease negativity (MRD negativity)**	631 (2 RCTs)	⊕◯◯◯ VERY LOW	OR 29.88 (4.04 to 220.78)	3 per 1 000	86 more per 1 000 (10 more to 417 more)
**stringent complete response (sCR)**	631 (2 RCTs)	⊕◯◯◯ VERY LOW	OR 3.12 (1.93 to 5.04)	85 per 1 000	140 more per 1 000 (67 more to 234 more)
**complete response or better (CR or better)**	631 (2 RCTs)	⊕◯◯◯ VERY LOW	OR 3.78 (2.63 to 5.43)	183 per 1 000	275 more per 1 000 (188 more to 366 more)
**death or disease progression**	1076 (5 RCTs)	⊕⊕◯◯ LOW	HR 0.38 (0.29 to 0.50)	722 per 1 000	337 fewer per 1 000 (412 fewer to 249 fewer)

***The risk in the intervention group (and its 95% confidence interval) is based on the assumed risk in the comparison group and the relative effect of the intervention (and its 95% CI).**
CI: Confidence interval; OR: Odds ratio; HR: Hazard Ratio

GRADE Working Group grades of evidence
High certainty: We are very confident that the true effect lies close to that of the estimate of the effect
Moderate certainty: We are moderately confident in the effect estimate: The true effect is likely to be close to the estimate of the effect, but there is a possibility that it is substantially different
Low certainty: Our confidence in the effect estimate is limited: The true effect may be substantially different from the estimate of the effect
Very low certainty: We have very little confidence in the effect estimate: The true effect is likely to be substantially different from the estimate of effect

Patient or population: relapsed/refractory multiple myeloma with standard cytogenetic risk

Intervention: daratumumab + backbone therapy

Comparison: backbone therapy

Outcomes	№ of participants (studies)	Certainty of the evidence (GRADE)	Relative effect (95% CI)	Anticipated absolute effects	
				Risk with backbone therapy	Risk difference with daratumumab + backbone therapy
**all grade thrombocytopenia**	641 (2 RCTs)	⨁⨁◯◯ LOW	OR 1.48 (1.07 to 2.04)	333 per 1 000	92 more per 1 000 (15 more to 172 more)
**grade 3-4 thrombocytopenia**	641 (2 RCTs)	⨁⨁◯◯ LOW	OR 1.32 (0.92 to 1.90)	221 per 1 000	51 more per 1 000 (14 fewer to 129 more)
**all grade lymphopenia**	641 (2 RCTs)	⨁⨁◯◯ LOW	OR 1.06 (0.61 to 1.88)	80 per 1 000	4 more per 1 000 (30 fewer to 61 more)
**grade 3-4 lymphopenia**	641 (2 RCTs)	⨁⨁◯◯ LOW	OR 2.16 (1.01 to 4.65)	32 per 1 000	35 more per 1 000 (0 fewer to 101 more)
**all grade neutropenia**	641 (2 RCTs)	⨁⨁◯◯ LOW	OR 1.77 (1.28 to 2.45)	308 per 1 000	133 more per 1 000 (55 more to 214 more)
**grade 3-4 neutropenia**	641 (2 RCTs)	⨁⨁◯◯ LOW	OR 1.91 (1.36 to 2.69)	240 per 1 000	136 more per 1 000 (61 more to 219 more)

***The risk in the intervention group (and its 95% confidence interval) is based on the assumed risk in the comparison group and the relative effect of the intervention (and its 95% CI).**
CI: Confidence interval; OR: Odds ratio; HR: Hazard Ratio

GRADE Working Group grades of evidence
High certainty: We are very confident that the true effect lies close to that of the estimate of the effect
Moderate certainty: We are moderately confident in the effect estimate: The true effect is likely to be close to the estimate of the effect, but there is a possibility that it is substantially different. Low certainty: Our confidence in the effect estimate is limited: The true effect may be substantially different from the estimate of the effect. Very low certainty: We have very little confidence in the effect estimate: The true effect is likely to be substantially different from the estimate of effect

Patient or population: relapsed/refractory multiple myeloma with standard cytogenetic risk
Intervention: daratumumab + backbone therapy
Comparison: backbone therapy

Outcomes	№ of participants (studies)	Certainty of the evidence (GRADE)	Relative effect (95% CI)	Anticipated absolute effects	
				Risk with backbone therapy	Risk difference with daratumumab + backbone therapy
**all grade anaemia**	641 (2 RCTs)	⊕⊕◯◯ LOW	OR 1.11 (0.80 to 1.53)	330 per 1 000	23 more per 1 000 (47 fewer to 100 more)
**grade 3-4 anaemia**	641 (2 RCTs)	⊕⊕◯◯ LOW	OR 0.96 (0.64 to 1.44)	176 per 1 000	6 fewer per 1 000 (56 fewer to 59 more)
**all grade peripheral neuropathy**	270 (1 RCT)	⊕⊕◯◯ LOW	OR 1.72 (1.06 to 2.80)	368 per 1 000	132 more per 1 000 (14 more to 252 more)
**grade 3-4 peripheral neuropathy**	273 (1 RCT)	⊕⊕◯◯ LOW	OR 0.48 (0.14 to 1.64)	59 per 1 000	30 fewer per 1 000 (50 fewer to 34 more)
**all grade hypertension**	641 (2 RCTs)	⊕⊕◯◯ LOW	OR 3.22 (1.14 to 9.13)	42 per 1 000	81 more per 1 000 (6 more to 242 more)
**grade 3-4 hypertension**	641 (2 RCTs)	⊕⊕◯◯ LOW	OR 4.58 (1.30 to 16.09)	10 per 1 000	33 more per 1 000 (3 more to 125 more)

*The risk in the intervention group (and its 95% confidence interval) is based on the assumed risk in the comparison group and the relative effect of the intervention (and its 95% CI).
CI: Confidence interval; OR: Odds ratio; HR: Hazard Ratio

GRADE Working Group grades of evidence
High certainty: We are very confident that the true effect lies close to that of the estimate of the effect
Moderate certainty: We are moderately confident in the effect estimate: The true effect is likely to be close to the estimate of the effect, but there is a possibility that it is substantially different. Low certainty: Our confidence in the effect estimate is limited: The true effect may be substantially different from the estimate of the effect. Very low certainty: We have very little confidence in the effect estimate: The true effect is likely to be substantially different from the estimate of effect

Patient or population: relapsed/refractory multiple myeloma with standard cytogenetic risk

Intervention: daratumumab + backbone therapy

Comparison: backbone therapy

Outcomes	№ of participants (studies)	Certainty of the evidence (GRADE)	Relative effect (95% CI)	Anticipated absolute effects	
				Risk with backbone therapy	Risk difference with daratumumab + backbone therapy
**Second primary cancer**	Not reported.				
**Acute cardiac failure - not reported**	Not reported.				
**Ischaemic heart diease - not reported**	Not reported.				
**Renal failure - not reported**	Not reported.				

***The risk in the intervention group (and its 95% confidence interval) is based on the assumed risk in the comparison group and the relative effect of the intervention (and its 95% CI).**

CI: Confidence interval; HR: Hazard Ratio; OR: Odds ratio

GRADE Working Group grades of evidence
High certainty: We are very confident that the true effect lies close to that of the estimate of the effect
Moderate certainty: We are moderately confident in the effect estimate: The true effect is likely to be close to the estimate of the effect, but there is a possibility that it is substantially different
Low certainty: Our confidence in the effect estimate is limited: The true effect may be substantially different from the estimate of the effect
Very low certainty: We have very little confidence in the effect estimate: The true effect is likely to be substantially different from the estimate of effect

Patient or population: relapsed/refractory multiple myeloma with standard cytogenetic risk

Intervention: daratumumab + backbone therapy

Comparison: backbone therapy

Certainty assessment							№ of patients		Effect		Certainty	Importance
**№ of studies**	Study design	Risk of bias	Inconsistency	Indirectness	Imprecision	Other considerations	daratumumab + backbone therapy	backbone therapy	Relative (95% CI)	Absolute (95% CI)		
**minimal residual disease negativity**												
2	randomised trials	not serious	not serious	serious	very serious	none	29/325 (8.9%)	1/306 (0.3%)	OR 29.88 (4.04 to 220.78)	86 more per 1 000 (from 10 more to 417 more)	⊕◯◯◯ VERY LOW	IMPORTANT
**stringent complete response**												
2	randomised trials	not serious	not serious	serious	very serious	none	73/325 (22.5%)	26/306 (8.5%)	OR 3.12 (1.93 to 5.04)	140 more per 1 000 (from 67 more to 234 more)	⊕◯◯◯ VERY LOW	IMPORTANT
**complete response or better**												
2	randomised trials	not serious	not serious	serious	very serious	none	149/325 (45.8%)	56/306 (18.3%)	OR 3.78 (2.63 to 5.43)	275 more per 1 000 (from 188 more to 366 more)	⊕◯◯◯ VERY LOW	IMPORTANT
**death or disease progression**												
5	randomised trials	not serious	serious	serious	not serious	none	239/594 (40.2%)	348/482 (72.2%)	HR 0.38 (0.29 to 0.50)	337 fewer per 1 000 (from 412 fewer to 249 fewer)	⊕⊕◯◯ LOW	CRITICAL
**all grade thrombocytopenia**												
2	randomised trials	not serious	not serious	serious	serious	none	140/329 (42.6%)	104/312 (33.3%)	OR 1.48 (1.07 to 2.04)	92 more per 1 000 (from 15 more to 172 more)	⊕⊕◯◯ LOW	IMPORTANT
**grade 3-4 thrombocytopenia**												
2	randomised trials	not serious	not serious	serious	serious	none	90/329 (27.4%)	69/312 (22.1%)	OR 1.32 (0.92 to 1.90)	51 more per 1 000 (from 14 fewer to 129 more)	⊕⊕◯◯ LOW	IMPORTANT

Patient or population: relapsed/refractory multiple myeloma with standard cytogenetic risk

Intervention: daratumumab + backbone therapy

Comparison: backbone therapy

Certainty assessment							№ of patients		Effect		Certainty	Importance
**№ of studies**	Study design	Risk of bias	Inconsistency	Indirectness	Imprecision	Other considerations	daratumumab + backbone therapy	backbone therapy	Relative (95% CI)	Absolute (95% CI)		
**all grade lymphopenia**												
2	randomised trials	not serious	not serious	serious	serious	none	28/329 (8.5%)	25/312 (8.0%)	OR 1.06 (0.61 to 1.88)	4 more per 1 000 (from 30 fewer to 61 more)	⊕⊕◯◯ LOW	IMPORTANT
**grade 3-4 lymphopenia**												
2	randomised trials	not serious	not serious	serious	serious	none	22/329 (6.7%)	10/312 (3.2%)	OR 2.16 (1.01 to 4.65)	35 more per 1 000 (from 0 fewer to 101 more)	⊕⊕◯◯ LOW	IMPORTANT
**all grade neutropenia**												
2	randomised trials	not serious	not serious	serious	serious	none	145/329 (44.1%)	96/312 (30.8%)	OR 1.77 (1.28 to 2.45)	133 more per 1 000 (from 55 more to 214 more)	⊕⊕◯◯ LOW	IMPORTANT
**grade 3-4 neutropenia**												
2	randomised trials	not serious	not serious	serious	serious	none	124/329 (37.7%)	75/312 (24.0%)	OR 1.91 (1.36 to 2.69)	136 more per 1 000 (from 61 more to 219 more)	⊕⊕◯◯ LOW	IMPORTANT
**all grade anaemia**												
2	randomised trials	not serious	not serious	serious	serious	none	116/329 (35.3%)	103/312 (33.0%)	OR 1.11 (0.80 to 1.53)	23 more per 1 000 (from 47 fewer to 100 more)	⊕⊕◯◯ LOW	IMPORTANT
**grade 3-4 anaemia**												
2	randomised trials	not serious	not serious	serious	serious	none	56/329 (17.0%)	55/312 (17.6%)	OR 0.96 (0.64 to 1.44)	6 fewer per 1 000 (from 56 fewer to 59 more)	⊕⊕◯◯ LOW	IMPORTANT

Patient or population: relapsed/refractory multiple myeloma with standard cytogenetic risk

Intervention: daratumumab + backbone therapy

Comparison: backbone therapy

Certainty assessment							№ of patients		Effect		Certainty	Importance
**№ of studies**	Study design	Risk of bias	Inconsistency	Indirectness	Imprecision	Other considerations	daratumumab + backbone therapy	backbone therapy	Relative (95% CI)	Absolute (95% CI)		
**all grade peripheral neuropathy**												
1	randomised trials	not serious	not serious	not serious	very serious	none	67/134 (50.0%)	50/136 (36.8%)	OR 1.72 (1.06 to 2.80)	132 more per 1 000 (from 14 more to 252 more)	⨁⨁◯◯ LOW	IMPORTANT
**grade 3-4 peripheral neuropathy**												
1	randomised trials	not serious	not serious	not serious	very serious	none	4/137 (2.9%)	8/136 (5.9%)	OR 0.48 (0.14 to 1.64)	30 fewer per 1 000 (from 50 fewer to 34 more)	⨁⨁◯◯ LOW	IMPORTANT
**all grade hypertension**												
2	randomised trials	not serious	not serious	not serious	very serious	none	27/329 (8.2%)	13/312 (4.2%)	OR 3.22 (1.14 to 9.13)	81 more per 1 000 (from 6 more to 242 more)	⨁⨁◯◯ LOW	IMPORTANT
**grade 3-4 hypertension**												
2	randomised trials	not serious	not serious	not serious	very serious	none	14/329 (4.3%)	3/312 (1.0%)	OR 4.58 (1.30 to 16.09)	33 more per 1 000 (from 3 more to 125 more)	⨁⨁◯◯ LOW	IMPORTANT

Second primary cancer, acute cardiac failure, ischaemic heart failure, and renal failure were not reported.

Patient or population: relapsed/refractory multiple myeloma with high cytogenetic risk

Intervention: daratumumab + backbone therapy

Comparison: backbone therapy

Outcomes	№ of participants (studies)	Certainty of the evidence (GRADE)	Relative effect (95% CI)	Anticipated absolute effects	
				Risk with backbone therapy	Risk difference with daratumumab + backbone therapy
**minimal residual disease negativity (MRD negativity)**	142 (2 RCTs)	⊕◯◯◯ VERY LOW	OR 18.48 (2.38 to 143.67)	0 per 1 000	0 fewer per 1 000 (0 fewer to 0 fewer)
**stringent complete response (sCR)**	142 (2 RCTs)	⊕◯◯◯ VERY LOW	OR 8.74 (0.45 to 168.00)	15 per 1 000	101 more per 1 000 (8 fewer to 700 more)
**complete response or better (CR or better)**	73 (1 RCT)	⊕⊕◯◯ LOW	OR 6.29 (1.28 to 30.82)	59 per 1 000	223 more per 1 000 (15 more to 599 more)
**death or disease progression**	366 (5 RCTs)	⊕⊕⊕◯ MODERATE	HR 0.52 (0.35 to 0.76)	734 per 1 000	236 fewer per 1 000 (363 fewer to 100 fewer)

***The risk in the intervention group (and its 95% confidence interval) is based on the assumed risk in the comparison group and the relative effect of the intervention (and its 95% CI).**
CI: Confidence interval; OR: Odds ratio; HR: Hazard Ratio

GRADE Working Group grades of evidence
High certainty: We are very confident that the true effect lies close to that of the estimate of the effect
Moderate certainty: We are moderately confident in the effect estimate: The true effect is likely to be close to the estimate of the effect, but there is a possibility that it is substantially different. Low certainty: Our confidence in the effect estimate is limited: The true effect may be substantially different from the estimate of the effect. Very low certainty: We have very little confidence in the effect estimate: The true effect is likely to be substantially different from the estimate of effect

Patient or population: relapsed/refractory multiple myeloma with high cytogenetic risk
Intervention: daratumumab + backbone therapy
Comparison: backbone therapy

Outcomes	№ of participants (studies)	Certainty of the evidence (GRADE)	Relative effect (95% CI)	Anticipated absolute effects	
				Risk with backbone therapy	Risk difference with daratumumab + backbone therapy
**all grade thrombocytopenia**	143 (2 RCTs)	⊕⊕◯◯ LOW	OR 1.23 (0.64 to 2.38)	441 per 1 000	51 more per 1 000 (106 fewer to 211 more)
**grade 3-4 thrombocytopenia**	143 (2 RCTs)	⊕⊕◯◯ LOW	OR 1.18 (0.59 to 2.35)	324 per 1 000	37 more per 1 000 (103 fewer to 206 more)
**all grade lymphopenia**	143 (2 RCTs)	⊕⊕◯◯ LOW	OR 1.18 (0.42 to 3.39)	103 per 1 000	16 more per 1 000 (57 fewer to 177 more)
**grade 3-4 lymphopenia**	143 (2 RCTs)	⊕⊕◯◯ LOW	OR 1.23 (0.41 to 3.78)	88 per 1 000	18 more per 1 000 (50 fewer to 180 more)
**all grade neutropenia**	143 (2 RCTs)	⊕⊕◯◯ LOW	OR 1.58 (0.79 to 3.14)	309 per 1 000	105 more per 1 000 (48 fewer to 275 more)
**grade 3-4 neutropenia**	143 (2 RCTs)	⊕⊕◯◯ LOW	OR 1.23 (0.59 to 2.55)	265 per 1 000	42 more per 1 000 (90 fewer to 214 more)

***The risk in the intervention group (and its 95% confidence interval) is based on the assumed risk in the comparison group and the relative effect of the intervention (and its 95% CI).**
CI: Confidence interval; OR: Odds ratio; HR: Hazard Ratio

GRADE Working Group grades of evidence
High certainty: We are very confident that the true effect lies close to that of the estimate of the effect
Moderate certainty: We are moderately confident in the effect estimate: The true effect is likely to be close to the estimate of the effect, but there is a possibility that it is substantially different. Low certainty: Our confidence in the effect estimate is limited: The true effect may be substantially different from the estimate of the effect. Very low certainty: We have very little confidence in the effect estimate: The true effect is likely to be substantially different from the estimate of effect

Patient or population: relapsed/refractory multiple myeloma with high cytogenetic risk
Intervention: daratumumab + backbone therapy
Comparison: backbone therapy

Outcomes	№ of participants (studies)	Certainty of the evidence (GRADE)	Relative effect (95% CI)	Anticipated absolute effects	
				Risk with backbone therapy	Risk difference with daratumumab + backbone therapy
**all grade anaemia**	143 (2 RCTs)	⨁⨁◯◯ LOW	OR 0.49 (0.24 to 0.99)	426 per 1 000	159 fewer per 1 000 (275 fewer to 2 fewer)
**grade 3-4 anaemia**	143 (2 RCTs)	⨁⨁◯◯ LOW	OR 0.56 (0.24 to 1.31)	235 per 1 000	88 fewer per 1 000 (167 fewer to 52 more)
**all grade peripheral neuropathy**	74 (1 RCT)	⨁⨁◯◯ LOW	OR 1.97 (0.78 to 5.01)	382 per 1 000	167 more per 1 000 (57 fewer to 374 more)
**grade 3-4 peripheral neuropathy**	74 (1 RCT)	⨁⨁◯◯ LOW	OR 0.39 (0.07 to 2.30)	118 per 1 000	68 fewer per 1 000 (108 fewer to 117 more)
**all grade hypertension**	143 (2 RCTs)	⨁◯◯◯ VERY LOW	OR 4.54 (1.23 to 16.71)	44 per 1 000	129 more per 1 000 (10 more to 391 more)
**grade 3-4 hypertension**	143 (2 RCTs)	⨁⨁◯◯ LOW	OR 12.81 (0.71 to 231.89)	0 per 1 000	0 fewer per 1 000 (0 fewer to 0 fewer)

***The risk in the intervention group (and its 95% confidence interval) is based on the assumed risk in the comparison group and the relative effect of the intervention (and its 95% CI).**
CI: Confidence interval; OR: Odds ratio; HR: Hazard Ratio

GRADE Working Group grades of evidence
High certainty: We are very confident that the true effect lies close to that of the estimate of the effect
Moderate certainty: We are moderately confident in the effect estimate: The true effect is likely to be close to the estimate of the effect, but there is a possibility that it is substantially different. Low certainty: Our confidence in the effect estimate is limited: The true effect may be substantially different from the estimate of the effect. Very low certainty: We have very little confidence in the effect estimate: The true effect is likely to be substantially different from the estimate of effect

Patient or population: relapsed/refractory multiple myeloma with high cytogenetic risk

Intervention: daratumumab + backbone therapy

Comparison: backbone therapy

Outcomes	№ of participants (studies)	Certainty of the evidence (GRADE)	Relative effect (95% CI)	Anticipated absolute effects	
				Risk with backbone therapy	Risk difference with daratumumab + backbone therapy
**Second primary cancer**	Not reported.				
**Acute cardiac failure - not reported**	Not reported.				
**Ischaemic heart diease - not reported**	Not reported.				
**Renal failure - not reported**	Not reported.				

***The risk in the intervention group (and its 95% confidence interval) is based on the assumed risk in the comparison group and the relative effect of the intervention (and its 95% CI).**

CI: Confidence interval; HR: Hazard Ratio; OR: Odds ratio

GRADE Working Group grades of evidence
High certainty: We are very confident that the true effect lies close to that of the estimate of the effect
Moderate certainty: We are moderately confident in the effect estimate: The true effect is likely to be close to the estimate of the effect, but there is a possibility that it is substantially different
Low certainty: Our confidence in the effect estimate is limited: The true effect may be substantially different from the estimate of the effect
Very low certainty: We have very little confidence in the effect estimate: The true effect is likely to be substantially different from the estimate of effect

Patient or population: relapsed/refractory multiple myeloma with high cytogenetic risk

Intervention: daratumumab + backbone therapy

Comparison: backbone therapy

**Certainty assessment**							**№ of patients**		**Effect**		**Certainty**	**Importance**
**№ of studies**	Study design	Risk of bias	Inconsistency	Indirectness	Imprecision	Other considerations	daratumumab + backbone therapy	backbone therapy	Relative (95% CI)	Absolute (95% CI)		
**minimal residual disease negativity**												
2	randomised trials	not serious	not serious	serious	very serious	none	4/74 (5.4%)	0/68 (0.0%)	OR 18.48 (2.38 to 143.67)	0 fewer per 1 000 (from 0 fewer to 0 fewer)	⨁◯◯◯ VERY LOW	IMPORTANT
**stringent complete response**												
2	randomised trials	not serious	not serious	serious	very serious	none	14/74 (18.9%)	1/68 (1.5%)	OR 8.74 (0.45 to 168.00)	101 more per 1 000 (from 8 fewer to 700 more)	⨁◯◯◯ VERY LOW	IMPORTANT
**complete response or better**												
1	randomised trials	not serious	not serious	not serious	very serious	none	11/39 (28.2%)	2/34 (5.9%)	OR 6.29 (1.28 to 30.82)	223 more per 1 000 (from 15 more to 599 more)	⨁⨁◯◯ LOW	IMPORTANT
**death or disease progression**												
5	randomised trials	not serious	not serious	serious	not serious	none	103/208 (49.5%)	116/158 (73.4%)	HR 0.52 (0.35 to 0.76)	236 fewer per 1 000 (from 363 fewer to 100 fewer)	⨁⨁⨁◯ MODERATE	CRITICAL
**all grade thrombocytopenia**												
2	randomised trials	not serious	not serious	serious	serious	none	37/75 (49.3%)	30/68 (44.1%)	OR 1.23 (0.64 to 2.38)	51 more per 1 000 (from 106 fewer to 211 more)	⨁⨁◯◯ LOW	IMPORTANT
**grade 3-4 thrombocytopenia**												
2	randomised trials	not serious	not serious	serious	serious	none	27/75 (36.0%)	22/68 (32.4%)	OR 1.18 (0.59 to 2.35)	37 more per 1 000 (from 103 fewer to 206 more)	⨁⨁◯◯ LOW	IMPORTANT

Patient or population: relapsed/refractory multiple myeloma with high cytogenetic risk

Intervention: daratumumab + backbone therapy

Comparison: backbone therapy

Certainty assessment							№ of patients		Effect		Certainty	Importance
**№ of studies**	Study design	Risk of bias	Inconsistency	Indirectness	Imprecision	Other considerations	daratumumab + backbone therapy	backbone therapy	Relative (95% CI)	Absolute (95% CI)		
**all grade lymphopenia**												
2	randomised trials	not serious	not serious	serious	serious	none	9/75 (12.0%)	7/68 (10.3%)	OR 1.18 (0.42 to 3.39)	16 more per 1 000 (from 57 fewer to 177 more)	⊕⊕◯◯ LOW	IMPORTANT
**grade 3-4 lymphopenia**												
2	randomised trials	not serious	not serious	serious	serious	none	8/75 (10.7%)	6/68 (8.8%)	OR 1.23 (0.41 to 3.78)	18 more per 1 000 (from 50 fewer to 180 more)	⊕⊕◯◯ LOW	IMPORTANT
**all grade neutropenia**												
2	randomised trials	not serious	not serious	serious	serious	none	31/75 (41.3%)	21/68 (30.9%)	OR 1.58 (0.79 to 3.14)	105 more per 1 000 (from 48 fewer to 275 more)	⊕⊕◯◯ LOW	IMPORTANT
**grade 3-4 neutropenia**												
2	randomised trials	not serious	not serious	serious	serious	none	23/75 (30.7%)	18/68 (26.5%)	OR 1.23 (0.59 to 2.55)	42 more per 1 000 (from 90 fewer to 214 more)	⊕⊕◯◯ LOW	IMPORTANT
**all grade anaemia**												
2	randomised trials	not serious	not serious	serious	serious	none	20/75 (26.7%)	29/68 (42.6%)	OR 0.49 (0.24 to 0.99)	159 fewer per 1 000 (from 275 fewer to 2 fewer)	⊕⊕◯◯ LOW	IMPORTANT
**grade 3-4 anaemia**												
2	randomised trials	not serious	not serious	serious	serious	none	11/75 (14.7%)	16/68 (23.5%)	OR 0.56 (0.24 to 1.31)	88 fewer per 1 000 (from 167 fewer to 52 more)	⊕⊕◯◯ LOW	IMPORTANT

Patient or population: relapsed/refractory multiple myeloma with high cytogenetic risk

Intervention: daratumumab + backbone therapy

Comparison: backbone therapy

Certainty assessment							№ of patients		Effect		Certainty	Importance
**№ of studies**	Study design	Risk of bias	Inconsistency	Indirectness	Imprecision	Other considerations	daratumumab + backbone therapy	backbone therapy	Relative (95% CI)	Absolute (95% CI)		
**all grade peripheral neuropathy**												
1	randomised trials	not serious	not serious	not serious	very serious	none	22/40 (55.0%)	13/34 (38.2%)	OR 1.97 (0.78 to 5.01)	167 more per 1 000 (from 57 fewer to 374 more)	⨁⨁◯◯ LOW	IMPORTANT
**grade 3-4 peripheral neuropathy**												
1	randomised trials	not serious	not serious	not serious	very serious	none	2/40 (5.0%)	4/34 (11.8%)	OR 0.39 (0.07 to 2.30)	68 fewer per 1 000 (from 108 fewer to 117 more)	⨁⨁◯◯ LOW	IMPORTANT
**all grade hypertension**												
2	randomised trials	not serious	not serious	serious	very serious	none	13/75 (17.3%)	3/68 (4.4%)	OR 4.54 (1.23 to 16.71)	129 more per 1 000 (from 10 more to 391 more)	⨁◯◯◯ VERY LOW	IMPORTANT
**grade 3-4 hypertension**												
2	randomised trials	not serious	not serious	serious	serious	none	6/75 (8.0%)	0/68 (0.0%)	OR 12.81 (0.71 to 231.89)	0 fewer per 1 000 (from 0 fewer to 0 fewer)	⨁⨁◯◯ LOW	IMPORTANT

Second primary cancer, acute cardiac failure, ischaemic heart failure, and renal failure were not reported.
