# Supplementary material for: Addition of daratumumab to multiple myeloma backbone regimens significantly improves clinical outcomes: a systematic review and meta-analysis of randomised controlled trials
Source: Sci Rep. 2021 Nov 9;11:21916. doi: 10.1038/s41598-021-01440-x (PMC8578422; doi:10.1038/s41598-021-01440-x)
Supplement: Supplementary file 3 — Supplementary Information 3. [file 41598_2021_1440_MOESM3_ESM.pdf]

Supplementary Table: Detailed search strategy

**MEDLINE (via PubMed)**	
Date of search	2021.08.03.
Field of search	All Fields
Filters and restrictions	no
Search key	(daratumumab) OR (humax-CD38) OR (humax-CD 38) OR (Darzalex) OR (anti-CD38) OR (antiCD38) OR (L01XC24) OR (945721-28-8) OR (DB09331) OR (4Z63YK6E0E) OR (D10777)
Translation	"daratumumab"[Supplementary Concept] OR "daratumumab"[All Fields] OR "daratumumab"[Supplementary Concept] OR "daratumumab"[All Fields] OR "humax cd38"[All Fields] OR "daratumumab"[Supplementary Concept] OR "daratumumab"[All Fields] OR "daratumumab"[Supplementary Concept] OR "daratumumab"[All Fields] OR "darzalex"[All Fields] OR "anti-CD38"[All Fields] OR "antiCD38"[All Fields] OR "945721-28-8"[All Fields] OR "4z63yk6e0e"[EC/RN Number]
Number of records	1,266
**Embase**	
Date of search	2021.08.03.
Field of search	All fields
Filters and restrictions	no
Search key	(daratumumab) OR (humax-CD38) OR (humax-CD 38) OR (Darzalex) OR (anti-CD38) OR (antiCD38) OR (L01XC24) OR (945721-28-8) OR (DB09331) OR (4Z63YK6E0E) OR (D10777)
Translation	daratumumab OR 'humax cd38' OR ('humax cd' AND 38) OR darzalex OR 'anti cd38' OR anticd38 OR l01xc24 OR '945721 28 8' OR db09331 OR 4z63yk6e0e OR d10777
Number of records	4,330
**Cochrane Central Register of Controlled Trials (CENTRAL)**	
Date of search	2021.08.03.
Field of search	All text
Filters and restrictions	no
Search key	(daratumumab) OR (humax-CD38) OR (humax-CD 38) OR (Darzalex) OR (anti-CD38) OR (antiCD38) OR (L01XC24) OR (945721-28-8) OR (DB09331) OR (4Z63YK6E0E) OR (D10777)
Translation	(daratumumab) OR (humax-CD38) OR (humax-CD 38) OR (Darzalex) OR (anti-CD38) OR (antiCD38) OR (L01XC24) OR (945721-28-8) OR (DB09331) OR (4Z63YK6E0E) OR (D10777) in All Text - (Word variations have been searched)
Number of records	441

Supplementary Table: Detailed search strategy (continued)

**Scopus**	
Date of search	2021.08.03.
Field of search	All Fields
Filters and restrictions	no
Search key	(daratumumab) OR (humax-CD38) OR (humax-CD 38) OR (Darzalex) OR (anti-CD38) OR (antiCD38) OR (L01XC24) OR (945721-28-8) OR (DB09331) OR (4Z63YK6E0E) OR (D10777)
Translation	ALL ((daratumumab) OR (humax-cd38) OR (humax-cd 38) OR (darzalex) OR (anti-cd38) OR (anticd38) OR (l01xc24) OR (945721-28-8) OR (db09331) OR (4z63yk6e0e) OR (d10777))
Number of records	5,209
**Web of Science**	
Date of search	2021.08.03.
Field of search	All fields
Filters and restrictions	No
Search key	(daratumumab) OR (humax-CD38) OR (humax-CD 38) OR (Darzalex) OR (anti-CD38) OR (antiCD38) OR (L01XC24) OR (945721-28-8) OR (DB09331) OR (4Z63YK6E0E) OR (D10777)
Translation	ALL=((daratumumab) OR (humax-CD38) OR (humax-CD 38) OR (Darzalex) OR (anti-CD38) OR (antiCD38) OR (L01XC24) OR (945721-28-8) OR (DB09331) OR (4Z63YK6E0E) OR (D10777))
Number of records	2,275
